# Solid-State Interconversions: Unique 100 % Reversible Transformations between the Ground and Metastable States in Single-Crystals of a Series of Nickel(II) Nitro Complexes

**DOI:** 10.1002/chem.201302053

**Published:** 2014-03-18

**Authors:** Mark R Warren, Timothy L Easun, Simon K Brayshaw, Robert J Deeth, Michael W George, Andrew L Johnson, Stefanie Schiffers, Simon J Teat, Anna J Warren, John E Warren, Chick C Wilson, Christopher H Woodall, Paul R Raithby

**Affiliations:** [a] Department of Chemistry, University of BathBath, BA2 7AY (UK) E-mail: p.r.raithby@bath.ac.uk; [b] Department of Chemistry, University of NottinghamNottingham NG7 2RD (UK); [c] Department of Chemistry, University of WarwickCoventry CV4 7AL (UK); [d] Advanced Light Source, Lawrence Berkeley National LaboratoryBerkeley CA 94720 (USA); [e] STFC Daresbury LaboratoryDaresbury, Warrington WA4 4AD (UK); [f] The Research Complex at Harwell, Rutherford Appleton LaboratoryDidcot, Oxon, OX11 0FA (UK)

**Keywords:** linkage isomerism, metastable compounds, nickel, photocrystallography, single-crystals

## Abstract

The solid-state, low-temperature linkage isomerism in a series of five square planar group 10 phosphino nitro complexes have been investigated by a combination of photocrystallographic experiments, Raman spectroscopy and computer modelling. The factors influencing the reversible solid-state interconversion between the nitro and nitrito structural isomers have also been investigated, providing insight into the dynamics of this process. The *cis*-[Ni(dcpe)(NO_2_)_2_] (**1**) and *cis*-[Ni(dppe)(NO_2_)_2_] (**2**) complexes show reversible 100 % interconversion between the η^*1*^*-N*O_2_ nitro isomer and the η^*1*^*-O*NO nitrito form when single-crystals are irradiated with 400 nm light at 100 K. Variable temperature photocrystallographic studies for these complexes established that the metastable nitrito isomer reverted to the ground-state nitro isomer at temperatures above 180 K. By comparison, the related *trans* complex [Ni(PCy_3_)_2_(NO_2_)_2_] (**3**) showed 82 % conversion under the same experimental conditions at 100 K. The level of conversion to the metastable nitrito isomers is further reduced when the nickel centre is replaced by palladium or platinum. Prolonged irradiation of the *trans*-[Pd(PCy_3_)_2_(NO_2_)_2_] (**4**) and *trans*-[Pt(PCy_3_)_2_(NO_2_)_2_] (**5**) with 400 nm light gives reversible conversions of 44 and 27 %, respectively, consistent with the slower kinetics associated with the heavier members of group 10. The mechanism of the interconversion has been investigated by theoretical calculations based on the model complex [Ni(dmpe)Cl(NO_2_)].

## Introduction

Photocrystallography is a rapidly developing technique in which it is possible to determine the full three-dimensional structures of photoactivated metastable or transient species when a crystalline material is activated by light of the appropriate wavelength.[1] In the identification of short lifetime species laser-pump X-ray-probe techniques have been used effectively.[1a, 2] Single-crystal Laue X-ray diffraction techniques have been applied in the area of macromolecular crystallography to obtain snapshots of chemical processes, characterising species with lifetimes of a few hundreds of picoseconds.[3] In the area of “small molecule” crystallography, monochromatic X-ray radiation has been used to determine the structures of photoactivated molecular complexes with lifetimes in the microsecond range when the crystals are irradiated at temperatures below 30 K.[4] Because of the non-equilibrium nature of the pump-probe experiments, at any one time the number of photoactivated molecules within the crystal is small and thus only a few percent of “excited state” molecules are observed in the photocrystallographic experiments.

The situation can be less complex when studying metastable systems. In favourable cases, if the crystalline samples are maintained at low temperatures, the lifetime of the metastable state is essentially infinite, that is, longer than the duration of the X-ray experiment, and a non-pulsed light source, such as a continuous wave laser or a set of LEDs, can be used. Indeed, high percentage conversions to the metastable species, in the order of 20–50 %, have been observed.[5] The pioneering work on the structural determination of metastable species was performed by Woike et al. in the 1990s by investigating the structure of sodium nitroprusside,[6] and has continued with Coppens and Woike, and co-workers, separately, demonstrating that photogenerated, low-temperature linkage isomerism occurs in a wide range of transition metal nitro, nitrosyl and sulfur dioxide complexes in the solid state.[7] Raithby, Cole and co-workers have reported a new, photogenerated metastable coordination mode for the SO_2_ ligand in which the SO_2_ group is *η^1^-O*SO bound at temperatures below 100 K after irradiation with white light in single crystals containing the [Ru(NH_3_)_4_(H_2_O)(SO_2_)]^2+^ cation.[8]

However, even with metastable systems, high levels of conversion are rarely achieved. A rare exception is the 92 % single-crystal conversion of [Ru(py)_4_Cl(NO)][PF_6_]_2_**⋅**0.5 H_2_O from a *η^1^-N*O nitrosyl to a metastable *η^1^-O*N nitrosyl upon irradiation reported by Woike,[7n] however, excitation levels around 50 % are typical. If metastable linkage isomers are to find applications in devices[9] then reversible 100 % interconversion between isomers is essential. Various explanations have been put forward to justify why complete interconversion between isomeric forms is difficult to achieve. These include the introduction of unacceptable levels of strain within a crystal resulting from the photoinduced molecular rearrangement, that intermolecular interactions present within the crystal disfavour the molecular rearrangement,[10] or simply that the spectral overlap of the absorption bands of the ground state and the metastable state at the wavelength of irradiation is sufficient to set up an equilibrium between the two states so that complete conversion will not occur.[7n]

**Scheme 1:**
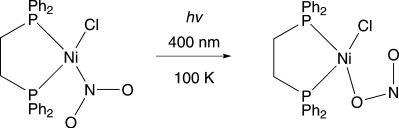
Photoactivated linkage isomerism of [Ni(dppe)Cl(*η^1^-N*O_2_)] into [Ni(dppe)Cl(*η^1^-O*NO)] in the single-crystal.

Recently, we commenced a systematic study to investigate the factors that would favour high levels of conversion between the linkage isomers. To establish whether steric strain within the crystal prevents high levels of interconversion upon photoactivation and rearrangement of the ambidentate ligands, we have adopted two approaches. In the first approach we immobilised the photoactive site into a metal organic framework, where it can sit in a cavity and rearrange without deforming the three-dimensional network. In this way it has been possible to convert an immobilised Mn(diimine)(CO)_3_Cl moiety from a *fac* to a *mer* isomer.[11] In the second approach, we have introduced bulky ligands around a metal centre, which allows a small ambidentate ligand to sit in a molecular “pocket” or “reaction cavity” that would permit ligand rearrangement without significantly disturbing the crystal packing. As a result of this approach, we recently reported the first example of a solid state 100 % conversion, reversible linkage isomerisation between a [Ni(dppe)Cl(η^1^-NO_2_)] nitro complex and the metastable [Ni(dppe)Cl(η^1^-ONO)] nitrito isomer upon irradiation with UV light at temperatures between 100–160 K (Scheme 1).[12] Warming the sample to above 160 K caused immediate reversion to the ground-state structure and the process could be repeated without apparent crystal decomposition.

Clearly, transferring the latter approach to other ligand systems and metals to enable us to build up a library of materials capable of undergoing 100 % reversible linkage isomerisation, would be a considerable step towards the goal of developing controllable metastable-state switchable materials. We now report the results of our investigations into the primary factors affecting the photoactivated transformation: the steric effects of the phosphine ligand, the electronic effect of changing the *cis*/*trans* configuration of the complexes, and the fundamental importance of the metal centre. We describe systematic Raman spectroscopic and single-crystal photocrystallographic studies on the symmetric dinitro complexes *cis*-[Ni(dcpe)(NO_2_)_2_] (**1**) and *cis*-[Ni(dppe)(NO_2_)_2_] (**2**), and investigate the impact of changing the phosphine from dppe (dppe=Ph_2_PCH_2_CH_2_PPh_2_) to dcpe (dcpe=Cy_2_PCH_2_CH_2_PCy_2_; Cy=cyclohexyl). We report the results of a computational study on the model complex [Ni(dmpe)Cl(NO_2_)] (dmpe=(CH_3_)_2_PCH_2_CH_2_P(CH_3_)_2_), undertaken to explore the feasibility of modelling the isomerisation process theoretically. Raman and photocrystallographic studies on the *trans* complex [Ni(PCy_3_)_2_(NO_2_)_2_] (**3**) are described, allowing assessment of the electronic effect of a *trans* arrangement of phosphines. The influence of the metal centre has also been investigated by the photocrystallographic study of the analogous complexes [Pd(PCy_3_)_2_(NO_2_)_2_] (**4**) and [Pt(PCy_3_)_2_(NO_2_)_2_] (**5**).

## Results and Discussion

**Scheme 2:**
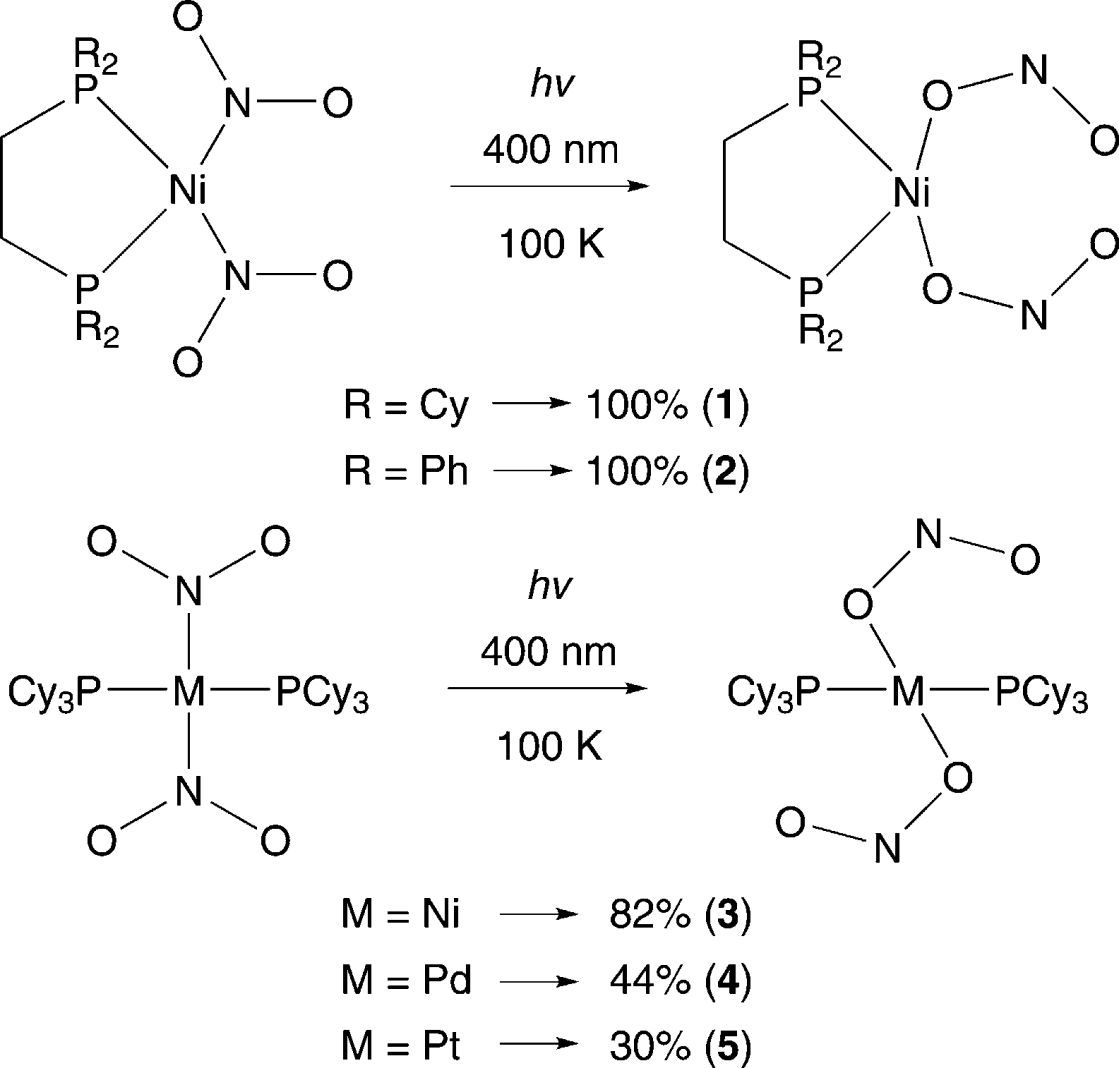
Photoisomerisation reactions of complexes 1–5 (photocrystallographically measured conversion shown).

The successful strategy behind the reversible 100 % photoisomerisation of [Ni(dppe)Cl(η^1^-NO_2_)] in the solid-state was the use of bulky phosphine ligands to allow the nitro group to sit in a sterically sheltered “reaction cavity” so that the interconversion could occur without disrupting the crystal packing. Following on from this success, we synthesised the complexes *cis*-[Ni(dcpe)(NO_2_)_2_] (**1**) and *cis*-[Ni(dppe)(NO_2_)_2_] (**2**). In **1** we replaced the dppe ligand by the chelating dcpe ligand, modifying the steric bulk around the metal centre by changing phenyl rings for cyclohexyl rings. In both complexes we replaced the chlorine ligand by a second nitro ligand in order to eliminate any complexity introduced by the electronic effects of the chlorine. Since electronic factors may play a significant role in the photoisomerisation process we also investigated a possible *trans* effect by studying the *trans* complex [Ni(PCy_3_)_2_(NO_2_)_2_] (**3**). We have also included an investigation of Pd^II^ and Pt^II^ analogues (Scheme 2).

As with the photocrystallographic investigation of [Ni(dppe)Cl(η^*1*^*-N*O_2_)][12] we initially monitored photoconversion between nitro/nitrito isomers within single crystals of **1–5** using variable temperature Raman spectroscopy. We first highlight this approach with [Ni(dcpe)(NO_2_)_2_] (**1**) and present here the results of spectroscopic experiments recorded at variable temperatures on the series of complexes.

**Figure 1:**
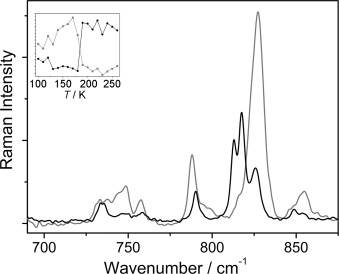
Raman spectra of 1 in the ground state (recorded at 250 K, black) and in the excited state (recorded at 100 K, grey). Inset: peak intensities recorded with increasing temperature at 814 cm^−1^ (black) and 828 cm^−1^ (grey).

Raman spectra of single crystals of **1** were recorded at 100 K before and after the crystal was irradiated with 400 nm light for 1 min (see the Experimental Section for details). Almost complete loss of the parent bands at 813 and 817 cm^−1^ (characteristic of the δ(NO_2_) deformation) and formation of a new band at 828 cm^−1^ was observed, consistent with a change in bonding from the nitro-(*η^1^-N*O_2_) to the nitrito-(*η^1^-O*NO) metastable species (Figure 1).[12] The Raman spectra of the crystal were then recorded on warming at 10 K intervals (Figure 1, inset) and a rapid reversal of the spectrum to that of the parent was evident at about 180 K, confirming the metastable nature of the photogenerated nitrito isomer. However, the Raman spectral changes do not definitively identify the nature of the nitrito-(*η^1^-O*NO) metastable species and the full photocrystallographic experiment is required to establish the bulk distribution of the possible conformers of the nitrito linkage isomer.

**Figure 2:**
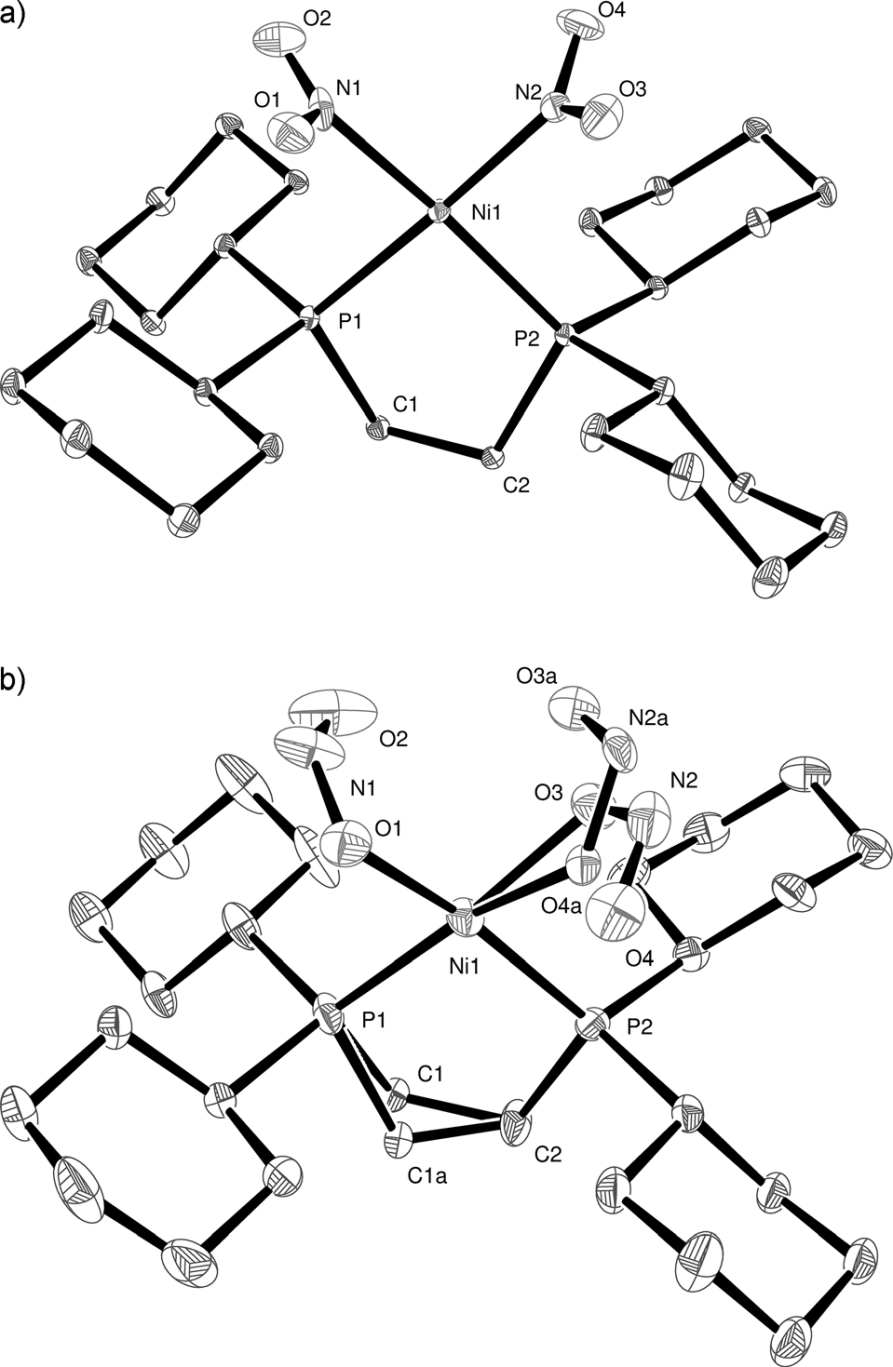
X-ray structure of: a) the ground state, and b) the metastable state of 1 with thermal ellipsoids set at 30 % probability and hydrogen atoms removed for clarity. In the metastable state the C1 and C1a atoms of the dcpe ligand exhibits disorder and one of the metastable nitrito ligands is disordered over two orientations.

Complex **1** crystallised in the monoclinic space group *P*2_1_/*c* together with one molecule of toluene solvent in the asymmetric unit. A high resolution dataset was collected in the absence of light at 100 K after cooling in the dark, in order to determine the “ground-state” structure. The nickel(II) atom adopts the expected square planar coordination geometry and is bound to a bidentate dcpe ligand and two *cis* nitro-(*η^1^-N*O_2_) groups (Figure 2 a).

After collection of the ground-state crystal structure, the crystal was irradiated with 400 nm light, from six LEDs, with 350 mcd intensity and 100 mW power, for a period of 1 h on the diffractometer, after which a second data set was collected using exactly the same data collection parameters as for the ground-state structure. After structure solution and refinement it was found that a 100 % single-crystal to single-crystal transformation of both nitro-(*η^1^-N*O_2_) groups to the nitrito-(*η^1^-O*NO) metastable species had occurred (Figure 2 b). While the geometry at the Ni centre remains unchanged, disorder is now present within the C1–C2 unit of the dcpe ligand and, most significantly, the two newly transformed nitrito ligands adopt an *endo* conformation with respect to the metal centre (see Figure 7 for a depiction of *endo* vs. *exo* nitrito binding in **3**). The O1–N1–O2 nitrito group is fully occupied, whereas the second nitrito group is disordered over two positions. The nitrito groups O3–N2–O4 and O3A–N2A–O4A have occupancies of 60 and 40 %, respectively. In the refinement model the occupancies of the pairs of N2 and N2A, O3 and O3A, O4 and O4A, were constrained to sum to unity, and for each pair the displacement parameters were constrained to be the same. No restraints were placed on the bond parameters for these atoms.

An analysis of intermolecular interactions using Hirshfeld surfaces[13] was undertaken (see the Supporting Information) to establish why the O1–N1–O2 only adopts one conformation in the metastable state but that O3–N2–O4 adopts two. In the ground-state the nitro O1 atom has close contact with two cyclohexyl H atoms with intermolecular distances of 2.257 and 2.432 Å, and the O2 shortest contact is 2.690 Å. The nearest interatomic contacts for O3 and O4 are longer, at 2.818 and 2.825 Å, respectively. Thus, when the nitro N1-O1,O2 is photoactivated, the nitrito conformation adopts the orientation which is not sterically hindered. Because the contacts for the N2–O3,O4 nitro ligand are longer there is more space to allow either conformation of the photoactivated species to be adopted.

Also worthy of note within the molecular structures is a small but significant shortening of the Ni–P bond lengths of about 0.02 Å upon photoactivation. This corresponds to the change from the phosphorus being *trans* to the N-bound nitro to being *trans* to the O-bound nitrito ligands. Ni1–P1 and Ni1–P2 distances change from 2.1841(4) to 2.1638(19) Å and 2.1934(4) to 2.1767(19) Å, respectively.

A significant change is observed when the crystal packing of the ground-state and metastable-state structures is compared. While the overall unit-cell volume changes by only 0.65 %, the toluene solvent molecule has adopted a new orientation and is disordered over two positions in the metastable state structure.

To establish the temperature at which the photoactivated metastable species can rapidly thermally convert back to the ground state, the sample was warmed after irradiation at 100 K and X-ray data sets were collected at 10 K intervals. Complete loss of the metastable nitrito species and recovery of the nitro ground-state species was observed at 180 K, in agreement with the temperature established by the Raman experiment. The process of cooling from above 180 to 100 K in the dark, irradiating with 400 nm light, and warming back to above 180 K, was repeated five times, with structures being collected at 100 K before and after irradiation, and at 180 K, in every cycle. No appreciable decrease in crystal quality was observed, meaning that this is another example of a reversible, 100 % photoisomerisation of a Ni-nitro complex in a single-crystal, this time with a metastable temperature slightly higher than our previously published example [Ni(dppe)Cl(η^*1*^*-N*O_2_)] (160 K).[12]

**Scheme 3:**
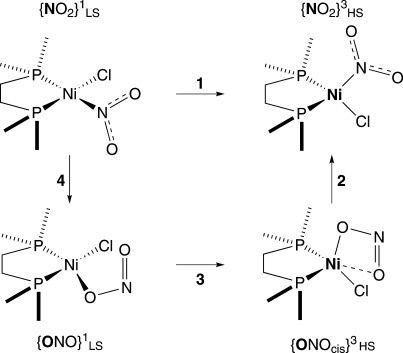
Calculated scheme for conversion from nitro to nitrito complex.

A computational study on the model complex [Ni(dmpe)Cl(NO_2_)] was undertaken, exploring the feasibility of theoretically modelling the isomerisation process, including establishing a plausible mechanism. The general outline is depicted in Scheme 3. In step 1, the starting point is the planar, singlet nitro complex, {NO_2_}^1^_LS_, and computes the energy (at the low-spin geometry) of the lowest triplet state ({NO_2_}^3^_LS_, not shown) before allowing the system to relax to a tetrahedral structure ({NO_2_}^3^_HS_), passing through the triplet-singlet minimum energy crossing point on the way. The nitro group isomerises to nitrito in step 2 ({ONO_*cis*_}^3^_HS_), all on the triplet surface (vide infra), before relaxing to the planar singlet structure ({ONO_*cis*_}^1^_LS_) in step 3. In step 4, the nitrito form reverts back to the original nitro complex.

**Figure 3:**
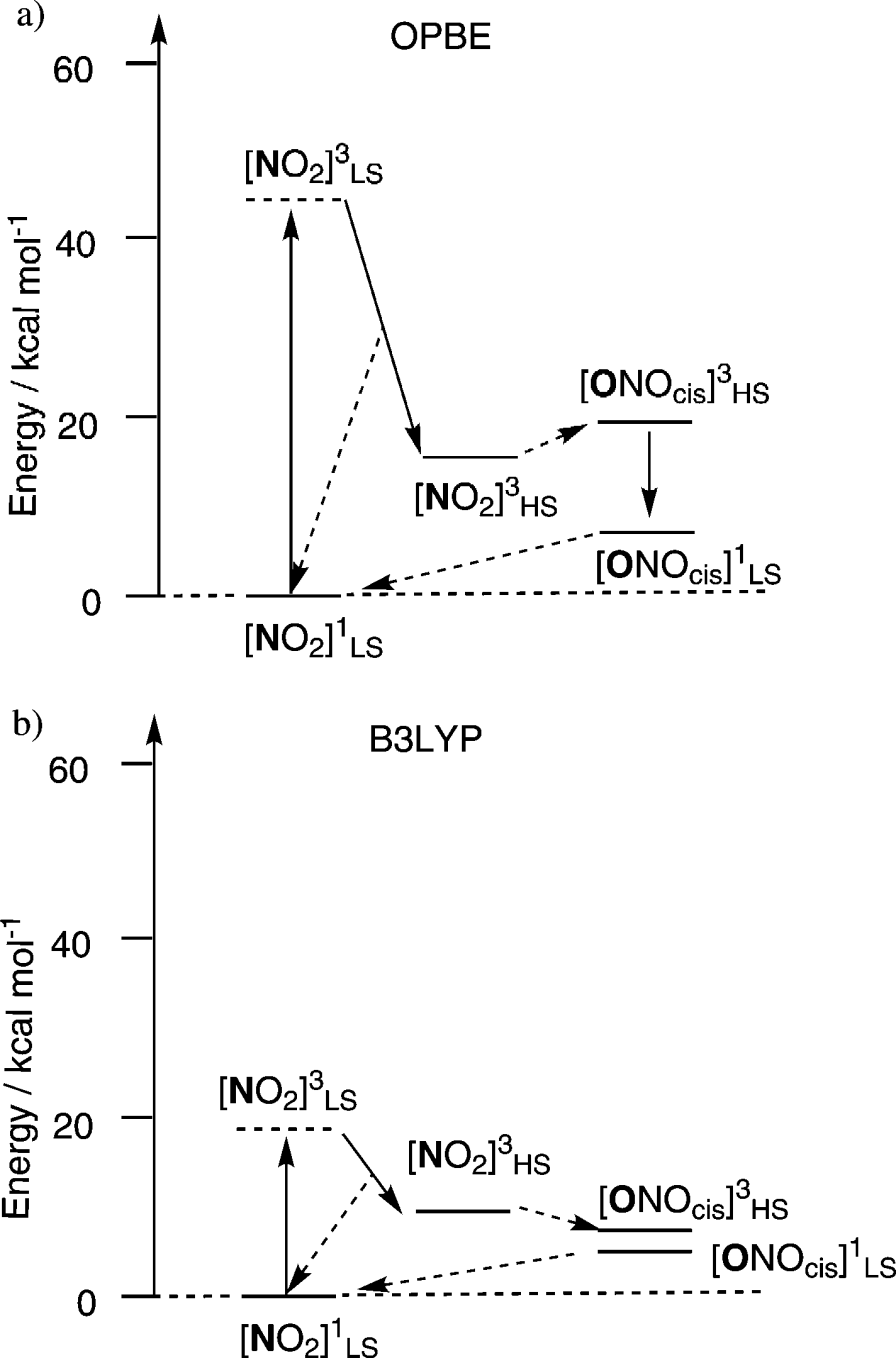
Energy profiles for OPBE and B3LYP. The dotted arrows refer to either a minimum energy crossing point or to a one or two-step intervening process.

**Scheme 4:**
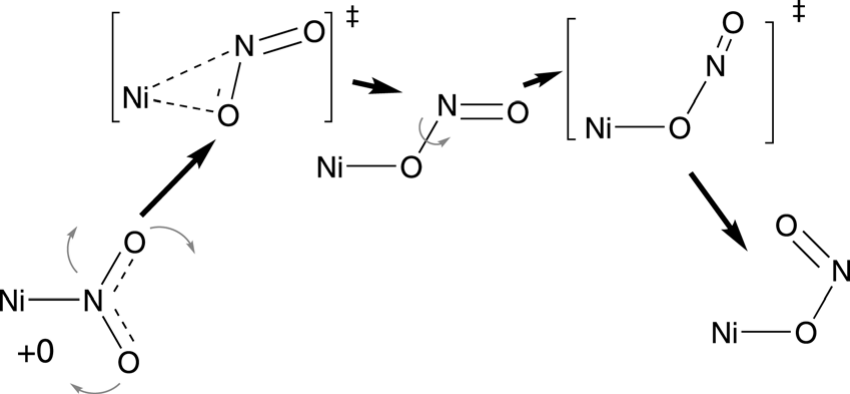
Isomerisation mechanism from B3LYP calculations. The curved arrows indicate the general motions involved. The energetics are described in more detail in the text.

The OPBE pathway does not appear to be consistent with experiment. N-bonded nitro coordination is always lowest in energy on both the singlet and triplet surfaces. For the latter, there is thus no driving force for isomerisation. In contrast, B3LYP predicts the N-bonded nitro configuration to be lower than nitrito on the singlet surface but that O-bonded nitrito is lower in energy on the triplet surface. The computed energies are given in Table 1 and are shown schematically in Figure 3. This qualitative feature does not depend on the inclusion of COSMO effects, dispersion energies[14] or zero-point energies. Reoptimising the structures using B3LYP also does not affect this result. The difference appears to be due to the presence or absence of Hartree–Fock exchange. Thus, common hybrid functionals, like B3LYP, B3LYP*, PBE0 and Becke-half-and-half LYP all suggest nitrito is the lowest energy coordination mode on the triplet surface while “pure” functionals, such as BLYP, PW91, BP86 and LDA all have N-bonded nitro as the lowest coordination mode on the triplet surface. We have observed the curious nature of Ni–NO_2_ coordination before in the context of spin and charge densities for *trans*-[Ni(NH_3_)_4_(NO_2_)_2_].[15] Meanwhile, the isomerisation mechanism on the triplet surface has been analysed in more detail. Nitro to nitrito isomerisation occurs in two steps (Scheme 4). In the first, the nitro group rotates about a vector perpendicular to the ligand plane to generate an *exo*-nitrito species before rotating about the O–N single bond to bring the terminal oxygen back over the metal centre, to yield the *endo*-nitrito form. With respect to the starting nitro species, the barrier heights for both process are very similar (11–15 kcal mol^−1^ depending on the method) with the intermediate sitting in a very shallow minimum with barriers of only 1–2 kcal mol^−1^ on either side.

**Table 1:** Calculated energies for stationary points along the reaction path

Species	*E*(OPBE)	Δ*E*(OPBE)	*E*(B3LYP)	Δ*E*(B3LYP)^[a]^
{NO_2_}^1^_LS_	−3520.56	0.0	−4032.28	0.0
{NO_2_}^3^_LS_	−3487.29	33.3	−4012.91	19.4
{NO_2_}^3^_HS_	−3503.85	16.7	−4022.89	9.4
{ONO_*trans*_}^3^_HS_	−3490.17	30.4	−4012.91	19.4
{ONO_*cis*_}^3^_HS_	−3499.73	20.8	−4026.45	5.8
{ONO}^1^_LS_	−3513.13	7.4	−4028.44	3.8

[a] At OPBE geometry.

**Figure 4:**
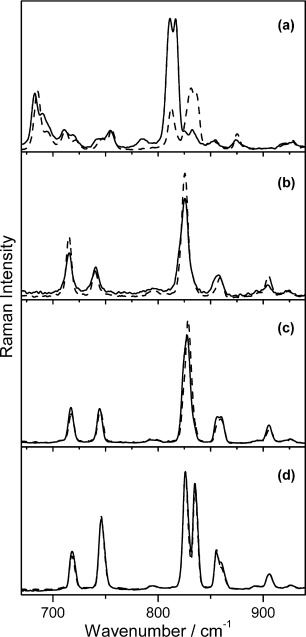
Raman spectra recorded at 100 K before (dashed) and after irradiation (solid) with 400 nm LEDs for the complexes: a) 2, b) 3, c) 4, and d) 5.

The combined Raman and photocrystallographic methodologies for complexes **2**–**5** were repeated. The Raman spectroscopic experiments are summarised in Figure 4 and Table 2. In order to investigate the influence of the bulky periphery of the complex, the diphosphine ligand was changed from dcpe to dppe (cyclohexyl-substituted to phenyl-substituted). For *cis*-[Ni(dppe)(NO_2_)_2_] (**2**), complete conversion from the ground-state nitro-isomer to metastable nitrito-isomer was observed in the Raman experiment after 80 min irradiation at 100 K.

**Table 2:** Conversion of nitro-isomers to nitrito-isomers on irradiation with 400 nm light measured by Raman and crystallographic methods

	Raman experiments			Crystallographic experiments			Raman bands [cm^−1^]	
	irradiation time^[a]^ [min]	conversion [%]^[b]^	metastable *T* [K]	irradiation time^[a]^ [min]	conversion [%]^[b]^	metastable *T* [K]	ground state	metastable state
**1**	1	100	180	60	100	170	813, 817	828
**2**	80	100	170	120	100	170	812, 831 836	811, 816
**3**	10	20	140	11	82	130	826, 829	856
**4**	100	12	220^[c]^	338	44	∼180^[d]^	828	–
**5**	140	0	–	240	27	300	826, 835	–

[a] After which max conversion was reached. [b] Approximate percentage loss of ground state. [c] Temperature uncertain due to poor spectral quality. [d] Partial recovery of ground state (see text).

**Figure 5:**
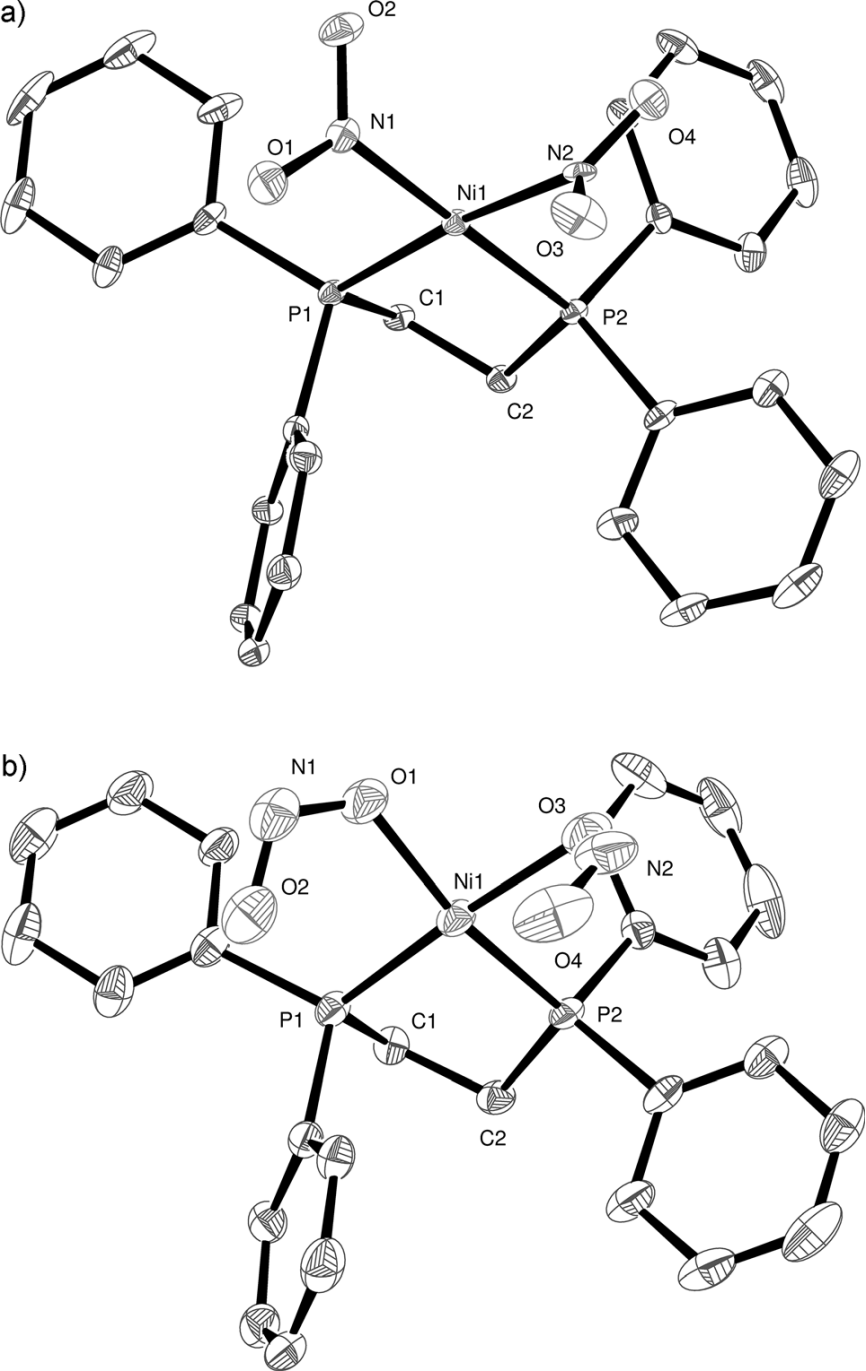
X-ray structure of: a) ground state, and b) metastable state of 2 with thermal ellipsoids set at 30 % probability and hydrogen atoms removed for clarity.

The crystal structure of the complex [Ni(dppe)(*η^1^-N*O_2_)_2_] (**2**) was determined at 100 K after a single crystal was cooled in the dark (Figure 5 a). The complex crystallises in the orthorhombic space group *P*2_1_2_1_2_1_, and the asymmetric unit also contains one water molecule of crystallisation. The square-planar nickel atom is bound to the bidentate dppe ligand and two *cis* nitro-(*η^1^-N*O_2_) groups. The crystal was then irradiated on the diffractometer at 100 K for 2 h using 400 nm LEDs. The resulting crystal structure showed a 100 % double transformation from N-bound nitro to the metastable O-bound nitrito ligands (Figure 5 b).

Overlaying the ground-state and metastable-state structures and comparing the difference in positions of all non-hydrogen atoms excluding the nitro/nitrito groups showed there was no major rearrangement of the structures; the average positional change (RMS) was 0.109 Å. As observed in **1**, there is a notable shortening of the Ni–P distances of about 0.015 Å, as the group *trans* to the P-donor atoms changes from N-bound nitro to O-bound nitrito. The photoactivated transformation is accompanied by an increase of approximately 1 % of the unit-cell volume, this increase being commensurate with less efficient crystal packing of the metastable state (see the analysis of the intermolecular interactions in the Supporting Information). Variable temperature photocrystallographic studies show that the metastable species exist with the same occupancy between temperatures of 100–170 K. The metastable nitrito isomer reverts back to the nitro isomer at or above 180 K.

Comparing **2** with **1** shows that both complexes demonstrate 100 % photoisomerisation at 100 K. The metastable temperature for both complexes is around 170–180 K, but it appears that complex **2**, with the phenyl-substituted phosphines, requires longer photolysis under the same conditions to achieve full conversion.

**Figure 6:**
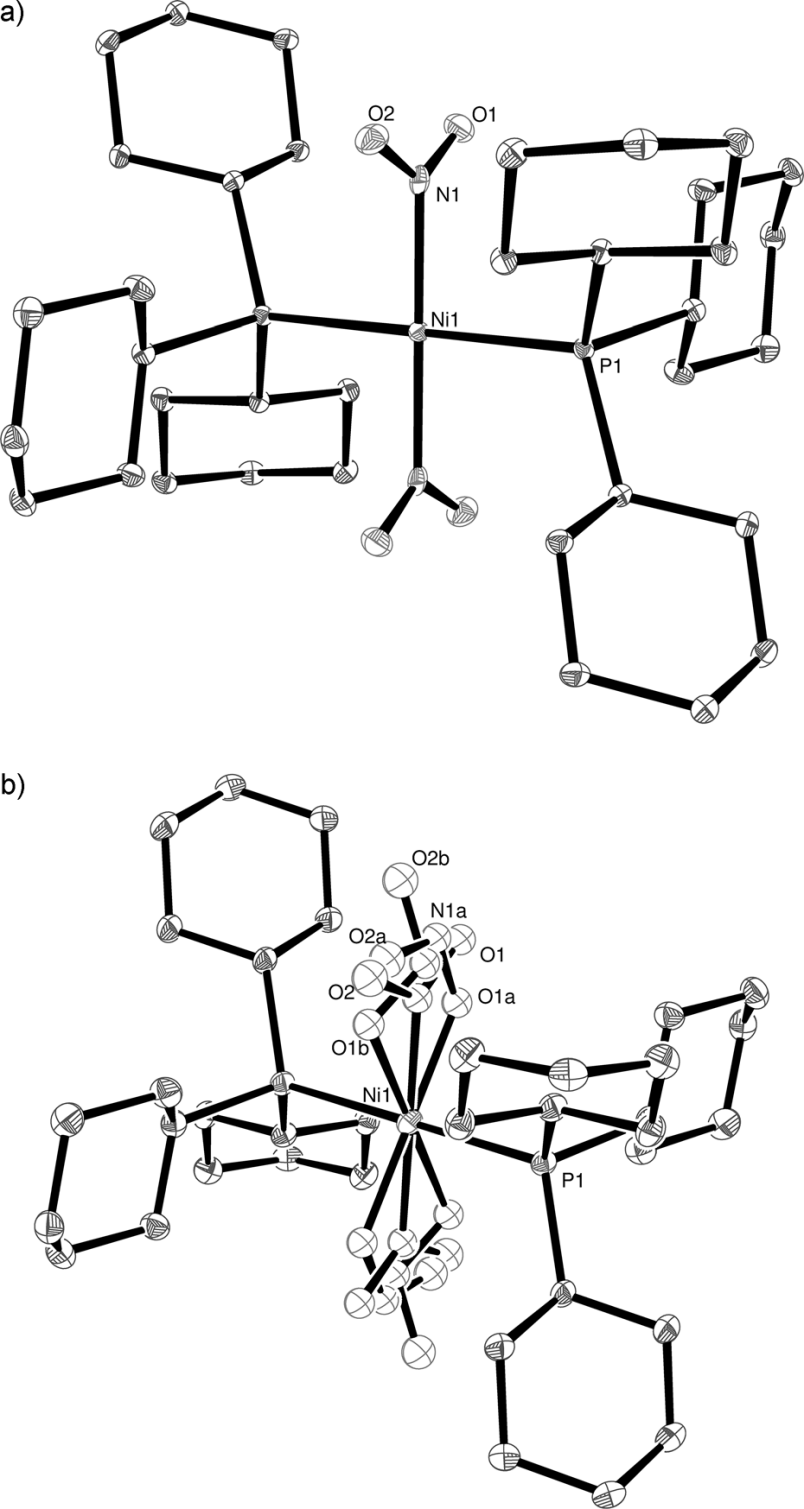
X-ray structure of: a) ground state, and b) metastable state of 3 with thermal ellipsoids set at 30 % probability and hydrogen atoms removed for clarity.

**Figure 7:**
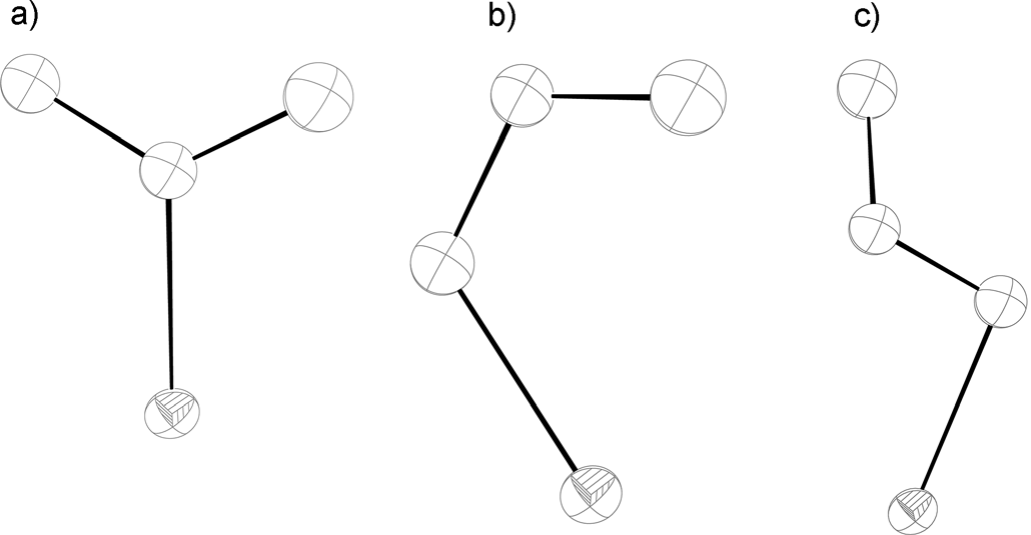
Constituent components of the nitro/nitrito transformation: a) nitro-(*η*^1^-*N*O_2_); b) *endo*-nitrito-(*η*^1^-*O*NO) thermal ellipsoids set at 30 % probability; c) *exo*-nitrito-(*η*^1^-*O*NO).

Since it appears that periphery of the phosphine ligand may impact the photoisomerisation process, studying [Ni(PCy_3_)_2_(*η^1^-N*O_2_)_2_] (**3**) serves two purposes: to investigate the effect of “decoupling” the two phosphine donors to the metal into two monodentate ligands (perhaps changing the overall flexibility of the peripheral ligands), and separately to alter the arrangement of the donor ligands such that a possible *trans* effect can be studied. Furthermore, this complex is the first in the series of three complexes which allow investigation of the effect of changing metal centre on photoisomerisation (see below). The Raman spectroscopic experiments interestingly showed a *lower* conversion to the metastable nitrito-isomer on 400 nm irradiation at 100 K than in either **1** or **2**, reaching a maximum of only 20 % loss of the ground-state band at 825 cm^−1^ over a period of 10 min. As before the structure of **3** was determined in the dark at 100 K. The complex crystallised in the triclinic space group *P*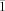. The square-planar nickel atom is positioned on a crystallographic inversion centre and is bound to two *trans*-PCy_3_ ligands and two *trans*-nitro groups (Figure 6 a). The crystal was irradiated (400 nm) at 100 K for 11 min and the resulting crystal structure (Figure 6 b) had three different orientations of the NO_2_ ligands, illustrated separately for clarity in Figure 7. The components consist of the nitro-(*η^1^-N*O_2_) ground-state species, an *endo*-nitrito (*η^1^-O*NO) species and an *exo*-nitrito (*η^1^-O*NO) species; the crystallographically determined occupancies of the three species are 18, 62 and 20 %, respectively (the occupancies of the related atoms were summed to unity with SUMP, and N–O and O–*N*–O parameters were restrained with DFIX and DANG). Unlike **1** and **2**, crystal decomposition (characterised by the loss of coherent Bragg peaks and high angle data) was observed during irradiation and the highest level of conversion achieved was 82 %. Overlaying the crystal structures of the ground-state and metastable states of **3** showed there was very little change in structural conformation other than the nitro/nitrito group, the mean squares positional change of non-hydrogen atoms being 0.030 Å and the unit-cell volume change only being approximately 1 %. These limited changes suggest a fairly rigid structure around the phosphines, and the presence of both *endo*- and *exo*-nitrito isomers is interesting since the latter orientation was not observed with **1** or **2**. Also noteworthy is the drop in metastable temperature to approximately 130 K. Thermal back-conversion (i.e., nitrito- to nitro-isomer) of related [Co(en)_2_(ONO)_2_] complexes in solution[16] has been shown to be about ten times *faster* for *trans*-[Co(en)_2_(ONO)_2_] than for *cis*-[Co(en)_2_(ONO)_2_], a finding commensurate with the reduced metastable temperature we observe here for complex **3** when compared with complex **1**, and evidence that a significant *trans* effect is operating. It is also possible that the lack of complete photoconversion of **3** may be the result of an equilibrium being set up between the nitro and nitrito isomers during the irradiation.

One would expect the lability of the M–NO_2_ bonding to decrease as M goes from first-row to third-row transition metal and indeed, the conversion from nitro to nitrito isomer in the *trans* series of [M(PCy_3_)_2_(NO_2_)_2_] complexes measured by the Raman experiments is highest for M=Ni (ca. 20 %) and lowest (no conversion identified) for M=Pt when irradiated (400 nm) at 100 K (Table 2).

**Figure 8:**
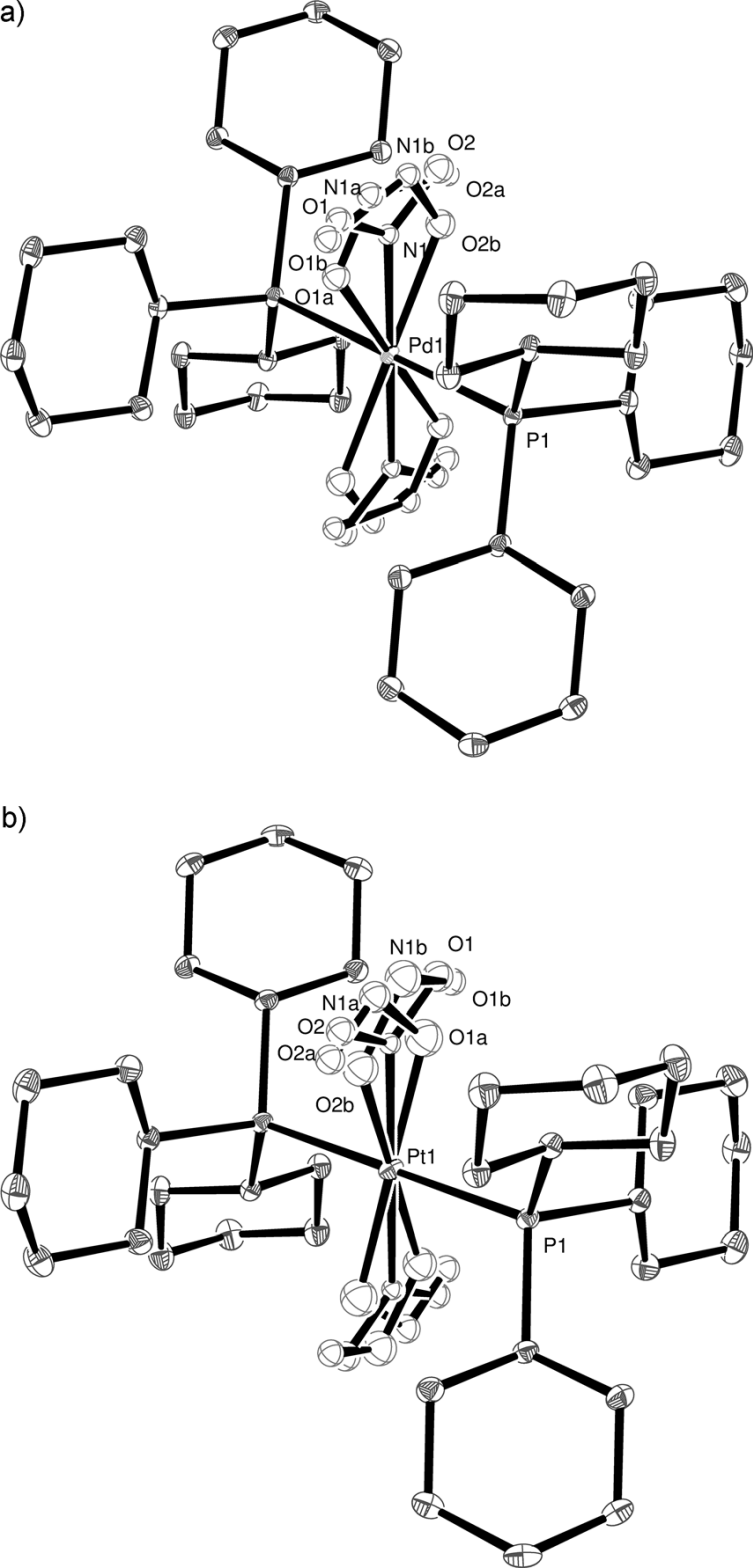
Irradiated X-ray structure of: a) 4, and b) 5 with thermal ellipsoids set at 30 % probability and hydrogen atoms removed for clarity.

The ground-state crystal structures of [Pd(PCy_3_)_2_(NO_2_)_2_] (**4**) and [Pt(PCy_3_)_2_(NO_2_)_2_] (**5**) were obtained at 100 K in the dark and showed the presence of only the nitro isomer. Both complexes crystallise in the triclinic space group *P*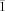 and are isomorphous with the nickel complex **3**; as such, within each structure there is only one unique nitro group, the other being related by crystallographic symmetry. The crystal of **4** (Pd) was irradiated (400 nm) at 100 K for 5.5 h and the resulting crystal structure is shown in Figure 8 a. After irradiation the total level of conversion from nitro-(*η^1^-N*O_2_) to nitrito-(*η^1^-O*NO) was 44 %; the nitrito ligands being disordered over two positions with occupancy 23 and 21 %, both in the *endo* configuration (Figure 8 a). Even with further irradiation higher conversion was not achieved. The metastable species exist with the same occupancy between temperatures of 100–170 K. At 180 K the total occupancy of the metastable state reduces from 44 to 33 % and reduces again to 20 % at 190 K. The 20 % conversion remains in the crystal structure even at room temperature. Similarly, the 100 K ground-state structure of a crystal of **5** (Pt) was collected, the crystal irradiated for 4 h and the structure redetermined. The resulting crystal structure displayed a conversion from nitro-(*η^1^-N*O_2_) to nitrito-(*η^1^-O*NO) of only 27 %; the nitrito ligands being disordered over two positions with occupancy 14 and 13 %, both in the *endo* configuration (Figure 8 b). Further irradiation did not increase the conversion to the nitrito isomer. However, variable temperature studies show that once formed a nitrito isomer component is stable even at room temperature. In the refinement model for these two structures the occupancies of the N and O atoms in the ground state NO_2_ group and the two orientations of the metastable *endo*-ONO group were summed to unity using the SUMP command. These atoms were refined with isotropic displacement parameters, and N–O bond lengths and ONO angles were restrained with DFIX and DANG.

We attribute the contrast in the conversion levels of 44 and 27 % from the crystal structure determinations of the Pd and Pt systems, respectively, to the apparent lack of conversion of these species from the Raman data, which may be attributed to the time taken to run the experiments. The Raman spectra were run after irradiation times of a few minutes whereas 5.5 and 4 h irradiation periods were required to achieve the level of conversion observed from the crystal structure determinations.

The increasing difficulty in converting nitro- to nitrito-isomers in the Pd and Pt complexes, combined with the marked increase in thermal stability of the nitrito isomers once formed is a clear demonstration of the expected decrease in photoreactivity of the complexes going from Ni to Pd to Pt. In addition to this trend an examination of the reaction cavity provides additional evidence that both thermodynamic and kinetic factors play a part in the isomerisation process. A direct comparison can be made for **3**, **4** and **5**, which are isomorphous. The percentage increase in the void space in going from the NO_2_ ground state to the metastable state in the cases of **3**–**5** are shown in Table 3. The change in void space for **3** is negligible, at 0.1 %, therefore inducing limited lattice strain. In the structures of **4** and **5**, in comparison, the change is greater than 1 % suggesting that larger lattice strain was involved in the rearrangement, a factor previously suggested to be a limiting factor in the level of conversion observed.[17] The smaller the change in cavity size upon conversion the smaller is the strain induced.

**Table 3:** Cavity size percentage increase from ground to metastable states in complexes 3–5

	Ground state void space as a [%] of volume	Metastable state void space as a [%] of volume	[%] increase
**3**	1.2	1.3	0.1
**4**	1.3	2.4	1.2
**5**	1.7	2.8	1.1

## Conclusion

We have synthesised and characterised a series of square planar group 10 metal complexes that exhibit switchable metastable-state behaviour. Single-crystal X-ray photocrystallographic and solid-state Raman spectroscopy studies of these materials, containing two nitro groups in both *cis* or *trans* arrangements, supported by bulky phosphine ligands, show that they undergo high levels of interconversion between the nitro and nitrito linkage isomers in single crystals when irradiated with UV LEDs. An initial computational study of the model complex [Ni(dmpe)Cl(NO)_2_] demonstrated that B3LYP appears to provide a viable method for modelling these systems and we will report a fuller analysis of the role of HF exchange in these systems plus extend the calculations to the dinitro complexes of Ni, Pd and Pt in due course. Experimentally, the nickel(II) complexes *cis*-[Ni(dcpe)(NO_2_)_2_] (**1**) and *cis*-[Ni(dppe)(NO_2_)_2_] (**2**) with bidentate phosphine ligands, display 100 % reversible conversion from nitro-(*η^1^-N*O_2_) to nitrito-(*η^1^-O*NO) upon irradiation. Complex **2** requires a longer irradiation time to achieve complete conversion, possibly due to the change in steric crowding around the nitro groups caused by changing the peripheral substituents from cyclohexyl to phenyl rings. The complex [Ni(PCy_3_)_2_(NO_2_)_2_] (**3**) with monodentate *trans*-phosphine ligands partially degrades upon irradiation but still gives an 82 % conversion to the nitrito isomer, along with a reduced metastable temperature of 130 K (compared with ∼180 K for **1** and **2**). This reduction in metastable temperature highlights the effect of the change in geometry from *cis*- to *trans*-nitro groups. Palladium (**4**) and platinum (**5**) analogues of **3** reach a photostationary point upon prolonged irradiation and the metastable species are thermally stable to higher temperatures than the equivalent nickel complex. These species retain crystallinity upon irradiation. The rate of photoreaction reduces going from nickel to palladium to platinum, offering an elegant demonstration of the expected chemical reactivity of these compounds, coupled with the use of “cavity size” arguments, to rationalise the levels of conversion between the linkage isomers observed. These results reinforce our contention that these metastable switchable state materials can be understood, rationalised and further developed using core chemical principles.

## Experimental Section

Raman spectra were recorded on a confocal Horiba Scientific LabRAM HR Raman Microscope using a 660 nm diode laser and a 600 lines per mm grating. The detector was a Synapse CCD detector. The spectra shown were recorded using an approximately 10 mW laser power, 48 acquisitions of 2.5 s/acquisition per spectrum. Temperature control was achieved using a Linkam FTIR600 variable-temperature stage with CaF_2_ windows and modified tubing to fit the Raman spectrometer. Crystal irradiation was performed using a cluster of seven UV LEDs (400 nm) placed 8 mm above the sample.

X-ray diffraction investigations were carried out on Station 11.3.1 of the Advanced Light Source (ALS),[18] Lawrence Berkeley National Laboratory and on Station 9.8 of Daresbury Synchrotron Radiation Source.[19] At both facilities single-crystal X-ray data collections were carried out on a Bruker APEXII CCD diffractometer equipped with an Oxford Cryosystems cryostream cooling device (for temperature studies in the range 90–300 K). Irradiation was performed using a ring of six LEDs affixed to the crystal cooling apparatus within 1 cm of the crystal.[20]

Suitable single crystals were mounted on the diffractometer and cooled to 100 K. Ground-state structures (“dark”) were collected with no external light. The crystals were then irradiated in situ with LEDs (UV: 400 nm, 350 mcd at 3.7 V). During irradiation the crystal was continuously rotated. After initial irradiation a second dataset (“light”) was collected, the level of photoactivation conversion was assessed through structure solution and the structure was refined with the nitro and nitrito components being treated as a disorder model with the total occupancy of each atom being summed to unity. The sample was then irradiated further and the process was repeated until the level of conversion to the nitrito complex reached a maximum. The crystal structure was then recorded at 10 K intervals, warming from 100 K to assess the temperature range over which the metastable state was present.

The program APEX[21] was used for collecting frames of data, indexing reflections, and determination of lattice parameters, SAINT[21] for integration of the intensity of reflections and scaling, and SADABS[22] for absorption correction. The structures were solved by direct methods using SHELXS-86[23] and refined by full-matrix least-squares on *F*^2^ using SHELXL97.[24] CCDC-015636 http://www.ccdc.cam.ac.uk/cgi-bin/catreq.cgi(**2**, ground state), CCDC-915637 http://www.ccdc.cam.ac.uk/cgi-bin/catreq.cgi(**2**, metastable state), CCDC-915638 http://www.ccdc.cam.ac.uk/cgi-bin/catreq.cgi(**1**, ground state), CCDC-915639 http://www.ccdc.cam.ac.uk/cgi-bin/catreq.cgi(**1**, metastable state), CCDC-915640 http://www.ccdc.cam.ac.uk/cgi-bin/catreq.cgi(**3**, ground state), CCDC-915641 http://www.ccdc.cam.ac.uk/cgi-bin/catreq.cgi(**3**, metastable state), CCDC-915642 http://www.ccdc.cam.ac.uk/cgi-bin/catreq.cgi(**4**, ground state), CCDC-915643 http://www.ccdc.cam.ac.uk/cgi-bin/catreq.cgi(**4**, metastable state), CCDC-915644 http://www.ccdc.cam.ac.uk/cgi-bin/catreq.cgi(**5**, ground state), and CCDC-915645 http://www.ccdc.cam.ac.uk/cgi-bin/catreq.cgi(**5**, metastable state) contain the supplementary crystallographic data for this paper. These data can be obtained free of charge from The Cambridge Crystallographic Data Centre via http://www.ccdc.cam.ac.uk/data_request/cif.

The complexes **1**, **2** and **4** were synthesised according to literature procedures.[25] The syntheses of **3** and **5** are described in the Supporting Information. X-ray quality crystals were grown by slow solvent evaporation from CH_2_Cl_2_/toluene for **1**; acetone/water (30:1) for **2**; CH_2_Cl_2_/THF for **3** and CH_2_Cl_2_/acetone for both **4** and **5**.

All Kohn–Sham DFT calculations used the Amsterdam density functional (ADF) program, version 2009,[26] using restricted and unrestricted formalisms for spin singlet and spin triplet states, respectively. Geometries were optimised using the OPBE function in conjunction with an uncontracted STO basis set of triple-ζ plus polarisation (TZP) quality with default SCF and geometrical convergence criteria apart from the Cartesian displacement and gradient threshold, which were both set to 0.002. The integration level was fixed at 6 throughout. Condensed phase environmental effects were incorporated using the conductor-like screening model (COSMO) as implemented in ADF,[27] with a dielectric constant of *ε*=78, and probe radius of 1.9 Å. Stationary points were confirmed by frequency calculations using analytical second derivatives for gas-phase calculations and two-point finite differences of analytical first derivatives for COSMO calculations. B3LYP energies were computed at the OPBE-optimised geometries.

## Supporting Information

As a service to our authors and readers, this journal provides supporting information supplied by the authors. Such materials are peer reviewed and may be re-organized for online delivery, but are not copy-edited or typeset. Technical support issues arising from supporting information (other than missing files) should be addressed to the authors.

miscellaneous_information

## References

[1a] Coppens P, Vorontsov II, Graber T, Gembicky M, Kovalevsky AY (2005). Acta Crystallogr. Sect. A.

[1b] Cole J (2008). Acta Crystallogr. Sect. A.

[1c] Cole JM (2008). Z. Kristallogr. Cryst. Mater.

[1d] Coppens P, Zheng S-L, Gembicky M (2008). Z. Kristallogr. Cryst. Mater.

[1e] Raithby PR (2007). Crystallogr. Rev.

[2] Davaasambuu J, Durand P, Techert S (2004). J. Synchrotron Radiat.

[3a] Šrajer V, Teng T-y, Ursby T, Pradervand C, Ren Z, Adachi S-i, Schildkamp W, Bourgeois D, Wulff M, Moffat K (1996). Science.

[3b] Schotte F, Lim M, Jackson TA, Smirnov AV, Soman J, Olson JS, Phillips GN, Wulff M, Anfinrud PA (2003). Science.

[4a] Coppens P, Vorontsov II, Graber T, Kovalevsky AY, Chen Y-S, Wu G, Gembicky M, Novozhilova IV (2004). J. Am. Chem. Soc.

[4b] Coppens P, Gerlits O, Vorontsov II, Kovalevsky AY, Chen Y-S, Graber T, Gembicky M, Novozhilova IV (2004). Chem. Commun.

[4c] Kim CD, Pillet S, Wu G, Fullagar WK, Coppens P (2002). Acta Crystallogr. Sect. A.

[4d] Novozhilova IV, Volkov AV, Coppens P (2003). J. Am. Chem. Soc.

[5a] Coppens P, Fomitchev DV, Carducci MD, Culp K (1998). J. Chem. Soc. Dalton Trans.

[5b] Coppens P, Novozhilova I, Kovalevsky A (2002). Chem. Rev.

[6] Rüdlinger M, Schefer J, Vogt T, Woike T, Haussühl S, Zöllner H (1992). Physica B.

[7a] Pressprich MR, White MA, Vekhter Y, Coppens P (1994). J. Am. Chem. Soc.

[7b] Carducci MD, Pressprich MR, Coppens P (1997). J. Am. Chem. Soc.

[7c] Fomitchev DV, Coppens P, Li T, Bagley KA, Chen L, Richter-Addo GB (1999). Chem. Commun.

[7d] Cheng L, Novozhilova I, Kim C, Kovalevsky A, Bagley KA, Coppens P, Richter-Addo GB (2000). J. Am. Chem. Soc.

[7e] Fomitchev DV, Bagley KA, Coppens P (2000). J. Am. Chem. Soc.

[7f] Fomitchev DV, Novozhilova I, Coppens P (2000). Tetrahedron.

[7g] Kim C, Novozhilova I, Goodman MS, Bagley KA, Coppens P (2000). Inorg. Chem.

[7h] Kovalevsky AY, Bagley KA, Cole JM, Coppens P (2003). Inorg. Chem.

[7i] Lee J, Kovalevsky AY, Novozhilova IV, Bagley KA, Coppens P, Richter-Addo GB (2004). J. Am. Chem. Soc.

[7j] Kovalevsky AY, King G, Bagley KA, Coppens P (2005). Chem. Eur. J.

[7k] Zangl A, Klüfers P, Schaniel D, Woike T (2009). Inorg. Chem. Commun.

[7l] Schefer J, Schaniel D, Petříček V, Woike T (2008). Z. Kristallogr. Cryst. Mater.

[7m] Schaniel D, Woike T, Schefer J, Petříček V, Krämer KW, Güdel HU (2006). Phys. Rev. B.

[7n] Cormary B, Malfant I, Valade L, Buron-Le Cointe M, Buron-Le Cointe M, Toupet L, Todorova T, Delley B, Schaniel D, Mockus N, Woike T, Fejfarová K, Petrícek V, Dusek M (2009). Acta Crystallogr. Sect. B.

[8] Bowes KF, Cole JM, Husheer SLG, Raithby PR, Savarese TL, Sparkes HA, Teat SJ, Warren JE (2006). Chem. Commun.

[9a] Schaniel D, Mockus N, Woike T, Klein A, Sheptyakov D, Todorova T, Delley B (2010). Phys. Chem. Chem. Phys.

[9b] Schaniel D, Imlau M, Weisemoeller T, Woike T, Krämer KW, Güdel HU (2007). Adv. Mater.

[9c] Imlau M, Woike T, Schieder R, Rupp RA (1999). Phys. Rev. Lett.

[9d] Woike T, Imlau M, Haussühl S, Rupp RA, Schieder R (1998). Phys. Rev. B.

[9e] Woike T, Haussühl S, Sugg B, Rupp RA, Beckers J, Imlau M, Schieder R (1996). Appl. Phys. B.

[10a] Coppens P, Ma B, Gerlits O, Zhang Y, Kulshrestha P (2002). CrystEngComm.

[10b] Cole JM (2008). Models, Mysteries, and Magic of Molecules.

[11] Blake AJ, Champness NR, Easun TL, Allan DR, Nowell H, George MW, Jia J, Sun XZ (2010). Nat. Chem.

[12] Warren MR, Brayshaw SK, Johnson AL, Schiffers S, Raithby PR, Easun TL, George MW, Warren JE, Teat SJ (2009). Angew. Chem.

[b01] (2009). Angew. Chem. Int. Ed.

[14] Grimme S (2004). J. Comput. Chem.

[15] Chandler GS, Deeth RJ, Figgis BN, Phillips RA (1990). J. Chem. Soc. Dalton Trans.

[16a] Miyoshi K, Katoda N, Yoneda H (1983). Inorg. Chem.

[16b] Grenthe I, Nordin E (1979). Inorg. Chem.

[17] Ohashi Y, Yanagi K, Kurihara T, Sasada Y, Ohgo Y (1981). J. Am. Chem. Soc.

[18] http://www.als.lbl.gov/als/techspecs/bl11.3.1.html.

[19] http://www.srs.ac.uk/srs/stations/station9.8.htm.

[20] Brayshaw SK, Knight JW, Raithby PR, Savarese TL, Schiffers S, Teat SJ, Warren JE, Warren MR (2010). J. Appl. Crystallogr.

[23] Sheldrick G (1990). Acta Crystallogr. Sect. A.

[25a] Angulo IM, Bouwman E, Gorkum Rv, Lok SM, Lutz MH, Spek AL (2003). J. Mol. Catal. A.

[25b] Kriege-Simondsen J, Feltham RD (1983). Inorg. Chim. Acta.

[25c] Doughty DT, Gordon G, Stewart RP (1979). J. Am. Chem. Soc.

[25d] Feltham RD, Elbaze G, Ortega R, Eck C, Dubrawski J (1985). Inorg. Chem.

[27] te Velde G, Bickelhaupt FM, Baerends EJ, Fonseca Guerra C, van Gisbergen SJA, Snijders JG, Ziegler T (2001). J. Comput. Chem.

