# Supplementary material for: Solid-State Interconversions: Unique 100 % Reversible Transformations between the Ground and Metastable States in Single-Crystals of a Series of Nickel(II) Nitro Complexes
Source: Chemistry. 2014 Mar 18;20(18):5468–77. doi: 10.1002/chem.201302053 (PMC4164279; doi:10.1002/chem.201302053)
Supplement: Supplementary file 1 — miscellaneous_information [file chem0020-5468-sd1.pdf]

CHEMISTRY
A European Journal

Supporting Information

© Copyright Wiley-VCH Verlag GmbH & Co. KGaA, 69451 Weinheim, 2014

Solid-State Interconversions: Unique 100 % Reversible Transformations between the Ground and Metastable States in Single-Crystals of a Series of Nickel(II) Nitro Complexes

Mark R. Warren,[a, f] Timothy L. Easun,[b] Simon K. Brayshaw,[a] Robert J. Deeth,*[c] Michael W. George,*[b] Andrew L. Johnson,[a] Stefanie Schiffers,[a] Simon J. Teat,[d] Anna J. Warren,[a, f] John E. Warren,[e] Chick C. Wilson,[a, f] Christopher H. Woodall,[a, f] and Paul R. Raithby*[a, f]

chem_201302053_sm_miscellaneous_information.pdf

SUPPORTING INFORMATION

Solid State Interconversions: Unique 100% Reversible Transformations between the Ground and Metastable States in Single-crystals of a Series of Nickel(II) Nitro Complexes

Mark R. Warren, Timothy L. Easun, Simon K. Brayshaw, Robert J. Deeth, Michael W. George, Andrew L. Johnson, Stefanie Schiffers, Simon J. Teat, Anna J. Warren, John E. Warren, Chick C. Wilson, Christopher H. Woodall, Paul R. Raithby

Crystallography
Table S1 Structural data of the compounds **1** and **2** before and after irradiation
Table S2 Structural data of the compounds **3**, **4** and **5** before and after irradiation

Diagrams
Structure of **1** showing the disordered toluene.
Structure of **1** showing the intermolecular contacts
Hirshfeld surface for **1**
Structure of **2** showing the intermolecular contacts
Compound**s 1-5:** Slant Plane Fourier maps of the ground state vs. the metastable state.

Computational Studies
Energies and Cartesian Coordinates

Table S1 Structural data of the compounds **1** and **2** before and after irradiation

Compound reference	**1**	**1** irradiated	**2**	**2** irradiated
Chemical formula	$C_{26}H_{48}N_2NiO_4P_2•C_7H_8$	$C_{26}H_{48}N_2NiO_4P_2•C_7H_8$	$C_{26}H_{24}N_2NiO_4P_2•H_2O$	$C_{26}H_{24}N_2NiO_4P_2•H_2O$
Formula Mass	665.45	665.45	565.12	565.12
Crystal system	Monoclinic	Monoclinic	Orthorhombic	Orthorhombic
a/Å	12.2125(5)	12.199(10)	8.1137(2)	7.9997(5)
b/Å	18.7052(8)	19.146(8)	13.7208(4)	13.9307(8)
c/Å	15.1718(6)	15.093(10)	22.6701(7)	22.8956(14)
α/°	90.00	90.00	90.00	90.00
β/°	97.2610(10)	101.00(5)	90.00	90.00
γ/°	90.00	90.00	90.00	90.00
Unit cell volume/Å^3	3438.0(2)	3461(4)	2523.78(12)	2551.5(3)
Temperature/K	100(2)	100(2)	100(2)	100(2)
Space group	*P2(1)/c*	*P2(1)/c*	$P2_12_12_1$	$P2_12_12_1$
No. of formula units per unit cell, Z	4	4	4	4
Radiation type	Synchrotron	Synchrotron	Synchrotron	Synchrotron
Wavelength/Å	0.6896	0.6896	0.6896	0.6896
No. of reflections measured	37994	38030	28401	27756
No. of independent reflections	10185	10272	7512	7527
R_{int}	0.0474	0.0437	0.0517	0.0463
Final R_1 values ($I > 2\sigma(I)$)	0.0394	0.0946	0.0427	0.0742
Final $wR(F^2)$ values ($I > 2\sigma(I)$)	0.0933	0.2501	0.1106	0.1959
Final R_1 values (all data)	0.0497	0.1177	0.0472	0.1076
Final $wR(F^2)$ values (all data)	0.0992	0.2704	0.1141	0.2198

Table S2 Structural data of the compounds **3**, **4** and **5** before and after irradiation

Compound reference	**3**	**3** irradiated	**4**	**4** irradiated	**5**	**5** irradiated
Chemical formula	$C_{36}H_{66}N_2NiO_4P_2$	$C_{36}H_{66}N_2NiO_4P_2$	$C_{36}H_{66}N_2O_4P_2Pd$	$C_{36}H_{66}N_2O_4P_2Pd$	$C_{36}H_{66}N_2O_4P_2Pt$	$C_{36}H_{66}N_2O_4P_2Pt$
Formula Mass	711.56	711.56	759.25	759.25	847.94	847.94
Crystal system	Triclinic	Triclinic	Triclinic	Triclinic	Triclinic	Triclinic
a/Å	10.084(2)	10.005(4)	10.103(5)	10.0521(7)	10.122(5)	10.107(5)
b/Å	10.369(2)	10.344(4)	10.412(5)	10.4185(7)	10.388(5)	10.428(5)
c/Å	10.464(2)	10.713(4)	10.582(5)	10.6814(7)	10.617(5)	10.676(5)
α/°	111.269(3)	112.515(6)	111.425(5)	112.0220(10)	111.736(5)	112.184(5)
β/°	110.575(3)	110.465(6)	110.355(5)	110.3450(10)	110.638(5)	110.509(5)
γ/°	94.503(3)	93.518(6)	94.508(5)	94.0300(10)	94.229(5)	93.825(5)
Unit cell volume/Å^3	927.5(3)	934.9(7)	944.2(8)	946.07(11)	943.3(8)	949.8(8)
Temperature/K	100(2)	100(2)	100(2)	100(2)	100(2)	100(2)
Space group	$P\bar{1}$	$P\bar{1}$	$P\bar{1}$	$P\bar{1}$	$P\bar{1}$	$P\bar{1}$
No. of formula units per unit cell, Z	1	1	1	1	1	1
Radiation type	Synchrotron	Synchrotron	Synchrotron	Synchrotron	Synchrotron	Synchrotron
Wavelength/Å	0.6896	0.6896	0.7749	0.7749	0.7749	0.7749
No. of reflections measured	13115	13698	7454	14110	18288	23431
No. of independent reflections	5575	5619	5002	5889	6334	9014
R_{int}	0.0926	0.0531	0.0545	0.0587	0.0287	0.0430
Final R_1 values ($I > 2\sigma(I)$)	0.0778	0.0596	0.0559	0.0399	0.0185	0.0340
Final $wR(F^2)$ values ($I > 2\sigma(I)$)	0.2008	0.1524	0.1620	0.1033	0.0352	0.0559
Final R_1 values (all data)	0.0888	0.0988	0.0591	0.0425	0.0185	0.0402
Final $wR(F^2)$ values (all data)	0.2118	0.1726	0.1655	0.1054	0.0352	0.0572

LED information:
Single crystal x-ray diffraction experiments:
LED: Six LEDs 1cm from crystal, 400 nm wavelength, 350 mcd intensity, 100 mW power and 30º viewing angle.
S. K. Brayshaw, J. W. Knight, P. R. Raithby, T. L. Savarese, S. Schiffers, S. J. Teat, J. E. Warren, M. R. Warren, J. Appl. Crystallogr. 2010, 43, 337-340.
Raman Spectroscopy:
LED: Six LEDs 1cm from crystal, 400 nm wavelength, 350 mcd intensity, 100 mW power and 30º viewing angle.
Crystal irradiation was performed using a cluster of seven UV LEDs (400 nm) placed 8 mm above the sample.

Compound 1: X-ray crystal structure of excited state compound 1 with toluene solvent

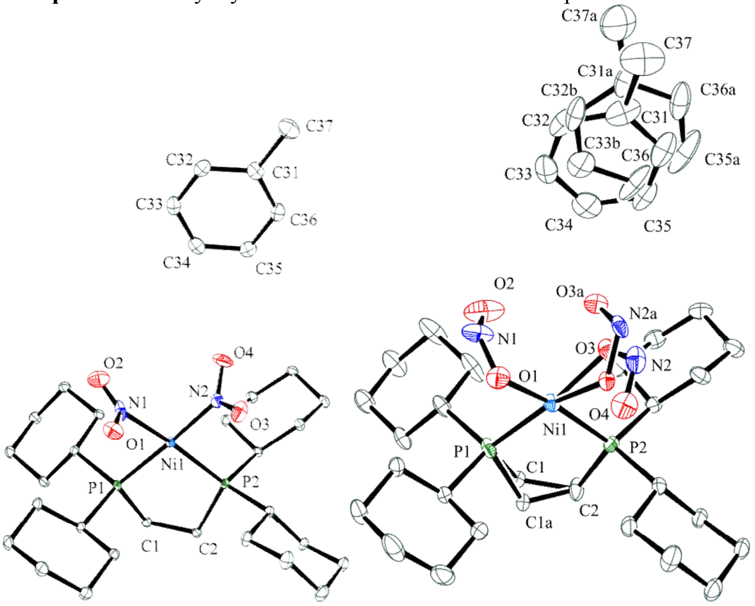

Compound 1: Hirshfeld surfaces (red is close contact) interactions

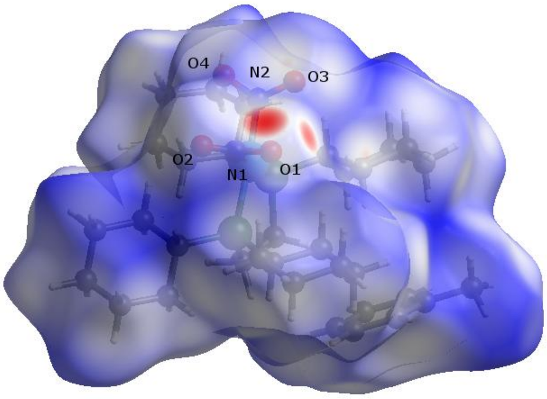

Compound 1: Close contacts for the nitrite ligand in the ground and metastable states structures

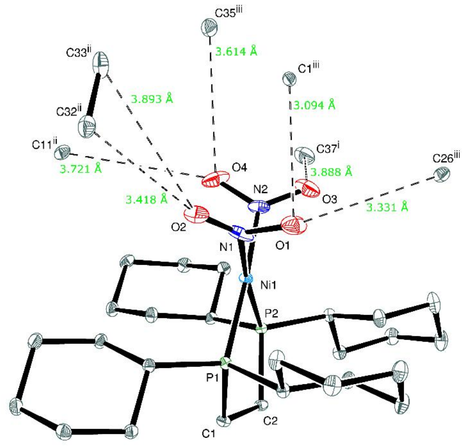

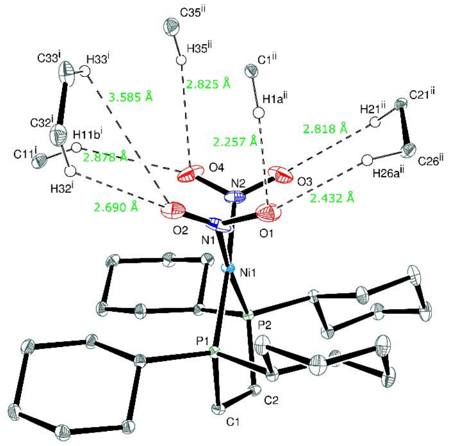

Compounds 1-5: Slant Plane Fourier maps of the ground state vs. the metastable state. The dotted lines are the indication of a decrease and solid lines indicate an increase in electron density. The zero contours are omitted for clarity.

Compound 1:

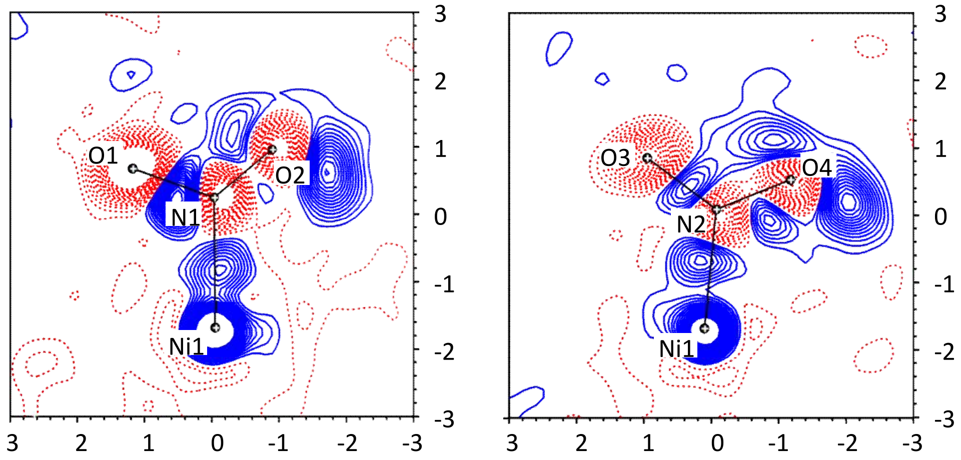

Compound 2

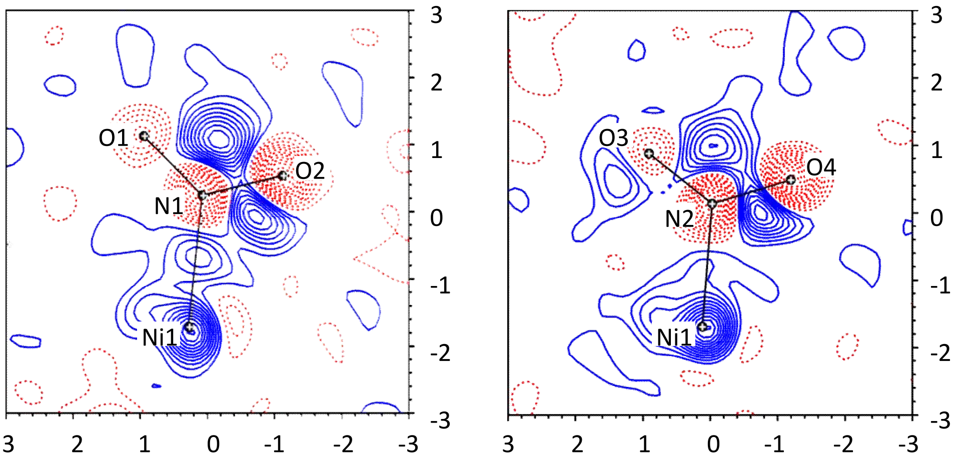

Compound 3:

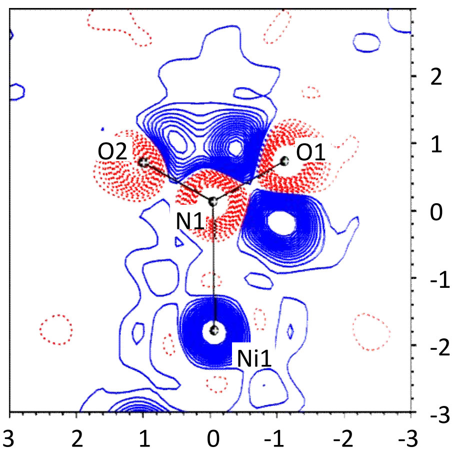

Compound 4:

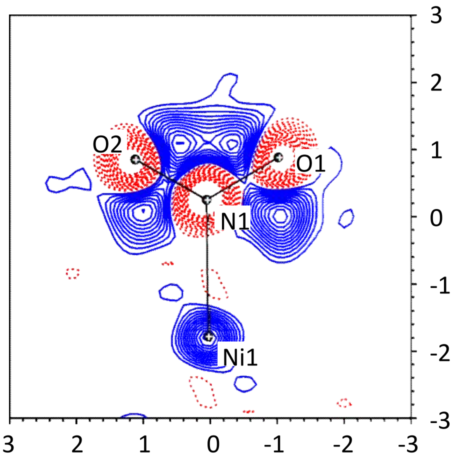

Compound 5:

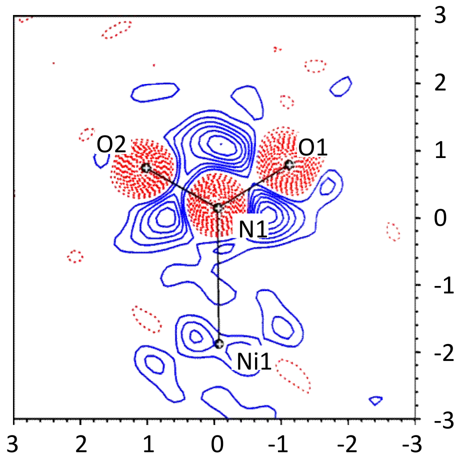
O2
O1
N1
Ni1
3
2
1
0
-1
-2
-3
3
2
1
0
-1
-2
-3

Computational Studies
Energies and Cartesian coordinates.

$\{NO_2\}^1_{LS}$: NO2.sr1

	kcal mol^{-1}	kJ mol^{-1}
Electrostatic Energy:	-2584.66	-10814.22
Kinetic Energy:	3071.33	12850.43
Coulomb (Steric+OrbInt) Energy:	-837.39	-3503.64
XC Energy:	-3134.17	-13113.37
Solvation:	-35.67	-149.23
	----------	-----------
Total Bonding Energy:	-3520.56	-14730.02

E(B3LYP): -4032.28

Ni	8.088600000	9.106900000	2.698200000
Cl	7.319400000	8.839200000	0.616200000
P	8.978900000	9.316700000	4.647100000
P	9.510700000	7.456100000	2.372700000
O	5.659300000	10.131500000	3.424800000
O	7.045500000	11.667900000	2.805100000
N	6.791600000	10.472900000	3.039200000
C	9.987400000	7.827600000	5.043800000
H	10.682600000	8.057100000	5.861500000
H	9.290900000	7.060400000	5.407700000
C	10.699600000	7.368700000	3.782300000
H	11.104800000	6.353100000	3.876000000
H	11.538800000	8.033600000	3.537100000
C	10.131900000	10.727400000	4.725000000
H	10.900700000	10.626600000	3.951200000
H	10.607500000	10.756000000	5.712900000
H	9.582000000	11.658300000	4.555700000
C	7.873300000	9.516600000	6.078900000
H	7.146500000	8.697500000	6.091700000
H	7.339700000	10.469500000	6.017300000
H	8.468600000	9.491100000	6.999400000
C	10.580600000	7.459900000	0.896400000
H	11.154400000	8.391800000	0.854500000
H	9.974500000	7.366100000	-0.009000000
H	11.273500000	6.611700000	0.963400000
C	8.690500000	5.826500000	2.329000000
H	8.088700000	5.678500000	3.232100000
H	9.453200000	5.040600000	2.265400000
H	8.037800000	5.776600000	1.451500000

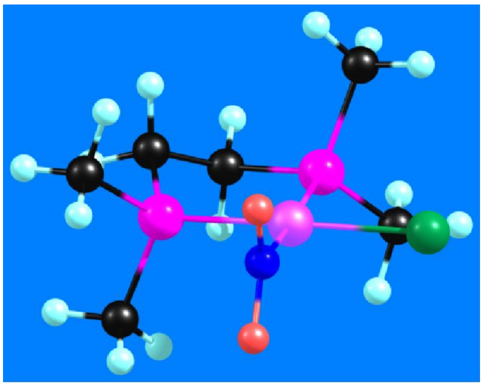

$\{NO_2\}^3_{LS}$:

VERTICAL EXCITATION

-3487.29 kcal mol^{-1}

E(B3LYP): -4012.91

$\{NO_2\}^3_{HS}$: **NO2.sr3:**

Electrostatic Energy:	-2521.10	-10548.26
Kinetic Energy:	3071.44	12850.91
Coulomb (Steric+OrbInt) Energy:	-907.22	-3795.80
XC Energy:	-3115.93	-13037.07
Solvation:	-31.04	-129.89
	----------	-----------
Total Bonding Energy:	-3503.85	-14660.11

E(B3LYP): -4022.89

Ni	0.428415000	0.297902000	0.126787000
Cl	2.547754000	-0.471498000	0.294744000
P	-0.530205000	2.355525000	0.041387000
P	-0.515383000	-0.145749000	-1.896045000
C	-1.701669000	1.229070000	-2.213193000
H	-1.930324000	1.288758000	-3.285427000
H	-2.632407000	0.961533000	-1.695232000

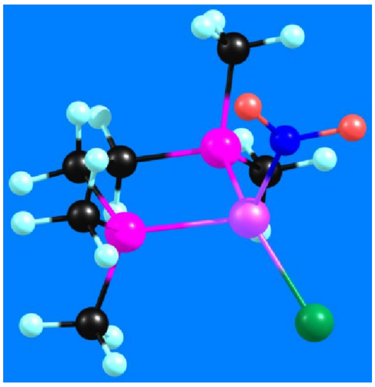

C	-1.144674000	2.549632000	-1.686883000
H	-1.897145000	3.348017000	-1.724643000
H	-0.288266000	2.881658000	-2.288834000
C	0.692006000	-0.087342000	-3.265835000
H	1.276356000	0.837432000	-3.217555000
H	0.174030000	-0.144445000	-4.230395000
H	1.377167000	-0.936288000	-3.167496000
C	-1.457695000	-1.676764000	-2.200764000
H	-2.269426000	-1.759559000	-1.472455000
H	-0.790511000	-2.538513000	-2.089034000
H	-1.870011000	-1.667043000	-3.216808000
C	0.548216000	3.796293000	0.343272000
H	1.437350000	3.732905000	-0.292774000
H	0.862424000	3.790233000	1.392939000
H	0.009254000	4.727380000	0.131276000
C	-1.977078000	2.657866000	1.112798000
H	-2.747707000	1.902134000	0.930970000
H	-1.668053000	2.601213000	2.161849000
H	-2.386662000	3.653869000	0.908627000
O	-1.694529000	-1.205335000	1.318340000
N	-0.683405000	-0.520282000	1.502437000
O	-0.253625000	-0.306055000	2.650840000

$\{\mathbf{ONO}_{trans}\}^3_{HS}$: **ONO_sr5:**

Electrostatic Energy:	-2468.01	-10326.16
Kinetic Energy:	3092.65	12939.64
Coulomb (Steric+OrbInt) Energy:	-972.27	-4067.97
XC Energy:	-3106.92	-12999.36
Solvation:	-35.62	-149.01
	----------	-----------
Total Bonding Energy:	-3490.17	-14602.87

E(B3LYP): -4012.91

Ni	0.020628000	0.085852000	0.045777000
Cl	2.282592000	0.065014000	0.071163000
P	-0.524778000	2.284609000	-0.098671000
P	-0.473262000	-0.095187000	-2.163041000
C	-1.349688000	1.463526000	-2.616142000
H	-1.329256000	1.599768000	-3.705047000
H	-2.400089000	1.332161000	-2.324230000
C	-0.727717000	2.654020000	-1.892164000
H	-1.329484000	3.563618000	-2.018041000
H	0.275791000	2.870012000	-2.281802000
C	0.914935000	-0.237336000	-3.337988000
H	1.603878000	0.602754000	-3.207855000
H	0.529077000	-0.244664000	-4.364210000
H	1.457146000	-1.168100000	-3.142339000
C	-1.609431000	-1.441661000	-2.640311000
H	-2.502429000	-1.411342000	-2.007032000
H	-1.102946000	-2.401768000	-2.492630000
H	-1.902237000	-1.342204000	-3.692228000
C	0.616051000	3.558794000	0.530123000
H	1.597563000	3.432864000	0.062892000
H	0.720950000	3.451898000	1.614874000
H	0.223208000	4.556417000	0.299320000
C	-2.129153000	2.669453000	0.683818000
H	-2.902197000	1.987202000	0.315032000
H	-2.034796000	2.536881000	1.767184000
H	-2.420000000	3.703934000	0.466115000
O	-0.599198000	-0.773956000	1.735804000
N	-0.041680000	-0.618275000	2.902699000
O	-0.628376000	-1.190265000	3.823832000

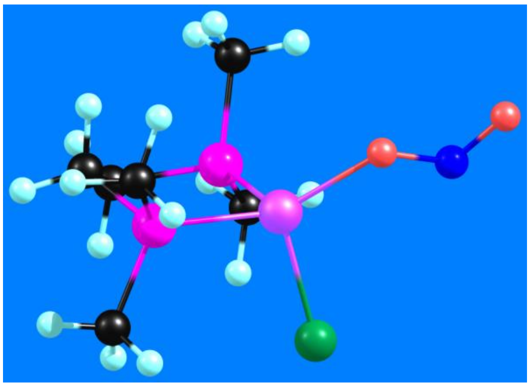

ono_ts1a: TS for cis-trans ONO rotation

Electrostatic Energy:	-2475.05	-10355.60
Kinetic Energy:	3107.58	13002.11
Coulomb (Steric+OrbInt) Energy:	-979.95	-4100.12
XC Energy:	-3108.53	-13006.11
Solvation:	-33.09	-138.47
	----------	-----------
Total Bonding Energy:	-3489.05	-14598.19

$[\{\mathbf{ONO}_{cis/trans}\}^3_{HS}]^\ddagger$

Ni	-0.099572000	0.052326000	-0.177907000
Cl	2.158256000	-0.102909000	-0.074839000
P	-0.498820000	2.275259000	-0.064374000
P	-0.573518000	0.118680000	-2.387232000
C	-1.070460000	1.861107000	-2.749635000
H	-0.854884000	2.093792000	-3.800186000
H	-2.160267000	1.907977000	-2.625837000
C	-0.376535000	2.845050000	-1.809573000
H	-0.794874000	3.855707000	-1.906827000
H	0.697231000	2.910916000	-2.031954000
C	0.802389000	-0.273140000	-3.516229000
H	1.655343000	0.376714000	-3.298925000
H	0.483698000	-0.137547000	-4.556244000
H	1.104806000	-1.313650000	-3.356059000
C	-1.958462000	-0.908431000	-2.984284000
H	-2.852931000	-0.707649000	-2.385017000
H	-1.690596000	-1.965375000	-2.877639000
H	-2.167553000	-0.690554000	-4.038448000
C	0.627495000	3.338132000	0.892985000
H	1.658122000	3.163022000	0.570329000
H	0.538749000	3.085789000	1.955308000
H	0.367267000	4.393434000	0.747432000
C	-2.167583000	2.725036000	0.522400000
H	-2.931889000	2.177623000	-0.038814000
H	-2.251864000	2.455240000	1.580947000
H	-2.331071000	3.803423000	0.407537000
O	-0.917443000	-0.914550000	1.337376000
N	-0.571139000	-2.093949000	1.768937000
O	-1.501075000	-2.880879000	1.934241000

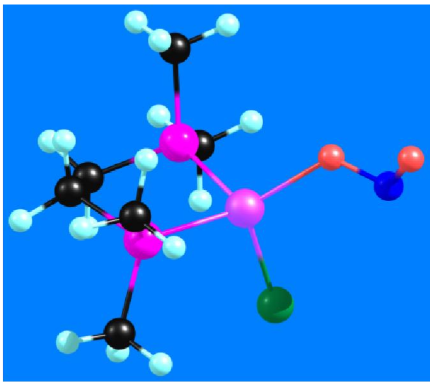

$\{\mathbf{ONO}_{cis}\}^{3}_{HS}$: **ONO_sr2a:**

Electrostatic Energy:	-2480.21	-10377.18
Kinetic Energy:	3060.63	12805.68
Coulomb (Steric+OrbInt) Energy:	-950.94	-3978.74
XC Energy:	-3101.80	-12977.92
Solvation:	-27.42	-114.72
	----------	-----------
Total Bonding Energy:	-3499.73	-14642.88

E(B3LYP): -4026.45

Ni	8.127439000	9.028576000	2.574999000
Cl	8.866499000	10.037503000	0.649373000
P	9.392621000	9.602558000	4.376572000
P	9.272212000	7.050069000	2.500570000
C	10.339023000	8.089912000	4.844714000
H	11.218801000	8.375567000	5.436277000
H	9.681461000	7.505926000	5.502687000
C	10.727598000	7.283937000	3.609342000
H	11.152348000	6.309164000	3.883387000
H	11.485228000	7.815362000	3.017438000
C	10.636402000	10.896152000	4.044041000
H	11.244964000	10.617182000	3.178449000
H	11.280217000	11.037726000	4.920709000
H	10.117129000	11.833953000	3.817160000
C	8.591657000	10.144673000	5.923684000
H	7.847355000	9.404378000	6.234348000
H	8.090306000	11.104100000	5.755948000
H	9.341400000	10.262752000	6.715344000
C	9.975697000	6.435702000	0.932237000
H	10.576771000	7.224683000	0.470411000
H	9.156837000	6.173457000	0.253674000
H	10.598300000	5.551317000	1.110249000
C	8.373672000	5.605129000	3.171431000
H	7.983987000	5.830545000	4.169329000
H	7.528563000	5.379317000	2.511655000
H	9.036723000	4.733658000	3.225380000
O	6.416927000	8.429065000	3.573268000
O	6.368937000	10.413110000	2.869631000
N	5.740320000	9.502959000	3.464799000

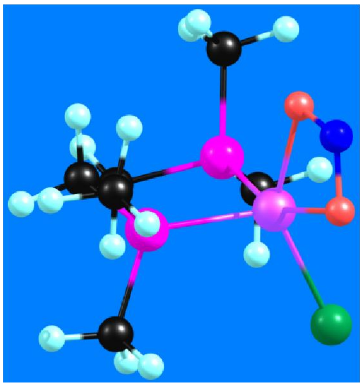

$\{\mathbf{ONO}_{cis}\}^{1}_{LS}$: **ONO_sr1:**

**Electrostatic Energy:**	**-2563.79**	**-10726.91**
**Kinetic Energy:**	**3090.74**	**12931.64**
**Coulomb (Steric+OrbInt) Energy:**	**-878.63**	**-3676.17**
**XC Energy:**	**-3128.18**	**-13088.30**
**Solvation:**	**-33.27**	**-139.19**
	**----------**	**-----------**
**Total Bonding Energy:**	**-3513.13**	**-14698.93**

E(B3LYP): -4028.44

Ni	8.422585000	9.101437000	2.615416000
Cl	7.708575000	8.895405000	0.509516000
P	9.283550000	9.337719000	4.563771000
P	9.607073000	7.316446000	2.423270000
C	10.169076000	7.805418000	5.065220000
H	10.908071000	8.034167000	5.843496000
H	9.415500000	7.142067000	5.510355000
C	10.794674000	7.178064000	3.829389000
H	11.072930000	6.128648000	3.986711000
H	11.702784000	7.717514000	3.530187000
C	10.512433000	10.685740000	4.559072000
H	11.297761000	10.485110000	3.822921000
H	10.957855000	10.782750000	5.556726000
H	10.008833000	11.621609000	4.293741000
C	8.190093000	9.733249000	5.966133000
H	7.402351000	8.978080000	6.045767000
H	7.731990000	10.714899000	5.812086000
H	8.768770000	9.758696000	6.896834000
C	10.640476000	7.092514000	0.939977000
H	11.292588000	7.962720000	0.812012000
H	10.004307000	6.986722000	0.057060000
H	11.253567000	6.191964000	1.064662000
C	8.561730000	5.823747000	2.496676000
H	8.031582000	5.783792000	3.451560000
H	7.830323000	5.861903000	1.684018000
H	9.185451000	4.930287000	2.387453000
O	5.819447000	9.455747000	3.477461000
O	7.403977000	10.756188000	2.841756000
N	6.181506000	10.608419000	3.221538000

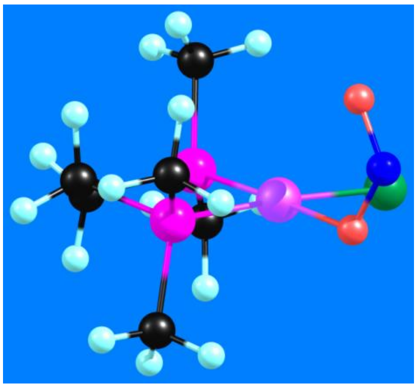

```
data_bath803

_audit_creation_method            SHELXL-97
_chemical_name_systematic
;
(1,2-Bis(diphenylphosphino)ethane)bis(?1-NO2)nickel(II) hydrate
;
_chemical_name_common             ?
_chemical_melting_point           ?
_chemical_formula_moiety          'C26 H24 N2 Ni O4 P2, O'
_chemical_formula_sum                'C26 H24 N2 Ni O5 P2'
_chemical_formula_weight          565.12

loop_
 _atom_type_symbol
 _atom_type_description
 _atom_type_scat_dispersion_real
 _atom_type_scat_dispersion_imag
 _atom_type_scat_source
 'C'  'C'   0.0033   0.0016
 'International Tables Vol C Tables 4.2.6.8 and 6.1.1.4'
 'H'  'H'   0.0000   0.0000
 'International Tables Vol C Tables 4.2.6.8 and 6.1.1.4'
 'N'  'N'   0.0061   0.0033
 'International Tables Vol C Tables 4.2.6.8 and 6.1.1.4'
 'O'  'O'   0.0106   0.0060
 'International Tables Vol C Tables 4.2.6.8 and 6.1.1.4'
 'Ni'  'Ni'   0.3393   1.1124
 'International Tables Vol C Tables 4.2.6.8 and 6.1.1.4'
 'P'  'P'   0.1023   0.0942
 'International Tables Vol C Tables 4.2.6.8 and 6.1.1.4'
 'Cl'  'Cl'   0.1484   0.1585
 'International Tables Vol C Tables 4.2.6.8 and 6.1.1.4'

_symmetry_cell_setting            orthorhombic
_symmetry_space_group_name_H-M    'P 21 21 21'

loop_
 _symmetry_equiv_pos_as_xyz
 'x, y, z'
 '-x+1/2, -y, z+1/2'
 'x+1/2, -y+1/2, -z'
 '-x, y+1/2, -z+1/2'

_cell_length_a                    8.1137(2)
_cell_length_b                    13.7208(4)
_cell_length_c                    22.6701(7)
_cell_angle_alpha                 90.00
_cell_angle_beta                  90.00
_cell_angle_gamma                 90.00
_cell_volume                      2523.78(12)
_cell_formula_units_Z             4
_cell_measurement_temperature     100(2)
_cell_measurement_reflns_used     7279
_cell_measurement_theta_min       2.61
_cell_measurement_theta_max       28.13
```

```
_exptl_crystal_description        block
_exptl_crystal_colour             red/orange
_exptl_crystal_size_max           0.08
_exptl_crystal_size_mid           0.08
_exptl_crystal_size_min           0.04
_exptl_crystal_density_meas       ?
_exptl_crystal_density_diffrn     1.487
_exptl_crystal_density_method     'not measured'
_exptl_crystal_F_000              1168
_exptl_absorpt_coefficient_mu     0.936
_exptl_absorpt_correction_T_min   0.6193
_exptl_absorpt_correction_T_max   0.7459
_exptl_absorpt_correction_type    'multi-scan'
_exptl_absorpt_process_details    'SADABS - Bruker2004'

_exptl_special_details
;
multi-scan from symmetry-related measurements
 SADABS - Bruker2004
;

_diffrn_ambient_temperature       100(2)
_diffrn_radiation_wavelength      0.68960
_diffrn_radiation_type            Synchrotron
_diffrn_radiation_source          'Station 9.8 Daresbury Laboratory'
_diffrn_radiation_monochromator   'Si (111)'
_diffrn_measurement_device_type   'Bruker APEX2'
_diffrn_measurement_method        '1800 0.36 degree images with \w
scans'
_diffrn_detector_area_resol_mean  ?
_diffrn_standards_number          ?
_diffrn_standards_interval_count  0
_diffrn_standards_interval_time   ?
_diffrn_standards_decay_%         0
_diffrn_reflns_number             28401
_diffrn_reflns_av_R_equivalents   0.0517
_diffrn_reflns_av_sigmaI/netI     0.0516
_diffrn_reflns_limit_h_min        -11
_diffrn_reflns_limit_h_max        11
_diffrn_reflns_limit_k_min        -19
_diffrn_reflns_limit_k_max        19
_diffrn_reflns_limit_l_min        -32
_diffrn_reflns_limit_l_max        32
_diffrn_reflns_theta_min          1.70
_diffrn_reflns_theta_max          29.73
_reflns_number_total              7512
_reflns_number_gt                 6856
_reflns_threshold_expression      >2sigma(I)

_computing_data_collection      'Bruker SMART'
_computing_cell_refinement      'Bruker SMART'
_computing_data_reduction       'Bruker SAINT'
_computing_structure_solution   'SHELXTS (Sheldrick, 1997)'
_computing_structure_refinement 'SHELXTL (Sheldrick, 1997)'
_computing_molecular_graphics   'ORTEP'
_computing_publication_material 'SHELXTL (Sheldrick, 1997)'
```

```
_refine_special_details
;
 Refinement of F^2^ against ALL reflections.  The weighted R-factor wR
and
 goodness of fit S are based on F^2^, conventional R-factors R are
based
 on F, with F set to zero for negative F^2^. The threshold expression
of
 F^2^ > 2sigma(F^2^) is used only for calculating R-factors(gt) etc.
and is
 not relevant to the choice of reflections for refinement.  R-factors
based
 on F^2^ are statistically about twice as large as those based on F,
and R-
 factors based on ALL data will be even larger.
;

_refine_ls_structure_factor_coef  Fsqd
_refine_ls_matrix_type            full
_refine_ls_weighting_scheme       calc
_refine_ls_weighting_details
 'calc w=1/[\s^2^(Fo^2^)+(0.0642P)^2^+0.3158P] where
P=(Fo^2^+2Fc^2^)/3'
_atom_sites_solution_primary      direct
_atom_sites_solution_secondary    difmap
_atom_sites_solution_hydrogens    geom
_refine_ls_hydrogen_treatment     constr
_refine_ls_extinction_method      none
_refine_ls_extinction_coef        ?
_refine_ls_abs_structure_details
 'Flack H D (1983), Acta Cryst. A39, 876-881'
_refine_ls_abs_structure_Flack    0.023(11)
_refine_ls_number_reflns          7512
_refine_ls_number_parameters      325
_refine_ls_number_restraints      0
_refine_ls_R_factor_all           0.0472
_refine_ls_R_factor_gt            0.0427
_refine_ls_wR_factor_ref          0.1141
_refine_ls_wR_factor_gt           0.1106
_refine_ls_goodness_of_fit_ref    1.049
_refine_ls_restrained_S_all       1.049
_refine_ls_shift/su_max           0.001
_refine_ls_shift/su_mean          0.000

loop_
 _atom_site_label
 _atom_site_type_symbol
 _atom_site_fract_x
 _atom_site_fract_y
 _atom_site_fract_z
 _atom_site_U_iso_or_equiv
 _atom_site_adp_type
 _atom_site_occupancy
 _atom_site_symmetry_multiplicity
 _atom_site_calc_flag
 _atom_site_refinement_flags
```

```
 _atom_site_disorder_assembly
 _atom_site_disorder_group
Ni1 Ni 0.88611(4) 0.43959(2) 0.850253(14) 0.01807(8) Uani 1 1 d . . .
P2 P 0.69908(8) 0.36513(5) 0.79765(3) 0.01788(13) Uani 1 1 d . . .
P1 P 0.68279(8) 0.52345(5) 0.88602(3) 0.01873(13) Uani 1 1 d . . .
C1 C 0.5188(3) 0.5246(2) 0.83080(12) 0.0212(5) Uani 1 1 d . . .
H1A H 0.4158 0.5511 0.8478 0.025 Uiso 1 1 calc R . .
H1B H 0.5509 0.5651 0.7965 0.025 Uiso 1 1 calc R . .
C2 C 0.4950(3) 0.4175(2) 0.81206(12) 0.0219(5) Uani 1 1 d . . .
H2A H 0.4261 0.4140 0.7761 0.026 Uiso 1 1 calc R . .
H2B H 0.4390 0.3807 0.8438 0.026 Uiso 1 1 calc R . .
C21 C 0.6835(3) 0.23504(18) 0.81012(13) 0.0240(5) Uani 1 1 d . . .
C26 C 0.5906(4) 0.2014(2) 0.85766(15) 0.0352(7) Uani 1 1 d . . .
H26 H 0.5341 0.2463 0.8823 0.042 Uiso 1 1 calc R . .
C25 C 0.5807(5) 0.1016(2) 0.86888(16) 0.0399(8) Uani 1 1 d . . .
H25 H 0.5181 0.0782 0.9013 0.048 Uiso 1 1 calc R . .
C24 C 0.6628(4) 0.0369(2) 0.83243(17) 0.0425(9) Uani 1 1 d . . .
H24 H 0.6528 -0.0312 0.8391 0.051 Uiso 1 1 calc R . .
C23 C 0.7583(4) 0.0700(2) 0.78674(17) 0.0392(8) Uani 1 1 d . . .
H23 H 0.8164 0.0246 0.7629 0.047 Uiso 1 1 calc R . .
C22 C 0.7712(3) 0.1699(2) 0.77480(15) 0.0291(6) Uani 1 1 d . . .
H22 H 0.8382 0.1928 0.7433 0.035 Uiso 1 1 calc R . .
C15 C 0.7296(3) 0.3872(2) 0.71957(12) 0.0224(5) Uani 1 1 d . . .
C20 C 0.6524(4) 0.3303(2) 0.67665(13) 0.0319(6) Uani 1 1 d . . .
H20 H 0.5892 0.2748 0.6876 0.038 Uiso 1 1 calc R . .
C19 C 0.6694(5) 0.3561(3) 0.61760(14) 0.0437(9) Uani 1 1 d . . .
H19 H 0.6206 0.3167 0.5879 0.052 Uiso 1 1 calc R . .
C18 C 0.7570(5) 0.4389(3) 0.60180(13) 0.0441(9) Uani 1 1 d . . .
H18 H 0.7649 0.4568 0.5614 0.053 Uiso 1 1 calc R . .
C17 C 0.8328(4) 0.4955(3) 0.64409(14) 0.0389(8) Uani 1 1 d . . .
H17 H 0.8934 0.5518 0.6330 0.047 Uiso 1 1 calc R . .
C16 C 0.8193(4) 0.4691(2) 0.70331(12) 0.0263(5) Uani 1 1 d . . .
H16 H 0.8717 0.5074 0.7327 0.032 Uiso 1 1 calc R . .
C3 C 0.7157(3) 0.64812(19) 0.90916(13) 0.0254(5) Uani 1 1 d . . .
C4 C 0.6548(4) 0.6843(2) 0.96148(14) 0.0312(6) Uani 1 1 d . . .
H4 H 0.5927 0.6435 0.9870 0.037 Uiso 1 1 calc R . .
C5 C 0.6848(5) 0.7817(2) 0.97692(18) 0.0486(10) Uani 1 1 d . . .
H5 H 0.6430 0.8070 1.0129 0.058 Uiso 1 1 calc R . .
C6 C 0.7747(5) 0.8403(2) 0.9398(2) 0.0546(12) Uani 1 1 d . . .
H6 H 0.7947 0.9062 0.9504 0.065 Uiso 1 1 calc R . .
C7 C 0.8361(4) 0.8048(2) 0.8876(2) 0.0504(11) Uani 1 1 d . . .
H7 H 0.8975 0.8462 0.8622 0.060 Uiso 1 1 calc R . .
C8 C 0.8082(4) 0.7080(2) 0.87186(18) 0.0379(8) Uani 1 1 d . . .
H8 H 0.8518 0.6829 0.8360 0.045 Uiso 1 1 calc R . .
C9 C 0.6005(3) 0.45351(17) 0.94759(10) 0.0194(5) Uani 1 1 d . . .
C10 C 0.4331(3) 0.4586(2) 0.96168(12) 0.0239(5) Uani 1 1 d . . .
H10 H 0.3625 0.5018 0.9408 0.029 Uiso 1 1 calc R . .
C11 C 0.3704(4) 0.3998(2) 1.00652(12) 0.0280(6) Uani 1 1 d . . .
H11 H 0.2569 0.4036 1.0166 0.034 Uiso 1 1 calc R . .
C12 C 0.4722(4) 0.3365(2) 1.03614(12) 0.0278(6) Uani 1 1 d . . .
H12 H 0.4282 0.2966 1.0665 0.033 Uiso 1 1 calc R . .
C13 C 0.6382(4) 0.3301(2) 1.02229(13) 0.0294(6) Uani 1 1 d . . .
H13 H 0.7076 0.2861 1.0430 0.035 Uiso 1 1 calc R . .
C14 C 0.7026(4) 0.38876(19) 0.97769(12) 0.0247(5) Uani 1 1 d . . .
H14 H 0.8161 0.3846 0.9678 0.030 Uiso 1 1 calc R . .
O5 O 0.6900(8) 0.6895(5) 0.7445(3) 0.151(2) Uani 1 1 d . . .
O3 O 1.1127(3) 0.2959(2) 0.85901(12) 0.0492(7) Uani 1 1 d . . .
```

```
N2 N 1.0600(3) 0.35037(16) 0.82267(9) 0.0189(4) Uani 1 1 d . . .
N1 N 1.0430(3) 0.51349(18) 0.89370(11) 0.0260(5) Uani 1 1 d . . .
O1 O 1.0528(3) 0.50620(19) 0.94818(10) 0.0390(6) Uani 1 1 d . . .
O2 O 1.1335(3) 0.56973(16) 0.86663(11) 0.0368(5) Uani 1 1 d . . .
O4 O 1.1093(3) 0.35634(17) 0.77202(9) 0.0357(5) Uani 1 1 d . . .

loop_
 _atom_site_aniso_label
 _atom_site_aniso_U_11
 _atom_site_aniso_U_22
 _atom_site_aniso_U_33
 _atom_site_aniso_U_23
 _atom_site_aniso_U_13
 _atom_site_aniso_U_12
Ni1 0.01341(14) 0.01799(14) 0.02283(14) 0.00026(12) -0.00034(12) -
0.00020(12)
P2 0.0158(3) 0.0164(3) 0.0214(3) 0.0004(2) -0.0010(2) -0.0017(2)
P1 0.0141(3) 0.0161(3) 0.0260(3) -0.0002(2) 0.0006(2) -0.0003(2)
C1 0.0159(11) 0.0203(11) 0.0276(12) 0.0032(10) -0.0011(9) 0.0007(9)
C2 0.0148(11) 0.0259(13) 0.0251(11) -0.0005(10) -0.0020(9) -0.0020(9)
C21 0.0199(12) 0.0163(11) 0.0358(14) 0.0006(10) -0.0059(11) -0.0036(9)
C26 0.0384(17) 0.0277(14) 0.0394(17) 0.0077(12) -0.0003(14) -
0.0048(12)
C25 0.043(2) 0.0287(15) 0.0479(18) 0.0137(13) -0.0118(15) -0.0155(14)
C24 0.0423(19) 0.0213(14) 0.064(2) 0.0089(13) -0.0281(16) -0.0062(12)
C23 0.0346(16) 0.0220(14) 0.061(2) -0.0044(14) -0.0209(15) 0.0049(12)
C22 0.0190(13) 0.0253(13) 0.0431(16) -0.0029(12) -0.0072(11)
0.0020(10)
C15 0.0227(13) 0.0234(12) 0.0211(11) -0.0022(9) 0.0008(9) 0.0046(10)
C20 0.0325(16) 0.0347(15) 0.0286(14) -0.0054(12) -0.0096(12)
0.0041(12)
C19 0.051(2) 0.054(2) 0.0265(14) -0.0110(14) -0.0099(14) 0.0211(17)
C18 0.052(2) 0.057(2) 0.0228(13) 0.0054(15) 0.0065(13) 0.0242(19)
C17 0.0379(17) 0.0475(19) 0.0314(15) 0.0160(14) 0.0121(13) 0.0121(14)
C16 0.0258(13) 0.0278(13) 0.0254(12) 0.0038(10) 0.0023(11) 0.0036(11)
C3 0.0189(12) 0.0167(11) 0.0407(15) -0.0017(10) -0.0068(11) 0.0012(9)
C4 0.0372(17) 0.0204(12) 0.0358(15) -0.0045(11) -0.0129(12) 0.0055(11)
C5 0.068(3) 0.0228(15) 0.055(2) -0.0120(14) -0.034(2) 0.0121(16)
C6 0.052(2) 0.0148(13) 0.097(3) -0.0057(17) -0.042(2) 0.0000(14)
C7 0.0307(17) 0.0218(14) 0.099(3) 0.0072(18) -0.012(2) -0.0048(12)
C8 0.0241(14) 0.0213(13) 0.068(2) 0.0062(13) 0.0017(15) -0.0038(11)
C9 0.0197(11) 0.0171(11) 0.0215(10) -0.0031(8) -0.0004(9) 0.0004(9)
C10 0.0194(12) 0.0266(13) 0.0257(12) -0.0011(10) 0.0010(9) -0.0012(9)
C11 0.0254(14) 0.0300(13) 0.0286(13) -0.0029(11) 0.0055(11) -
0.0056(12)
C12 0.0368(16) 0.0228(12) 0.0238(12) -0.0003(10) 0.0026(11) -
0.0075(11)
C13 0.0379(17) 0.0218(12) 0.0284(13) 0.0015(10) -0.0007(12) 0.0021(12)
C14 0.0240(13) 0.0237(12) 0.0263(12) -0.0008(10) 0.0018(10) 0.0030(10)
O5 0.150(5) 0.161(6) 0.141(5) 0.059(4) -0.017(4) -0.048(5)
O3 0.0453(14) 0.0510(14) 0.0513(15) 0.0203(12) 0.0095(13) 0.0214(12)
N2 0.0135(9) 0.0228(10) 0.0205(10) 0.0039(8) -0.0004(7) -0.0077(8)
N1 0.0167(10) 0.0257(11) 0.0355(12) -0.0049(10) -0.0029(9) 0.0023(9)
O1 0.0270(11) 0.0538(15) 0.0361(12) -0.0096(10) -0.0061(9) -0.0044(10)
O2 0.0214(10) 0.0296(11) 0.0593(14) 0.0000(10) 0.0010(9) -0.0044(9)
O4 0.0319(11) 0.0396(12) 0.0356(11) -0.0008(9) 0.0025(10) 0.0044(10)
```

```
_geom_special_details
;
 All esds (except the esd in the dihedral angle between two l.s.
planes)
 are estimated using the full covariance matrix.  The cell esds are
taken
 into account individually in the estimation of esds in distances,
angles
 and torsion angles; correlations between esds in cell parameters are
only
 used when they are defined by crystal symmetry.  An approximate
(isotropic)
 treatment of cell esds is used for estimating esds involving l.s.
planes.
;

loop_
 _geom_bond_atom_site_label_1
 _geom_bond_atom_site_label_2
 _geom_bond_distance
 _geom_bond_site_symmetry_2
 _geom_bond_publ_flag
Ni1 N1 1.902(2) . ?
Ni1 N2 1.970(2) . ?
Ni1 P1 2.1686(7) . ?
Ni1 P2 2.1837(7) . ?
P2 C21 1.812(3) . ?
P2 C15 1.813(3) . ?
P2 C2 1.835(3) . ?
P1 C3 1.809(3) . ?
P1 C9 1.821(3) . ?
P1 C1 1.827(3) . ?
C1 C2 1.542(4) . ?
C1 H1A 0.9900 . ?
C1 H1B 0.9900 . ?
C2 H2A 0.9900 . ?
C2 H2B 0.9900 . ?
C21 C26 1.394(4) . ?
C21 C22 1.395(4) . ?
C26 C25 1.395(4) . ?
C26 H26 0.9500 . ?
C25 C24 1.384(6) . ?
C25 H25 0.9500 . ?
C24 C23 1.371(5) . ?
C24 H24 0.9500 . ?
C23 C22 1.402(4) . ?
C23 H23 0.9500 . ?
C22 H22 0.9500 . ?
C15 C16 1.389(4) . ?
C15 C20 1.396(4) . ?
C20 C19 1.392(4) . ?
C20 H20 0.9500 . ?
C19 C18 1.388(6) . ?
C19 H19 0.9500 . ?
C18 C17 1.378(5) . ?
C18 H18 0.9500 . ?
C17 C16 1.395(4) . ?
```

```
C17 H17 0.9500 . ?
C16 H16 0.9500 . ?
C3 C4 1.378(4) . ?
C3 C8 1.398(4) . ?
C4 C5 1.402(4) . ?
C4 H4 0.9500 . ?
C5 C6 1.374(7) . ?
C5 H5 0.9500 . ?
C6 C7 1.373(7) . ?
C6 H6 0.9500 . ?
C7 C8 1.394(5) . ?
C7 H7 0.9500 . ?
C8 H8 0.9500 . ?
C9 C14 1.393(4) . ?
C9 C10 1.397(4) . ?
C10 C11 1.393(4) . ?
C10 H10 0.9500 . ?
C11 C12 1.374(4) . ?
C11 H11 0.9500 . ?
C12 C13 1.386(5) . ?
C12 H12 0.9500 . ?
C13 C14 1.394(4) . ?
C13 H13 0.9500 . ?
C14 H14 0.9500 . ?
O3 N2 1.192(3) . ?
N2 O4 1.219(3) . ?
N1 O2 1.229(3) . ?
N1 O1 1.242(3) . ?

loop_
 _geom_angle_atom_site_label_1
 _geom_angle_atom_site_label_2
 _geom_angle_atom_site_label_3
 _geom_angle
 _geom_angle_site_symmetry_1
 _geom_angle_site_symmetry_3
 _geom_angle_publ_flag
N1 Ni1 N2 90.93(10) . . ?
N1 Ni1 P1 91.87(7) . . ?
N2 Ni1 P1 173.36(6) . . ?
N1 Ni1 P2 175.67(8) . . ?
N2 Ni1 P2 91.94(6) . . ?
P1 Ni1 P2 85.63(3) . . ?
C21 P2 C15 109.04(13) . . ?
C21 P2 C2 107.17(13) . . ?
C15 P2 C2 103.41(12) . . ?
C21 P2 Ni1 115.10(9) . . ?
C15 P2 Ni1 111.11(10) . . ?
C2 P2 Ni1 110.30(9) . . ?
C3 P1 C9 109.28(13) . . ?
C3 P1 C1 107.33(12) . . ?
C9 P1 C1 105.25(12) . . ?
C3 P1 Ni1 119.86(10) . . ?
C9 P1 Ni1 106.62(8) . . ?
C1 P1 Ni1 107.60(9) . . ?
C2 C1 P1 105.78(17) . . ?
C2 C1 H1A 110.6 . . ?
```

P1 C1 H1A 110.6 . . ?
C2 C1 H1B 110.6 . . ?
P1 C1 H1B 110.6 . . ?
H1A C1 H1B 108.7 . . ?
C1 C2 P2 107.99(17) . . ?
C1 C2 H2A 110.1 . . ?
P2 C2 H2A 110.1 . . ?
C1 C2 H2B 110.1 . . ?
P2 C2 H2B 110.1 . . ?
H2A C2 H2B 108.4 . . ?
C26 C21 C22 120.5(3) . . ?
C26 C21 P2 119.0(2) . . ?
C22 C21 P2 120.4(2) . . ?
C21 C26 C25 119.8(3) . . ?
C21 C26 H26 120.1 . . ?
C25 C26 H26 120.1 . . ?
C24 C25 C26 119.6(3) . . ?
C24 C25 H25 120.2 . . ?
C26 C25 H25 120.2 . . ?
C23 C24 C25 120.7(3) . . ?
C23 C24 H24 119.7 . . ?
C25 C24 H24 119.7 . . ?
C24 C23 C22 120.8(3) . . ?
C24 C23 H23 119.6 . . ?
C22 C23 H23 119.6 . . ?
C21 C22 C23 118.5(3) . . ?
C21 C22 H22 120.7 . . ?
C23 C22 H22 120.7 . . ?
C16 C15 C20 120.2(3) . . ?
C16 C15 P2 117.8(2) . . ?
C20 C15 P2 121.7(2) . . ?
C19 C20 C15 118.9(3) . . ?
C19 C20 H20 120.5 . . ?
C15 C20 H20 120.5 . . ?
C18 C19 C20 120.5(3) . . ?
C18 C19 H19 119.8 . . ?
C20 C19 H19 119.8 . . ?
C17 C18 C19 120.7(3) . . ?
C17 C18 H18 119.7 . . ?
C19 C18 H18 119.7 . . ?
C18 C17 C16 119.2(3) . . ?
C18 C17 H17 120.4 . . ?
C16 C17 H17 120.4 . . ?
C15 C16 C17 120.4(3) . . ?
C15 C16 H16 119.8 . . ?
C17 C16 H16 119.8 . . ?
C4 C3 C8 120.1(3) . . ?
C4 C3 P1 122.5(2) . . ?
C8 C3 P1 117.4(2) . . ?
C3 C4 C5 119.8(3) . . ?
C3 C4 H4 120.1 . . ?
C5 C4 H4 120.1 . . ?
C6 C5 C4 119.8(4) . . ?
C6 C5 H5 120.1 . . ?
C4 C5 H5 120.1 . . ?
C7 C6 C5 120.9(3) . . ?
C7 C6 H6 119.6 . . ?

```
C5 C6 H6 119.6 . . ?
C6 C7 C8 120.0(4) . . ?
C6 C7 H7 120.0 . . ?
C8 C7 H7 120.0 . . ?
C7 C8 C3 119.5(4) . . ?
C7 C8 H8 120.2 . . ?
C3 C8 H8 120.2 . . ?
C14 C9 C10 119.8(2) . . ?
C14 C9 P1 119.6(2) . . ?
C10 C9 P1 120.37(19) . . ?
C11 C10 C9 119.6(3) . . ?
C11 C10 H10 120.2 . . ?
C9 C10 H10 120.2 . . ?
C12 C11 C10 120.1(3) . . ?
C12 C11 H11 119.9 . . ?
C10 C11 H11 119.9 . . ?
C11 C12 C13 120.9(3) . . ?
C11 C12 H12 119.5 . . ?
C13 C12 H12 119.5 . . ?
C12 C13 C14 119.4(3) . . ?
C12 C13 H13 120.3 . . ?
C14 C13 H13 120.3 . . ?
C9 C14 C13 120.1(3) . . ?
C9 C14 H14 120.0 . . ?
C13 C14 H14 120.0 . . ?
O3 N2 O4 125.2(3) . . ?
O3 N2 Ni1 115.3(2) . . ?
O4 N2 Ni1 119.48(19) . . ?
O2 N1 O1 120.6(3) . . ?
O2 N1 Ni1 118.4(2) . . ?
O1 N1 Ni1 121.0(2) . . ?

loop_
 _geom_torsion_atom_site_label_1
 _geom_torsion_atom_site_label_2
 _geom_torsion_atom_site_label_3
 _geom_torsion_atom_site_label_4
 _geom_torsion
 _geom_torsion_site_symmetry_1
 _geom_torsion_site_symmetry_2
 _geom_torsion_site_symmetry_3
 _geom_torsion_site_symmetry_4
 _geom_torsion_publ_flag
N1 Ni1 P2 C21 -174.3(10) . . . . ?
N2 Ni1 P2 C21 54.37(13) . . . . ?
P1 Ni1 P2 C21 -119.44(11) . . . . ?
N1 Ni1 P2 C15 61.2(10) . . . . ?
N2 Ni1 P2 C15 -70.17(11) . . . . ?
P1 Ni1 P2 C15 116.02(10) . . . . ?
N1 Ni1 P2 C2 -52.9(11) . . . . ?
N2 Ni1 P2 C2 175.76(11) . . . . ?
P1 Ni1 P2 C2 1.95(10) . . . . ?
N1 Ni1 P1 C3 27.46(13) . . . . ?
N2 Ni1 P1 C3 142.3(6) . . . . ?
P2 Ni1 P1 C3 -149.00(11) . . . . ?
N1 Ni1 P1 C9 -97.20(11) . . . . ?
N2 Ni1 P1 C9 17.7(6) . . . . ?
```

P2 Ni1 P1 C9 86.34(9) . . . . ?
N1 Ni1 P1 C1 150.31(12) . . . . ?
N2 Ni1 P1 C1 -94.8(6) . . . . ?
P2 Ni1 P1 C1 -26.15(9) . . . . ?
C3 P1 C1 C2 179.78(18) . . . . ?
C9 P1 C1 C2 -63.89(19) . . . . ?
Ni1 P1 C1 C2 49.53(18) . . . . ?
P1 C1 C2 P2 -47.1(2) . . . . ?
C21 P2 C2 C1 153.56(18) . . . . ?
C15 P2 C2 C1 -91.31(19) . . . . ?
Ni1 P2 C2 C1 27.57(19) . . . . ?
C15 P2 C21 C26 -151.4(2) . . . . ?
C2 P2 C21 C26 -40.1(3) . . . . ?
Ni1 P2 C21 C26 83.0(2) . . . . ?
C15 P2 C21 C22 32.4(3) . . . . ?
C2 P2 C21 C22 143.7(2) . . . . ?
Ni1 P2 C21 C22 -93.2(2) . . . . ?
C22 C21 C26 C25 -2.1(5) . . . . ?
P2 C21 C26 C25 -178.3(3) . . . . ?
C21 C26 C25 C24 -0.3(5) . . . . ?
C26 C25 C24 C23 2.3(5) . . . . ?
C25 C24 C23 C22 -1.9(5) . . . . ?
C26 C21 C22 C23 2.6(4) . . . . ?
P2 C21 C22 C23 178.7(2) . . . . ?
C24 C23 C22 C21 -0.6(4) . . . . ?
C21 P2 C15 C16 -149.9(2) . . . . ?
C2 P2 C15 C16 96.3(2) . . . . ?
Ni1 P2 C15 C16 -22.0(2) . . . . ?
C21 P2 C15 C20 36.6(3) . . . . ?
C2 P2 C15 C20 -77.1(3) . . . . ?
Ni1 P2 C15 C20 164.5(2) . . . . ?
C16 C15 C20 C19 1.2(4) . . . . ?
P2 C15 C20 C19 174.5(3) . . . . ?
C15 C20 C19 C18 -2.2(5) . . . . ?
C20 C19 C18 C17 1.9(5) . . . . ?
C19 C18 C17 C16 -0.6(5) . . . . ?
C20 C15 C16 C17 0.1(4) . . . . ?
P2 C15 C16 C17 -173.5(2) . . . . ?
C18 C17 C16 C15 -0.4(4) . . . . ?
C9 P1 C3 C4 -11.8(3) . . . . ?
C1 P1 C3 C4 101.9(2) . . . . ?
Ni1 P1 C3 C4 -135.1(2) . . . . ?
C9 P1 C3 C8 167.6(2) . . . . ?
C1 P1 C3 C8 -78.7(2) . . . . ?
Ni1 P1 C3 C8 44.2(3) . . . . ?
C8 C3 C4 C5 0.5(4) . . . . ?
P1 C3 C4 C5 179.9(2) . . . . ?
C3 C4 C5 C6 0.0(5) . . . . ?
C4 C5 C6 C7 -0.1(6) . . . . ?
C5 C6 C7 C8 -0.4(6) . . . . ?
C6 C7 C8 C3 0.9(5) . . . . ?
C4 C3 C8 C7 -1.0(5) . . . . ?
P1 C3 C8 C7 179.6(3) . . . . ?
C3 P1 C9 C14 -104.8(2) . . . . ?
C1 P1 C9 C14 140.2(2) . . . . ?
Ni1 P1 C9 C14 26.1(2) . . . . ?
C3 P1 C9 C10 80.3(2) . . . . ?

```
C1 P1 C9 C10 -34.7(2) . . . . ?
Ni1 P1 C9 C10 -148.76(19) . . . . ?
C14 C9 C10 C11 1.1(4) . . . . ?
P1 C9 C10 C11 175.9(2) . . . . ?
C9 C10 C11 C12 -0.8(4) . . . . ?
C10 C11 C12 C13 0.3(4) . . . . ?
C11 C12 C13 C14 0.0(4) . . . . ?
C10 C9 C14 C13 -0.9(4) . . . . ?
P1 C9 C14 C13 -175.7(2) . . . . ?
C12 C13 C14 C9 0.3(4) . . . . ?
N1 Ni1 N2 O3 72.6(2) . . . . ?
P1 Ni1 N2 O3 -42.3(7) . . . . ?
P2 Ni1 N2 O3 -110.7(2) . . . . ?
N1 Ni1 N2 O4 -105.0(2) . . . . ?
P1 Ni1 N2 O4 140.1(5) . . . . ?
P2 Ni1 N2 O4 71.8(2) . . . . ?
N2 Ni1 N1 O2 76.6(2) . . . . ?
P1 Ni1 N1 O2 -109.4(2) . . . . ?
P2 Ni1 N1 O2 -54.8(11) . . . . ?
N2 Ni1 N1 O1 -104.3(2) . . . . ?
P1 Ni1 N1 O1 69.7(2) . . . . ?
P2 Ni1 N1 O1 124.3(10) . . . . ?

_diffrn_measured_fraction_theta_max    0.987
_diffrn_reflns_theta_full              29.73
_diffrn_measured_fraction_theta_full   0.987
_refine_diff_density_max    0.789
_refine_diff_density_min   -0.712
_refine_diff_density_rms    0.085


data_bath806

_audit_creation_method            SHELXL-97
_chemical_name_systematic
;
(1,2-Bis(diphenylphosphino)ethane)bis(?1-NO2)nickel(II) hydrate
;
_chemical_name_common             ?
_chemical_melting_point           ?
_chemical_formula_moiety          'C26 H24 N2 Ni O4 P2, O'
_chemical_formula_sum                'C26 H24 N2 Ni O5 P2'
_chemical_formula_weight          565.12

loop_
 _atom_type_symbol
 _atom_type_description
 _atom_type_scat_dispersion_real
 _atom_type_scat_dispersion_imag
 _atom_type_scat_source
 'C'  'C'   0.0033   0.0016
 'International Tables Vol C Tables 4.2.6.8 and 6.1.1.4'
 'H'  'H'   0.0000   0.0000
 'International Tables Vol C Tables 4.2.6.8 and 6.1.1.4'
 'N'  'N'   0.0061   0.0033
 'International Tables Vol C Tables 4.2.6.8 and 6.1.1.4'
 'O'  'O'   0.0106   0.0060
```

```
 'International Tables Vol C Tables 4.2.6.8 and 6.1.1.4'
 'Ni'  'Ni'   0.3393   1.1124
 'International Tables Vol C Tables 4.2.6.8 and 6.1.1.4'
 'P'  'P'   0.1023   0.0942
 'International Tables Vol C Tables 4.2.6.8 and 6.1.1.4'

_symmetry_cell_setting            orthorhombic
_symmetry_space_group_name_H-M    'P 21 21 21'

loop_
 _symmetry_equiv_pos_as_xyz
 'x, y, z'
 '-x+1/2, -y, z+1/2'
 'x+1/2, -y+1/2, -z'
 '-x, y+1/2, -z+1/2'

_cell_length_a                    7.9997(5)
_cell_length_b                    13.9307(8)
_cell_length_c                    22.8956(14)
_cell_angle_alpha                 90.00
_cell_angle_beta                  90.00
_cell_angle_gamma                 90.00
_cell_volume                      2551.5(3)
_cell_formula_units_Z             4
_cell_measurement_temperature     100(2)
_cell_measurement_reflns_used     6033
_cell_measurement_theta_min       2.64
_cell_measurement_theta_max       29.41

_exptl_crystal_description        block
_exptl_crystal_colour             red/orange
_exptl_crystal_size_max           0.08
_exptl_crystal_size_mid           0.08
_exptl_crystal_size_min           0.04
_exptl_crystal_density_meas       ?
_exptl_crystal_density_diffrn     1.471
_exptl_crystal_density_method     'not measured'
_exptl_crystal_F_000              1168
_exptl_absorpt_coefficient_mu     0.926
_exptl_absorpt_correction_T_min   0.9296
_exptl_absorpt_correction_T_max   0.9639
_exptl_absorpt_correction_type    'multi-scan'
_exptl_absorpt_process_details    'SADABS - Bruker2004'

_exptl_special_details
;
multi-scan from symmetry-related measurements
 SADABS - Bruker2004
;

_diffrn_ambient_temperature       100(2)
_diffrn_radiation_wavelength      0.68960
_diffrn_radiation_type            Synchrotron
_diffrn_radiation_source          'Station 9.8 Daresbury Laboratory'
_diffrn_radiation_monochromator   'Si (111)'
_diffrn_measurement_device_type   'Bruker APEX2'
```

```
_diffrn_measurement_method        '1800 0.36 degree images with \w
scans'
_diffrn_detector_area_resol_mean  ?
_diffrn_standards_number          ?
_diffrn_standards_interval_count  ?
_diffrn_standards_interval_time   ?
_diffrn_standards_decay_%         0
_diffrn_reflns_number             27756
_diffrn_reflns_av_R_equivalents   0.0463
_diffrn_reflns_av_sigmaI/netI     0.0549
_diffrn_reflns_limit_h_min        -11
_diffrn_reflns_limit_h_max        11
_diffrn_reflns_limit_k_min        -19
_diffrn_reflns_limit_k_max        19
_diffrn_reflns_limit_l_min        -32
_diffrn_reflns_limit_l_max        32
_diffrn_reflns_theta_min          2.64
_diffrn_reflns_theta_max          29.84
_reflns_number_total              7527
_reflns_number_gt                 5301
_reflns_threshold_expression      >2sigma(I)

_computing_data_collection       'Bruker SMART'
_computing_cell_refinement       'Bruker SMART'
_computing_data_reduction        'Bruker SAINT'
_computing_structure_solution    'SHELXTS (Sheldrick, 1997)'
_computing_structure_refinement 'SHELXTL (Sheldrick, 1997)'
_computing_molecular_graphics    'ORTEP'
_computing_publication_material 'SHELXTL (Sheldrick, 1997)'

_refine_special_details
;
 Refinement of F^2^ against ALL reflections.  The weighted R-factor wR
and
 goodness of fit S are based on F^2^, conventional R-factors R are
based
 on F, with F set to zero for negative F^2^. The threshold expression
of
 F^2^ > 2sigma(F^2^) is used only for calculating R-factors(gt) etc.
and is
 not relevant to the choice of reflections for refinement.  R-factors
based
 on F^2^ are statistically about twice as large as those based on F,
and R-
 factors based on ALL data will be even larger.
;

_refine_ls_structure_factor_coef  Fsqd
_refine_ls_matrix_type            full
_refine_ls_weighting_scheme       calc
_refine_ls_weighting_details
 'calc w=1/[\s^2^(Fo^2^)+(0.1440P)^2^+0.0000P] where
P=(Fo^2^+2Fc^2^)/3'
_atom_sites_solution_primary      direct
_atom_sites_solution_secondary    difmap
_atom_sites_solution_hydrogens    geom
_refine_ls_hydrogen_treatment     constr
```

```
_refine_ls_extinction_method      none
_refine_ls_extinction_coef        ?
_refine_ls_abs_structure_details
 'Flack H D (1983), Acta Cryst. A39, 876-881'
_refine_ls_abs_structure_Flack    0.04(2)
_refine_ls_number_reflns          7527
_refine_ls_number_parameters      325
_refine_ls_number_restraints      0
_refine_ls_R_factor_all           0.1076
_refine_ls_R_factor_gt            0.0742
_refine_ls_wR_factor_ref          0.2198
_refine_ls_wR_factor_gt           0.1959
_refine_ls_goodness_of_fit_ref    1.062
_refine_ls_restrained_S_all       1.062
_refine_ls_shift/su_max           0.001
_refine_ls_shift/su_mean          0.000

loop_
 _atom_site_label
 _atom_site_type_symbol
 _atom_site_fract_x
 _atom_site_fract_y
 _atom_site_fract_z
 _atom_site_U_iso_or_equiv
 _atom_site_adp_type
 _atom_site_occupancy
 _atom_site_symmetry_multiplicity
 _atom_site_calc_flag
 _atom_site_refinement_flags
 _atom_site_disorder_assembly
 _atom_site_disorder_group
Ni1 Ni 0.90178(8) 0.44261(4) 0.83792(3) 0.04008(19) Uani 1 1 d . . .
P1 P 0.71001(16) 0.52277(8) 0.88241(6) 0.0351(3) Uani 1 1 d . . .
P2 P 0.70223(15) 0.36760(9) 0.79416(5) 0.0332(3) Uani 1 1 d . . .
O1 O 1.0885(5) 0.5226(3) 0.8589(2) 0.0610(11) Uani 1 1 d . . .
O2 O 1.0256(7) 0.5191(4) 0.9484(2) 0.0750(15) Uani 1 1 d . . .
O3 O 1.0567(5) 0.3665(4) 0.7885(2) 0.0606(11) Uani 1 1 d . . .
O4 O 1.0993(8) 0.3017(5) 0.8642(3) 0.095(2) Uani 1 1 d . . .
O5 O 0.680(3) 0.6752(19) 0.7352(7) 0.304(12) Uani 1 1 d . . .
N1 N 1.1184(7) 0.5504(4) 0.9091(3) 0.0635(14) Uani 1 1 d . . .
N2 N 1.1212(9) 0.3107(5) 0.8158(3) 0.0736(18) Uani 1 1 d . . .
C1 C 0.5317(6) 0.5248(4) 0.8316(2) 0.0380(10) Uani 1 1 d . . .
H1A H 0.4317 0.5522 0.8508 0.046 Uiso 1 1 calc R . .
H1B H 0.5584 0.5641 0.7968 0.046 Uiso 1 1 calc R . .
C2 C 0.4998(6) 0.4187(4) 0.8138(2) 0.0386(10) Uani 1 1 d . . .
H2A H 0.4221 0.4156 0.7802 0.046 Uiso 1 1 calc R . .
H2B H 0.4500 0.3828 0.8468 0.046 Uiso 1 1 calc R . .
C3 C 0.7488(7) 0.6464(4) 0.9018(2) 0.0440(11) Uani 1 1 d . . .
C4 C 0.7094(10) 0.6843(4) 0.9545(3) 0.0557(15) Uani 1 1 d . . .
H4 H 0.6535 0.6463 0.9829 0.067 Uiso 1 1 calc R . .
C5 C 0.7524(12) 0.7821(5) 0.9672(3) 0.071(2) Uani 1 1 d . . .
H5 H 0.7300 0.8086 1.0046 0.086 Uiso 1 1 calc R . .
C6 C 0.8269(11) 0.8370(4) 0.9242(4) 0.074(2) Uani 1 1 d . . .
H6 H 0.8537 0.9021 0.9323 0.089 Uiso 1 1 calc R . .
C7 C 0.8631(10) 0.8002(5) 0.8706(4) 0.0667(18) Uani 1 1 d . . .
H7 H 0.9155 0.8390 0.8418 0.080 Uiso 1 1 calc R . .
C8 C 0.8222(8) 0.7046(4) 0.8584(3) 0.0504(13) Uani 1 1 d . . .
```

```
H8 H 0.8439 0.6788 0.8207 0.061 Uiso 1 1 calc R . .
C9 C 0.6281(6) 0.4579(3) 0.9460(2) 0.0369(10) Uani 1 1 d . . .
C10 C 0.4672(7) 0.4746(5) 0.9659(2) 0.0448(12) Uani 1 1 d . . .
H10 H 0.4037 0.5259 0.9499 0.054 Uiso 1 1 calc R . .
C11 C 0.3992(9) 0.4175(4) 1.0084(3) 0.0541(14) Uani 1 1 d . . .
H11 H 0.2868 0.4272 1.0204 0.065 Uiso 1 1 calc R . .
C12 C 0.4941(10) 0.3458(5) 1.0337(3) 0.0587(17) Uani 1 1 d . . .
H12 H 0.4475 0.3069 1.0636 0.070 Uiso 1 1 calc R . .
C13 C 0.6593(10) 0.3303(4) 1.0152(3) 0.0601(17) Uani 1 1 d . . .
H13 H 0.7263 0.2828 1.0336 0.072 Uiso 1 1 calc R . .
C14 C 0.7233(8) 0.3846(4) 0.9701(2) 0.0442(12) Uani 1 1 d . . .
H14 H 0.8323 0.3720 0.9556 0.053 Uiso 1 1 calc R . .
C15 C 0.7166(7) 0.3881(4) 0.7161(2) 0.0409(11) Uani 1 1 d . . .
C16 C 0.8097(8) 0.4682(4) 0.6977(3) 0.0510(13) Uani 1 1 d . . .
H16 H 0.8710 0.5048 0.7254 0.061 Uiso 1 1 calc R . .
C17 C 0.8119(11) 0.4936(6) 0.6393(3) 0.075(2) Uani 1 1 d . . .
H17 H 0.8734 0.5484 0.6271 0.089 Uiso 1 1 calc R . .
C18 C 0.7278(13) 0.4415(7) 0.5997(3) 0.080(3) Uani 1 1 d . . .
H18 H 0.7307 0.4598 0.5598 0.096 Uiso 1 1 calc R . .
C19 C 0.6336(13) 0.3587(8) 0.6168(3) 0.091(3) Uani 1 1 d . . .
H19 H 0.5755 0.3212 0.5887 0.109 Uiso 1 1 calc R . .
C20 C 0.6296(9) 0.3344(5) 0.6770(2) 0.0570(15) Uani 1 1 d . . .
H20 H 0.5662 0.2808 0.6899 0.068 Uiso 1 1 calc R . .
C21 C 0.6916(7) 0.2392(3) 0.8055(2) 0.0403(11) Uani 1 1 d . . .
C22 C 0.7711(6) 0.1756(4) 0.7694(3) 0.0466(12) Uani 1 1 d . . .
H22 H 0.8316 0.1980 0.7364 0.056 Uiso 1 1 calc R . .
C23 C 0.7624(8) 0.0771(4) 0.7815(3) 0.0574(16) Uani 1 1 d . . .
H23 H 0.8152 0.0325 0.7561 0.069 Uiso 1 1 calc R . .
C24 C 0.6785(10) 0.0449(5) 0.8297(4) 0.072(2) Uani 1 1 d . . .
H24 H 0.6748 -0.0220 0.8377 0.087 Uiso 1 1 calc R . .
C25 C 0.6008(10) 0.1067(5) 0.8662(3) 0.0637(18) Uani 1 1 d . . .
H25 H 0.5430 0.0827 0.8994 0.076 Uiso 1 1 calc R . .
C26 C 0.6044(10) 0.2059(4) 0.8557(3) 0.0581(15) Uani 1 1 d . . .
H26 H 0.5501 0.2495 0.8814 0.070 Uiso 1 1 calc R . .

loop_
 _atom_site_aniso_label
 _atom_site_aniso_U_11
 _atom_site_aniso_U_22
 _atom_site_aniso_U_33
 _atom_site_aniso_U_23
 _atom_site_aniso_U_13
 _atom_site_aniso_U_12
Ni1 0.0263(3) 0.0371(3) 0.0569(4) -0.0051(3) -0.0028(3) -0.0038(2)
P1 0.0286(5) 0.0290(5) 0.0476(7) -0.0030(5) -0.0026(5) -0.0006(4)
P2 0.0275(5) 0.0315(5) 0.0407(6) -0.0011(5) -0.0027(5) -0.0082(4)
O1 0.0358(19) 0.060(2) 0.087(3) -0.015(2) -0.001(2) -0.0079(19)
O2 0.063(3) 0.085(4) 0.077(3) -0.007(3) -0.027(3) -0.012(3)
O3 0.041(2) 0.054(2) 0.087(3) -0.004(2) 0.000(2) -0.0071(19)
O4 0.062(3) 0.089(4) 0.135(5) 0.042(4) -0.023(4) -0.006(3)
O5 0.269(18) 0.43(3) 0.216(13) -0.133(18) -0.081(15) 0.12(2)
N1 0.040(3) 0.064(3) 0.086(4) -0.011(3) -0.019(3) 0.001(3)
N2 0.074(4) 0.074(4) 0.073(4) 0.021(3) 0.005(3) -0.026(4)
C1 0.036(2) 0.038(2) 0.040(2) -0.0010(19) -0.0061(19) 0.0033(18)
C2 0.029(2) 0.046(3) 0.041(2) 0.001(2) -0.0034(19) -0.0062(19)
C3 0.044(3) 0.030(2) 0.058(3) 0.001(2) -0.008(2) -0.0018(19)
C4 0.082(4) 0.030(2) 0.054(3) 0.003(2) -0.014(3) 0.002(3)
```

```
C5 0.102(6) 0.039(3) 0.073(4) -0.012(3) -0.031(4) 0.002(3)
C6 0.083(5) 0.027(2) 0.112(6) 0.000(3) -0.020(5) 0.003(3)
C7 0.071(5) 0.040(3) 0.089(5) -0.001(3) 0.006(4) -0.011(3)
C8 0.050(3) 0.033(2) 0.068(3) -0.004(2) 0.003(3) -0.008(2)
C9 0.037(2) 0.033(2) 0.041(2) -0.0041(17) 0.0009(19) -0.0018(18)
C10 0.037(2) 0.056(3) 0.042(3) -0.008(2) -0.005(2) -0.002(2)
C11 0.045(3) 0.056(3) 0.061(3) -0.016(3) 0.003(3) -0.010(3)
C12 0.080(5) 0.045(3) 0.051(3) -0.004(2) 0.009(3) -0.028(3)
C13 0.083(5) 0.036(3) 0.061(4) 0.000(3) -0.006(3) 0.004(3)
C14 0.052(3) 0.032(2) 0.049(3) -0.004(2) -0.003(2) 0.002(2)
C15 0.041(3) 0.040(2) 0.041(2) -0.0055(19) 0.002(2) 0.005(2)
C16 0.049(3) 0.057(3) 0.047(3) 0.013(2) 0.012(2) 0.007(3)
C17 0.076(5) 0.081(5) 0.067(4) 0.029(4) 0.030(4) 0.034(4)
C18 0.116(7) 0.084(5) 0.039(3) 0.004(4) 0.008(4) 0.049(5)
C19 0.119(8) 0.107(7) 0.047(3) -0.027(4) -0.029(4) 0.063(6)
C20 0.066(4) 0.059(3) 0.046(3) -0.008(3) -0.008(3) 0.018(3)
C21 0.038(2) 0.032(2) 0.051(3) 0.0022(19) -0.012(2) -0.0122(19)
C22 0.032(2) 0.036(2) 0.072(3) 0.002(2) -0.011(2) -0.0006(19)
C23 0.047(3) 0.034(3) 0.091(5) -0.005(3) -0.020(3) 0.006(2)
C24 0.073(4) 0.041(3) 0.103(6) 0.019(4) -0.045(4) -0.012(3)
C25 0.069(4) 0.054(3) 0.068(4) 0.022(3) -0.011(4) -0.025(3)
C26 0.067(4) 0.041(3) 0.066(3) 0.008(2) -0.004(3) -0.019(3)

_geom_special_details
;
 All esds (except the esd in the dihedral angle between two l.s.
planes)
 are estimated using the full covariance matrix.  The cell esds are
taken
 into account individually in the estimation of esds in distances,
angles
 and torsion angles; correlations between esds in cell parameters are
only
 used when they are defined by crystal symmetry.  An approximate
(isotropic)
 treatment of cell esds is used for estimating esds involving l.s.
planes.
;

loop_
 _geom_bond_atom_site_label_1
 _geom_bond_atom_site_label_2
 _geom_bond_distance
 _geom_bond_site_symmetry_2
 _geom_bond_publ_flag
Ni1 O1 1.924(4) . ?
Ni1 O3 1.985(5) . ?
Ni1 P1 2.1536(14) . ?
Ni1 P2 2.1550(12) . ?
P1 C3 1.806(5) . ?
P1 C9 1.835(5) . ?
P1 C1 1.841(5) . ?
P2 C21 1.809(5) . ?
P2 C15 1.814(5) . ?
P2 C2 1.826(5) . ?
O1 N1 1.238(7) . ?
O2 N1 1.245(9) . ?
```

```
O3 N2 1.123(9) . ?
O4 N2 1.127(9) . ?
C1 C2 1.554(7) . ?
C1 H1A 0.9900 . ?
C1 H1B 0.9900 . ?
C2 H2A 0.9900 . ?
C2 H2B 0.9900 . ?
C3 C4 1.354(9) . ?
C3 C8 1.410(8) . ?
C4 C5 1.435(9) . ?
C4 H4 0.9500 . ?
C5 C6 1.382(12) . ?
C5 H5 0.9500 . ?
C6 C7 1.361(11) . ?
C6 H6 0.9500 . ?
C7 C8 1.400(8) . ?
C7 H7 0.9500 . ?
C8 H8 0.9500 . ?
C9 C10 1.385(7) . ?
C9 C14 1.387(7) . ?
C10 C11 1.369(9) . ?
C10 H10 0.9500 . ?
C11 C12 1.382(10) . ?
C11 H11 0.9500 . ?
C12 C13 1.404(11) . ?
C12 H12 0.9500 . ?
C13 C14 1.379(9) . ?
C13 H13 0.9500 . ?
C14 H14 0.9500 . ?
C15 C20 1.359(8) . ?
C15 C16 1.405(8) . ?
C16 C17 1.385(9) . ?
C16 H16 0.9500 . ?
C17 C18 1.341(14) . ?
C17 H17 0.9500 . ?
C18 C19 1.433(15) . ?
C18 H18 0.9500 . ?
C19 C20 1.418(10) . ?
C19 H19 0.9500 . ?
C20 H20 0.9500 . ?
C21 C22 1.368(8) . ?
C21 C26 1.423(8) . ?
C22 C23 1.402(8) . ?
C22 H22 0.9500 . ?
C23 C24 1.367(11) . ?
C23 H23 0.9500 . ?
C24 C25 1.351(12) . ?
C24 H24 0.9500 . ?
C25 C26 1.403(9) . ?
C25 H25 0.9500 . ?
C26 H26 0.9500 . ?

loop_
 _geom_angle_atom_site_label_1
 _geom_angle_atom_site_label_2
 _geom_angle_atom_site_label_3
 _geom_angle
```

```
 _geom_angle_site_symmetry_1
 _geom_angle_site_symmetry_3
 _geom_angle_publ_flag
O1 Ni1 O3 88.10(19) . . ?
O1 Ni1 P1 97.75(14) . . ?
O3 Ni1 P1 172.44(15) . . ?
O1 Ni1 P2 166.33(17) . . ?
O3 Ni1 P2 86.47(14) . . ?
P1 Ni1 P2 86.77(5) . . ?
C3 P1 C9 109.6(2) . . ?
C3 P1 C1 105.9(2) . . ?
C9 P1 C1 103.5(2) . . ?
C3 P1 Ni1 119.2(2) . . ?
C9 P1 Ni1 111.99(16) . . ?
C1 P1 Ni1 105.13(17) . . ?
C21 P2 C15 107.5(3) . . ?
C21 P2 C2 108.0(2) . . ?
C15 P2 C2 103.8(2) . . ?
C21 P2 Ni1 116.58(17) . . ?
C15 P2 Ni1 109.56(19) . . ?
C2 P2 Ni1 110.70(16) . . ?
N1 O1 Ni1 124.3(4) . . ?
N2 O3 Ni1 109.8(5) . . ?
O1 N1 O2 116.5(6) . . ?
O3 N2 O4 123.6(9) . . ?
C2 C1 P1 106.1(3) . . ?
C2 C1 H1A 110.5 . . ?
P1 C1 H1A 110.5 . . ?
C2 C1 H1B 110.5 . . ?
P1 C1 H1B 110.5 . . ?
H1A C1 H1B 108.7 . . ?
C1 C2 P2 106.8(3) . . ?
C1 C2 H2A 110.4 . . ?
P2 C2 H2A 110.4 . . ?
C1 C2 H2B 110.4 . . ?
P2 C2 H2B 110.4 . . ?
H2A C2 H2B 108.6 . . ?
C4 C3 C8 120.1(5) . . ?
C4 C3 P1 123.4(4) . . ?
C8 C3 P1 116.5(4) . . ?
C3 C4 C5 119.6(7) . . ?
C3 C4 H4 120.2 . . ?
C5 C4 H4 120.2 . . ?
C6 C5 C4 119.0(7) . . ?
C6 C5 H5 120.5 . . ?
C4 C5 H5 120.5 . . ?
C7 C6 C5 121.7(6) . . ?
C7 C6 H6 119.1 . . ?
C5 C6 H6 119.1 . . ?
C6 C7 C8 119.2(7) . . ?
C6 C7 H7 120.4 . . ?
C8 C7 H7 120.4 . . ?
C7 C8 C3 120.3(6) . . ?
C7 C8 H8 119.9 . . ?
C3 C8 H8 119.9 . . ?
C10 C9 C14 120.2(5) . . ?
C10 C9 P1 120.7(4) . . ?
```

```
C14 C9 P1 118.8(4) . . ?
C11 C10 C9 120.4(6) . . ?
C11 C10 H10 119.8 . . ?
C9 C10 H10 119.8 . . ?
C10 C11 C12 119.9(6) . . ?
C10 C11 H11 120.0 . . ?
C12 C11 H11 120.0 . . ?
C11 C12 C13 120.1(6) . . ?
C11 C12 H12 119.9 . . ?
C13 C12 H12 119.9 . . ?
C14 C13 C12 119.4(6) . . ?
C14 C13 H13 120.3 . . ?
C12 C13 H13 120.3 . . ?
C13 C14 C9 119.9(6) . . ?
C13 C14 H14 120.1 . . ?
C9 C14 H14 120.1 . . ?
C20 C15 C16 120.7(5) . . ?
C20 C15 P2 122.0(5) . . ?
C16 C15 P2 117.0(4) . . ?
C17 C16 C15 119.9(7) . . ?
C17 C16 H16 120.0 . . ?
C15 C16 H16 120.0 . . ?
C18 C17 C16 120.5(8) . . ?
C18 C17 H17 119.8 . . ?
C16 C17 H17 119.8 . . ?
C17 C18 C19 121.0(6) . . ?
C17 C18 H18 119.5 . . ?
C19 C18 H18 119.5 . . ?
C20 C19 C18 118.0(7) . . ?
C20 C19 H19 121.0 . . ?
C18 C19 H19 121.0 . . ?
C15 C20 C19 119.8(8) . . ?
C15 C20 H20 120.1 . . ?
C19 C20 H20 120.1 . . ?
C22 C21 C26 120.3(5) . . ?
C22 C21 P2 122.1(4) . . ?
C26 C21 P2 117.5(4) . . ?
C21 C22 C23 119.4(6) . . ?
C21 C22 H22 120.3 . . ?
C23 C22 H22 120.3 . . ?
C24 C23 C22 120.4(6) . . ?
C24 C23 H23 119.8 . . ?
C22 C23 H23 119.8 . . ?
C25 C24 C23 121.0(6) . . ?
C25 C24 H24 119.5 . . ?
C23 C24 H24 119.5 . . ?
C24 C25 C26 120.9(7) . . ?
C24 C25 H25 119.6 . . ?
C26 C25 H25 119.6 . . ?
C25 C26 C21 118.0(7) . . ?
C25 C26 H26 121.0 . . ?
C21 C26 H26 121.0 . . ?

loop_
 _geom_torsion_atom_site_label_1
 _geom_torsion_atom_site_label_2
 _geom_torsion_atom_site_label_3
```

```
 _geom_torsion_atom_site_label_4
 _geom_torsion
 _geom_torsion_site_symmetry_1
 _geom_torsion_site_symmetry_2
 _geom_torsion_site_symmetry_3
 _geom_torsion_site_symmetry_4
 _geom_torsion_publ_flag
O1 Ni1 P1 C3 19.9(3) . . . . ?
O3 Ni1 P1 C3 -120.5(12) . . . . ?
P2 Ni1 P1 C3 -147.1(2) . . . . ?
O1 Ni1 P1 C9 -110.0(2) . . . . ?
O3 Ni1 P1 C9 109.6(12) . . . . ?
P2 Ni1 P1 C9 82.98(18) . . . . ?
O1 Ni1 P1 C1 138.3(2) . . . . ?
O3 Ni1 P1 C1 -2.1(12) . . . . ?
P2 Ni1 P1 C1 -28.70(18) . . . . ?
O1 Ni1 P2 C21 130.9(7) . . . . ?
O3 Ni1 P2 C21 64.1(3) . . . . ?
P1 Ni1 P2 C21 -119.3(2) . . . . ?
O1 Ni1 P2 C15 8.6(7) . . . . ?
O3 Ni1 P2 C15 -58.1(2) . . . . ?
P1 Ni1 P2 C15 118.47(19) . . . . ?
O1 Ni1 P2 C2 -105.2(7) . . . . ?
O3 Ni1 P2 C2 -172.0(2) . . . . ?
P1 Ni1 P2 C2 4.65(18) . . . . ?
O3 Ni1 O1 N1 -136.1(5) . . . . ?
P1 Ni1 O1 N1 48.7(5) . . . . ?
P2 Ni1 O1 N1 157.3(5) . . . . ?
O1 Ni1 O3 N2 84.9(5) . . . . ?
P1 Ni1 O3 N2 -134.3(11) . . . . ?
P2 Ni1 O3 N2 -107.7(5) . . . . ?
Ni1 O1 N1 O2 4.4(8) . . . . ?
Ni1 O3 N2 O4 -0.5(10) . . . . ?
C3 P1 C1 C2 179.0(3) . . . . ?
C9 P1 C1 C2 -65.7(4) . . . . ?
Ni1 P1 C1 C2 51.9(3) . . . . ?
P1 C1 C2 P2 -47.8(4) . . . . ?
C21 P2 C2 C1 154.4(3) . . . . ?
C15 P2 C2 C1 -91.8(4) . . . . ?
Ni1 P2 C2 C1 25.7(4) . . . . ?
C9 P1 C3 C4 -4.7(6) . . . . ?
C1 P1 C3 C4 106.3(5) . . . . ?
Ni1 P1 C3 C4 -135.6(5) . . . . ?
C9 P1 C3 C8 176.2(4) . . . . ?
C1 P1 C3 C8 -72.8(5) . . . . ?
Ni1 P1 C3 C8 45.2(5) . . . . ?
C8 C3 C4 C5 -4.0(10) . . . . ?
P1 C3 C4 C5 176.9(5) . . . . ?
C3 C4 C5 C6 2.8(11) . . . . ?
C4 C5 C6 C7 -1.2(13) . . . . ?
C5 C6 C7 C8 0.7(13) . . . . ?
C6 C7 C8 C3 -1.8(11) . . . . ?
C4 C3 C8 C7 3.6(10) . . . . ?
P1 C3 C8 C7 -177.2(5) . . . . ?
C3 P1 C9 C10 71.9(5) . . . . ?
C1 P1 C9 C10 -40.7(5) . . . . ?
Ni1 P1 C9 C10 -153.4(4) . . . . ?
```

```
C3 P1 C9 C14 -114.9(4) . . . . ?
C1 P1 C9 C14 132.5(4) . . . . ?
Ni1 P1 C9 C14 19.8(4) . . . . ?
C14 C9 C10 C11 -1.6(8) . . . . ?
P1 C9 C10 C11 171.5(4) . . . . ?
C9 C10 C11 C12 3.2(8) . . . . ?
C10 C11 C12 C13 -1.1(9) . . . . ?
C11 C12 C13 C14 -2.5(9) . . . . ?
C12 C13 C14 C9 4.1(9) . . . . ?
C10 C9 C14 C13 -2.0(8) . . . . ?
P1 C9 C14 C13 -175.3(4) . . . . ?
C21 P2 C15 C20 38.5(6) . . . . ?
C2 P2 C15 C20 -75.7(5) . . . . ?
Ni1 P2 C15 C20 166.1(4) . . . . ?
C21 P2 C15 C16 -147.4(4) . . . . ?
C2 P2 C15 C16 98.4(5) . . . . ?
Ni1 P2 C15 C16 -19.9(5) . . . . ?
C20 C15 C16 C17 0.6(9) . . . . ?
P2 C15 C16 C17 -173.5(5) . . . . ?
C15 C16 C17 C18 -1.0(10) . . . . ?
C16 C17 C18 C19 0.1(12) . . . . ?
C17 C18 C19 C20 1.1(11) . . . . ?
C16 C15 C20 C19 0.6(9) . . . . ?
P2 C15 C20 C19 174.4(5) . . . . ?
C18 C19 C20 C15 -1.4(10) . . . . ?
C15 P2 C21 C22 32.4(5) . . . . ?
C2 P2 C21 C22 143.7(4) . . . . ?
Ni1 P2 C21 C22 -91.0(4) . . . . ?
C15 P2 C21 C26 -151.0(4) . . . . ?
C2 P2 C21 C26 -39.7(5) . . . . ?
Ni1 P2 C21 C26 85.6(5) . . . . ?
C26 C21 C22 C23 1.5(8) . . . . ?
P2 C21 C22 C23 178.0(4) . . . . ?
C21 C22 C23 C24 -1.4(9) . . . . ?
C22 C23 C24 C25 0.8(10) . . . . ?
C23 C24 C25 C26 -0.2(11) . . . . ?
C24 C25 C26 C21 0.2(10) . . . . ?
C22 C21 C26 C25 -0.9(9) . . . . ?
P2 C21 C26 C25 -177.6(5) . . . . ?

_diffrn_measured_fraction_theta_max    0.974
_diffrn_reflns_theta_full              29.84
_diffrn_measured_fraction_theta_full   0.974
_refine_diff_density_max    1.924
_refine_diff_density_min   -0.896
_refine_diff_density_rms    0.135


data_bath822

_audit_creation_method            SHELXL-97
_chemical_name_systematic
;
(1,2-Bis(dicyclohexylphosphino)ethane)bis(n1-nitito)nickel(II)
;
_chemical_name_common             ?
_chemical_melting_point           ?
```

```
_chemical_formula_moiety          'C26 H48 N2 Ni O4 P2, C7 H8'
_chemical_formula_sum             'C33 H56 N2 Ni O4 P2'
_chemical_formula_weight          665.45

loop_
 _atom_type_symbol
 _atom_type_description
 _atom_type_scat_dispersion_real
 _atom_type_scat_dispersion_imag
 _atom_type_scat_source
 'C'  'C'   0.0033   0.0016
 'International Tables Vol C Tables 4.2.6.8 and 6.1.1.4'
 'H'  'H'   0.0000   0.0000
 'International Tables Vol C Tables 4.2.6.8 and 6.1.1.4'
 'N'  'N'   0.0061   0.0033
 'International Tables Vol C Tables 4.2.6.8 and 6.1.1.4'
 'O'  'O'   0.0106   0.0060
 'International Tables Vol C Tables 4.2.6.8 and 6.1.1.4'
 'P'  'P'   0.1023   0.0942
 'International Tables Vol C Tables 4.2.6.8 and 6.1.1.4'
 'Ni'  'Ni'   0.3393   1.1124
 'International Tables Vol C Tables 4.2.6.8 and 6.1.1.4'

_symmetry_cell_setting            monoclinic
_symmetry_space_group_name_H-M    P2(1)/c

loop_
 _symmetry_equiv_pos_as_xyz
 'x, y, z'
 '-x, y+1/2, -z+1/2'
 '-x, -y, -z'
 'x, -y-1/2, z-1/2'

_cell_length_a                    12.2125(5)
_cell_length_b                    18.7052(8)
_cell_length_c                    15.1718(6)
_cell_angle_alpha                 90.00
_cell_angle_beta                  97.2610(10)
_cell_angle_gamma                 90.00
_cell_volume                      3438.0(2)
_cell_formula_units_Z             4
_cell_measurement_temperature     100(2)
_cell_measurement_reflns_used     9715
_cell_measurement_theta_min       2.48
_cell_measurement_theta_max       29.32

_exptl_crystal_description        plate
_exptl_crystal_colour             orange
_exptl_crystal_size_max           0.10
_exptl_crystal_size_mid           0.05
_exptl_crystal_size_min           0.02
_exptl_crystal_density_meas       ?
_exptl_crystal_density_diffrn     1.286
_exptl_crystal_density_method     'not measured'
_exptl_crystal_F_000              1432
_exptl_absorpt_coefficient_mu     0.695
_exptl_absorpt_correction_T_min   0.6176
```

```
_exptl_absorpt_correction_T_max   0.7459
_exptl_absorpt_correction_type    'multi-scan'
_exptl_absorpt_process_details    'SADABS - Bruker2004'

_exptl_special_details
;
multi-scan from symmetry-related measurements
 SADABS - Bruker2004
;

_diffrn_ambient_temperature       100(2)
_diffrn_radiation_wavelength      0.68960
_diffrn_radiation_type            Synchrotron
_diffrn_radiation_source          'Station 9.8 Daresbury Laboratory'
_diffrn_radiation_monochromator   'Si (111)'
_diffrn_measurement_device_type   'Bruker APEX2'
_diffrn_measurement_method        '1800 0.36 degree images with \w
scans'
_diffrn_detector_area_resol_mean  ?
_diffrn_standards_number          ?
_diffrn_standards_interval_count  ?
_diffrn_standards_interval_time   ?
_diffrn_standards_decay_%         0
_diffrn_reflns_number             37994
_diffrn_reflns_av_R_equivalents   0.0474
_diffrn_reflns_av_sigmaI/netI     0.0441
_diffrn_reflns_limit_h_min        -17
_diffrn_reflns_limit_h_max        17
_diffrn_reflns_limit_k_min        -26
_diffrn_reflns_limit_k_max        26
_diffrn_reflns_limit_l_min        -20
_diffrn_reflns_limit_l_max        21
_diffrn_reflns_theta_min          1.70
_diffrn_reflns_theta_max          29.70
_reflns_number_total              10185
_reflns_number_gt                 8442
_reflns_threshold_expression      >2sigma(I)

_computing_data_collection      'Bruker SMART'
_computing_cell_refinement      'Bruker SMART'
_computing_data_reduction       'Bruker SAINT'
_computing_structure_solution   'SHELXTS (Sheldrick, 1997)'
_computing_structure_refinement 'SHELXTL (Sheldrick, 1997)'
_computing_molecular_graphics   'ORTEP'
_computing_publication_material 'SHELXTL (Sheldrick, 1997)'

_refine_special_details
;
 Refinement of F^2^ against ALL reflections.  The weighted R-factor wR
and
 goodness of fit S are based on F^2^, conventional R-factors R are
based
 on F, with F set to zero for negative F^2^. The threshold expression
of
 F^2^ > 2sigma(F^2^) is used only for calculating R-factors(gt) etc.
and is
```

```
 not relevant to the choice of reflections for refinement.  R-factors
based
 on F^2^ are statistically about twice as large as those based on F,
and R-
 factors based on ALL data will be even larger.
;

_refine_ls_structure_factor_coef  Fsqd
_refine_ls_matrix_type            full
_refine_ls_weighting_scheme       calc
_refine_ls_weighting_details
 'calc w=1/[\s^2^(Fo^2^)+(0.0427P)^2^+1.4823P] where
P=(Fo^2^+2Fc^2^)/3'
_atom_sites_solution_primary      direct
_atom_sites_solution_secondary    difmap
_atom_sites_solution_hydrogens    geom
_refine_ls_hydrogen_treatment     constr
_refine_ls_extinction_method      none
_refine_ls_extinction_coef        ?
_refine_ls_number_reflns          10185
_refine_ls_number_parameters      380
_refine_ls_number_restraints      0
_refine_ls_R_factor_all           0.0497
_refine_ls_R_factor_gt            0.0394
_refine_ls_wR_factor_ref          0.0992
_refine_ls_wR_factor_gt           0.0933
_refine_ls_goodness_of_fit_ref    1.043
_refine_ls_restrained_S_all       1.043
_refine_ls_shift/su_max           0.002
_refine_ls_shift/su_mean          0.000

loop_
 _atom_site_label
 _atom_site_type_symbol
 _atom_site_fract_x
 _atom_site_fract_y
 _atom_site_fract_z
 _atom_site_U_iso_or_equiv
 _atom_site_adp_type
 _atom_site_occupancy
 _atom_site_symmetry_multiplicity
 _atom_site_calc_flag
 _atom_site_refinement_flags
 _atom_site_disorder_assembly
 _atom_site_disorder_group
Ni1 Ni 0.853675(15) 0.261550(10) 0.762928(12) 0.01336(6) Uani 1 1 d .
. .
P1 P 0.98200(3) 0.20144(2) 0.84601(2) 0.01283(8) Uani 1 1 d . . .
P2 P 0.73778(3) 0.23358(2) 0.85590(2) 0.01261(8) Uani 1 1 d . . .
N1 N 0.95224(13) 0.27609(11) 0.67669(10) 0.0316(4) Uani 1 1 d . . .
O1 O 0.93837(14) 0.22870(8) 0.61552(10) 0.0413(4) Uani 1 1 d . . .
O2 O 1.01541(15) 0.31856(9) 0.67701(10) 0.0453(4) Uani 1 1 d . . .
N2 N 0.74643(13) 0.31829(9) 0.69164(10) 0.0262(3) Uani 1 1 d . . .
O3 O 0.67805(12) 0.29181(10) 0.63544(10) 0.0444(4) Uani 1 1 d . . .
O4 O 0.75268(15) 0.38441(8) 0.70131(11) 0.0476(4) Uani 1 1 d . . .
C1 C 0.92857(12) 0.17459(8) 0.94868(10) 0.0154(3) Uani 1 1 d . . .
H1A H 0.9616 0.1281 0.9689 0.018 Uiso 1 1 calc R . .
```

H1B H 0.9510 0.2104 0.9955 0.018 Uiso 1 1 calc R . .
C2 C 0.80244(12) 0.16776(8) 0.93610(10) 0.0146(3) Uani 1 1 d . . .
H2A H 0.7746 0.1748 0.9940 0.018 Uiso 1 1 calc R . .
H2B H 0.7816 0.1190 0.9149 0.018 Uiso 1 1 calc R . .
C3 C 1.10847(12) 0.25282(8) 0.88153(10) 0.0146(3) Uani 1 1 d . . .
H3 H 1.1461 0.2613 0.8276 0.018 Uiso 1 1 calc R . .
C4 C 1.08156(13) 0.32647(8) 0.91860(11) 0.0173(3) Uani 1 1 d . . .
H4A H 1.0330 0.3532 0.8729 0.021 Uiso 1 1 calc R . .
H4B H 1.0415 0.3200 0.9708 0.021 Uiso 1 1 calc R . .
C5 C 1.18728(14) 0.36944(9) 0.94576(12) 0.0214(3) Uani 1 1 d . . .
H5A H 1.2229 0.3805 0.8922 0.026 Uiso 1 1 calc R . .
H5B H 1.1682 0.4153 0.9727 0.026 Uiso 1 1 calc R . .
C6 C 1.26826(14) 0.32816(9) 1.01212(12) 0.0223(3) Uani 1 1 d . . .
H6A H 1.2355 0.3210 1.0679 0.027 Uiso 1 1 calc R . .
H6B H 1.3370 0.3562 1.0262 0.027 Uiso 1 1 calc R . .
C7 C 1.29529(13) 0.25556(9) 0.97379(12) 0.0209(3) Uani 1 1 d . . .
H7A H 1.3462 0.2290 1.0182 0.025 Uiso 1 1 calc R . .
H7B H 1.3328 0.2628 0.9203 0.025 Uiso 1 1 calc R . .
C8 C 1.18998(13) 0.21180(8) 0.94904(11) 0.0176(3) Uani 1 1 d . . .
H8A H 1.1550 0.2018 1.0031 0.021 Uiso 1 1 calc R . .
H8B H 1.2088 0.1655 0.9231 0.021 Uiso 1 1 calc R . .
C9 C 1.02800(12) 0.11760(8) 0.79730(10) 0.0156(3) Uani 1 1 d . . .
H9 H 1.0632 0.0876 0.8476 0.019 Uiso 1 1 calc R . .
C10 C 1.11419(13) 0.12801(9) 0.73285(11) 0.0205(3) Uani 1 1 d . . .
H10A H 1.0841 0.1604 0.6841 0.025 Uiso 1 1 calc R . .
H10B H 1.1812 0.1505 0.7647 0.025 Uiso 1 1 calc R . .
C11 C 1.14479(14) 0.05607(9) 0.69388(12) 0.0225(3) Uani 1 1 d . . .
H11A H 1.1977 0.0642 0.6506 0.027 Uiso 1 1 calc R . .
H11B H 1.1813 0.0254 0.7421 0.027 Uiso 1 1 calc R . .
C12 C 1.04293(14) 0.01790(9) 0.64792(12) 0.0246(3) Uani 1 1 d . . .
H12A H 1.0647 -0.0285 0.6241 0.029 Uiso 1 1 calc R . .
H12B H 1.0085 0.0472 0.5976 0.029 Uiso 1 1 calc R . .
C13 C 0.95984(14) 0.00539(9) 0.71377(12) 0.0236(3) Uani 1 1 d . . .
H13A H 0.8933 -0.0184 0.6831 0.028 Uiso 1 1 calc R . .
H13B H 0.9928 -0.0264 0.7621 0.028 Uiso 1 1 calc R . .
C14 C 0.92713(13) 0.07616(8) 0.75307(11) 0.0173(3) Uani 1 1 d . . .
H14A H 0.8763 0.0667 0.7977 0.021 Uiso 1 1 calc R . .
H14B H 0.8873 0.1058 0.7053 0.021 Uiso 1 1 calc R . .
C15 C 0.61209(12) 0.18973(8) 0.80298(10) 0.0155(3) Uani 1 1 d . . .
H15 H 0.5693 0.2266 0.7653 0.019 Uiso 1 1 calc R . .
C16 C 0.63824(13) 0.12921(9) 0.74050(11) 0.0217(3) Uani 1 1 d . . .
H16A H 0.6841 0.1480 0.6964 0.026 Uiso 1 1 calc R . .
H16B H 0.6808 0.0914 0.7752 0.026 Uiso 1 1 calc R . .
C17 C 0.53170(15) 0.09754(11) 0.69242(13) 0.0289(4) Uani 1 1 d . . .
H17A H 0.5498 0.0571 0.6547 0.035 Uiso 1 1 calc R . .
H17B H 0.4927 0.1343 0.6533 0.035 Uiso 1 1 calc R . .
C18 C 0.45610(15) 0.07131(10) 0.75871(14) 0.0300(4) Uani 1 1 d . . .
H18A H 0.3865 0.0529 0.7260 0.036 Uiso 1 1 calc R . .
H18B H 0.4924 0.0316 0.7944 0.036 Uiso 1 1 calc R . .
C19 C 0.43035(13) 0.13173(10) 0.82025(12) 0.0244(3) Uani 1 1 d . . .
H19A H 0.3832 0.1134 0.8637 0.029 Uiso 1 1 calc R . .
H19B H 0.3890 0.1698 0.7850 0.029 Uiso 1 1 calc R . .
C20 C 0.53679(13) 0.16297(9) 0.86986(11) 0.0188(3) Uani 1 1 d . . .
H20A H 0.5757 0.1259 0.9085 0.023 Uiso 1 1 calc R . .
H20B H 0.5186 0.2031 0.9080 0.023 Uiso 1 1 calc R . .
C21 C 0.69015(12) 0.30760(8) 0.92171(10) 0.0148(3) Uani 1 1 d . . .
H21 H 0.6647 0.2865 0.9762 0.018 Uiso 1 1 calc R . .

```
C22 C 0.59306(13) 0.34966(9) 0.87221(11) 0.0190(3) Uani 1 1 d . . .
H22A H 0.6156 0.3708 0.8174 0.023 Uiso 1 1 calc R . .
H22B H 0.5305 0.3168 0.8548 0.023 Uiso 1 1 calc R . .
C23 C 0.55663(14) 0.40904(9) 0.93124(12) 0.0218(3) Uani 1 1 d . . .
H23A H 0.5290 0.3876 0.9838 0.026 Uiso 1 1 calc R . .
H23B H 0.4954 0.4363 0.8979 0.026 Uiso 1 1 calc R . .
C24 C 0.65194(14) 0.45981(9) 0.96185(12) 0.0214(3) Uani 1 1 d . . .
H24A H 0.6747 0.4850 0.9098 0.026 Uiso 1 1 calc R . .
H24B H 0.6271 0.4961 1.0025 0.026 Uiso 1 1 calc R . .
C25 C 0.75018(14) 0.41885(9) 1.00947(11) 0.0215(3) Uani 1 1 d . . .
H25A H 0.7298 0.3986 1.0655 0.026 Uiso 1 1 calc R . .
H25B H 0.8126 0.4522 1.0248 0.026 Uiso 1 1 calc R . .
C26 C 0.78649(13) 0.35841(8) 0.95193(11) 0.0193(3) Uani 1 1 d . . .
H26A H 0.8471 0.3312 0.9862 0.023 Uiso 1 1 calc R . .
H26B H 0.8149 0.3789 0.8991 0.023 Uiso 1 1 calc R . .
C31 C 0.67455(16) -0.07945(11) 0.89216(13) 0.0297(4) Uani 1 1 d . . .
C32 C 0.78644(16) -0.06982(10) 0.88681(13) 0.0298(4) Uani 1 1 d . . .
H32 H 0.8186 -0.0910 0.8393 0.036 Uiso 1 1 calc R . .
C33 C 0.85159(16) -0.02976(11) 0.94981(14) 0.0314(4) Uani 1 1 d . . .
H33 H 0.9279 -0.0237 0.9451 0.038 Uiso 1 1 calc R . .
C34 C 0.80666(17) 0.00142(10) 1.01946(13) 0.0311(4) Uani 1 1 d . . .
H34 H 0.8519 0.0285 1.0628 0.037 Uiso 1 1 calc R . .
C35 C 0.69557(17) -0.00691(10) 1.02575(13) 0.0299(4) Uani 1 1 d . . .
H35 H 0.6639 0.0150 1.0731 0.036 Uiso 1 1 calc R . .
C36 C 0.62995(16) -0.04747(11) 0.96267(13) 0.0304(4) Uani 1 1 d . . .
H36 H 0.5537 -0.0535 0.9677 0.037 Uiso 1 1 calc R . .
C37 C 0.6033(2) -0.12282(14) 0.82368(16) 0.0464(6) Uani 1 1 d . . .
H37A H 0.5319 -0.1321 0.8445 0.070 Uiso 1 1 calc R . .
H37B H 0.6399 -0.1683 0.8143 0.070 Uiso 1 1 calc R . .
H37C H 0.5916 -0.0962 0.7676 0.070 Uiso 1 1 calc R . .

loop_
 _atom_site_aniso_label
 _atom_site_aniso_U_11
 _atom_site_aniso_U_22
 _atom_site_aniso_U_33
 _atom_site_aniso_U_23
 _atom_site_aniso_U_13
 _atom_site_aniso_U_12
Ni1 0.01281(10) 0.01610(10) 0.01148(9) 0.00174(7) 0.00269(7)
0.00089(7)
P1 0.01186(16) 0.01410(18) 0.01269(17) -0.00103(13) 0.00218(13)
0.00045(13)
P2 0.01197(17) 0.01297(17) 0.01317(17) 0.00037(13) 0.00264(13)
0.00094(13)
N1 0.0248(8) 0.0580(12) 0.0130(7) 0.0077(7) 0.0054(6) 0.0217(8)
O1 0.0604(10) 0.0402(9) 0.0254(7) 0.0039(6) 0.0138(7) 0.0120(7)
O2 0.0619(11) 0.0465(9) 0.0269(8) 0.0125(7) 0.0032(7) -0.0040(8)
N2 0.0249(7) 0.0352(9) 0.0203(7) 0.0108(6) 0.0100(6) 0.0073(6)
O3 0.0292(8) 0.0730(12) 0.0290(8) 0.0156(8) -0.0044(6) -0.0045(8)
O4 0.0688(11) 0.0293(8) 0.0482(10) 0.0183(7) 0.0211(8) 0.0187(8)
C1 0.0162(7) 0.0168(7) 0.0132(6) 0.0012(5) 0.0021(5) 0.0022(5)
C2 0.0151(7) 0.0148(7) 0.0146(7) 0.0026(5) 0.0043(5) 0.0015(5)
C3 0.0126(6) 0.0151(7) 0.0160(7) -0.0006(5) 0.0015(5) -0.0004(5)
C4 0.0164(7) 0.0131(7) 0.0220(7) -0.0009(6) 0.0009(6) 0.0002(5)
C5 0.0208(8) 0.0153(7) 0.0277(8) -0.0005(6) 0.0008(6) -0.0014(6)
C6 0.0197(8) 0.0201(8) 0.0259(8) -0.0038(6) -0.0026(6) -0.0031(6)
```

```
C7 0.0151(7) 0.0212(8) 0.0252(8) -0.0006(6) -0.0020(6) 0.0019(6)
C8 0.0166(7) 0.0139(7) 0.0215(7) -0.0004(6) -0.0008(6) 0.0019(5)
C9 0.0140(7) 0.0154(7) 0.0176(7) -0.0031(5) 0.0027(5) 0.0012(5)
C10 0.0158(7) 0.0215(8) 0.0257(8) -0.0064(6) 0.0084(6) -0.0015(6)
C11 0.0196(8) 0.0230(8) 0.0263(8) -0.0050(6) 0.0084(6) 0.0041(6)
C12 0.0254(8) 0.0213(8) 0.0284(9) -0.0102(7) 0.0092(7) 0.0002(6)
C13 0.0240(8) 0.0178(8) 0.0302(9) -0.0078(6) 0.0084(7) -0.0030(6)
C14 0.0154(7) 0.0163(7) 0.0208(7) -0.0044(6) 0.0051(6) -0.0007(5)
C15 0.0127(6) 0.0175(7) 0.0167(7) -0.0018(5) 0.0033(5) -0.0005(5)
C16 0.0169(7) 0.0259(8) 0.0229(8) -0.0090(6) 0.0048(6) -0.0019(6)
C17 0.0223(8) 0.0356(10) 0.0287(9) -0.0154(8) 0.0023(7) -0.0059(7)
C18 0.0215(8) 0.0301(10) 0.0380(10) -0.0100(8) 0.0029(7) -0.0097(7)
C19 0.0152(7) 0.0286(9) 0.0297(9) -0.0023(7) 0.0046(6) -0.0035(6)
C20 0.0155(7) 0.0219(8) 0.0198(7) -0.0010(6) 0.0057(6) -0.0020(6)
C21 0.0161(7) 0.0131(7) 0.0157(7) -0.0007(5) 0.0044(5) 0.0017(5)
C22 0.0167(7) 0.0188(7) 0.0210(8) -0.0018(6) 0.0011(6) 0.0040(6)
C23 0.0190(7) 0.0199(8) 0.0271(8) -0.0015(6) 0.0048(6) 0.0057(6)
C24 0.0255(8) 0.0134(7) 0.0264(8) 0.0003(6) 0.0074(7) 0.0032(6)
C25 0.0232(8) 0.0175(7) 0.0233(8) -0.0053(6) 0.0015(6) -0.0004(6)
C26 0.0165(7) 0.0170(7) 0.0242(8) -0.0039(6) 0.0020(6) 0.0010(6)
C31 0.0317(10) 0.0298(10) 0.0259(9) 0.0063(7) -0.0027(7) 0.0010(8)
C32 0.0326(10) 0.0290(9) 0.0289(9) 0.0077(7) 0.0082(8) 0.0076(8)
C33 0.0229(9) 0.0297(10) 0.0410(11) 0.0157(8) 0.0018(8) 0.0013(7)
C34 0.0354(10) 0.0222(9) 0.0331(10) 0.0109(7) -0.0054(8) -0.0033(7)
C35 0.0366(10) 0.0272(9) 0.0264(9) 0.0048(7) 0.0061(8) 0.0041(8)
C36 0.0249(9) 0.0344(10) 0.0322(10) 0.0068(8) 0.0042(7) 0.0019(7)
C37 0.0519(14) 0.0480(14) 0.0358(12) -0.0038(10) -0.0078(10) -
0.0062(11)

_geom_special_details
;
 All esds (except the esd in the dihedral angle between two l.s.
planes)
 are estimated using the full covariance matrix.  The cell esds are
taken
 into account individually in the estimation of esds in distances,
angles
 and torsion angles; correlations between esds in cell parameters are
only
 used when they are defined by crystal symmetry.  An approximate
(isotropic)
 treatment of cell esds is used for estimating esds involving l.s.
planes.
;

loop_
 _geom_bond_atom_site_label_1
 _geom_bond_atom_site_label_2
 _geom_bond_distance
 _geom_bond_site_symmetry_2
 _geom_bond_publ_flag
Ni1 N1 1.9060(16) . ?
Ni1 N2 1.9114(15) . ?
Ni1 P2 2.1843(4) . ?
Ni1 P1 2.1931(4) . ?
P1 C1 1.8335(15) . ?
P1 C3 1.8410(15) . ?
```

P1 C9 1.8510(15) . ?
P2 C15 1.8336(15) . ?
P2 C2 1.8376(15) . ?
P2 C21 1.8437(15) . ?
N1 O2 1.107(2) . ?
N1 O1 1.279(2) . ?
N2 O3 1.221(2) . ?
N2 O4 1.247(2) . ?
C1 C2 1.533(2) . ?
C1 H1A 0.9900 . ?
C1 H1B 0.9900 . ?
C2 H2A 0.9900 . ?
C2 H2B 0.9900 . ?
C3 C4 1.539(2) . ?
C3 C8 1.540(2) . ?
C3 H3 1.0000 . ?
C4 C5 1.532(2) . ?
C4 H4A 0.9900 . ?
C4 H4B 0.9900 . ?
C5 C6 1.529(2) . ?
C5 H5A 0.9900 . ?
C5 H5B 0.9900 . ?
C6 C7 1.530(2) . ?
C6 H6A 0.9900 . ?
C6 H6B 0.9900 . ?
C7 C8 1.531(2) . ?
C7 H7A 0.9900 . ?
C7 H7B 0.9900 . ?
C8 H8A 0.9900 . ?
C8 H8B 0.9900 . ?
C9 C14 1.536(2) . ?
C9 C10 1.537(2) . ?
C9 H9 1.0000 . ?
C10 C11 1.535(2) . ?
C10 H10A 0.9900 . ?
C10 H10B 0.9900 . ?
C11 C12 1.525(2) . ?
C11 H11A 0.9900 . ?
C11 H11B 0.9900 . ?
C12 C13 1.529(2) . ?
C12 H12A 0.9900 . ?
C12 H12B 0.9900 . ?
C13 C14 1.526(2) . ?
C13 H13A 0.9900 . ?
C13 H13B 0.9900 . ?
C14 H14A 0.9900 . ?
C14 H14B 0.9900 . ?
C15 C16 1.536(2) . ?
C15 C20 1.537(2) . ?
C15 H15 1.0000 . ?
C16 C17 1.528(2) . ?
C16 H16A 0.9900 . ?
C16 H16B 0.9900 . ?
C17 C18 1.530(3) . ?
C17 H17A 0.9900 . ?
C17 H17B 0.9900 . ?
C18 C19 1.524(3) . ?

```
C18 H18A 0.9900 . ?
C18 H18B 0.9900 . ?
C19 C20 1.533(2) . ?
C19 H19A 0.9900 . ?
C19 H19B 0.9900 . ?
C20 H20A 0.9900 . ?
C20 H20B 0.9900 . ?
C21 C26 1.537(2) . ?
C21 C22 1.537(2) . ?
C21 H21 1.0000 . ?
C22 C23 1.528(2) . ?
C22 H22A 0.9900 . ?
C22 H22B 0.9900 . ?
C23 C24 1.528(2) . ?
C23 H23A 0.9900 . ?
C23 H23B 0.9900 . ?
C24 C25 1.526(2) . ?
C24 H24A 0.9900 . ?
C24 H24B 0.9900 . ?
C25 C26 1.528(2) . ?
C25 H25A 0.9900 . ?
C25 H25B 0.9900 . ?
C26 H26A 0.9900 . ?
C26 H26B 0.9900 . ?
C31 C32 1.391(3) . ?
C31 C36 1.395(3) . ?
C31 C37 1.506(3) . ?
C32 C33 1.384(3) . ?
C32 H32 0.9500 . ?
C33 C34 1.380(3) . ?
C33 H33 0.9500 . ?
C34 C35 1.381(3) . ?
C34 H34 0.9500 . ?
C35 C36 1.393(3) . ?
C35 H35 0.9500 . ?
C36 H36 0.9500 . ?
C37 H37A 0.9800 . ?
C37 H37B 0.9800 . ?
C37 H37C 0.9800 . ?

loop_
 _geom_angle_atom_site_label_1
 _geom_angle_atom_site_label_2
 _geom_angle_atom_site_label_3
 _geom_angle
 _geom_angle_site_symmetry_1
 _geom_angle_site_symmetry_3
 _geom_angle_publ_flag
N1 Ni1 N2 88.82(6) . . ?
N1 Ni1 P2 173.95(6) . . ?
N2 Ni1 P2 92.57(4) . . ?
N1 Ni1 P1 90.02(5) . . ?
N2 Ni1 P1 177.03(5) . . ?
P2 Ni1 P1 88.874(16) . . ?
C1 P1 C3 105.65(7) . . ?
C1 P1 C9 105.41(7) . . ?
C3 P1 C9 105.87(7) . . ?
```

C1 P1 Ni1 108.88(5) . . ?
C3 P1 Ni1 114.38(5) . . ?
C9 P1 Ni1 115.83(5) . . ?
C15 P2 C2 104.84(7) . . ?
C15 P2 C21 105.61(7) . . ?
C2 P2 C21 106.45(7) . . ?
C15 P2 Ni1 113.54(5) . . ?
C2 P2 Ni1 109.20(5) . . ?
C21 P2 Ni1 116.38(5) . . ?
O2 N1 O1 121.94(17) . . ?
O2 N1 Ni1 126.84(16) . . ?
O1 N1 Ni1 111.22(15) . . ?
O3 N2 O4 120.74(17) . . ?
O3 N2 Ni1 121.96(14) . . ?
O4 N2 Ni1 117.19(14) . . ?
C2 C1 P1 112.14(10) . . ?
C2 C1 H1A 109.2 . . ?
P1 C1 H1A 109.2 . . ?
C2 C1 H1B 109.2 . . ?
P1 C1 H1B 109.2 . . ?
H1A C1 H1B 107.9 . . ?
C1 C2 P2 111.56(10) . . ?
C1 C2 H2A 109.3 . . ?
P2 C2 H2A 109.3 . . ?
C1 C2 H2B 109.3 . . ?
P2 C2 H2B 109.3 . . ?
H2A C2 H2B 108.0 . . ?
C4 C3 C8 110.67(12) . . ?
C4 C3 P1 111.36(10) . . ?
C8 C3 P1 112.49(10) . . ?
C4 C3 H3 107.4 . . ?
C8 C3 H3 107.4 . . ?
P1 C3 H3 107.4 . . ?
C5 C4 C3 110.87(13) . . ?
C5 C4 H4A 109.5 . . ?
C3 C4 H4A 109.5 . . ?
C5 C4 H4B 109.5 . . ?
C3 C4 H4B 109.5 . . ?
H4A C4 H4B 108.1 . . ?
C6 C5 C4 111.42(13) . . ?
C6 C5 H5A 109.3 . . ?
C4 C5 H5A 109.3 . . ?
C6 C5 H5B 109.3 . . ?
C4 C5 H5B 109.3 . . ?
H5A C5 H5B 108.0 . . ?
C5 C6 C7 110.54(13) . . ?
C5 C6 H6A 109.5 . . ?
C7 C6 H6A 109.5 . . ?
C5 C6 H6B 109.5 . . ?
C7 C6 H6B 109.5 . . ?
H6A C6 H6B 108.1 . . ?
C6 C7 C8 110.68(13) . . ?
C6 C7 H7A 109.5 . . ?
C8 C7 H7A 109.5 . . ?
C6 C7 H7B 109.5 . . ?
C8 C7 H7B 109.5 . . ?
H7A C7 H7B 108.1 . . ?

C7 C8 C3 110.44(13) . . ?
C7 C8 H8A 109.6 . . ?
C3 C8 H8A 109.6 . . ?
C7 C8 H8B 109.6 . . ?
C3 C8 H8B 109.6 . . ?
H8A C8 H8B 108.1 . . ?
C14 C9 C10 111.22(13) . . ?
C14 C9 P1 109.56(10) . . ?
C10 C9 P1 114.43(11) . . ?
C14 C9 H9 107.1 . . ?
C10 C9 H9 107.1 . . ?
P1 C9 H9 107.1 . . ?
C11 C10 C9 110.72(13) . . ?
C11 C10 H10A 109.5 . . ?
C9 C10 H10A 109.5 . . ?
C11 C10 H10B 109.5 . . ?
C9 C10 H10B 109.5 . . ?
H10A C10 H10B 108.1 . . ?
C12 C11 C10 111.25(13) . . ?
C12 C11 H11A 109.4 . . ?
C10 C11 H11A 109.4 . . ?
C12 C11 H11B 109.4 . . ?
C10 C11 H11B 109.4 . . ?
H11A C11 H11B 108.0 . . ?
C11 C12 C13 110.08(14) . . ?
C11 C12 H12A 109.6 . . ?
C13 C12 H12A 109.6 . . ?
C11 C12 H12B 109.6 . . ?
C13 C12 H12B 109.6 . . ?
H12A C12 H12B 108.2 . . ?
C14 C13 C12 110.53(14) . . ?
C14 C13 H13A 109.5 . . ?
C12 C13 H13A 109.5 . . ?
C14 C13 H13B 109.5 . . ?
C12 C13 H13B 109.5 . . ?
H13A C13 H13B 108.1 . . ?
C13 C14 C9 111.93(13) . . ?
C13 C14 H14A 109.2 . . ?
C9 C14 H14A 109.2 . . ?
C13 C14 H14B 109.2 . . ?
C9 C14 H14B 109.2 . . ?
H14A C14 H14B 107.9 . . ?
C16 C15 C20 110.85(13) . . ?
C16 C15 P2 111.84(10) . . ?
C20 C15 P2 113.22(11) . . ?
C16 C15 H15 106.8 . . ?
C20 C15 H15 106.8 . . ?
P2 C15 H15 106.8 . . ?
C17 C16 C15 110.45(13) . . ?
C17 C16 H16A 109.6 . . ?
C15 C16 H16A 109.6 . . ?
C17 C16 H16B 109.6 . . ?
C15 C16 H16B 109.6 . . ?
H16A C16 H16B 108.1 . . ?
C16 C17 C18 111.02(15) . . ?
C16 C17 H17A 109.4 . . ?
C18 C17 H17A 109.4 . . ?

C16 C17 H17B 109.4 . . ?
C18 C17 H17B 109.4 . . ?
H17A C17 H17B 108.0 . . ?
C19 C18 C17 110.69(15) . . ?
C19 C18 H18A 109.5 . . ?
C17 C18 H18A 109.5 . . ?
C19 C18 H18B 109.5 . . ?
C17 C18 H18B 109.5 . . ?
H18A C18 H18B 108.1 . . ?
C18 C19 C20 110.83(14) . . ?
C18 C19 H19A 109.5 . . ?
C20 C19 H19A 109.5 . . ?
C18 C19 H19B 109.5 . . ?
C20 C19 H19B 109.5 . . ?
H19A C19 H19B 108.1 . . ?
C19 C20 C15 109.94(13) . . ?
C19 C20 H20A 109.7 . . ?
C15 C20 H20A 109.7 . . ?
C19 C20 H20B 109.7 . . ?
C15 C20 H20B 109.7 . . ?
H20A C20 H20B 108.2 . . ?
C26 C21 C22 110.09(12) . . ?
C26 C21 P2 110.12(10) . . ?
C22 C21 P2 113.35(11) . . ?
C26 C21 H21 107.7 . . ?
C22 C21 H21 107.7 . . ?
P2 C21 H21 107.7 . . ?
C23 C22 C21 110.45(13) . . ?
C23 C22 H22A 109.6 . . ?
C21 C22 H22A 109.6 . . ?
C23 C22 H22B 109.6 . . ?
C21 C22 H22B 109.6 . . ?
H22A C22 H22B 108.1 . . ?
C22 C23 C24 111.27(13) . . ?
C22 C23 H23A 109.4 . . ?
C24 C23 H23A 109.4 . . ?
C22 C23 H23B 109.4 . . ?
C24 C23 H23B 109.4 . . ?
H23A C23 H23B 108.0 . . ?
C25 C24 C23 110.78(13) . . ?
C25 C24 H24A 109.5 . . ?
C23 C24 H24A 109.5 . . ?
C25 C24 H24B 109.5 . . ?
C23 C24 H24B 109.5 . . ?
H24A C24 H24B 108.1 . . ?
C24 C25 C26 111.63(14) . . ?
C24 C25 H25A 109.3 . . ?
C26 C25 H25A 109.3 . . ?
C24 C25 H25B 109.3 . . ?
C26 C25 H25B 109.3 . . ?
H25A C25 H25B 108.0 . . ?
C25 C26 C21 111.19(13) . . ?
C25 C26 H26A 109.4 . . ?
C21 C26 H26A 109.4 . . ?
C25 C26 H26B 109.4 . . ?
C21 C26 H26B 109.4 . . ?
H26A C26 H26B 108.0 . . ?

```
C32 C31 C36 118.09(18) . . ?
C32 C31 C37 121.1(2) . . ?
C36 C31 C37 120.83(19) . . ?
C33 C32 C31 120.87(18) . . ?
C33 C32 H32 119.6 . . ?
C31 C32 H32 119.6 . . ?
C34 C33 C32 120.54(18) . . ?
C34 C33 H33 119.7 . . ?
C32 C33 H33 119.7 . . ?
C33 C34 C35 119.63(19) . . ?
C33 C34 H34 120.2 . . ?
C35 C34 H34 120.2 . . ?
C34 C35 C36 119.93(19) . . ?
C34 C35 H35 120.0 . . ?
C36 C35 H35 120.0 . . ?
C35 C36 C31 120.93(18) . . ?
C35 C36 H36 119.5 . . ?
C31 C36 H36 119.5 . . ?
C31 C37 H37A 109.5 . . ?
C31 C37 H37B 109.5 . . ?
H37A C37 H37B 109.5 . . ?
C31 C37 H37C 109.5 . . ?
H37A C37 H37C 109.5 . . ?
H37B C37 H37C 109.5 . . ?

loop_
 _geom_torsion_atom_site_label_1
 _geom_torsion_atom_site_label_2
 _geom_torsion_atom_site_label_3
 _geom_torsion_atom_site_label_4
 _geom_torsion
 _geom_torsion_site_symmetry_1
 _geom_torsion_site_symmetry_2
 _geom_torsion_site_symmetry_3
 _geom_torsion_site_symmetry_4
 _geom_torsion_publ_flag
N1 Ni1 P1 C1 178.09(8) . . . . ?
N2 Ni1 P1 C1 111.2(9) . . . . ?
P2 Ni1 P1 C1 -7.86(6) . . . . ?
N1 Ni1 P1 C3 60.18(8) . . . . ?
N2 Ni1 P1 C3 -6.7(9) . . . . ?
P2 Ni1 P1 C3 -125.76(6) . . . . ?
N1 Ni1 P1 C9 -63.39(8) . . . . ?
N2 Ni1 P1 C9 -130.3(9) . . . . ?
P2 Ni1 P1 C9 110.67(6) . . . . ?
N1 Ni1 P2 C15 -45.7(4) . . . . ?
N2 Ni1 P2 C15 57.42(8) . . . . ?
P1 Ni1 P2 C15 -125.18(6) . . . . ?
N1 Ni1 P2 C2 70.9(4) . . . . ?
N2 Ni1 P2 C2 174.01(7) . . . . ?
P1 Ni1 P2 C2 -8.60(5) . . . . ?
N1 Ni1 P2 C21 -168.6(4) . . . . ?
N2 Ni1 P2 C21 -65.51(8) . . . . ?
P1 Ni1 P2 C21 111.89(6) . . . . ?
N2 Ni1 N1 O2 93.13(18) . . . . ?
P2 Ni1 N1 O2 -163.5(3) . . . . ?
P1 Ni1 N1 O2 -84.14(17) . . . . ?
```

N2 Ni1 N1 O1 -87.75(13) . . . . ?
P2 Ni1 N1 O1 15.6(5) . . . . ?
P1 Ni1 N1 O1 94.99(12) . . . . ?
N1 Ni1 N2 O3 91.68(15) . . . . ?
P2 Ni1 N2 O3 -82.43(14) . . . . ?
P1 Ni1 N2 O3 158.6(8) . . . . ?
N1 Ni1 N2 O4 -84.46(14) . . . . ?
P2 Ni1 N2 O4 101.44(13) . . . . ?
P1 Ni1 N2 O4 -17.5(10) . . . . ?
C3 P1 C1 C2 149.77(11) . . . . ?
C9 P1 C1 C2 -98.40(11) . . . . ?
Ni1 P1 C1 C2 26.49(12) . . . . ?
P1 C1 C2 P2 -33.87(13) . . . . ?
C15 P2 C2 C1 148.91(10) . . . . ?
C21 P2 C2 C1 -99.47(11) . . . . ?
Ni1 P2 C2 C1 26.93(11) . . . . ?
C1 P1 C3 C4 -71.88(12) . . . . ?
C9 P1 C3 C4 176.62(11) . . . . ?
Ni1 P1 C3 C4 47.85(12) . . . . ?
C1 P1 C3 C8 53.01(12) . . . . ?
C9 P1 C3 C8 -58.49(12) . . . . ?
Ni1 P1 C3 C8 172.74(9) . . . . ?
C8 C3 C4 C5 55.51(17) . . . . ?
P1 C3 C4 C5 -178.58(11) . . . . ?
C3 C4 C5 C6 -55.58(18) . . . . ?
C4 C5 C6 C7 56.51(18) . . . . ?
C5 C6 C7 C8 -57.64(18) . . . . ?
C6 C7 C8 C3 57.95(18) . . . . ?
C4 C3 C8 C7 -56.81(17) . . . . ?
P1 C3 C8 C7 177.92(11) . . . . ?
C1 P1 C9 C14 76.76(12) . . . . ?
C3 P1 C9 C14 -171.56(11) . . . . ?
Ni1 P1 C9 C14 -43.65(12) . . . . ?
C1 P1 C9 C10 -157.55(11) . . . . ?
C3 P1 C9 C10 -45.88(13) . . . . ?
Ni1 P1 C9 C10 82.03(12) . . . . ?
C14 C9 C10 C11 -53.57(18) . . . . ?
P1 C9 C10 C11 -178.38(11) . . . . ?
C9 C10 C11 C12 56.48(19) . . . . ?
C10 C11 C12 C13 -58.61(19) . . . . ?
C11 C12 C13 C14 57.95(19) . . . . ?
C12 C13 C14 C9 -56.10(19) . . . . ?
C10 C9 C14 C13 54.01(18) . . . . ?
P1 C9 C14 C13 -178.49(11) . . . . ?
C2 P2 C15 C16 -71.42(12) . . . . ?
C21 P2 C15 C16 176.36(11) . . . . ?
Ni1 P2 C15 C16 47.68(12) . . . . ?
C2 P2 C15 C20 54.68(12) . . . . ?
C21 P2 C15 C20 -57.54(12) . . . . ?
Ni1 P2 C15 C20 173.79(9) . . . . ?
C20 C15 C16 C17 56.77(18) . . . . ?
P2 C15 C16 C17 -175.84(12) . . . . ?
C15 C16 C17 C18 -56.4(2) . . . . ?
C16 C17 C18 C19 56.9(2) . . . . ?
C17 C18 C19 C20 -57.5(2) . . . . ?
C18 C19 C20 C15 57.57(19) . . . . ?
C16 C15 C20 C19 -57.21(17) . . . . ?

```
P2 C15 C20 C19 176.16(11) . . . . ?
C15 P2 C21 C26 -167.18(11) . . . . ?
C2 P2 C21 C26 81.74(12) . . . . ?
Ni1 P2 C21 C26 -40.21(12) . . . . ?
C15 P2 C21 C22 -43.35(13) . . . . ?
C2 P2 C21 C22 -154.44(11) . . . . ?
Ni1 P2 C21 C22 83.62(11) . . . . ?
C26 C21 C22 C23 -57.27(17) . . . . ?
P2 C21 C22 C23 178.89(11) . . . . ?
C21 C22 C23 C24 57.59(18) . . . . ?
C22 C23 C24 C25 -56.10(18) . . . . ?
C23 C24 C25 C26 54.94(18) . . . . ?
C24 C25 C26 C21 -55.53(18) . . . . ?
C22 C21 C26 C25 56.34(17) . . . . ?
P2 C21 C26 C25 -177.96(11) . . . . ?
C36 C31 C32 C33 0.1(3) . . . . ?
C37 C31 C32 C33 179.85(18) . . . . ?
C31 C32 C33 C34 0.1(3) . . . . ?
C32 C33 C34 C35 -0.6(3) . . . . ?
C33 C34 C35 C36 0.9(3) . . . . ?
C34 C35 C36 C31 -0.7(3) . . . . ?
C32 C31 C36 C35 0.2(3) . . . . ?
C37 C31 C36 C35 -179.54(19) . . . . ?

_diffrn_measured_fraction_theta_max    0.974
_diffrn_reflns_theta_full              29.70
_diffrn_measured_fraction_theta_full   0.974
_refine_diff_density_max    1.156
_refine_diff_density_min   -0.686
_refine_diff_density_rms    0.069


data_bath824

_audit_creation_method            SHELXL-97
_chemical_name_systematic
;
(1,2-Bis(dicyclohexylphosphino)ethane)bis(n1-nitrito)nickel(II)
;
_chemical_name_common             ?
_chemical_melting_point           ?
_chemical_formula_moiety          'C26 H48 N2 Ni O4 P2, C7 H8'
_chemical_formula_sum             'C33 H56 N2 Ni O4 P2'
_chemical_formula_weight          665.45

loop_
 _atom_type_symbol
 _atom_type_description
 _atom_type_scat_dispersion_real
 _atom_type_scat_dispersion_imag
 _atom_type_scat_source
 'C'  'C'   0.0033   0.0016
 'International Tables Vol C Tables 4.2.6.8 and 6.1.1.4'
 'H'  'H'   0.0000   0.0000
 'International Tables Vol C Tables 4.2.6.8 and 6.1.1.4'
 'N'  'N'   0.0061   0.0033
 'International Tables Vol C Tables 4.2.6.8 and 6.1.1.4'
```

```
 'O'  'O'   0.0106   0.0060
 'International Tables Vol C Tables 4.2.6.8 and 6.1.1.4'
 'P'  'P'   0.1023   0.0942
 'International Tables Vol C Tables 4.2.6.8 and 6.1.1.4'
 'Ni'  'Ni'   0.3393   1.1124
 'International Tables Vol C Tables 4.2.6.8 and 6.1.1.4'

_symmetry_cell_setting            monoclinic
_symmetry_space_group_name_H-M    P2(1)/c

loop_
 _symmetry_equiv_pos_as_xyz
 'x, y, z'
 '-x, y+1/2, -z+1/2'
 '-x, -y, -z'
 'x, -y-1/2, z-1/2'

_cell_length_a                    12.199(10)
_cell_length_b                    19.146(8)
_cell_length_c                    15.093(10)
_cell_angle_alpha                 90.00
_cell_angle_beta                  101.00(5)
_cell_angle_gamma                 90.00
_cell_volume                      3461(4)
_cell_formula_units_Z             4
_cell_measurement_temperature     100(2)
_cell_measurement_reflns_used     9715
_cell_measurement_theta_min       2.48
_cell_measurement_theta_max       29.32

_exptl_crystal_description        plate
_exptl_crystal_colour             orange
_exptl_crystal_size_max           0.10
_exptl_crystal_size_mid           0.05
_exptl_crystal_size_min           0.02
_exptl_crystal_density_meas       ?
_exptl_crystal_density_diffrn     1.277
_exptl_crystal_density_method     'not measured'
_exptl_crystal_F_000              1432
_exptl_absorpt_coefficient_mu     0.690
_exptl_absorpt_correction_T_min   0.6099
_exptl_absorpt_correction_T_max   0.7459
_exptl_absorpt_correction_type    'multi-scan'
_exptl_absorpt_process_details    'SADABS - Bruker2004'

_exptl_special_details
;
multi-scan from symmetry-related measurements
 SADABS - Bruker2004
;

_diffrn_ambient_temperature       100(2)
_diffrn_radiation_wavelength      0.68960
_diffrn_radiation_type            Synchrotron
_diffrn_radiation_source          'Station 9.8 Daresbury Laboratory'
_diffrn_radiation_monochromator   'Si (111)'
_diffrn_measurement_device_type   'Bruker APEX2'
```

```
_diffrn_measurement_method        '1800 0.36 degree images with \w
scans'
_diffrn_detector_area_resol_mean  ?
_diffrn_standards_number          ?
_diffrn_standards_interval_count  ?
_diffrn_standards_interval_time   ?
_diffrn_standards_decay_%         0
_diffrn_reflns_number             38030
_diffrn_reflns_av_R_equivalents   0.0437
_diffrn_reflns_av_sigmaI/netI     0.0429
_diffrn_reflns_limit_h_min        -17
_diffrn_reflns_limit_h_max        17
_diffrn_reflns_limit_k_min        -27
_diffrn_reflns_limit_k_max        27
_diffrn_reflns_limit_l_min        -20
_diffrn_reflns_limit_l_max        21
_diffrn_reflns_theta_min          2.19
_diffrn_reflns_theta_max          29.73
_reflns_number_total              10272
_reflns_number_gt                 7698
_reflns_threshold_expression      >2sigma(I)

_computing_data_collection      'Bruker SMART'
_computing_cell_refinement      'Bruker SMART'
_computing_data_reduction       'Bruker SAINT'
_computing_structure_solution   'SHELXTS (Sheldrick, 1997)'
_computing_structure_refinement 'SHELXTL (Sheldrick, 1997)'
_computing_molecular_graphics   'ORTEP'
_computing_publication_material 'SHELXTL (Sheldrick, 1997)'

_refine_special_details
;
 Refinement of F^2^ against ALL reflections.  The weighted R-factor wR
and
 goodness of fit S are based on F^2^, conventional R-factors R are
based
 on F, with F set to zero for negative F^2^. The threshold expression
of
 F^2^ > 2sigma(F^2^) is used only for calculating R-factors(gt) etc.
and is
 not relevant to the choice of reflections for refinement.  R-factors
based
 on F^2^ are statistically about twice as large as those based on F,
and R-
 factors based on ALL data will be even larger.
;

_refine_ls_structure_factor_coef  Fsqd
_refine_ls_matrix_type            full
_refine_ls_weighting_scheme       calc
_refine_ls_weighting_details
 'calc w=1/[\s^2^(Fo^2^)+(0.1410P)^2^+8.9713P] where
P=(Fo^2^+2Fc^2^)/3'
_atom_sites_solution_primary      direct
_atom_sites_solution_secondary    difmap
_atom_sites_solution_hydrogens    geom
_refine_ls_hydrogen_treatment     constr
```

```
_refine_ls_extinction_method      none
_refine_ls_extinction_coef        ?
_refine_ls_number_reflns          10272
_refine_ls_number_parameters      460
_refine_ls_number_restraints      0
_refine_ls_R_factor_all           0.1177
_refine_ls_R_factor_gt            0.0946
_refine_ls_wR_factor_ref          0.2704
_refine_ls_wR_factor_gt           0.2501
_refine_ls_goodness_of_fit_ref    1.046
_refine_ls_restrained_S_all       1.046
_refine_ls_shift/su_max           0.000
_refine_ls_shift/su_mean          0.000

loop_
 _atom_site_label
 _atom_site_type_symbol
 _atom_site_fract_x
 _atom_site_fract_y
 _atom_site_fract_z
 _atom_site_U_iso_or_equiv
 _atom_site_adp_type
 _atom_site_occupancy
 _atom_site_symmetry_multiplicity
 _atom_site_calc_flag
 _atom_site_refinement_flags
 _atom_site_disorder_assembly
 _atom_site_disorder_group
Ni1 Ni 0.84799(4) 0.26253(3) 0.75646(3) 0.03106(16) Uani 1 1 d . . .
P1 P 0.98002(8) 0.20328(6) 0.83836(7) 0.0336(2) Uani 1 1 d . A .
P2 P 0.74248(8) 0.24167(5) 0.85465(6) 0.0272(2) Uani 1 1 d . A .
N1 N 0.9885(4) 0.3146(2) 0.6619(3) 0.0587(13) Uani 1 1 d . . .
O1 O 0.9299(3) 0.26320(18) 0.6593(2) 0.0460(8) Uani 1 1 d . A .
O2 O 1.0044(6) 0.3557(3) 0.7165(3) 0.0967(19) Uani 1 1 d . A .
N2 N 0.6660(5) 0.3122(4) 0.6303(4) 0.0502(15) Uani 0.758(9) 1 d P A 1
O3 O 0.7408(4) 0.3324(3) 0.6991(3) 0.0460(13) Uani 0.758(9) 1 d P A 1
O4 O 0.6664(5) 0.2503(3) 0.6144(3) 0.0558(15) Uani 0.758(9) 1 d P A 1
N2A N 0.7133(17) 0.3537(12) 0.6460(14) 0.051(6) Uani 0.242(9) 1 d P A
2
O3A O 0.7837(14) 0.3889(8) 0.7000(12) 0.057(5) Uani 0.242(9) 1 d P A 2
O4A O 0.7231(9) 0.2865(7) 0.6650(7) 0.029(3) Uani 0.242(9) 1 d P A 2
C1 C 0.9445(7) 0.1876(7) 0.9505(6) 0.024(3) Uani 0.44(2) 1 d P A 1
H1A H 0.9742 0.1415 0.9733 0.029 Uiso 0.44(2) 1 calc PR A 1
H1B H 0.9807 0.2237 0.9933 0.029 Uiso 0.44(2) 1 calc PR A 1
C1A C 0.9171(6) 0.1555(5) 0.9262(5) 0.025(2) Uani 0.56(2) 1 d P A 2
H1A1 H 0.8965 0.1077 0.9041 0.030 Uiso 0.56(2) 1 calc PR A 2
H1A2 H 0.9743 0.1513 0.9821 0.030 Uiso 0.56(2) 1 calc PR A 2
C2 C 0.8206(3) 0.1892(3) 0.9471(3) 0.0454(11) Uani 1 1 d . . .
H2A H 0.8064 0.2080 1.0050 0.054 Uiso 1 1 calc R A 1
H2B H 0.7918 0.1407 0.9411 0.054 Uiso 1 1 calc R A 1
C3 C 1.1065(4) 0.2538(2) 0.8826(3) 0.0390(9) Uani 1 1 d . . .
H3 H 1.1396 0.2680 0.8295 0.047 Uiso 1 1 calc R A .
C4 C 1.0788(5) 0.3217(3) 0.9291(3) 0.0605(16) Uani 1 1 d . A .
H4A H 1.0220 0.3488 0.8873 0.073 Uiso 1 1 calc R . .
H4B H 1.0480 0.3102 0.9833 0.073 Uiso 1 1 calc R . .
C5 C 1.1852(6) 0.3652(3) 0.9560(4) 0.072(2) Uani 1 1 d . . .
H5A H 1.1679 0.4084 0.9866 0.086 Uiso 1 1 calc R A .
```

H5B H 1.2134 0.3788 0.9012 0.086 Uiso 1 1 calc R . .
C6 C 1.2759(5) 0.3238(3) 1.0195(4) 0.0620(16) Uani 1 1 d . A .
H6A H 1.3444 0.3525 1.0351 0.074 Uiso 1 1 calc R . .
H6B H 1.2497 0.3125 1.0759 0.074 Uiso 1 1 calc R . .
C7 C 1.3018(4) 0.2564(3) 0.9732(3) 0.0501(12) Uani 1 1 d . . .
H7A H 1.3324 0.2680 0.9188 0.060 Uiso 1 1 calc R A .
H7B H 1.3591 0.2295 1.0148 0.060 Uiso 1 1 calc R . .
C8 C 1.1966(4) 0.2121(3) 0.9463(3) 0.0398(9) Uani 1 1 d . A .
H8A H 1.1681 0.1984 1.0010 0.048 Uiso 1 1 calc R . .
H8B H 1.2146 0.1691 0.9157 0.048 Uiso 1 1 calc R . .
C9 C 1.0299(3) 0.1248(2) 0.7883(3) 0.0327(8) Uani 1 1 d . . .
H9 H 1.0699 0.0954 0.8391 0.039 Uiso 1 1 calc R A .
C10 C 1.1115(3) 0.1384(2) 0.7246(3) 0.0332(8) Uani 1 1 d . A .
H10A H 1.0743 0.1668 0.6727 0.040 Uiso 1 1 calc R . .
H10B H 1.1766 0.1650 0.7569 0.040 Uiso 1 1 calc R . .
C11 C 1.1509(4) 0.0692(3) 0.6906(3) 0.0452(10) Uani 1 1 d . . .
H11A H 1.2036 0.0786 0.6495 0.054 Uiso 1 1 calc R A .
H11B H 1.1909 0.0417 0.7423 0.054 Uiso 1 1 calc R . .
C12 C 1.0523(5) 0.0275(3) 0.6408(5) 0.0685(18) Uani 1 1 d . A .
H12A H 1.0150 0.0539 0.5870 0.082 Uiso 1 1 calc R . .
H12B H 1.0790 -0.0173 0.6201 0.082 Uiso 1 1 calc R . .
C13 C 0.9702(5) 0.0134(3) 0.7011(6) 0.078(2) Uani 1 1 d . . .
H13A H 1.0056 -0.0164 0.7521 0.093 Uiso 1 1 calc R A .
H13B H 0.9052 -0.0122 0.6668 0.093 Uiso 1 1 calc R . .
C14 C 0.9306(4) 0.0810(2) 0.7374(5) 0.0560(14) Uani 1 1 d . A .
H14A H 0.8791 0.0700 0.7789 0.067 Uiso 1 1 calc R . .
H14B H 0.8888 0.1088 0.6867 0.067 Uiso 1 1 calc R . .
C15 C 0.6188(3) 0.1911(2) 0.8054(3) 0.0298(7) Uani 1 1 d . . .
H15 H 0.5735 0.2211 0.7579 0.036 Uiso 1 1 calc R A .
C16 C 0.6504(3) 0.1256(2) 0.7577(4) 0.0450(11) Uani 1 1 d . A .
H16A H 0.6985 0.1389 0.7145 0.054 Uiso 1 1 calc R . .
H16B H 0.6933 0.0934 0.8027 0.054 Uiso 1 1 calc R . .
C17 C 0.5461(4) 0.0887(3) 0.7074(4) 0.0596(15) Uani 1 1 d . . .
H17A H 0.5678 0.0457 0.6786 0.072 Uiso 1 1 calc R A .
H17B H 0.5060 0.1196 0.6593 0.072 Uiso 1 1 calc R . .
C18 C 0.4688(4) 0.0697(3) 0.7730(4) 0.0511(12) Uani 1 1 d . A .
H18A H 0.4001 0.0474 0.7393 0.061 Uiso 1 1 calc R . .
H18B H 0.5068 0.0358 0.8181 0.061 Uiso 1 1 calc R . .
C19 C 0.4377(3) 0.1344(2) 0.8210(3) 0.0415(9) Uani 1 1 d . . .
H19A H 0.3897 0.1209 0.8641 0.050 Uiso 1 1 calc R A .
H19B H 0.3948 0.1668 0.7763 0.050 Uiso 1 1 calc R . .
C20 C 0.5425(3) 0.1713(2) 0.8716(3) 0.0345(8) Uani 1 1 d . A .
H20A H 0.5831 0.1400 0.9190 0.041 Uiso 1 1 calc R . .
H20B H 0.5209 0.2139 0.9013 0.041 Uiso 1 1 calc R . .
C21 C 0.6886(3) 0.3150(2) 0.9126(2) 0.0303(7) Uani 1 1 d . . .
H21 H 0.6594 0.2946 0.9645 0.036 Uiso 1 1 calc R A .
C22 C 0.5911(4) 0.3542(2) 0.8542(3) 0.0403(9) Uani 1 1 d . A .
H22A H 0.6166 0.3763 0.8025 0.048 Uiso 1 1 calc R . .
H22B H 0.5306 0.3209 0.8303 0.048 Uiso 1 1 calc R . .
C23 C 0.5465(4) 0.4103(2) 0.9105(3) 0.0454(10) Uani 1 1 d . . .
H23A H 0.4846 0.4359 0.8720 0.055 Uiso 1 1 calc R A .
H23B H 0.5166 0.3878 0.9600 0.055 Uiso 1 1 calc R . .
C24 C 0.6383(5) 0.4616(2) 0.9502(3) 0.0476(11) Uani 1 1 d . A .
H24A H 0.6637 0.4872 0.9008 0.057 Uiso 1 1 calc R . .
H24B H 0.6086 0.4960 0.9884 0.057 Uiso 1 1 calc R . .
C25 C 0.7364(4) 0.4231(3) 1.0063(3) 0.0467(10) Uani 1 1 d . . .
H25A H 0.7125 0.4019 1.0594 0.056 Uiso 1 1 calc R A .

```
H25B H 0.7967 0.4569 1.0287 0.056 Uiso 1 1 calc R . .
C26 C 0.7819(4) 0.3659(3) 0.9527(3) 0.0429(10) Uani 1 1 d . A .
H26A H 0.8422 0.3402 0.9927 0.051 Uiso 1 1 calc R . .
H26B H 0.8137 0.3872 0.9035 0.051 Uiso 1 1 calc R . .
C31 C 0.7190(8) -0.0639(5) 0.9089(5) 0.068(4) Uani 0.521(13) 1 d PG B
1
C32 C 0.8301(8) -0.0430(5) 0.9277(6) 0.064(4) Uani 0.521(13) 1 d PG B
1
H32 H 0.8798 -0.0580 0.8901 0.077 Uiso 0.521(13) 1 calc PR B 1
C33 C 0.8687(6) -0.0004(5) 1.0016(7) 0.066(4) Uani 0.521(13) 1 d PG B
1
H33 H 0.9447 0.0139 1.0144 0.079 Uiso 0.521(13) 1 calc PR B 1
C34 C 0.7961(8) 0.0215(4) 1.0566(5) 0.067(4) Uani 0.521(13) 1 d PG B 1
H34 H 0.8224 0.0507 1.1071 0.081 Uiso 0.521(13) 1 calc PR B 1
C35 C 0.6849(8) 0.0007(6) 1.0378(6) 0.063(4) Uani 0.521(13) 1 d PG B 1
H35 H 0.6352 0.0156 1.0755 0.076 Uiso 0.521(13) 1 calc PR B 1
C36 C 0.6463(6) -0.0420(6) 0.9640(6) 0.070(5) Uani 0.521(13) 1 d PG B
1
H36 H 0.5703 -0.0562 0.9511 0.083 Uiso 0.521(13) 1 calc PR B 1
C37 C 0.679(2) -0.1097(11) 0.8295(10) 0.105(7) Uani 0.521(13) 1 d P B
1
H37A H 0.5993 -0.1190 0.8247 0.158 Uiso 0.521(13) 1 calc PR B 1
H37B H 0.7205 -0.1539 0.8370 0.158 Uiso 0.521(13) 1 calc PR B 1
H37C H 0.6916 -0.0863 0.7745 0.158 Uiso 0.521(13) 1 calc PR B 1
C31B C 0.7150(7) -0.0916(6) 0.8728(7) 0.060(4) Uani 0.479(13) 1 d PG B
3
C32B C 0.8066(6) -0.0519(6) 0.9124(7) 0.062(5) Uani 0.479(13) 1 d PG B
3
H32B H 0.8730 -0.0526 0.8881 0.074 Uiso 0.479(13) 1 calc PR B 3
C33B C 0.8013(9) -0.0110(5) 0.9876(7) 0.065(4) Uani 0.479(13) 1 d PG B
3
H33B H 0.8639 0.0162 1.0147 0.078 Uiso 0.479(13) 1 calc PR B 3
C34B C 0.7042(11) -0.0099(6) 1.0232(7) 0.087(9) Uani 0.479(13) 1 d PG
B 3
H34B H 0.7005 0.0180 1.0745 0.104 Uiso 0.479(13) 1 calc PR B 3
C35B C 0.6126(8) -0.0497(8) 0.9835(9) 0.117(12) Uani 0.479(13) 1 d PG
B 3
H35B H 0.5463 -0.0489 1.0079 0.141 Uiso 0.479(13) 1 calc PR B 3
C36B C 0.6180(6) -0.0905(6) 0.9084(8) 0.078(6) Uani 0.479(13) 1 d PG B
3
H36B H 0.5553 -0.1177 0.8813 0.094 Uiso 0.479(13) 1 calc PR B 3
C37B C 0.7247(18) -0.1365(13) 0.7911(17) 0.113(8) Uani 0.479(13) 1 d P
B 3
H37D H 0.6544 -0.1616 0.7705 0.169 Uiso 0.479(13) 1 calc PR B 3
H37E H 0.7855 -0.1703 0.8079 0.169 Uiso 0.479(13) 1 calc PR B 3
H37F H 0.7404 -0.1066 0.7423 0.169 Uiso 0.479(13) 1 calc PR B 3

loop_
 _atom_site_aniso_label
 _atom_site_aniso_U_11
 _atom_site_aniso_U_22
 _atom_site_aniso_U_33
 _atom_site_aniso_U_23
 _atom_site_aniso_U_13
 _atom_site_aniso_U_12
Ni1 0.0307(3) 0.0363(3) 0.0244(2) 0.00473(18) 0.00091(18) 0.00413(18)
P1 0.0318(5) 0.0430(6) 0.0276(4) 0.0109(4) 0.0099(4) 0.0176(4)
```

P2 0.0247(4) 0.0308(5) 0.0248(4) 0.0061(3) 0.0013(3) -0.0013(3)
N1 0.075(3) 0.036(2) 0.046(2) 0.0051(17) -0.037(2) -0.0118(19)
O1 0.055(2) 0.0517(19) 0.0314(15) 0.0051(13) 0.0099(14) 0.0000(15)
O2 0.140(5) 0.065(3) 0.071(3) 0.016(2) -0.015(3) -0.037(3)
N2 0.039(3) 0.077(5) 0.034(3) 0.010(3) 0.006(2) 0.009(3)
O3 0.049(2) 0.051(3) 0.037(2) 0.012(2) 0.0063(18) 0.012(2)
O4 0.067(3) 0.072(4) 0.029(2) -0.002(2) 0.009(2) -0.002(3)
N2A 0.042(10) 0.066(13) 0.054(11) 0.040(10) 0.028(9) 0.031(10)
O3A 0.058(9) 0.037(8) 0.076(11) 0.003(7) 0.016(8) -0.001(6)
O4A 0.027(5) 0.044(8) 0.018(5) 0.011(5) 0.008(4) 0.006(5)
C1 0.023(4) 0.031(6) 0.019(4) 0.001(3) 0.004(3) -0.001(3)
C1A 0.020(3) 0.032(4) 0.024(3) -0.001(3) 0.005(2) 0.001(3)
C2 0.0244(17) 0.071(3) 0.041(2) 0.029(2) 0.0070(15) 0.0125(18)
C3 0.044(2) 0.044(2) 0.0246(16) -0.0034(15) -0.0036(15) 0.0213(18)
C4 0.066(3) 0.062(3) 0.042(2) -0.021(2) -0.018(2) 0.042(3)
C5 0.088(4) 0.053(3) 0.056(3) -0.023(3) -0.030(3) 0.032(3)
C6 0.064(3) 0.060(3) 0.050(3) -0.019(2) -0.021(2) 0.020(3)
C7 0.047(3) 0.055(3) 0.041(2) -0.006(2) -0.0094(19) 0.017(2)
C8 0.039(2) 0.048(2) 0.0297(18) 0.0021(16) -0.0010(15) 0.0217(18)
C9 0.0250(16) 0.0285(17) 0.046(2) 0.0074(15) 0.0107(15) 0.0050(13)
C10 0.0277(17) 0.037(2) 0.0364(19) -0.0045(15) 0.0096(14) 0.0027(14)
C11 0.035(2) 0.048(2) 0.052(3) -0.011(2) 0.0060(18) 0.0145(18)
C12 0.048(3) 0.048(3) 0.100(5) -0.036(3) -0.009(3) 0.016(2)
C13 0.043(3) 0.030(2) 0.151(7) -0.010(3) -0.006(3) -0.004(2)
C14 0.0277(19) 0.032(2) 0.109(5) 0.005(2) 0.014(2) -0.0025(16)
C15 0.0215(15) 0.0298(17) 0.0367(18) 0.0002(14) 0.0024(13) 0.0023(12)
C16 0.0274(18) 0.037(2) 0.073(3) -0.013(2) 0.0153(19) 0.0000(15)
C17 0.044(3) 0.058(3) 0.082(4) -0.037(3) 0.025(3) -0.014(2)
C18 0.030(2) 0.047(3) 0.078(3) -0.018(2) 0.012(2) -0.0109(18)
C19 0.0216(16) 0.044(2) 0.058(3) -0.0073(19) 0.0062(16) -0.0040(15)
C20 0.0228(16) 0.038(2) 0.042(2) -0.0022(16) 0.0058(14) -0.0031(14)
C21 0.0298(17) 0.0312(17) 0.0285(16) 0.0017(13) 0.0023(13) -0.0027(13)
C22 0.045(2) 0.0291(19) 0.041(2) 0.0013(16) -0.0069(17) 0.0050(16)
C23 0.049(2) 0.030(2) 0.053(3) -0.0024(18) -0.001(2) 0.0042(17)
C24 0.065(3) 0.030(2) 0.047(2) -0.0021(18) 0.010(2) -0.0080(19)
C25 0.051(3) 0.043(2) 0.044(2) -0.0065(19) 0.004(2) -0.011(2)
C26 0.041(2) 0.048(2) 0.039(2) -0.0094(18) 0.0075(17) -0.0166(18)
C31 0.096(10) 0.072(9) 0.030(5) 0.012(5) -0.002(6) 0.039(8)
C32 0.075(8) 0.063(9) 0.066(8) 0.039(7) 0.041(7) 0.035(7)
C33 0.047(6) 0.044(6) 0.103(10) 0.035(6) 0.006(6) 0.000(5)
C34 0.085(10) 0.046(6) 0.068(8) 0.025(5) 0.007(7) 0.006(6)
C35 0.052(8) 0.078(9) 0.066(8) 0.028(6) 0.029(6) 0.029(7)
C36 0.047(7) 0.111(12) 0.049(7) 0.035(8) 0.006(6) 0.034(7)
C37 0.16(2) 0.102(14) 0.044(8) 0.009(8) -0.017(9) 0.028(13)
C31B 0.038(6) 0.085(11) 0.055(9) 0.024(8) 0.006(6) 0.022(6)
C32B 0.036(6) 0.075(11) 0.080(10) 0.051(9) 0.027(6) 0.019(6)
C33B 0.075(9) 0.044(6) 0.081(9) 0.035(6) 0.027(8) 0.014(6)
C34B 0.092(14) 0.108(15) 0.082(12) 0.057(11) 0.066(12) 0.067(13)
C35B 0.097(14) 0.15(2) 0.130(19) 0.101(17) 0.087(14) 0.077(15)
C36B 0.037(6) 0.091(11) 0.107(13) 0.057(10) 0.016(7) 0.014(6)
C37B 0.084(13) 0.123(18) 0.112(17) -0.014(14) -0.028(12) 0.028(12)

_geom_special_details
;
 All esds (except the esd in the dihedral angle between two l.s.
planes)

```
 are estimated using the full covariance matrix.  The cell esds are
taken
 into account individually in the estimation of esds in distances,
angles
 and torsion angles; correlations between esds in cell parameters are
only
 used when they are defined by crystal symmetry.  An approximate
(isotropic)
 treatment of cell esds is used for estimating esds involving l.s.
planes.
;

loop_
 _geom_bond_atom_site_label_1
 _geom_bond_atom_site_label_2
 _geom_bond_distance
 _geom_bond_site_symmetry_2
 _geom_bond_publ_flag
Ni1 O4A 1.906(11) . ?
Ni1 O1 1.926(4) . ?
Ni1 O3 1.953(5) . ?
Ni1 P1 2.1563(16) . ?
Ni1 P2 2.1774(17) . ?
P1 C3 1.835(5) . ?
P1 C9 1.837(4) . ?
P1 C1 1.850(8) . ?
P1 C1A 1.889(7) . ?
P2 C15 1.827(4) . ?
P2 C2 1.832(4) . ?
P2 C21 1.841(4) . ?
N1 O2 1.129(7) . ?
N1 O1 1.213(5) . ?
N2 O4 1.209(9) . ?
N2 O3 1.304(9) . ?
N2A O3A 1.26(3) . ?
N2A O4A 1.32(3) . ?
C1 C2 1.503(9) . ?
C1 H1A 0.9900 . ?
C1 H1B 0.9900 . ?
C1A C2 1.430(7) . ?
C1A H1A1 0.9900 . ?
C1A H1A2 0.9900 . ?
C2 H2A 0.9900 . ?
C2 H2B 0.9900 . ?
C3 C8 1.538(5) . ?
C3 C4 1.545(6) . ?
C3 H3 1.0000 . ?
C4 C5 1.531(9) . ?
C4 H4A 0.9900 . ?
C4 H4B 0.9900 . ?
C5 C6 1.538(7) . ?
C5 H5A 0.9900 . ?
C5 H5B 0.9900 . ?
C6 C7 1.528(7) . ?
C6 H6A 0.9900 . ?
C6 H6B 0.9900 . ?
C7 C8 1.528(7) . ?
```

C7 H7A 0.9900 . ?
C7 H7B 0.9900 . ?
C8 H8A 0.9900 . ?
C8 H8B 0.9900 . ?
C9 C10 1.533(5) . ?
C9 C14 1.551(6) . ?
C9 H9 1.0000 . ?
C10 C11 1.532(6) . ?
C10 H10A 0.9900 . ?
C10 H10B 0.9900 . ?
C11 C12 1.518(7) . ?
C11 H11A 0.9900 . ?
C11 H11B 0.9900 . ?
C12 C13 1.500(11) . ?
C12 H12A 0.9900 . ?
C12 H12B 0.9900 . ?
C13 C14 1.520(8) . ?
C13 H13A 0.9900 . ?
C13 H13B 0.9900 . ?
C14 H14A 0.9900 . ?
C14 H14B 0.9900 . ?
C15 C16 1.531(6) . ?
C15 C20 1.538(5) . ?
C15 H15 1.0000 . ?
C16 C17 1.526(7) . ?
C16 H16A 0.9900 . ?
C16 H16B 0.9900 . ?
C17 C18 1.536(8) . ?
C17 H17A 0.9900 . ?
C17 H17B 0.9900 . ?
C18 C19 1.518(7) . ?
C18 H18A 0.9900 . ?
C18 H18B 0.9900 . ?
C19 C20 1.531(5) . ?
C19 H19A 0.9900 . ?
C19 H19B 0.9900 . ?
C20 H20A 0.9900 . ?
C20 H20B 0.9900 . ?
C21 C26 1.532(5) . ?
C21 C22 1.534(5) . ?
C21 H21 1.0000 . ?
C22 C23 1.532(6) . ?
C22 H22A 0.9900 . ?
C22 H22B 0.9900 . ?
C23 C24 1.523(7) . ?
C23 H23A 0.9900 . ?
C23 H23B 0.9900 . ?
C24 C25 1.519(7) . ?
C24 H24A 0.9900 . ?
C24 H24B 0.9900 . ?
C25 C26 1.528(7) . ?
C25 H25A 0.9900 . ?
C25 H25B 0.9900 . ?
C26 H26A 0.9900 . ?
C26 H26B 0.9900 . ?
C31 C32 1.3900 . ?
C31 C36 1.3900 . ?

```
C31 C37 1.490(19) . ?
C32 C33 1.3900 . ?
C32 H32 0.9500 . ?
C33 C34 1.3900 . ?
C33 H33 0.9500 . ?
C34 C35 1.3900 . ?
C34 H34 0.9500 . ?
C35 C36 1.3900 . ?
C35 H35 0.9500 . ?
C36 H36 0.9500 . ?
C37 H37A 0.9800 . ?
C37 H37B 0.9800 . ?
C37 H37C 0.9800 . ?
C31B C32B 1.3900 . ?
C31B C36B 1.3900 . ?
C31B C37B 1.53(2) . ?
C32B C33B 1.3900 . ?
C32B H32B 0.9500 . ?
C33B C34B 1.3900 . ?
C33B H33B 0.9500 . ?
C34B C35B 1.3900 . ?
C34B H34B 0.9500 . ?
C35B C36B 1.3900 . ?
C35B H35B 0.9500 . ?
C36B H36B 0.9500 . ?
C37B H37D 0.9800 . ?
C37B H37E 0.9800 . ?
C37B H37F 0.9800 . ?

loop_
 _geom_angle_atom_site_label_1
 _geom_angle_atom_site_label_2
 _geom_angle_atom_site_label_3
 _geom_angle
 _geom_angle_site_symmetry_1
 _geom_angle_site_symmetry_3
 _geom_angle_publ_flag
O4A Ni1 O1 84.6(3) . . ?
O4A Ni1 O3 30.6(4) . . ?
O1 Ni1 O3 93.38(18) . . ?
O4A Ni1 P1 161.4(4) . . ?
O1 Ni1 P1 90.04(12) . . ?
O3 Ni1 P1 167.92(17) . . ?
O4A Ni1 P2 92.4(3) . . ?
O1 Ni1 P2 168.41(11) . . ?
O3 Ni1 P2 89.68(15) . . ?
P1 Ni1 P2 89.26(7) . . ?
C3 P1 C9 105.04(18) . . ?
C3 P1 C1 94.6(4) . . ?
C9 P1 C1 113.4(4) . . ?
C3 P1 C1A 115.2(3) . . ?
C9 P1 C1A 95.9(3) . . ?
C1 P1 C1A 23.4(3) . . ?
C3 P1 Ni1 114.53(14) . . ?
C9 P1 Ni1 117.68(14) . . ?
C1 P1 Ni1 109.3(2) . . ?
C1A P1 Ni1 107.3(2) . . ?
```

C15 P2 C2 106.7(2) . . ?
C15 P2 C21 104.82(17) . . ?
C2 P2 C21 103.7(2) . . ?
C15 P2 Ni1 111.69(14) . . ?
C2 P2 Ni1 109.27(15) . . ?
C21 P2 Ni1 119.65(13) . . ?
O2 N1 O1 127.4(6) . . ?
N1 O1 Ni1 111.6(3) . . ?
O4 N2 O3 114.8(6) . . ?
N2 O3 Ni1 117.2(5) . . ?
O3A N2A O4A 111.0(17) . . ?
N2A O4A Ni1 114.8(15) . . ?
C2 C1 P1 112.0(5) . . ?
C2 C1 H1A 109.2 . . ?
P1 C1 H1A 109.2 . . ?
C2 C1 H1B 109.2 . . ?
P1 C1 H1B 109.2 . . ?
H1A C1 H1B 107.9 . . ?
C2 C1A P1 113.5(5) . . ?
C2 C1A H1A1 108.9 . . ?
P1 C1A H1A1 108.9 . . ?
C2 C1A H1A2 108.9 . . ?
P1 C1A H1A2 108.9 . . ?
H1A1 C1A H1A2 107.7 . . ?
C1A C2 C1 29.9(4) . . ?
C1A C2 P2 114.2(4) . . ?
C1 C2 P2 114.3(4) . . ?
C1A C2 H2A 130.4 . . ?
C1 C2 H2A 108.7 . . ?
P2 C2 H2A 108.7 . . ?
C1A C2 H2B 81.4 . . ?
C1 C2 H2B 108.7 . . ?
P2 C2 H2B 108.7 . . ?
H2A C2 H2B 107.6 . . ?
C8 C3 C4 110.1(3) . . ?
C8 C3 P1 113.9(3) . . ?
C4 C3 P1 111.6(3) . . ?
C8 C3 H3 107.0 . . ?
C4 C3 H3 107.0 . . ?
P1 C3 H3 107.0 . . ?
C5 C4 C3 109.3(4) . . ?
C5 C4 H4A 109.8 . . ?
C3 C4 H4A 109.8 . . ?
C5 C4 H4B 109.8 . . ?
C3 C4 H4B 109.8 . . ?
H4A C4 H4B 108.3 . . ?
C4 C5 C6 111.0(5) . . ?
C4 C5 H5A 109.4 . . ?
C6 C5 H5A 109.4 . . ?
C4 C5 H5B 109.4 . . ?
C6 C5 H5B 109.4 . . ?
H5A C5 H5B 108.0 . . ?
C7 C6 C5 109.8(4) . . ?
C7 C6 H6A 109.7 . . ?
C5 C6 H6A 109.7 . . ?
C7 C6 H6B 109.7 . . ?
C5 C6 H6B 109.7 . . ?

H6A C6 H6B 108.2 . . ?
C8 C7 C6 110.7(4) . . ?
C8 C7 H7A 109.5 . . ?
C6 C7 H7A 109.5 . . ?
C8 C7 H7B 109.5 . . ?
C6 C7 H7B 109.5 . . ?
H7A C7 H7B 108.1 . . ?
C7 C8 C3 110.0(4) . . ?
C7 C8 H8A 109.7 . . ?
C3 C8 H8A 109.7 . . ?
C7 C8 H8B 109.7 . . ?
C3 C8 H8B 109.7 . . ?
H8A C8 H8B 108.2 . . ?
C10 C9 C14 108.7(4) . . ?
C10 C9 P1 115.1(3) . . ?
C14 C9 P1 110.9(3) . . ?
C10 C9 H9 107.3 . . ?
C14 C9 H9 107.3 . . ?
P1 C9 H9 107.3 . . ?
C11 C10 C9 110.2(4) . . ?
C11 C10 H10A 109.6 . . ?
C9 C10 H10A 109.6 . . ?
C11 C10 H10B 109.6 . . ?
C9 C10 H10B 109.6 . . ?
H10A C10 H10B 108.1 . . ?
C12 C11 C10 110.5(4) . . ?
C12 C11 H11A 109.5 . . ?
C10 C11 H11A 109.5 . . ?
C12 C11 H11B 109.5 . . ?
C10 C11 H11B 109.5 . . ?
H11A C11 H11B 108.1 . . ?
C13 C12 C11 110.7(5) . . ?
C13 C12 H12A 109.5 . . ?
C11 C12 H12A 109.5 . . ?
C13 C12 H12B 109.5 . . ?
C11 C12 H12B 109.5 . . ?
H12A C12 H12B 108.1 . . ?
C12 C13 C14 111.1(5) . . ?
C12 C13 H13A 109.4 . . ?
C14 C13 H13A 109.4 . . ?
C12 C13 H13B 109.4 . . ?
C14 C13 H13B 109.4 . . ?
H13A C13 H13B 108.0 . . ?
C13 C14 C9 111.5(4) . . ?
C13 C14 H14A 109.3 . . ?
C9 C14 H14A 109.3 . . ?
C13 C14 H14B 109.3 . . ?
C9 C14 H14B 109.3 . . ?
H14A C14 H14B 108.0 . . ?
C16 C15 C20 110.0(3) . . ?
C16 C15 P2 111.3(3) . . ?
C20 C15 P2 114.9(3) . . ?
C16 C15 H15 106.7 . . ?
C20 C15 H15 106.7 . . ?
P2 C15 H15 106.7 . . ?
C17 C16 C15 110.6(3) . . ?
C17 C16 H16A 109.5 . . ?

C15 C16 H16A 109.5 . . ?
C17 C16 H16B 109.5 . . ?
C15 C16 H16B 109.5 . . ?
H16A C16 H16B 108.1 . . ?
C16 C17 C18 110.2(5) . . ?
C16 C17 H17A 109.6 . . ?
C18 C17 H17A 109.6 . . ?
C16 C17 H17B 109.6 . . ?
C18 C17 H17B 109.6 . . ?
H17A C17 H17B 108.1 . . ?
C19 C18 C17 110.8(4) . . ?
C19 C18 H18A 109.5 . . ?
C17 C18 H18A 109.5 . . ?
C19 C18 H18B 109.5 . . ?
C17 C18 H18B 109.5 . . ?
H18A C18 H18B 108.1 . . ?
C18 C19 C20 110.7(3) . . ?
C18 C19 H19A 109.5 . . ?
C20 C19 H19A 109.5 . . ?
C18 C19 H19B 109.5 . . ?
C20 C19 H19B 109.5 . . ?
H19A C19 H19B 108.1 . . ?
C19 C20 C15 109.9(3) . . ?
C19 C20 H20A 109.7 . . ?
C15 C20 H20A 109.7 . . ?
C19 C20 H20B 109.7 . . ?
C15 C20 H20B 109.7 . . ?
H20A C20 H20B 108.2 . . ?
C26 C21 C22 110.6(3) . . ?
C26 C21 P2 111.5(3) . . ?
C22 C21 P2 114.1(3) . . ?
C26 C21 H21 106.7 . . ?
C22 C21 H21 106.7 . . ?
P2 C21 H21 106.7 . . ?
C23 C22 C21 110.1(3) . . ?
C23 C22 H22A 109.6 . . ?
C21 C22 H22A 109.6 . . ?
C23 C22 H22B 109.6 . . ?
C21 C22 H22B 109.6 . . ?
H22A C22 H22B 108.2 . . ?
C24 C23 C22 110.9(4) . . ?
C24 C23 H23A 109.5 . . ?
C22 C23 H23A 109.5 . . ?
C24 C23 H23B 109.5 . . ?
C22 C23 H23B 109.5 . . ?
H23A C23 H23B 108.0 . . ?
C25 C24 C23 110.4(4) . . ?
C25 C24 H24A 109.6 . . ?
C23 C24 H24A 109.6 . . ?
C25 C24 H24B 109.6 . . ?
C23 C24 H24B 109.6 . . ?
H24A C24 H24B 108.1 . . ?
C24 C25 C26 112.3(4) . . ?
C24 C25 H25A 109.1 . . ?
C26 C25 H25A 109.1 . . ?
C24 C25 H25B 109.1 . . ?
C26 C25 H25B 109.1 . . ?

H25A C25 H25B 107.9 . . ?
C25 C26 C21 110.3(4) . . ?
C25 C26 H26A 109.6 . . ?
C21 C26 H26A 109.6 . . ?
C25 C26 H26B 109.6 . . ?
C21 C26 H26B 109.6 . . ?
H26A C26 H26B 108.1 . . ?
C32 C31 C36 120.0 . . ?
C32 C31 C37 119.2(13) . . ?
C36 C31 C37 120.8(13) . . ?
C31 C32 C33 120.0 . . ?
C31 C32 H32 120.0 . . ?
C33 C32 H32 120.0 . . ?
C32 C33 C34 120.0 . . ?
C32 C33 H33 120.0 . . ?
C34 C33 H33 120.0 . . ?
C33 C34 C35 120.0 . . ?
C33 C34 H34 120.0 . . ?
C35 C34 H34 120.0 . . ?
C36 C35 C34 120.0 . . ?
C36 C35 H35 120.0 . . ?
C34 C35 H35 120.0 . . ?
C35 C36 C31 120.0 . . ?
C35 C36 H36 120.0 . . ?
C31 C36 H36 120.0 . . ?
C31 C37 H37A 109.5 . . ?
C31 C37 H37B 109.5 . . ?
H37A C37 H37B 109.5 . . ?
C31 C37 H37C 109.5 . . ?
H37A C37 H37C 109.5 . . ?
H37B C37 H37C 109.5 . . ?
C32B C31B C36B 120.0 . . ?
C32B C31B C37B 118.4(11) . . ?
C36B C31B C37B 121.6(11) . . ?
C33B C32B C31B 120.0 . . ?
C33B C32B H32B 120.0 . . ?
C31B C32B H32B 120.0 . . ?
C32B C33B C34B 120.0 . . ?
C32B C33B H33B 120.0 . . ?
C34B C33B H33B 120.0 . . ?
C35B C34B C33B 120.0 . . ?
C35B C34B H34B 120.0 . . ?
C33B C34B H34B 120.0 . . ?
C36B C35B C34B 120.0 . . ?
C36B C35B H35B 120.0 . . ?
C34B C35B H35B 120.0 . . ?
C35B C36B C31B 120.0 . . ?
C35B C36B H36B 120.0 . . ?
C31B C36B H36B 120.0 . . ?
C31B C37B H37D 109.5 . . ?
C31B C37B H37E 109.5 . . ?
H37D C37B H37E 109.5 . . ?
C31B C37B H37F 109.5 . . ?
H37D C37B H37F 109.5 . . ?
H37E C37B H37F 109.5 . . ?

loop_

```
 _geom_torsion_atom_site_label_1
 _geom_torsion_atom_site_label_2
 _geom_torsion_atom_site_label_3
 _geom_torsion_atom_site_label_4
 _geom_torsion
 _geom_torsion_site_symmetry_1
 _geom_torsion_site_symmetry_2
 _geom_torsion_site_symmetry_3
 _geom_torsion_site_symmetry_4
 _geom_torsion_publ_flag
O4A Ni1 P1 C3 147.1(9) . . . . ?
O1 Ni1 P1 C3 74.09(19) . . . . ?
O3 Ni1 P1 C3 -32.5(7) . . . . ?
P2 Ni1 P1 C3 -117.48(16) . . . . ?
O4A Ni1 P1 C9 22.9(9) . . . . ?
O1 Ni1 P1 C9 -50.07(18) . . . . ?
O3 Ni1 P1 C9 -156.6(7) . . . . ?
P2 Ni1 P1 C9 118.36(15) . . . . ?
O4A Ni1 P1 C1 -108.3(10) . . . . ?
O1 Ni1 P1 C1 178.8(5) . . . . ?
O3 Ni1 P1 C1 72.2(8) . . . . ?
P2 Ni1 P1 C1 -12.8(5) . . . . ?
O4A Ni1 P1 C1A -83.7(10) . . . . ?
O1 Ni1 P1 C1A -156.6(4) . . . . ?
O3 Ni1 P1 C1A 96.8(8) . . . . ?
P2 Ni1 P1 C1A 11.8(4) . . . . ?
O4A Ni1 P2 C15 44.1(4) . . . . ?
O1 Ni1 P2 C15 -30.8(6) . . . . ?
O3 Ni1 P2 C15 74.6(2) . . . . ?
P1 Ni1 P2 C15 -117.39(14) . . . . ?
O4A Ni1 P2 C2 162.0(5) . . . . ?
O1 Ni1 P2 C2 87.1(6) . . . . ?
O3 Ni1 P2 C2 -167.5(3) . . . . ?
P1 Ni1 P2 C2 0.5(2) . . . . ?
O4A Ni1 P2 C21 -78.8(4) . . . . ?
O1 Ni1 P2 C21 -153.7(5) . . . . ?
O3 Ni1 P2 C21 -48.3(2) . . . . ?
P1 Ni1 P2 C21 119.70(14) . . . . ?
O2 N1 O1 Ni1 6.5(7) . . . . ?
O4A Ni1 O1 N1 105.6(5) . . . . ?
O3 Ni1 O1 N1 76.2(3) . . . . ?
P1 Ni1 O1 N1 -92.2(3) . . . . ?
P2 Ni1 O1 N1 -178.8(4) . . . . ?
O4 N2 O3 Ni1 5.7(7) . . . . ?
O4A Ni1 O3 N2 0.4(7) . . . . ?
O1 Ni1 O3 N2 73.9(5) . . . . ?
P1 Ni1 O3 N2 -179.9(5) . . . . ?
P2 Ni1 O3 N2 -94.9(4) . . . . ?
O3A N2A O4A Ni1 -6.8(17) . . . . ?
O1 Ni1 O4A N2A -84.0(10) . . . . ?
O3 Ni1 O4A N2A 22.0(9) . . . . ?
P1 Ni1 O4A N2A -157.8(9) . . . . ?
P2 Ni1 O4A N2A 107.2(10) . . . . ?
C3 P1 C1 C2 143.4(7) . . . . ?
C9 P1 C1 C2 -108.1(7) . . . . ?
C1A P1 C1 C2 -63.8(9) . . . . ?
Ni1 P1 C1 C2 25.4(9) . . . . ?
```

C3 P1 C1A C2 102.7(6) . . . . ?
C9 P1 C1A C2 -147.6(6) . . . . ?
C1 P1 C1A C2 72.5(9) . . . . ?
Ni1 P1 C1A C2 -26.1(8) . . . . ?
P1 C1A C2 C1 -69.4(9) . . . . ?
P1 C1A C2 P2 27.6(8) . . . . ?
P1 C1 C2 C1A 71.0(9) . . . . ?
P1 C1 C2 P2 -25.7(10) . . . . ?
C15 P2 C2 C1A 103.5(6) . . . . ?
C21 P2 C2 C1A -146.1(6) . . . . ?
Ni1 P2 C2 C1A -17.4(6) . . . . ?
C15 P2 C2 C1 136.3(7) . . . . ?
C21 P2 C2 C1 -113.3(7) . . . . ?
Ni1 P2 C2 C1 15.4(7) . . . . ?
C9 P1 C3 C8 -53.5(3) . . . . ?
C1 P1 C3 C8 62.2(4) . . . . ?
C1A P1 C3 C8 50.6(4) . . . . ?
Ni1 P1 C3 C8 175.9(2) . . . . ?
C9 P1 C3 C4 -178.9(3) . . . . ?
C1 P1 C3 C4 -63.2(5) . . . . ?
C1A P1 C3 C4 -74.8(4) . . . . ?
Ni1 P1 C3 C4 50.5(4) . . . . ?
C8 C3 C4 C5 58.2(6) . . . . ?
P1 C3 C4 C5 -174.3(4) . . . . ?
C3 C4 C5 C6 -58.3(6) . . . . ?
C4 C5 C6 C7 58.2(7) . . . . ?
C5 C6 C7 C8 -58.0(7) . . . . ?
C6 C7 C8 C3 58.5(5) . . . . ?
C4 C3 C8 C7 -58.5(5) . . . . ?
P1 C3 C8 C7 175.3(3) . . . . ?
C3 P1 C9 C10 -49.0(3) . . . . ?
C1 P1 C9 C10 -151.0(4) . . . . ?
C1A P1 C9 C10 -167.2(4) . . . . ?
Ni1 P1 C9 C10 79.7(3) . . . . ?
C3 P1 C9 C14 -173.0(3) . . . . ?
C1 P1 C9 C14 85.1(5) . . . . ?
C1A P1 C9 C14 68.9(4) . . . . ?
Ni1 P1 C9 C14 -44.2(4) . . . . ?
C14 C9 C10 C11 -57.4(4) . . . . ?
P1 C9 C10 C11 177.5(3) . . . . ?
C9 C10 C11 C12 59.2(6) . . . . ?
C10 C11 C12 C13 -58.3(6) . . . . ?
C11 C12 C13 C14 56.9(7) . . . . ?
C12 C13 C14 C9 -56.6(7) . . . . ?
C10 C9 C14 C13 56.4(6) . . . . ?
P1 C9 C14 C13 -176.1(5) . . . . ?
C2 P2 C15 C16 -67.9(4) . . . . ?
C21 P2 C15 C16 -177.5(3) . . . . ?
Ni1 P2 C15 C16 51.5(3) . . . . ?
C2 P2 C15 C20 58.0(3) . . . . ?
C21 P2 C15 C20 -51.7(3) . . . . ?
Ni1 P2 C15 C20 177.3(2) . . . . ?
C20 C15 C16 C17 58.3(5) . . . . ?
P2 C15 C16 C17 -173.2(4) . . . . ?
C15 C16 C17 C18 -57.5(6) . . . . ?
C16 C17 C18 C19 57.0(6) . . . . ?
C17 C18 C19 C20 -57.6(6) . . . . ?

```
C18 C19 C20 C15 58.0(5) . . . . ?
C16 C15 C20 C19 -58.1(4) . . . . ?
P2 C15 C20 C19 175.4(3) . . . . ?
C15 P2 C21 C26 -176.1(3) . . . . ?
C2 P2 C21 C26 72.1(3) . . . . ?
Ni1 P2 C21 C26 -49.9(3) . . . . ?
C15 P2 C21 C22 -49.9(3) . . . . ?
C2 P2 C21 C22 -161.7(3) . . . . ?
Ni1 P2 C21 C22 76.3(3) . . . . ?
C26 C21 C22 C23 -57.7(5) . . . . ?
P2 C21 C22 C23 175.6(3) . . . . ?
C21 C22 C23 C24 58.0(5) . . . . ?
C22 C23 C24 C25 -56.7(5) . . . . ?
C23 C24 C25 C26 55.7(5) . . . . ?
C24 C25 C26 C21 -55.6(5) . . . . ?
C22 C21 C26 C25 56.2(5) . . . . ?
P2 C21 C26 C25 -175.7(3) . . . . ?
C36 C31 C32 C33 0.0 . . . . ?
C37 C31 C32 C33 -179.9(11) . . . . ?
C31 C32 C33 C34 0.0 . . . . ?
C32 C33 C34 C35 0.0 . . . . ?
C33 C34 C35 C36 0.0 . . . . ?
C34 C35 C36 C31 0.0 . . . . ?
C32 C31 C36 C35 0.0 . . . . ?
C37 C31 C36 C35 179.9(11) . . . . ?
C36B C31B C32B C33B 0.0 . . . . ?
C37B C31B C32B C33B -179.3(13) . . . . ?
C31B C32B C33B C34B 0.0 . . . . ?
C32B C33B C34B C35B 0.0 . . . . ?
C33B C34B C35B C36B 0.0 . . . . ?
C34B C35B C36B C31B 0.0 . . . . ?
C32B C31B C36B C35B 0.0 . . . . ?
C37B C31B C36B C35B 179.2(13) . . . . ?

_diffrn_measured_fraction_theta_max    0.973
_diffrn_reflns_theta_full              29.73
_diffrn_measured_fraction_theta_full   0.973
_refine_diff_density_max    4.628
_refine_diff_density_min   -1.192
_refine_diff_density_rms    0.125


data_bath1003

_audit_creation_method            SHELXL-97
_chemical_name_systematic
;
Bis(tricyclohexanephosphine)bis(n1-nitro)nickel(II)
;
_chemical_name_common             ?
_chemical_melting_point           ?
_chemical_formula_moiety          'C36 H66 N2 Ni O4 P2'
_chemical_formula_sum             'C36 H66 N2 Ni O4 P2'
_chemical_formula_weight          711.56

loop_
 _atom_type_symbol
```

```
 _atom_type_description
 _atom_type_scat_dispersion_real
 _atom_type_scat_dispersion_imag
 _atom_type_scat_source
 'C'  'C'   0.0033   0.0016
 'International Tables Vol C Tables 4.2.6.8 and 6.1.1.4'
 'H'  'H'   0.0000   0.0000
 'International Tables Vol C Tables 4.2.6.8 and 6.1.1.4'
 'N'  'N'   0.0061   0.0033
 'International Tables Vol C Tables 4.2.6.8 and 6.1.1.4'
 'O'  'O'   0.0106   0.0060
 'International Tables Vol C Tables 4.2.6.8 and 6.1.1.4'
 'P'  'P'   0.1023   0.0942
 'International Tables Vol C Tables 4.2.6.8 and 6.1.1.4'
 'Ni'  'Ni'   0.3393   1.1124
 'International Tables Vol C Tables 4.2.6.8 and 6.1.1.4'

_symmetry_cell_setting            triclinic
_symmetry_space_group_name_H-M    P-1

loop_
 _symmetry_equiv_pos_as_xyz
 'x, y, z'
 '-x, -y, -z'

_cell_length_a                    10.084(2)
_cell_length_b                    10.369(2)
_cell_length_c                    10.464(2)
_cell_angle_alpha                 111.269(3)
_cell_angle_beta                  110.575(3)
_cell_angle_gamma                 94.503(3)
_cell_volume                      927.5(3)
_cell_formula_units_Z             1
_cell_measurement_temperature     100(2)
_cell_measurement_reflns_used     3795
_cell_measurement_theta_min       2.96
_cell_measurement_theta_max       33.65

_exptl_crystal_description        block
_exptl_crystal_colour             orange
_exptl_crystal_size_max           0.04
_exptl_crystal_size_mid           0.03
_exptl_crystal_size_min           0.03
_exptl_crystal_density_meas       ?
_exptl_crystal_density_diffrn     1.274
_exptl_crystal_density_method     'not measured'
_exptl_crystal_F_000              386
_exptl_absorpt_coefficient_mu     0.648
_exptl_absorpt_correction_T_min   0.5457
_exptl_absorpt_correction_T_max   0.7466
_exptl_absorpt_correction_type    'multi-scan'
_exptl_absorpt_process_details    'SORTAV (Blessing, 1995)'

_exptl_special_details
;
multi-scan from symmetry-related measurements
 Sortav (Blessing 1995)
```

```
;

_diffrn_ambient_temperature       100(2)
_diffrn_radiation_wavelength      0.77490
_diffrn_radiation_type            Synchrotron
_diffrn_radiation_source          'Station 11.3.1 ALS'
_diffrn_radiation_monochromator   'Channel-cut Si(111)'
_diffrn_measurement_device_type   'Bruker APEX2'
_diffrn_measurement_method        '3000 0.3 degree images with \f and
\w scans'
_diffrn_detector_area_resol_mean  ?
_diffrn_standards_number          ?
_diffrn_standards_interval_count  ?
_diffrn_standards_interval_time   ?
_diffrn_standards_decay_%         0
_diffrn_reflns_number             13115
_diffrn_reflns_av_R_equivalents   0.0926
_diffrn_reflns_av_sigmaI/netI     0.1306
_diffrn_reflns_limit_h_min        -14
_diffrn_reflns_limit_h_max        14
_diffrn_reflns_limit_k_min        -14
_diffrn_reflns_limit_k_max        14
_diffrn_reflns_limit_l_min        -14
_diffrn_reflns_limit_l_max        14
_diffrn_reflns_theta_min          2.96
_diffrn_reflns_theta_max          33.69
_reflns_number_total              5575
_reflns_number_gt                 4530
_reflns_threshold_expression      >2sigma(I)

_computing_data_collection      'Bruker SMART'
_computing_cell_refinement      'Bruker SMART'
_computing_data_reduction       'Bruker SAINT'
_computing_structure_solution   'SHELXTS (Sheldrick, 1997)'
_computing_structure_refinement 'SHELXTL (Sheldrick, 1997)'
_computing_molecular_graphics   'ORTEP'
_computing_publication_material 'SHELXTL (Sheldrick, 1997)'

_refine_special_details
;
 Refinement of F^2^ against ALL reflections.  The weighted R-factor wR
and
 goodness of fit S are based on F^2^, conventional R-factors R are
based
 on F, with F set to zero for negative F^2^. The threshold expression
of
 F^2^ > 2sigma(F^2^) is used only for calculating R-factors(gt) etc.
and is
 not relevant to the choice of reflections for refinement.  R-factors
based
 on F^2^ are statistically about twice as large as those based on F,
and R-
 factors based on ALL data will be even larger.
;

_refine_ls_structure_factor_coef  Fsqd
_refine_ls_matrix_type            full
```

```
_refine_ls_weighting_scheme       calc
_refine_ls_weighting_details
 'calc w=1/[\s^2^(Fo^2^)+(0.1000P)^2^+0.0000P] where
P=(Fo^2^+2Fc^2^)/3'
_atom_sites_solution_primary      direct
_atom_sites_solution_secondary    difmap
_atom_sites_solution_hydrogens    geom
_refine_ls_hydrogen_treatment     constr
_refine_ls_extinction_method      none
_refine_ls_extinction_coef        ?
_refine_ls_number_reflns          5575
_refine_ls_number_parameters      206
_refine_ls_number_restraints      0
_refine_ls_R_factor_all           0.0888
_refine_ls_R_factor_gt            0.0778
_refine_ls_wR_factor_ref          0.2118
_refine_ls_wR_factor_gt           0.2008
_refine_ls_goodness_of_fit_ref    1.083
_refine_ls_restrained_S_all       1.083
_refine_ls_shift/su_max           0.000
_refine_ls_shift/su_mean          0.000

loop_
 _atom_site_label
 _atom_site_type_symbol
 _atom_site_fract_x
 _atom_site_fract_y
 _atom_site_fract_z
 _atom_site_U_iso_or_equiv
 _atom_site_adp_type
 _atom_site_occupancy
 _atom_site_symmetry_multiplicity
 _atom_site_calc_flag
 _atom_site_refinement_flags
 _atom_site_disorder_assembly
 _atom_site_disorder_group
Ni1 Ni 0.5000 0.5000 0.5000 0.01221(15) Uani 1 2 d S . .
P1 P 0.29716(6) 0.46552(6) 0.29217(7) 0.01185(16) Uani 1 1 d . . .
O2 O 0.3273(2) 0.5236(3) 0.6510(2) 0.0274(5) Uani 1 1 d . . .
O1 O 0.3460(2) 0.3095(2) 0.5463(3) 0.0270(5) Uani 1 1 d . . .
N1 N 0.3784(2) 0.4376(2) 0.5798(2) 0.0175(4) Uani 1 1 d . . .
C7 C 0.3271(3) 0.3936(3) 0.1179(3) 0.0138(4) Uani 1 1 d . . .
H7 H 0.4079 0.4673 0.1327 0.017 Uiso 1 1 calc R . .
C9 C 0.4325(3) 0.2233(3) -0.0381(3) 0.0191(5) Uani 1 1 d . . .
H9A H 0.4643 0.1327 -0.0576 0.023 Uiso 1 1 calc R . .
H9B H 0.5168 0.2997 -0.0088 0.023 Uiso 1 1 calc R . .
C1 C 0.1293(3) 0.3533(3) 0.2705(3) 0.0146(4) Uani 1 1 d . . .
H1 H 0.1348 0.3820 0.3747 0.017 Uiso 1 1 calc R . .
C13 C 0.2449(3) 0.6353(3) 0.3008(3) 0.0151(4) Uani 1 1 d . . .
H13 H 0.1460 0.6100 0.2182 0.018 Uiso 1 1 calc R . .
C18 C 0.2307(3) 0.7137(3) 0.4497(3) 0.0202(5) Uani 1 1 d . . .
H18A H 0.1606 0.6499 0.4600 0.024 Uiso 1 1 calc R . .
H18B H 0.3264 0.7382 0.5346 0.024 Uiso 1 1 calc R . .
C3 C 0.0032(3) 0.1128(3) 0.2270(3) 0.0208(5) Uani 1 1 d . . .
H3A H 0.0212 0.1463 0.3344 0.025 Uiso 1 1 calc R . .
H3B H 0.0019 0.0101 0.1876 0.025 Uiso 1 1 calc R . .
C10 C 0.3095(3) 0.2109(3) -0.1820(3) 0.0198(5) Uani 1 1 d . . .
```

```
H10A H 0.3467 0.1963 -0.2609 0.024 Uiso 1 1 calc R . .
H10B H 0.2299 0.1272 -0.2184 0.024 Uiso 1 1 calc R . .
C12 C 0.1998(3) 0.3768(3) -0.0256(3) 0.0173(5) Uani 1 1 d . . .
H12A H 0.1644 0.4655 -0.0076 0.021 Uiso 1 1 calc R . .
H12B H 0.1184 0.2980 -0.0534 0.021 Uiso 1 1 calc R . .
C8 C 0.3842(3) 0.2563(3) 0.0911(3) 0.0167(5) Uani 1 1 d . . .
H8A H 0.4675 0.2688 0.1836 0.020 Uiso 1 1 calc R . .
H8B H 0.3063 0.1757 0.0666 0.020 Uiso 1 1 calc R . .
C4 C -0.1447(3) 0.1338(3) 0.1406(3) 0.0227(6) Uani 1 1 d . . .
H4A H -0.1684 0.0899 0.0316 0.027 Uiso 1 1 calc R . .
H4B H -0.2212 0.0859 0.1581 0.027 Uiso 1 1 calc R . .
C6 C -0.0179(3) 0.3755(3) 0.1808(3) 0.0172(5) Uani 1 1 d . . .
H6A H -0.0170 0.4781 0.2210 0.021 Uiso 1 1 calc R . .
H6B H -0.0332 0.3433 0.0742 0.021 Uiso 1 1 calc R . .
C11 C 0.2499(3) 0.3451(3) -0.1544(3) 0.0197(5) Uani 1 1 d . . .
H11A H 0.1667 0.3320 -0.2472 0.024 Uiso 1 1 calc R . .
H11B H 0.3267 0.4270 -0.1290 0.024 Uiso 1 1 calc R . .
C2 C 0.1264(3) 0.1943(3) 0.2128(3) 0.0165(5) Uani 1 1 d . . .
H2A H 0.1122 0.1567 0.1061 0.020 Uiso 1 1 calc R . .
H2B H 0.2209 0.1803 0.2714 0.020 Uiso 1 1 calc R . .
C5 C -0.1426(3) 0.2919(3) 0.1912(3) 0.0211(5) Uani 1 1 d . . .
H5A H -0.1329 0.3321 0.2964 0.025 Uiso 1 1 calc R . .
H5B H -0.2365 0.3032 0.1280 0.025 Uiso 1 1 calc R . .
C15 C 0.2919(3) 0.8703(3) 0.2839(3) 0.0209(5) Uani 1 1 d . . .
H15A H 0.3605 0.9346 0.2727 0.025 Uiso 1 1 calc R . .
H15B H 0.1955 0.8448 0.1996 0.025 Uiso 1 1 calc R . .
C17 C 0.1784(3) 0.8499(3) 0.4568(3) 0.0223(5) Uani 1 1 d . . .
H17A H 0.0782 0.8246 0.3788 0.027 Uiso 1 1 calc R . .
H17B H 0.1760 0.9006 0.5561 0.027 Uiso 1 1 calc R . .
C14 C 0.3461(3) 0.7349(3) 0.2771(3) 0.0196(5) Uani 1 1 d . . .
H14A H 0.4461 0.7610 0.3558 0.023 Uiso 1 1 calc R . .
H14B H 0.3495 0.6848 0.1782 0.023 Uiso 1 1 calc R . .
C16 C 0.2787(3) 0.9476(3) 0.4324(3) 0.0222(5) Uani 1 1 d . . .
H16A H 0.3765 0.9808 0.5163 0.027 Uiso 1 1 calc R . .
H16B H 0.2396 1.0321 0.4319 0.027 Uiso 1 1 calc R . .

loop_
 _atom_site_aniso_label
 _atom_site_aniso_U_11
 _atom_site_aniso_U_22
 _atom_site_aniso_U_33
 _atom_site_aniso_U_23
 _atom_site_aniso_U_13
 _atom_site_aniso_U_12
Ni1 0.0114(2) 0.0154(2) 0.0101(2) 0.00575(18) 0.00431(16) 0.00236(16)
P1 0.0122(3) 0.0126(3) 0.0101(3) 0.0048(2) 0.0040(2) 0.0026(2)
O2 0.0237(10) 0.0383(12) 0.0224(10) 0.0120(9) 0.0123(8) 0.0082(9)
O1 0.0256(10) 0.0295(11) 0.0265(11) 0.0166(9) 0.0071(8) 0.0015(8)
N1 0.0115(9) 0.0198(10) 0.0131(10) 0.0055(8) -0.0023(7) 0.0028(8)
C7 0.0142(10) 0.0151(11) 0.0098(10) 0.0034(9) 0.0044(8) 0.0039(8)
C9 0.0193(12) 0.0195(12) 0.0168(12) 0.0040(10) 0.0092(10) 0.0053(9)
C1 0.0126(10) 0.0154(11) 0.0162(11) 0.0071(9) 0.0061(9) 0.0031(8)
C13 0.0163(10) 0.0147(11) 0.0145(11) 0.0060(9) 0.0065(9) 0.0042(8)
C18 0.0279(13) 0.0173(12) 0.0194(12) 0.0077(10) 0.0138(11) 0.0060(10)
C3 0.0181(12) 0.0196(12) 0.0284(14) 0.0130(11) 0.0107(10) 0.0031(9)
C10 0.0234(12) 0.0197(12) 0.0124(11) 0.0023(10) 0.0081(10) 0.0015(10)
C12 0.0197(12) 0.0201(12) 0.0110(10) 0.0064(10) 0.0051(9) 0.0044(9)
```

```
C8 0.0193(11) 0.0173(12) 0.0157(11) 0.0071(10) 0.0088(9) 0.0069(9)
C4 0.0164(12) 0.0255(14) 0.0283(14) 0.0158(12) 0.0070(11) 0.0039(10)
C6 0.0135(10) 0.0188(12) 0.0194(12) 0.0097(10) 0.0050(9) 0.0044(9)
C11 0.0217(12) 0.0216(13) 0.0152(11) 0.0085(10) 0.0065(10) 0.0031(10)
C2 0.0151(11) 0.0144(11) 0.0201(12) 0.0074(10) 0.0073(9) 0.0024(8)
C5 0.0134(11) 0.0272(14) 0.0238(13) 0.0119(11) 0.0074(10) 0.0057(10)
C15 0.0239(13) 0.0172(12) 0.0231(13) 0.0117(11) 0.0075(10) 0.0058(10)
C17 0.0253(13) 0.0186(13) 0.0245(14) 0.0067(11) 0.0135(11) 0.0086(10)
C14 0.0218(12) 0.0178(12) 0.0236(13) 0.0105(11) 0.0124(10) 0.0050(9)
C16 0.0227(13) 0.0169(12) 0.0241(13) 0.0089(11) 0.0060(11) 0.0054(10)

_geom_special_details
;
 All esds (except the esd in the dihedral angle between two l.s.
planes)
 are estimated using the full covariance matrix.  The cell esds are
taken
 into account individually in the estimation of esds in distances,
angles
 and torsion angles; correlations between esds in cell parameters are
only
 used when they are defined by crystal symmetry.  An approximate
(isotropic)
 treatment of cell esds is used for estimating esds involving l.s.
planes.
;

loop_
 _geom_bond_atom_site_label_1
 _geom_bond_atom_site_label_2
 _geom_bond_distance
 _geom_bond_site_symmetry_2
 _geom_bond_publ_flag
Ni1 N1 1.899(2) . ?
Ni1 N1 1.899(2) 2_666 ?
Ni1 P1 2.2881(7) 2_666 ?
Ni1 P1 2.2881(7) . ?
P1 C7 1.846(2) . ?
P1 C13 1.857(3) . ?
P1 C1 1.871(2) . ?
O2 N1 1.215(3) . ?
O1 N1 1.228(3) . ?
C7 C12 1.534(4) . ?
C7 C8 1.546(3) . ?
C7 H7 1.0000 . ?
C9 C8 1.528(4) . ?
C9 C10 1.531(4) . ?
C9 H9A 0.9900 . ?
C9 H9B 0.9900 . ?
C1 C2 1.530(3) . ?
C1 C6 1.539(3) . ?
C1 H1 1.0000 . ?
C13 C14 1.539(3) . ?
C13 C18 1.538(4) . ?
C13 H13 1.0000 . ?
C18 C17 1.532(4) . ?
C18 H18A 0.9900 . ?
```

```
C18 H18B 0.9900 . ?
C3 C2 1.529(3) . ?
C3 C4 1.528(4) . ?
C3 H3A 0.9900 . ?
C3 H3B 0.9900 . ?
C10 C11 1.530(4) . ?
C10 H10A 0.9900 . ?
C10 H10B 0.9900 . ?
C12 C11 1.537(4) . ?
C12 H12A 0.9900 . ?
C12 H12B 0.9900 . ?
C8 H8A 0.9900 . ?
C8 H8B 0.9900 . ?
C4 C5 1.525(4) . ?
C4 H4A 0.9900 . ?
C4 H4B 0.9900 . ?
C6 C5 1.529(3) . ?
C6 H6A 0.9900 . ?
C6 H6B 0.9900 . ?
C11 H11A 0.9900 . ?
C11 H11B 0.9900 . ?
C2 H2A 0.9900 . ?
C2 H2B 0.9900 . ?
C5 H5A 0.9900 . ?
C5 H5B 0.9900 . ?
C15 C16 1.527(4) . ?
C15 C14 1.533(4) . ?
C15 H15A 0.9900 . ?
C15 H15B 0.9900 . ?
C17 C16 1.524(4) . ?
C17 H17A 0.9900 . ?
C17 H17B 0.9900 . ?
C14 H14A 0.9900 . ?
C14 H14B 0.9900 . ?
C16 H16A 0.9900 . ?
C16 H16B 0.9900 . ?

loop_
 _geom_angle_atom_site_label_1
 _geom_angle_atom_site_label_2
 _geom_angle_atom_site_label_3
 _geom_angle
 _geom_angle_site_symmetry_1
 _geom_angle_site_symmetry_3
 _geom_angle_publ_flag
N1 Ni1 N1 179.999(1) . 2_666 ?
N1 Ni1 P1 90.61(7) . 2_666 ?
N1 Ni1 P1 89.39(7) 2_666 2_666 ?
N1 Ni1 P1 89.39(7) . . ?
N1 Ni1 P1 90.61(7) 2_666 . ?
P1 Ni1 P1 180.0 2_666 . ?
C7 P1 C13 104.66(11) . . ?
C7 P1 C1 110.21(12) . . ?
C13 P1 C1 103.27(11) . . ?
C7 P1 Ni1 112.23(8) . . ?
C13 P1 Ni1 111.86(8) . . ?
C1 P1 Ni1 113.87(8) . . ?
```

O2 N1 O1 123.1(3) . . ?
O2 N1 Ni1 118.88(19) . . ?
O1 N1 Ni1 117.79(19) . . ?
C12 C7 C8 110.3(2) . . ?
C12 C7 P1 115.59(17) . . ?
C8 C7 P1 114.76(16) . . ?
C12 C7 H7 105.0 . . ?
C8 C7 H7 105.0 . . ?
P1 C7 H7 105.0 . . ?
C8 C9 C10 111.8(2) . . ?
C8 C9 H9A 109.3 . . ?
C10 C9 H9A 109.3 . . ?
C8 C9 H9B 109.3 . . ?
C10 C9 H9B 109.3 . . ?
H9A C9 H9B 107.9 . . ?
C2 C1 C6 109.8(2) . . ?
C2 C1 P1 113.26(16) . . ?
C6 C1 P1 116.80(17) . . ?
C2 C1 H1 105.3 . . ?
C6 C1 H1 105.3 . . ?
P1 C1 H1 105.3 . . ?
C14 C13 C18 110.5(2) . . ?
C14 C13 P1 114.97(17) . . ?
C18 C13 P1 109.87(17) . . ?
C14 C13 H13 107.0 . . ?
C18 C13 H13 107.0 . . ?
P1 C13 H13 107.0 . . ?
C17 C18 C13 111.4(2) . . ?
C17 C18 H18A 109.3 . . ?
C13 C18 H18A 109.3 . . ?
C17 C18 H18B 109.3 . . ?
C13 C18 H18B 109.3 . . ?
H18A C18 H18B 108.0 . . ?
C2 C3 C4 111.4(2) . . ?
C2 C3 H3A 109.3 . . ?
C4 C3 H3A 109.3 . . ?
C2 C3 H3B 109.3 . . ?
C4 C3 H3B 109.3 . . ?
H3A C3 H3B 108.0 . . ?
C11 C10 C9 111.1(2) . . ?
C11 C10 H10A 109.4 . . ?
C9 C10 H10A 109.4 . . ?
C11 C10 H10B 109.4 . . ?
C9 C10 H10B 109.4 . . ?
H10A C10 H10B 108.0 . . ?
C7 C12 C11 109.8(2) . . ?
C7 C12 H12A 109.7 . . ?
C11 C12 H12A 109.7 . . ?
C7 C12 H12B 109.7 . . ?
C11 C12 H12B 109.7 . . ?
H12A C12 H12B 108.2 . . ?
C9 C8 C7 109.9(2) . . ?
C9 C8 H8A 109.7 . . ?
C7 C8 H8A 109.7 . . ?
C9 C8 H8B 109.7 . . ?
C7 C8 H8B 109.7 . . ?
H8A C8 H8B 108.2 . . ?

```
C5 C4 C3 110.5(2) . . ?
C5 C4 H4A 109.5 . . ?
C3 C4 H4A 109.5 . . ?
C5 C4 H4B 109.5 . . ?
C3 C4 H4B 109.5 . . ?
H4A C4 H4B 108.1 . . ?
C5 C6 C1 110.5(2) . . ?
C5 C6 H6A 109.5 . . ?
C1 C6 H6A 109.5 . . ?
C5 C6 H6B 109.5 . . ?
C1 C6 H6B 109.5 . . ?
H6A C6 H6B 108.1 . . ?
C10 C11 C12 111.1(2) . . ?
C10 C11 H11A 109.4 . . ?
C12 C11 H11A 109.4 . . ?
C10 C11 H11B 109.4 . . ?
C12 C11 H11B 109.4 . . ?
H11A C11 H11B 108.0 . . ?
C3 C2 C1 110.5(2) . . ?
C3 C2 H2A 109.6 . . ?
C1 C2 H2A 109.6 . . ?
C3 C2 H2B 109.6 . . ?
C1 C2 H2B 109.6 . . ?
H2A C2 H2B 108.1 . . ?
C4 C5 C6 112.5(2) . . ?
C4 C5 H5A 109.1 . . ?
C6 C5 H5A 109.1 . . ?
C4 C5 H5B 109.1 . . ?
C6 C5 H5B 109.1 . . ?
H5A C5 H5B 107.8 . . ?
C16 C15 C14 111.2(2) . . ?
C16 C15 H15A 109.4 . . ?
C14 C15 H15A 109.4 . . ?
C16 C15 H15B 109.4 . . ?
C14 C15 H15B 109.4 . . ?
H15A C15 H15B 108.0 . . ?
C16 C17 C18 111.1(2) . . ?
C16 C17 H17A 109.4 . . ?
C18 C17 H17A 109.4 . . ?
C16 C17 H17B 109.4 . . ?
C18 C17 H17B 109.4 . . ?
H17A C17 H17B 108.0 . . ?
C15 C14 C13 110.6(2) . . ?
C15 C14 H14A 109.5 . . ?
C13 C14 H14A 109.5 . . ?
C15 C14 H14B 109.5 . . ?
C13 C14 H14B 109.5 . . ?
H14A C14 H14B 108.1 . . ?
C17 C16 C15 111.0(2) . . ?
C17 C16 H16A 109.4 . . ?
C15 C16 H16A 109.4 . . ?
C17 C16 H16B 109.4 . . ?
C15 C16 H16B 109.4 . . ?
H16A C16 H16B 108.0 . . ?

loop_
 _geom_torsion_atom_site_label_1
```

```
 _geom_torsion_atom_site_label_2
 _geom_torsion_atom_site_label_3
 _geom_torsion_atom_site_label_4
 _geom_torsion
 _geom_torsion_site_symmetry_1
 _geom_torsion_site_symmetry_2
 _geom_torsion_site_symmetry_3
 _geom_torsion_site_symmetry_4
 _geom_torsion_publ_flag
N1 Ni1 P1 C7 -142.01(11) . . . . ?
N1 Ni1 P1 C7 37.99(11) 2_666 . . . ?
P1 Ni1 P1 C7 -33(5) 2_666 . . . ?
N1 Ni1 P1 C13 100.70(11) . . . . ?
N1 Ni1 P1 C13 -79.30(11) 2_666 . . . ?
P1 Ni1 P1 C13 -150(5) 2_666 . . . ?
N1 Ni1 P1 C1 -15.92(11) . . . . ?
N1 Ni1 P1 C1 164.08(11) 2_666 . . . ?
P1 Ni1 P1 C1 93(5) 2_666 . . . ?
N1 Ni1 N1 O2 37(20) 2_666 . . . ?
P1 Ni1 N1 O2 94.04(19) 2_666 . . . ?
P1 Ni1 N1 O2 -85.96(19) . . . . ?
N1 Ni1 N1 O1 -148(20) 2_666 . . . ?
P1 Ni1 N1 O1 -91.73(18) 2_666 . . . ?
P1 Ni1 N1 O1 88.27(18) . . . . ?
C13 P1 C7 C12 -54.5(2) . . . . ?
C1 P1 C7 C12 55.9(2) . . . . ?
Ni1 P1 C7 C12 -176.00(15) . . . . ?
C13 P1 C7 C8 175.39(18) . . . . ?
C1 P1 C7 C8 -74.2(2) . . . . ?
Ni1 P1 C7 C8 53.88(19) . . . . ?
C7 P1 C1 C2 50.9(2) . . . . ?
C13 P1 C1 C2 162.19(18) . . . . ?
Ni1 P1 C1 C2 -76.29(19) . . . . ?
C7 P1 C1 C6 -78.2(2) . . . . ?
C13 P1 C1 C6 33.2(2) . . . . ?
Ni1 P1 C1 C6 154.70(16) . . . . ?
C7 P1 C13 C14 -49.3(2) . . . . ?
C1 P1 C13 C14 -164.66(19) . . . . ?
Ni1 P1 C13 C14 72.5(2) . . . . ?
C7 P1 C13 C18 -174.70(17) . . . . ?
C1 P1 C13 C18 69.9(2) . . . . ?
Ni1 P1 C13 C18 -52.95(19) . . . . ?
C14 C13 C18 C17 55.6(3) . . . . ?
P1 C13 C18 C17 -176.46(18) . . . . ?
C8 C9 C10 C11 55.2(3) . . . . ?
C8 C7 C12 C11 -59.0(3) . . . . ?
P1 C7 C12 C11 168.80(18) . . . . ?
C10 C9 C8 C7 -56.2(3) . . . . ?
C12 C7 C8 C9 58.2(3) . . . . ?
P1 C7 C8 C9 -169.10(17) . . . . ?
C2 C3 C4 C5 -55.0(3) . . . . ?
C2 C1 C6 C5 57.1(3) . . . . ?
P1 C1 C6 C5 -172.26(19) . . . . ?
C9 C10 C11 C12 -55.6(3) . . . . ?
C7 C12 C11 C10 57.7(3) . . . . ?
C4 C3 C2 C1 58.1(3) . . . . ?
C6 C1 C2 C3 -58.6(3) . . . . ?
```

```
P1 C1 C2 C3 168.85(18) . . . . ?
C3 C4 C5 C6 54.0(3) . . . . ?
C1 C6 C5 C4 -55.5(3) . . . . ?
C13 C18 C17 C16 -55.8(3) . . . . ?
C16 C15 C14 C13 56.7(3) . . . . ?
C18 C13 C14 C15 -55.8(3) . . . . ?
P1 C13 C14 C15 179.09(18) . . . . ?
C18 C17 C16 C15 56.0(3) . . . . ?
C14 C15 C16 C17 -56.8(3) . . . . ?

_diffrn_measured_fraction_theta_max    0.980
_diffrn_reflns_theta_full              30.50
_diffrn_measured_fraction_theta_full   0.993
_refine_diff_density_max    1.232
_refine_diff_density_min   -0.617
_refine_diff_density_rms    0.154


data_bath1005

_audit_creation_method            SHELXL-97
_chemical_name_systematic
;
Bis(tricyclohexanephosphine)bis(n1-nitro)nickel(II)
;
_chemical_name_common             ?
_chemical_melting_point           ?
_chemical_formula_moiety          'C36 H66 N2 Ni O4 P2'
_chemical_formula_sum             'C36 H66 N2 Ni O4 P2'
_chemical_formula_weight          711.56

loop_
 _atom_type_symbol
 _atom_type_description
 _atom_type_scat_dispersion_real
 _atom_type_scat_dispersion_imag
 _atom_type_scat_source
 'C'  'C'   0.0033   0.0016
 'International Tables Vol C Tables 4.2.6.8 and 6.1.1.4'
 'H'  'H'   0.0000   0.0000
 'International Tables Vol C Tables 4.2.6.8 and 6.1.1.4'
 'N'  'N'   0.0061   0.0033
 'International Tables Vol C Tables 4.2.6.8 and 6.1.1.4'
 'O'  'O'   0.0106   0.0060
 'International Tables Vol C Tables 4.2.6.8 and 6.1.1.4'
 'P'  'P'   0.1023   0.0942
 'International Tables Vol C Tables 4.2.6.8 and 6.1.1.4'
 'Ni'  'Ni'   0.3393   1.1124
 'International Tables Vol C Tables 4.2.6.8 and 6.1.1.4'

_symmetry_cell_setting            triclinic
_symmetry_space_group_name_H-M    P-1

loop_
 _symmetry_equiv_pos_as_xyz
 'x, y, z'
 '-x, -y, -z'
```

```
_cell_length_a                    10.005(4)
_cell_length_b                    10.344(4)
_cell_length_c                    10.713(4)
_cell_angle_alpha                 112.515(6)
_cell_angle_beta                  110.465(6)
_cell_angle_gamma                 93.518(6)
_cell_volume                      934.9(7)
_cell_formula_units_Z             1
_cell_measurement_temperature     100(2)
_cell_measurement_reflns_used     3795
_cell_measurement_theta_min       2.96
_cell_measurement_theta_max       33.65

_exptl_crystal_description        block
_exptl_crystal_colour             orange
_exptl_crystal_size_max           0.04
_exptl_crystal_size_mid           0.03
_exptl_crystal_size_min           0.03
_exptl_crystal_density_meas       ?
_exptl_crystal_density_diffrn     1.264
_exptl_crystal_density_method     'not measured'
_exptl_crystal_F_000              386
_exptl_absorpt_coefficient_mu     0.643
_exptl_absorpt_correction_T_min   0.5457
_exptl_absorpt_correction_T_max   0.7466
_exptl_absorpt_correction_type    'multi-scan'
_exptl_absorpt_process_details    'SORTAV (Blessing, 1995)'

_exptl_special_details
;
multi-scan from symmetry-related measurements
 Sortav (Blessing 1995)
;

_diffrn_ambient_temperature       100(2)
_diffrn_radiation_wavelength      0.77490
_diffrn_radiation_type            Synchrotron
_diffrn_radiation_source          'Station 11.3.1 ALS'
_diffrn_radiation_monochromator   'Channel-cut Si(111)'
_diffrn_measurement_device_type   'Bruker APEX2'
_diffrn_measurement_method        '3000 0.3 degree images with \f and
\w scans'
_diffrn_detector_area_resol_mean  ?
_diffrn_standards_number          ?
_diffrn_standards_interval_count  ?
_diffrn_standards_interval_time   ?
_diffrn_standards_decay_%         0
_diffrn_reflns_number             13698
_diffrn_reflns_av_R_equivalents   0.0531
_diffrn_reflns_av_sigmaI/netI     0.0743
_diffrn_reflns_limit_h_min        -14
_diffrn_reflns_limit_h_max        14
_diffrn_reflns_limit_k_min        -14
_diffrn_reflns_limit_k_max        14
_diffrn_reflns_limit_l_min        -15
_diffrn_reflns_limit_l_max        15
```

```
_diffrn_reflns_theta_min          3.00
_diffrn_reflns_theta_max          33.81
_reflns_number_total              5619
_reflns_number_gt                 3617
_reflns_threshold_expression      >2sigma(I)

_computing_data_collection      'Bruker SMART'
_computing_cell_refinement      'Bruker SMART'
_computing_data_reduction       'Bruker SAINT'
_computing_structure_solution   'SHELXTS (Sheldrick, 1997)'
_computing_structure_refinement 'SHELXTL (Sheldrick, 1997)'
_computing_molecular_graphics   'POVRAY'
_computing_publication_material 'SHELXTL (Sheldrick, 1997)'

_refine_special_details
;
 Refinement of F^2^ against ALL reflections.  The weighted R-factor wR
and
 goodness of fit S are based on F^2^, conventional R-factors R are
based
 on F, with F set to zero for negative F^2^. The threshold expression
of
 F^2^ > 2sigma(F^2^) is used only for calculating R-factors(gt) etc.
and is
 not relevant to the choice of reflections for refinement.  R-factors
based
 on F^2^ are statistically about twice as large as those based on F,
and R-
 factors based on ALL data will be even larger.
;

_refine_ls_structure_factor_coef  Fsqd
_refine_ls_matrix_type            full
_refine_ls_weighting_scheme       calc
_refine_ls_weighting_details
 'calc w=1/[\s^2^(Fo^2^)+(0.1014P)^2^+0.0000P] where
P=(Fo^2^+2Fc^2^)/3'
_atom_sites_solution_primary      direct
_atom_sites_solution_secondary    difmap
_atom_sites_solution_hydrogens    geom
_refine_ls_hydrogen_treatment     constr
_refine_ls_extinction_method      none
_refine_ls_extinction_coef        ?
_refine_ls_number_reflns          5619
_refine_ls_number_parameters      217
_refine_ls_number_restraints      13
_refine_ls_R_factor_all           0.0988
_refine_ls_R_factor_gt            0.0596
_refine_ls_wR_factor_ref          0.1726
_refine_ls_wR_factor_gt           0.1524
_refine_ls_goodness_of_fit_ref    0.970
_refine_ls_restrained_S_all       0.975
_refine_ls_shift/su_max           0.000
_refine_ls_shift/su_mean          0.000

loop_
 _atom_site_label
```

```
 _atom_site_type_symbol
 _atom_site_fract_x
 _atom_site_fract_y
 _atom_site_fract_z
 _atom_site_U_iso_or_equiv
 _atom_site_adp_type
 _atom_site_occupancy
 _atom_site_symmetry_multiplicity
 _atom_site_calc_flag
 _atom_site_refinement_flags
 _atom_site_disorder_assembly
 _atom_site_disorder_group
Ni1 Ni 0.5000 0.5000 0.5000 0.02637(16) Uani 1 2 d SD . .
P1 P 0.29777(7) 0.46911(7) 0.29610(8) 0.02415(17) Uani 1 1 d . A .
C1 C 0.1288(3) 0.3573(3) 0.2714(3) 0.0261(5) Uani 1 1 d . . .
H1 H 0.1331 0.3860 0.3733 0.031 Uiso 1 1 calc R A .
C2 C 0.1262(3) 0.1952(3) 0.2110(3) 0.0306(6) Uani 1 1 d . A .
H2A H 0.1128 0.1574 0.1064 0.037 Uiso 1 1 calc R . .
H2B H 0.2206 0.1800 0.2681 0.037 Uiso 1 1 calc R . .
C3 C 0.0007(3) 0.1143(3) 0.2229(4) 0.0354(7) Uani 1 1 d . . .
H3A H -0.0012 0.0103 0.1820 0.042 Uiso 1 1 calc R A .
H3B H 0.0179 0.1479 0.3282 0.042 Uiso 1 1 calc R . .
C4 C -0.1465(3) 0.1382(4) 0.1393(4) 0.0392(7) Uani 1 1 d . A .
H4A H -0.1686 0.0955 0.0324 0.047 Uiso 1 1 calc R . .
H4B H -0.2246 0.0897 0.1536 0.047 Uiso 1 1 calc R . .
C5 C -0.1442(3) 0.2965(4) 0.1934(4) 0.0398(7) Uani 1 1 d . . .
H5A H -0.1335 0.3363 0.2973 0.048 Uiso 1 1 calc R A .
H5B H -0.2387 0.3095 0.1336 0.048 Uiso 1 1 calc R . .
C6 C -0.0190(3) 0.3799(3) 0.1838(4) 0.0337(7) Uani 1 1 d . A .
H6A H -0.0188 0.4835 0.2241 0.040 Uiso 1 1 calc R . .
H6B H -0.0338 0.3465 0.0791 0.040 Uiso 1 1 calc R . .
C7 C 0.3306(3) 0.3965(3) 0.1255(3) 0.0270(5) Uani 1 1 d . . .
H7 H 0.4096 0.4722 0.1407 0.032 Uiso 1 1 calc R A .
C8 C 0.3936(3) 0.2619(3) 0.1007(3) 0.0303(6) Uani 1 1 d . A .
H8A H 0.3188 0.1798 0.0784 0.036 Uiso 1 1 calc R . .
H8B H 0.4791 0.2783 0.1914 0.036 Uiso 1 1 calc R . .
C9 C 0.4403(3) 0.2275(4) -0.0286(3) 0.0349(7) Uani 1 1 d . . .
H9A H 0.5223 0.3055 -0.0011 0.042 Uiso 1 1 calc R A .
H9B H 0.4757 0.1375 -0.0477 0.042 Uiso 1 1 calc R . .
C10 C 0.3142(3) 0.2106(3) -0.1700(3) 0.0329(6) Uani 1 1 d . A .
H10A H 0.3506 0.1951 -0.2483 0.040 Uiso 1 1 calc R . .
H10B H 0.2368 0.1253 -0.2046 0.040 Uiso 1 1 calc R . .
C11 C 0.2501(3) 0.3435(3) -0.1425(3) 0.0339(6) Uani 1 1 d . . .
H11A H 0.3245 0.4267 -0.1186 0.041 Uiso 1 1 calc R A .
H11B H 0.1651 0.3278 -0.2334 0.041 Uiso 1 1 calc R . .
C12 C 0.2015(3) 0.3766(3) -0.0160(3) 0.0301(6) Uani 1 1 d . A .
H12A H 0.1214 0.2968 -0.0429 0.036 Uiso 1 1 calc R . .
H12B H 0.1636 0.4652 0.0019 0.036 Uiso 1 1 calc R . .
C13 C 0.2448(3) 0.6408(3) 0.3064(3) 0.0268(5) Uani 1 1 d . . .
H13 H 0.1444 0.6151 0.2269 0.032 Uiso 1 1 calc R A .
C14 C 0.3432(3) 0.7398(3) 0.2811(3) 0.0310(6) Uani 1 1 d . A .
H14A H 0.4446 0.7661 0.3564 0.037 Uiso 1 1 calc R . .
H14B H 0.3447 0.6881 0.1825 0.037 Uiso 1 1 calc R . .
C15 C 0.2872(4) 0.8756(3) 0.2906(3) 0.0350(7) Uani 1 1 d . . .
H15A H 0.3544 0.9395 0.2784 0.042 Uiso 1 1 calc R A .
H15B H 0.1896 0.8496 0.2092 0.042 Uiso 1 1 calc R . .
C16 C 0.2761(3) 0.9553(3) 0.4374(3) 0.0341(6) Uani 1 1 d . A .
```

```
H16A H 0.2358 1.0405 0.4383 0.041 Uiso 1 1 calc R . .
H16B H 0.3750 0.9890 0.5185 0.041 Uiso 1 1 calc R . .
C17 C 0.1780(4) 0.8586(3) 0.4627(4) 0.0375(7) Uani 1 1 d . . .
H17A H 0.0768 0.8327 0.3872 0.045 Uiso 1 1 calc R A .
H17B H 0.1763 0.9112 0.5611 0.045 Uiso 1 1 calc R . .
C18 C 0.2325(4) 0.7211(3) 0.4544(4) 0.0334(6) Uani 1 1 d . A .
H18A H 0.3295 0.7463 0.5365 0.040 Uiso 1 1 calc R . .
H18B H 0.1637 0.6577 0.4656 0.040 Uiso 1 1 calc R . .
O1 O 0.3478(13) 0.3086(10) 0.5450(14) 0.030(3) Uiso 0.168(3) 1 d PD A
1
O2 O 0.3238(11) 0.5238(10) 0.6485(11) 0.009(2) Uiso 0.168(3) 1 d PD A
1
N1 N 0.3779(13) 0.4361(10) 0.5765(14) 0.023(3) Uiso 0.168(3) 1 d PD A
1
O1A O 0.4079(3) 0.3460(3) 0.5152(3) 0.0287(9) Uiso 0.628(6) 1 d PD A 2
O2A O 0.3171(5) 0.4842(5) 0.6421(5) 0.0430(13) Uiso 0.628(6) 1 d PD A
2
N1A N 0.3271(5) 0.3659(6) 0.5889(5) 0.0309(11) Uiso 0.628(6) 1 d PD A
2
O1B O 0.364(2) 0.5313(18) 0.600(2) 0.101(7) Uiso 0.203(6) 1 d PD A 3
O2B O 0.233(3) 0.367(3) 0.601(4) 0.165(13) Uiso 0.203(6) 1 d PD A 3
N1B N 0.3476(14) 0.4121(16) 0.5997(15) 0.023(3) Uiso 0.203(6) 1 d PD A
3

loop_
 _atom_site_aniso_label
 _atom_site_aniso_U_11
 _atom_site_aniso_U_22
 _atom_site_aniso_U_33
 _atom_site_aniso_U_23
 _atom_site_aniso_U_13
 _atom_site_aniso_U_12
Ni1 0.0204(2) 0.0276(3) 0.0240(3) 0.0102(2) 0.00300(19) 0.00082(19)
P1 0.0199(3) 0.0220(3) 0.0236(3) 0.0079(3) 0.0039(3) 0.0013(2)
C1 0.0196(11) 0.0260(13) 0.0257(13) 0.0102(11) 0.0031(10) 0.0025(10)
C2 0.0252(13) 0.0269(14) 0.0352(15) 0.0129(12) 0.0085(11) 0.0025(11)
C3 0.0284(14) 0.0321(15) 0.0440(17) 0.0199(14) 0.0096(13) 0.0022(12)
C4 0.0243(13) 0.0379(17) 0.0501(19) 0.0213(16) 0.0081(13) -0.0010(12)
C5 0.0220(13) 0.0418(18) 0.053(2) 0.0231(16) 0.0100(13) 0.0057(12)
C6 0.0211(12) 0.0324(15) 0.0426(17) 0.0193(14) 0.0041(12) 0.0036(11)
C7 0.0234(12) 0.0263(13) 0.0238(13) 0.0082(11) 0.0047(10) 0.0017(10)
C8 0.0290(13) 0.0275(14) 0.0302(14) 0.0099(12) 0.0098(11) 0.0080(11)
C9 0.0289(14) 0.0341(16) 0.0351(16) 0.0093(13) 0.0120(12) 0.0062(12)
C10 0.0348(15) 0.0277(14) 0.0279(14) 0.0048(12) 0.0126(12) -0.0001(12)
C11 0.0349(15) 0.0324(15) 0.0262(14) 0.0083(12) 0.0090(12) 0.0025(12)
C12 0.0247(12) 0.0311(14) 0.0263(13) 0.0096(12) 0.0048(10) 0.0046(11)
C13 0.0254(12) 0.0213(12) 0.0264(13) 0.0077(11) 0.0057(10) 0.0033(10)
C14 0.0334(14) 0.0254(13) 0.0331(15) 0.0128(12) 0.0126(12) 0.0033(11)
C15 0.0376(15) 0.0282(14) 0.0336(15) 0.0153(13) 0.0066(12) 0.0047(12)
C16 0.0316(14) 0.0235(13) 0.0340(15) 0.0083(12) 0.0038(12) 0.0035(11)
C17 0.0369(16) 0.0258(14) 0.0414(17) 0.0060(13) 0.0160(14) 0.0075(12)
C18 0.0368(15) 0.0249(14) 0.0344(15) 0.0087(12) 0.0145(13) 0.0052(12)

_geom_special_details
;
 All esds (except the esd in the dihedral angle between two l.s.
planes)
```

are estimated using the full covariance matrix. The cell esds are taken
into account individually in the estimation of esds in distances, angles
and torsion angles; correlations between esds in cell parameters are only
used when they are defined by crystal symmetry. An approximate (isotropic)
treatment of cell esds is used for estimating esds involving l.s. planes.
;

```
loop_
 _geom_bond_atom_site_label_1
 _geom_bond_atom_site_label_2
 _geom_bond_distance
 _geom_bond_site_symmetry_2
 _geom_bond_publ_flag
Ni1 O1A 1.881(3) . ?
Ni1 O1A 1.881(3) 2_666 ?
Ni1 N1 1.901(8) 2_666 ?
Ni1 N1 1.901(8) . ?
Ni1 O1B 1.974(16) 2_666 ?
Ni1 O1B 1.974(16) . ?
Ni1 P1 2.2942(10) 2_666 ?
Ni1 P1 2.2942(10) . ?
P1 C7 1.847(3) . ?
P1 C13 1.858(3) . ?
P1 C1 1.865(3) . ?
C1 C6 1.537(4) . ?
C1 C2 1.543(4) . ?
C1 H1 1.0000 . ?
C2 C3 1.535(4) . ?
C2 H2A 0.9900 . ?
C2 H2B 0.9900 . ?
C3 C4 1.524(4) . ?
C3 H3A 0.9900 . ?
C3 H3B 0.9900 . ?
C4 C5 1.509(5) . ?
C4 H4A 0.9900 . ?
C4 H4B 0.9900 . ?
C5 C6 1.534(4) . ?
C5 H5A 0.9900 . ?
C5 H5B 0.9900 . ?
C6 H6A 0.9900 . ?
C6 H6B 0.9900 . ?
C7 C8 1.532(4) . ?
C7 C12 1.539(4) . ?
C7 H7 1.0000 . ?
C8 C9 1.536(4) . ?
C8 H8A 0.9900 . ?
C8 H8B 0.9900 . ?
C9 C10 1.533(4) . ?
C9 H9A 0.9900 . ?
C9 H9B 0.9900 . ?
C10 C11 1.523(4) . ?
C10 H10A 0.9900 . ?
```

```
C10 H10B 0.9900 . ?
C11 C12 1.522(4) . ?
C11 H11A 0.9900 . ?
C11 H11B 0.9900 . ?
C12 H12A 0.9900 . ?
C12 H12B 0.9900 . ?
C13 C18 1.536(4) . ?
C13 C14 1.539(4) . ?
C13 H13 1.0000 . ?
C14 C15 1.525(4) . ?
C14 H14A 0.9900 . ?
C14 H14B 0.9900 . ?
C15 C16 1.519(5) . ?
C15 H15A 0.9900 . ?
C15 H15B 0.9900 . ?
C16 C17 1.521(5) . ?
C16 H16A 0.9900 . ?
C16 H16B 0.9900 . ?
C17 C18 1.536(4) . ?
C17 H17A 0.9900 . ?
C17 H17B 0.9900 . ?
C18 H18A 0.9900 . ?
C18 H18B 0.9900 . ?
O1 N1 1.219(8) . ?
O2 N1 1.236(8) . ?
O1A N1A 1.289(5) . ?
O2A N1A 1.163(6) . ?
O1B N1B 1.234(9) . ?
O2B N1B 1.220(10) . ?

loop_
 _geom_angle_atom_site_label_1
 _geom_angle_atom_site_label_2
 _geom_angle_atom_site_label_3
 _geom_angle
 _geom_angle_site_symmetry_1
 _geom_angle_site_symmetry_3
 _geom_angle_publ_flag
O1A Ni1 O1A 180.00(11) . 2_666 ?
O1A Ni1 N1 147.9(3) . 2_666 ?
O1A Ni1 N1 32.1(3) 2_666 2_666 ?
O1A Ni1 N1 32.1(3) . . ?
O1A Ni1 N1 147.9(3) 2_666 . ?
N1 Ni1 N1 179.999(2) 2_666 . ?
O1A Ni1 O1B 119.9(5) . 2_666 ?
O1A Ni1 O1B 60.1(5) 2_666 2_666 ?
N1 Ni1 O1B 28.3(5) 2_666 2_666 ?
N1 Ni1 O1B 151.7(5) . 2_666 ?
O1A Ni1 O1B 60.1(5) . . ?
O1A Ni1 O1B 119.9(5) 2_666 . ?
N1 Ni1 O1B 151.7(5) 2_666 . ?
N1 Ni1 O1B 28.3(5) . . ?
O1B Ni1 O1B 180.0(6) 2_666 . ?
O1A Ni1 P1 88.68(10) . 2_666 ?
O1A Ni1 P1 91.32(10) 2_666 2_666 ?
N1 Ni1 P1 90.3(4) 2_666 2_666 ?
N1 Ni1 P1 89.7(4) . 2_666 ?
```

O1B Ni1 P1 84.3(7) 2_666 2_666 ?
O1B Ni1 P1 95.7(7) . 2_666 ?
O1A Ni1 P1 91.32(10) . . ?
O1A Ni1 P1 88.68(10) 2_666 . ?
N1 Ni1 P1 89.7(4) 2_666 . ?
N1 Ni1 P1 90.3(4) . . ?
O1B Ni1 P1 95.7(7) 2_666 . ?
O1B Ni1 P1 84.3(7) . . ?
P1 Ni1 P1 180.0 2_666 . ?
C7 P1 C13 104.29(13) . . ?
C7 P1 C1 110.26(13) . . ?
C13 P1 C1 103.27(13) . . ?
C7 P1 Ni1 111.99(9) . . ?
C13 P1 Ni1 112.72(9) . . ?
C1 P1 Ni1 113.60(9) . . ?
C6 C1 C2 109.3(2) . . ?
C6 C1 P1 117.3(2) . . ?
C2 C1 P1 113.31(19) . . ?
C6 C1 H1 105.3 . . ?
C2 C1 H1 105.3 . . ?
P1 C1 H1 105.3 . . ?
C3 C2 C1 110.1(2) . . ?
C3 C2 H2A 109.6 . . ?
C1 C2 H2A 109.6 . . ?
C3 C2 H2B 109.6 . . ?
C1 C2 H2B 109.6 . . ?
H2A C2 H2B 108.1 . . ?
C4 C3 C2 111.2(3) . . ?
C4 C3 H3A 109.4 . . ?
C2 C3 H3A 109.4 . . ?
C4 C3 H3B 109.4 . . ?
C2 C3 H3B 109.4 . . ?
H3A C3 H3B 108.0 . . ?
C5 C4 C3 110.7(3) . . ?
C5 C4 H4A 109.5 . . ?
C3 C4 H4A 109.5 . . ?
C5 C4 H4B 109.5 . . ?
C3 C4 H4B 109.5 . . ?
H4A C4 H4B 108.1 . . ?
C4 C5 C6 112.2(3) . . ?
C4 C5 H5A 109.2 . . ?
C6 C5 H5A 109.2 . . ?
C4 C5 H5B 109.2 . . ?
C6 C5 H5B 109.2 . . ?
H5A C5 H5B 107.9 . . ?
C5 C6 C1 110.3(2) . . ?
C5 C6 H6A 109.6 . . ?
C1 C6 H6A 109.6 . . ?
C5 C6 H6B 109.6 . . ?
C1 C6 H6B 109.6 . . ?
H6A C6 H6B 108.1 . . ?
C8 C7 C12 110.9(2) . . ?
C8 C7 P1 114.6(2) . . ?
C12 C7 P1 115.3(2) . . ?
C8 C7 H7 104.9 . . ?
C12 C7 H7 104.9 . . ?
P1 C7 H7 104.9 . . ?

C7 C8 C9 109.5(2) . . ?
C7 C8 H8A 109.8 . . ?
C9 C8 H8A 109.8 . . ?
C7 C8 H8B 109.8 . . ?
C9 C8 H8B 109.8 . . ?
H8A C8 H8B 108.2 . . ?
C10 C9 C8 112.1(2) . . ?
C10 C9 H9A 109.2 . . ?
C8 C9 H9A 109.2 . . ?
C10 C9 H9B 109.2 . . ?
C8 C9 H9B 109.2 . . ?
H9A C9 H9B 107.9 . . ?
C11 C10 C9 110.8(2) . . ?
C11 C10 H10A 109.5 . . ?
C9 C10 H10A 109.5 . . ?
C11 C10 H10B 109.5 . . ?
C9 C10 H10B 109.5 . . ?
H10A C10 H10B 108.1 . . ?
C12 C11 C10 111.2(3) . . ?
C12 C11 H11A 109.4 . . ?
C10 C11 H11A 109.4 . . ?
C12 C11 H11B 109.4 . . ?
C10 C11 H11B 109.4 . . ?
H11A C11 H11B 108.0 . . ?
C11 C12 C7 110.4(2) . . ?
C11 C12 H12A 109.6 . . ?
C7 C12 H12A 109.6 . . ?
C11 C12 H12B 109.6 . . ?
C7 C12 H12B 109.6 . . ?
H12A C12 H12B 108.1 . . ?
C18 C13 C14 110.7(2) . . ?
C18 C13 P1 109.26(19) . . ?
C14 C13 P1 115.8(2) . . ?
C18 C13 H13 106.9 . . ?
C14 C13 H13 106.9 . . ?
P1 C13 H13 106.9 . . ?
C15 C14 C13 110.6(3) . . ?
C15 C14 H14A 109.5 . . ?
C13 C14 H14A 109.5 . . ?
C15 C14 H14B 109.5 . . ?
C13 C14 H14B 109.5 . . ?
H14A C14 H14B 108.1 . . ?
C16 C15 C14 111.6(2) . . ?
C16 C15 H15A 109.3 . . ?
C14 C15 H15A 109.3 . . ?
C16 C15 H15B 109.3 . . ?
C14 C15 H15B 109.3 . . ?
H15A C15 H15B 108.0 . . ?
C15 C16 C17 110.8(3) . . ?
C15 C16 H16A 109.5 . . ?
C17 C16 H16A 109.5 . . ?
C15 C16 H16B 109.5 . . ?
C17 C16 H16B 109.5 . . ?
H16A C16 H16B 108.1 . . ?
C16 C17 C18 111.1(3) . . ?
C16 C17 H17A 109.4 . . ?
C18 C17 H17A 109.4 . . ?

```
C16 C17 H17B 109.4 . . ?
C18 C17 H17B 109.4 . . ?
H17A C17 H17B 108.0 . . ?
C13 C18 C17 110.9(3) . . ?
C13 C18 H18A 109.5 . . ?
C17 C18 H18A 109.5 . . ?
C13 C18 H18B 109.5 . . ?
C17 C18 H18B 109.5 . . ?
H18A C18 H18B 108.0 . . ?
O1 N1 O2 121.9(9) . . ?
O1 N1 Ni1 119.0(8) . . ?
O2 N1 Ni1 118.9(8) . . ?
N1A O1A Ni1 121.0(3) . . ?
O2A N1A O1A 114.4(5) . . ?
N1B O1B Ni1 96.9(11) . . ?
O2B N1B O1B 117.0(12) . . ?

loop_
 _geom_torsion_atom_site_label_1
 _geom_torsion_atom_site_label_2
 _geom_torsion_atom_site_label_3
 _geom_torsion_atom_site_label_4
 _geom_torsion
 _geom_torsion_site_symmetry_1
 _geom_torsion_site_symmetry_2
 _geom_torsion_site_symmetry_3
 _geom_torsion_site_symmetry_4
 _geom_torsion_publ_flag
O1A Ni1 P1 C7 -109.22(14) . . . . ?
O1A Ni1 P1 C7 70.78(14) 2_666 . . . ?
N1 Ni1 P1 C7 38.7(3) 2_666 . . . ?
N1 Ni1 P1 C7 -141.3(3) . . . . ?
O1B Ni1 P1 C7 11.0(5) 2_666 . . . ?
O1B Ni1 P1 C7 -169.0(5) . . . . ?
P1 Ni1 P1 C7 -137(12) 2_666 . . . ?
O1A Ni1 P1 C13 133.54(14) . . . . ?
O1A Ni1 P1 C13 -46.46(14) 2_666 . . . ?
N1 Ni1 P1 C13 -78.5(3) 2_666 . . . ?
N1 Ni1 P1 C13 101.5(3) . . . . ?
O1B Ni1 P1 C13 -106.3(5) 2_666 . . . ?
O1B Ni1 P1 C13 73.7(5) . . . . ?
P1 Ni1 P1 C13 106(12) 2_666 . . . ?
O1A Ni1 P1 C1 16.50(14) . . . . ?
O1A Ni1 P1 C1 -163.50(14) 2_666 . . . ?
N1 Ni1 P1 C1 164.4(3) 2_666 . . . ?
N1 Ni1 P1 C1 -15.6(3) . . . . ?
O1B Ni1 P1 C1 136.7(5) 2_666 . . . ?
O1B Ni1 P1 C1 -43.3(5) . . . . ?
P1 Ni1 P1 C1 -11(12) 2_666 . . . ?
C7 P1 C1 C6 -78.1(3) . . . . ?
C13 P1 C1 C6 32.8(3) . . . . ?
Ni1 P1 C1 C6 155.2(2) . . . . ?
C7 P1 C1 C2 50.8(2) . . . . ?
C13 P1 C1 C2 161.7(2) . . . . ?
Ni1 P1 C1 C2 -75.9(2) . . . . ?
C6 C1 C2 C3 -58.5(3) . . . . ?
P1 C1 C2 C3 168.7(2) . . . . ?
```

C1 C2 C3 C4 57.9(4) . . . . ?
C2 C3 C4 C5 -55.9(4) . . . . ?
C3 C4 C5 C6 55.5(4) . . . . ?
C4 C5 C6 C1 -56.9(4) . . . . ?
C2 C1 C6 C5 57.5(3) . . . . ?
P1 C1 C6 C5 -171.7(2) . . . . ?
C13 P1 C7 C8 173.27(19) . . . . ?
C1 P1 C7 C8 -76.5(2) . . . . ?
Ni1 P1 C7 C8 51.1(2) . . . . ?
C13 P1 C7 C12 -56.2(2) . . . . ?
C1 P1 C7 C12 54.1(2) . . . . ?
Ni1 P1 C7 C12 -178.35(18) . . . . ?
C12 C7 C8 C9 57.0(3) . . . . ?
P1 C7 C8 C9 -170.39(19) . . . . ?
C7 C8 C9 C10 -55.8(3) . . . . ?
C8 C9 C10 C11 55.3(3) . . . . ?
C9 C10 C11 C12 -55.6(3) . . . . ?
C10 C11 C12 C7 57.2(3) . . . . ?
C8 C7 C12 C11 -58.3(3) . . . . ?
P1 C7 C12 C11 169.4(2) . . . . ?
C7 P1 C13 C18 -173.16(19) . . . . ?
C1 P1 C13 C18 71.5(2) . . . . ?
Ni1 P1 C13 C18 -51.5(2) . . . . ?
C7 P1 C13 C14 -47.4(2) . . . . ?
C1 P1 C13 C14 -162.6(2) . . . . ?
Ni1 P1 C13 C14 74.3(2) . . . . ?
C18 C13 C14 C15 -55.6(3) . . . . ?
P1 C13 C14 C15 179.3(2) . . . . ?
C13 C14 C15 C16 56.5(3) . . . . ?
C14 C15 C16 C17 -56.9(3) . . . . ?
C15 C16 C17 C18 56.3(3) . . . . ?
C14 C13 C18 C17 55.4(3) . . . . ?
P1 C13 C18 C17 -175.9(2) . . . . ?
C16 C17 C18 C13 -55.9(3) . . . . ?
O1A Ni1 N1 O1 -1.9(8) . . . . ?
O1A Ni1 N1 O1 178.1(8) 2_666 . . . ?
N1 Ni1 N1 O1 -59(10) 2_666 . . . ?
O1B Ni1 N1 O1 -12(2) 2_666 . . . ?
O1B Ni1 N1 O1 168(2) . . . . ?
P1 Ni1 N1 O1 -89.8(12) 2_666 . . . ?
P1 Ni1 N1 O1 90.2(12) . . . . ?
O1A Ni1 N1 O2 -177.1(17) . . . . ?
O1A Ni1 N1 O2 2.9(17) 2_666 . . . ?
N1 Ni1 N1 O2 126(9) 2_666 . . . ?
O1B Ni1 N1 O2 172.4(15) 2_666 . . . ?
O1B Ni1 N1 O2 -7.6(15) . . . . ?
P1 Ni1 N1 O2 95.0(11) 2_666 . . . ?
P1 Ni1 N1 O2 -85.0(11) . . . . ?
O1A Ni1 O1A N1A 60(16) 2_666 . . . ?
N1 Ni1 O1A N1A -179.4(8) 2_666 . . . ?
N1 Ni1 O1A N1A 0.6(8) . . . . ?
O1B Ni1 O1A N1A 174.9(8) 2_666 . . . ?
O1B Ni1 O1A N1A -5.1(8) . . . . ?
P1 Ni1 O1A N1A 92.2(3) 2_666 . . . ?
P1 Ni1 O1A N1A -87.8(3) . . . . ?
Ni1 O1A N1A O2A 0.4(6) . . . . ?
O1A Ni1 O1B N1B 8.0(10) . . . . ?

```
O1A Ni1 O1B N1B -172.0(10) 2_666 . . . ?
N1 Ni1 O1B N1B -178.4(12) 2_666 . . . ?
N1 Ni1 O1B N1B 1.6(12) . . . . ?
O1B Ni1 O1B N1B 25(19) 2_666 . . . ?
P1 Ni1 O1B N1B -77.2(13) 2_666 . . . ?
P1 Ni1 O1B N1B 102.8(13) . . . . ?
Ni1 O1B N1B O2B -153(2) . . . . ?

_diffrn_measured_fraction_theta_max    0.968
_diffrn_reflns_theta_full              30.50
_diffrn_measured_fraction_theta_full   0.992
_refine_diff_density_max    1.260
_refine_diff_density_min   -0.737
_refine_diff_density_rms    0.095


data_bath1012

_audit_creation_method            SHELXL-97
_chemical_name_systematic
;
Bis(tricyclohexanephosphine)bis(n1-nitro)palladium(II)
;
_chemical_name_common             ?
_chemical_melting_point           ?
_chemical_formula_moiety          'C36 H66 N2 O4 P2 Pd'
_chemical_formula_sum             'C36 H66 N2 O4 P2 Pd'
_chemical_formula_weight          759.25

loop_
 _atom_type_symbol
 _atom_type_description
 _atom_type_scat_dispersion_real
 _atom_type_scat_dispersion_imag
 _atom_type_scat_source
 'C'  'C'   0.0033   0.0016
 'International Tables Vol C Tables 4.2.6.8 and 6.1.1.4'
 'H'  'H'   0.0000   0.0000
 'International Tables Vol C Tables 4.2.6.8 and 6.1.1.4'
 'N'  'N'   0.0061   0.0033
 'International Tables Vol C Tables 4.2.6.8 and 6.1.1.4'
 'O'  'O'   0.0106   0.0060
 'International Tables Vol C Tables 4.2.6.8 and 6.1.1.4'
 'P'  'P'   0.1023   0.0942
 'International Tables Vol C Tables 4.2.6.8 and 6.1.1.4'
 'Pd'  'Pd'  -0.9988   1.0072
 'International Tables Vol C Tables 4.2.6.8 and 6.1.1.4'

_symmetry_cell_setting            Triclinic
_symmetry_space_group_name_H-M    P-1

loop_
 _symmetry_equiv_pos_as_xyz
 'x, y, z'
 '-x, -y, -z'

_cell_length_a                    10.103(5)
```

```
_cell_length_b                    10.412(5)
_cell_length_c                    10.582(5)
_cell_angle_alpha                 111.425(5)
_cell_angle_beta                  110.355(5)
_cell_angle_gamma                 94.508(5)
_cell_volume                      944.2(8)
_cell_formula_units_Z             1
_cell_measurement_temperature     100(2)
_cell_measurement_reflns_used     3759
_cell_measurement_theta_min       2.95
_cell_measurement_theta_max       33.60

_exptl_crystal_description        block
_exptl_crystal_colour             colourless
_exptl_crystal_size_max           0.08
_exptl_crystal_size_mid           0.08
_exptl_crystal_size_min           0.07
_exptl_crystal_density_meas       ?
_exptl_crystal_density_diffrn     1.335
_exptl_crystal_density_method     'not measured'
_exptl_crystal_F_000              404
_exptl_absorpt_coefficient_mu     0.615
_exptl_absorpt_correction_T_min   0.5457
_exptl_absorpt_correction_T_max   0.7466
_exptl_absorpt_correction_type    'multi-scan'
_exptl_absorpt_process_details    'SORTAV (Blessing, 1995)'

_exptl_special_details
;
multi-scan from symmetry-related measurements
 Sortav (Blessing 1995)
;

_diffrn_ambient_temperature       100(2)
_diffrn_radiation_wavelength      0.77490
_diffrn_radiation_type            Synchrotron
_diffrn_radiation_source          'Station 11.3.1 ALS'
_diffrn_radiation_monochromator   'Channel-cut Si(111)'
_diffrn_measurement_device_type   'Bruker APEX2'
_diffrn_measurement_method        '3000 0.3 degree images with \f and
\w scans'
_diffrn_detector_area_resol_mean  ?
_diffrn_standards_number          ?
_diffrn_standards_interval_count  ?
_diffrn_standards_interval_time   ?
_diffrn_standards_decay_%         0
_diffrn_reflns_number             7454
_diffrn_reflns_av_R_equivalents   0.0545
_diffrn_reflns_av_sigmaI/netI     0.0877
_diffrn_reflns_limit_h_min        -14
_diffrn_reflns_limit_h_max        14
_diffrn_reflns_limit_k_min        -14
_diffrn_reflns_limit_k_max        13
_diffrn_reflns_limit_l_min        -15
_diffrn_reflns_limit_l_max        15
_diffrn_reflns_theta_min          2.96
_diffrn_reflns_theta_max          30.50
```

```
_reflns_number_total              5002
_reflns_number_gt                 4605
_reflns_threshold_expression      >2sigma(I)

_computing_data_collection      'Bruker SMART'
_computing_cell_refinement      'Bruker SMART'
_computing_data_reduction       'Bruker SAINT'
_computing_structure_solution   'SHELXTS (Sheldrick, 1997)'
_computing_structure_refinement 'SHELXTL (Sheldrick, 1997)'
_computing_molecular_graphics   'ORTEP'
_computing_publication_material 'SHELXTL (Sheldrick, 1997)'

_refine_special_details
;
 Refinement of F^2^ against ALL reflections.  The weighted R-factor wR
and
 goodness of fit S are based on F^2^, conventional R-factors R are
based
 on F, with F set to zero for negative F^2^. The threshold expression
of
 F^2^ > 2sigma(F^2^) is used only for calculating R-factors(gt) etc.
and is
 not relevant to the choice of reflections for refinement.  R-factors
based
 on F^2^ are statistically about twice as large as those based on F,
and R-
 factors based on ALL data will be even larger.
;

_refine_ls_structure_factor_coef  Fsqd
_refine_ls_matrix_type            full
_refine_ls_weighting_scheme       calc
_refine_ls_weighting_details
 'calc w=1/[\s^2^(Fo^2^)+(0.1136P)^2^+0.0000P] where
P=(Fo^2^+2Fc^2^)/3'
_atom_sites_solution_primary      direct
_atom_sites_solution_secondary    difmap
_atom_sites_solution_hydrogens    geom
_refine_ls_hydrogen_treatment     constr
_refine_ls_extinction_method      none
_refine_ls_extinction_coef        ?
_refine_ls_number_reflns          5002
_refine_ls_number_parameters      205
_refine_ls_number_restraints      0
_refine_ls_R_factor_all           0.0591
_refine_ls_R_factor_gt            0.0559
_refine_ls_wR_factor_ref          0.1655
_refine_ls_wR_factor_gt           0.1620
_refine_ls_goodness_of_fit_ref    1.045
_refine_ls_restrained_S_all       1.045
_refine_ls_shift/su_max           0.000
_refine_ls_shift/su_mean          0.000

loop_
 _atom_site_label
 _atom_site_type_symbol
 _atom_site_fract_x
```

```
 _atom_site_fract_y
 _atom_site_fract_z
 _atom_site_U_iso_or_equiv
 _atom_site_adp_type
 _atom_site_occupancy
 _atom_site_symmetry_multiplicity
 _atom_site_calc_flag
 _atom_site_refinement_flags
 _atom_site_disorder_assembly
 _atom_site_disorder_group
Pd1 Pd 0.5000 0.5000 1.0000 0.00954(12) Uani 1 2 d S . .
P1 P 0.70951(8) 0.53522(8) 1.21331(8) 0.01052(17) Uani 1 1 d . . .
O1 O 0.6661(3) 0.6929(3) 0.9475(3) 0.0201(5) Uani 1 1 d . . .
O2 O 0.6840(3) 0.4787(3) 0.8459(3) 0.0217(5) Uani 1 1 d . . .
N1 N 0.6323(3) 0.5648(3) 0.9180(3) 0.0142(5) Uani 1 1 d . . .
C1 C 0.8751(3) 0.6478(3) 1.2334(3) 0.0125(5) Uani 1 1 d . . .
H13 H 0.8709 0.6179 1.1306 0.015 Uiso 1 1 calc R . .
C2 C 0.8737(3) 0.8069(3) 1.2887(3) 0.0143(5) Uani 1 1 d . . .
H14A H 0.7789 0.8183 1.2293 0.017 Uiso 1 1 calc R . .
H14B H 0.8865 0.8451 1.3938 0.017 Uiso 1 1 calc R . .
C3 C 0.9960(3) 0.8899(4) 1.2747(4) 0.0184(6) Uani 1 1 d . . .
H15A H 0.9953 0.9919 1.3122 0.022 Uiso 1 1 calc R . .
H15B H 0.9799 0.8555 1.1688 0.022 Uiso 1 1 calc R . .
C4 C 1.1444(3) 0.8714(4) 1.3638(4) 0.0200(6) Uani 1 1 d . . .
H16A H 1.2216 0.9206 1.3487 0.024 Uiso 1 1 calc R . .
H16B H 1.1651 0.9149 1.4710 0.024 Uiso 1 1 calc R . .
C5 C 1.1453(3) 0.7149(4) 1.3149(4) 0.0184(6) Uani 1 1 d . . .
H17A H 1.2396 0.7055 1.3776 0.022 Uiso 1 1 calc R . .
H17B H 1.1359 0.6746 1.2109 0.022 Uiso 1 1 calc R . .
C6 C 1.0228(3) 0.6302(3) 1.3259(3) 0.0154(5) Uani 1 1 d . . .
H18A H 1.0363 0.6642 1.4310 0.019 Uiso 1 1 calc R . .
H18B H 1.0248 0.5286 1.2888 0.019 Uiso 1 1 calc R . .
C7 C 0.7607(3) 0.3654(3) 1.2029(3) 0.0128(5) Uani 1 1 d . . .
H7 H 0.8579 0.3895 1.2861 0.015 Uiso 1 1 calc R . .
C8 C 0.7789(4) 0.2899(3) 1.0559(3) 0.0165(6) Uani 1 1 d . . .
H8A H 0.6855 0.2683 0.9706 0.020 Uiso 1 1 calc R . .
H8B H 0.8523 0.3537 1.0500 0.020 Uiso 1 1 calc R . .
C9 C 0.8269(4) 0.1514(3) 1.0474(4) 0.0200(6) Uani 1 1 d . . .
H9A H 0.8326 0.1021 0.9504 0.024 Uiso 1 1 calc R . .
H9B H 0.9251 0.1743 1.1265 0.024 Uiso 1 1 calc R . .
C10 C 0.7221(4) 0.0533(4) 1.0652(4) 0.0206(6) Uani 1 1 d . . .
H10A H 0.7584 -0.0326 1.0638 0.025 Uiso 1 1 calc R . .
H10B H 0.6260 0.0231 0.9811 0.025 Uiso 1 1 calc R . .
C11 C 0.7060(4) 0.1288(3) 1.2117(3) 0.0182(6) Uani 1 1 d . . .
H11A H 0.8004 0.1512 1.2960 0.022 Uiso 1 1 calc R . .
H11B H 0.6344 0.0647 1.2193 0.022 Uiso 1 1 calc R . .
C12 C 0.6561(4) 0.2667(3) 1.2215(4) 0.0181(6) Uani 1 1 d . . .
H12A H 0.5575 0.2435 1.1431 0.022 Uiso 1 1 calc R . .
H12B H 0.6513 0.3154 1.3190 0.022 Uiso 1 1 calc R . .
C13 C 0.6751(3) 0.6053(3) 1.3836(3) 0.0128(5) Uani 1 1 d . . .
H1 H 0.5955 0.5306 1.3685 0.015 Uiso 1 1 calc R . .
C14 C 0.8021(3) 0.6259(3) 1.5280(3) 0.0157(6) Uani 1 1 d . . .
H2A H 0.8401 0.5392 1.5120 0.019 Uiso 1 1 calc R . .
H2B H 0.8818 0.7060 1.5556 0.019 Uiso 1 1 calc R . .
C15 C 0.7503(4) 0.6567(4) 1.6541(3) 0.0186(6) Uani 1 1 d . . .
H3A H 0.6745 0.5743 1.6288 0.022 Uiso 1 1 calc R . .
H3B H 0.8327 0.6713 1.7467 0.022 Uiso 1 1 calc R . .
```

```
C16 C 0.6888(4) 0.7888(4) 1.6788(3) 0.0182(6) Uani 1 1 d . . .
H4A H 0.7671 0.8727 1.7130 0.022 Uiso 1 1 calc R . .
H4B H 0.6519 0.8046 1.7572 0.022 Uiso 1 1 calc R . .
C17 C 0.5647(4) 0.7729(4) 1.5347(3) 0.0180(6) Uani 1 1 d . . .
H5A H 0.4824 0.6950 1.5060 0.022 Uiso 1 1 calc R . .
H5B H 0.5302 0.8617 1.5526 0.022 Uiso 1 1 calc R . .
C18 C 0.6151(3) 0.7410(3) 1.4073(3) 0.0139(5) Uani 1 1 d . . .
H6A H 0.6917 0.8223 1.4318 0.017 Uiso 1 1 calc R . .
H6B H 0.5325 0.7267 1.3150 0.017 Uiso 1 1 calc R . .

loop_
 _atom_site_aniso_label
 _atom_site_aniso_U_11
 _atom_site_aniso_U_22
 _atom_site_aniso_U_33
 _atom_site_aniso_U_23
 _atom_site_aniso_U_13
 _atom_site_aniso_U_12
Pd1 0.00978(19) 0.01059(18) 0.00861(16) 0.00440(12) 0.00350(13)
0.00342(13)
P1 0.0107(4) 0.0107(4) 0.0099(3) 0.0045(3) 0.0033(3) 0.0038(3)
O1 0.0200(12) 0.0191(12) 0.0215(10) 0.0113(9) 0.0063(10) 0.0017(10)
O2 0.0223(12) 0.0261(13) 0.0223(10) 0.0099(10) 0.0145(10) 0.0101(10)
N1 0.0113(12) 0.0195(13) 0.0117(9) 0.0069(10) 0.0039(10) 0.0047(10)
C1 0.0133(14) 0.0119(13) 0.0125(11) 0.0054(10) 0.0049(11) 0.0037(11)
C2 0.0119(14) 0.0111(13) 0.0182(12) 0.0050(11) 0.0054(11) 0.0025(11)
C3 0.0152(15) 0.0167(15) 0.0254(14) 0.0108(13) 0.0086(13) 0.0041(12)
C4 0.0135(15) 0.0171(15) 0.0278(15) 0.0096(13) 0.0067(13) 0.0021(13)
C5 0.0130(14) 0.0199(16) 0.0226(13) 0.0080(12) 0.0081(12) 0.0056(12)
C6 0.0104(14) 0.0154(14) 0.0189(12) 0.0085(11) 0.0028(11) 0.0033(12)
C7 0.0132(14) 0.0106(13) 0.0124(10) 0.0044(10) 0.0032(11) 0.0023(11)
C8 0.0201(16) 0.0152(14) 0.0149(11) 0.0057(11) 0.0080(12) 0.0060(12)
C9 0.0217(17) 0.0120(14) 0.0219(13) 0.0026(12) 0.0086(13) 0.0049(13)
C10 0.0226(17) 0.0120(14) 0.0230(14) 0.0066(12) 0.0055(13) 0.0048(13)
C11 0.0200(16) 0.0118(14) 0.0224(13) 0.0092(12) 0.0059(13) 0.0046(12)
C12 0.0186(16) 0.0143(14) 0.0229(13) 0.0090(12) 0.0084(13) 0.0052(12)
C13 0.0138(14) 0.0148(14) 0.0119(11) 0.0068(10) 0.0057(11) 0.0065(11)
C14 0.0180(15) 0.0173(15) 0.0140(11) 0.0081(11) 0.0066(12) 0.0082(12)
C15 0.0213(16) 0.0201(16) 0.0126(11) 0.0058(11) 0.0061(12) 0.0038(13)
C16 0.0214(16) 0.0176(15) 0.0151(12) 0.0050(11) 0.0093(12) 0.0033(13)
C17 0.0184(16) 0.0176(15) 0.0191(12) 0.0058(12) 0.0106(13) 0.0062(12)
C18 0.0142(14) 0.0145(14) 0.0140(11) 0.0049(11) 0.0075(11) 0.0056(11)

_geom_special_details
;
 All esds (except the esd in the dihedral angle between two l.s.
planes)
 are estimated using the full covariance matrix.  The cell esds are
taken
 into account individually in the estimation of esds in distances,
angles
 and torsion angles; correlations between esds in cell parameters are
only
 used when they are defined by crystal symmetry.  An approximate
(isotropic)
 treatment of cell esds is used for estimating esds involving l.s.
planes.
```

```
;

loop_
 _geom_bond_atom_site_label_1
 _geom_bond_atom_site_label_2
 _geom_bond_distance
 _geom_bond_site_symmetry_2
 _geom_bond_publ_flag
Pd1 N1 2.022(3) 2_667 ?
Pd1 N1 2.022(3) . ?
Pd1 P1 2.3789(11) 2_667 ?
Pd1 P1 2.3789(11) . ?
P1 C13 1.848(3) . ?
P1 C7 1.857(3) . ?
P1 C1 1.865(3) . ?
O1 N1 1.242(4) . ?
O2 N1 1.228(3) . ?
C1 C6 1.541(4) . ?
C1 C2 1.546(4) . ?
C1 H13 1.0000 . ?
C2 C3 1.533(5) . ?
C2 H14A 0.9900 . ?
C2 H14B 0.9900 . ?
C3 C4 1.539(4) . ?
C3 H15A 0.9900 . ?
C3 H15B 0.9900 . ?
C4 C5 1.520(5) . ?
C4 H16A 0.9900 . ?
C4 H16B 0.9900 . ?
C5 C6 1.526(5) . ?
C5 H17A 0.9900 . ?
C5 H17B 0.9900 . ?
C6 H18A 0.9900 . ?
C6 H18B 0.9900 . ?
C7 C12 1.528(4) . ?
C7 C8 1.549(4) . ?
C7 H7 1.0000 . ?
C8 C9 1.540(4) . ?
C8 H8A 0.9900 . ?
C8 H8B 0.9900 . ?
C9 C10 1.520(5) . ?
C9 H9A 0.9900 . ?
C9 H9B 0.9900 . ?
C10 C11 1.534(5) . ?
C10 H10A 0.9900 . ?
C10 H10B 0.9900 . ?
C11 C12 1.539(4) . ?
C11 H11A 0.9900 . ?
C11 H11B 0.9900 . ?
C12 H12A 0.9900 . ?
C12 H12B 0.9900 . ?
C13 C14 1.543(4) . ?
C13 C18 1.552(4) . ?
C13 H1 1.0000 . ?
C14 C15 1.536(4) . ?
C14 H2A 0.9900 . ?
C14 H2B 0.9900 . ?
```

```
C15 C16 1.527(4) . ?
C15 H3A 0.9900 . ?
C15 H3B 0.9900 . ?
C16 C17 1.541(5) . ?
C16 H4A 0.9900 . ?
C16 H4B 0.9900 . ?
C17 C18 1.538(4) . ?
C17 H5A 0.9900 . ?
C17 H5B 0.9900 . ?
C18 H6A 0.9900 . ?
C18 H6B 0.9900 . ?

loop_
 _geom_angle_atom_site_label_1
 _geom_angle_atom_site_label_2
 _geom_angle_atom_site_label_3
 _geom_angle
 _geom_angle_site_symmetry_1
 _geom_angle_site_symmetry_3
 _geom_angle_publ_flag
N1 Pd1 N1 179.999(1) 2_667 . ?
N1 Pd1 P1 88.61(8) 2_667 2_667 ?
N1 Pd1 P1 91.39(8) . 2_667 ?
N1 Pd1 P1 91.39(8) 2_667 . ?
N1 Pd1 P1 88.61(8) . . ?
P1 Pd1 P1 180.00(4) 2_667 . ?
C13 P1 C7 105.05(13) . . ?
C13 P1 C1 111.40(14) . . ?
C7 P1 C1 103.96(13) . . ?
C13 P1 Pd1 111.22(10) . . ?
C7 P1 Pd1 111.58(10) . . ?
C1 P1 Pd1 113.11(9) . . ?
O2 N1 O1 120.7(3) . . ?
O2 N1 Pd1 119.8(2) . . ?
O1 N1 Pd1 119.32(19) . . ?
C6 C1 C2 109.6(3) . . ?
C6 C1 P1 116.7(2) . . ?
C2 C1 P1 112.58(19) . . ?
C6 C1 H13 105.7 . . ?
C2 C1 H13 105.7 . . ?
P1 C1 H13 105.7 . . ?
C3 C2 C1 110.2(2) . . ?
C3 C2 H14A 109.6 . . ?
C1 C2 H14A 109.6 . . ?
C3 C2 H14B 109.6 . . ?
C1 C2 H14B 109.6 . . ?
H14A C2 H14B 108.1 . . ?
C2 C3 C4 110.5(3) . . ?
C2 C3 H15A 109.5 . . ?
C4 C3 H15A 109.5 . . ?
C2 C3 H15B 109.5 . . ?
C4 C3 H15B 109.5 . . ?
H15A C3 H15B 108.1 . . ?
C5 C4 C3 110.6(3) . . ?
C5 C4 H16A 109.5 . . ?
C3 C4 H16A 109.5 . . ?
C5 C4 H16B 109.5 . . ?
```

C3 C4 H16B 109.5 . . ?
H16A C4 H16B 108.1 . . ?
C4 C5 C6 112.4(2) . . ?
C4 C5 H17A 109.1 . . ?
C6 C5 H17A 109.1 . . ?
C4 C5 H17B 109.1 . . ?
C6 C5 H17B 109.1 . . ?
H17A C5 H17B 107.8 . . ?
C5 C6 C1 110.0(2) . . ?
C5 C6 H18A 109.7 . . ?
C1 C6 H18A 109.7 . . ?
C5 C6 H18B 109.7 . . ?
C1 C6 H18B 109.7 . . ?
H18A C6 H18B 108.2 . . ?
C12 C7 C8 111.2(3) . . ?
C12 C7 P1 114.0(2) . . ?
C8 C7 P1 109.57(19) . . ?
C12 C7 H7 107.3 . . ?
C8 C7 H7 107.3 . . ?
P1 C7 H7 107.3 . . ?
C9 C8 C7 110.5(2) . . ?
C9 C8 H8A 109.5 . . ?
C7 C8 H8A 109.5 . . ?
C9 C8 H8B 109.5 . . ?
C7 C8 H8B 109.5 . . ?
H8A C8 H8B 108.1 . . ?
C10 C9 C8 111.6(3) . . ?
C10 C9 H9A 109.3 . . ?
C8 C9 H9A 109.3 . . ?
C10 C9 H9B 109.3 . . ?
C8 C9 H9B 109.3 . . ?
H9A C9 H9B 108.0 . . ?
C9 C10 C11 110.6(3) . . ?
C9 C10 H10A 109.5 . . ?
C11 C10 H10A 109.5 . . ?
C9 C10 H10B 109.5 . . ?
C11 C10 H10B 109.5 . . ?
H10A C10 H10B 108.1 . . ?
C10 C11 C12 111.2(2) . . ?
C10 C11 H11A 109.4 . . ?
C12 C11 H11A 109.4 . . ?
C10 C11 H11B 109.4 . . ?
C12 C11 H11B 109.4 . . ?
H11A C11 H11B 108.0 . . ?
C7 C12 C11 110.8(3) . . ?
C7 C12 H12A 109.5 . . ?
C11 C12 H12A 109.5 . . ?
C7 C12 H12B 109.5 . . ?
C11 C12 H12B 109.5 . . ?
H12A C12 H12B 108.1 . . ?
C14 C13 C18 110.1(2) . . ?
C14 C13 P1 115.51(19) . . ?
C18 C13 P1 114.30(18) . . ?
C14 C13 H1 105.3 . . ?
C18 C13 H1 105.3 . . ?
P1 C13 H1 105.3 . . ?
C15 C14 C13 110.0(2) . . ?

```
C15 C14 H2A 109.7 . . ?
C13 C14 H2A 109.7 . . ?
C15 C14 H2B 109.7 . . ?
C13 C14 H2B 109.7 . . ?
H2A C14 H2B 108.2 . . ?
C16 C15 C14 110.5(3) . . ?
C16 C15 H3A 109.6 . . ?
C14 C15 H3A 109.6 . . ?
C16 C15 H3B 109.6 . . ?
C14 C15 H3B 109.6 . . ?
H3A C15 H3B 108.1 . . ?
C15 C16 C17 111.1(3) . . ?
C15 C16 H4A 109.4 . . ?
C17 C16 H4A 109.4 . . ?
C15 C16 H4B 109.4 . . ?
C17 C16 H4B 109.4 . . ?
H4A C16 H4B 108.0 . . ?
C18 C17 C16 111.3(3) . . ?
C18 C17 H5A 109.4 . . ?
C16 C17 H5A 109.4 . . ?
C18 C17 H5B 109.4 . . ?
C16 C17 H5B 109.4 . . ?
H5A C17 H5B 108.0 . . ?
C17 C18 C13 109.3(2) . . ?
C17 C18 H6A 109.8 . . ?
C13 C18 H6A 109.8 . . ?
C17 C18 H6B 109.8 . . ?
C13 C18 H6B 109.8 . . ?
H6A C18 H6B 108.3 . . ?

loop_
 _geom_torsion_atom_site_label_1
 _geom_torsion_atom_site_label_2
 _geom_torsion_atom_site_label_3
 _geom_torsion_atom_site_label_4
 _geom_torsion
 _geom_torsion_site_symmetry_1
 _geom_torsion_site_symmetry_2
 _geom_torsion_site_symmetry_3
 _geom_torsion_site_symmetry_4
 _geom_torsion_publ_flag
N1 Pd1 P1 C13 -36.96(13) 2_667 . . . ?
N1 Pd1 P1 C13 143.04(13) . . . . ?
P1 Pd1 P1 C13 -36(17) 2_667 . . . ?
N1 Pd1 P1 C7 79.98(13) 2_667 . . . ?
N1 Pd1 P1 C7 -100.02(13) . . . . ?
P1 Pd1 P1 C7 81(17) 2_667 . . . ?
N1 Pd1 P1 C1 -163.22(12) 2_667 . . . ?
N1 Pd1 P1 C1 16.78(12) . . . . ?
P1 Pd1 P1 C1 -162(17) 2_667 . . . ?
N1 Pd1 N1 O2 69(9) 2_667 . . . ?
P1 Pd1 N1 O2 -93.3(2) 2_667 . . . ?
P1 Pd1 N1 O2 86.7(2) . . . . ?
N1 Pd1 N1 O1 -107(9) 2_667 . . . ?
P1 Pd1 N1 O1 90.2(2) 2_667 . . . ?
P1 Pd1 N1 O1 -89.8(2) . . . . ?
C13 P1 C1 C6 77.0(2) . . . . ?
```

```
C7 P1 C1 C6 -35.6(2) . . . . ?
Pd1 P1 C1 C6 -156.80(18) . . . . ?
C13 P1 C1 C2 -50.9(2) . . . . ?
C7 P1 C1 C2 -163.54(19) . . . . ?
Pd1 P1 C1 C2 75.3(2) . . . . ?
C6 C1 C2 C3 59.2(3) . . . . ?
P1 C1 C2 C3 -169.16(19) . . . . ?
C1 C2 C3 C4 -58.2(3) . . . . ?
C2 C3 C4 C5 55.7(3) . . . . ?
C3 C4 C5 C6 -55.5(4) . . . . ?
C4 C5 C6 C1 56.8(3) . . . . ?
C2 C1 C6 C5 -57.8(3) . . . . ?
P1 C1 C6 C5 172.8(2) . . . . ?
C13 P1 C7 C12 49.6(2) . . . . ?
C1 P1 C7 C12 166.8(2) . . . . ?
Pd1 P1 C7 C12 -71.0(2) . . . . ?
C13 P1 C7 C8 175.0(2) . . . . ?
C1 P1 C7 C8 -67.9(2) . . . . ?
Pd1 P1 C7 C8 54.3(2) . . . . ?
C12 C7 C8 C9 -55.2(3) . . . . ?
P1 C7 C8 C9 177.9(2) . . . . ?
C7 C8 C9 C10 55.8(4) . . . . ?
C8 C9 C10 C11 -56.6(3) . . . . ?
C9 C10 C11 C12 56.7(4) . . . . ?
C8 C7 C12 C11 55.6(3) . . . . ?
P1 C7 C12 C11 -179.9(2) . . . . ?
C10 C11 C12 C7 -56.4(4) . . . . ?
C7 P1 C13 C14 56.6(3) . . . . ?
C1 P1 C13 C14 -55.4(3) . . . . ?
Pd1 P1 C13 C14 177.4(2) . . . . ?
C7 P1 C13 C18 -174.2(2) . . . . ?
C1 P1 C13 C18 73.9(2) . . . . ?
Pd1 P1 C13 C18 -53.3(2) . . . . ?
C18 C13 C14 C15 59.5(3) . . . . ?
P1 C13 C14 C15 -169.2(2) . . . . ?
C13 C14 C15 C16 -58.3(4) . . . . ?
C14 C15 C16 C17 56.5(4) . . . . ?
C15 C16 C17 C18 -56.3(4) . . . . ?
C16 C17 C18 C13 56.7(4) . . . . ?
C14 C13 C18 C17 -58.4(3) . . . . ?
P1 C13 C18 C17 169.6(2) . . . . ?

_diffrn_measured_fraction_theta_max    0.868
_diffrn_reflns_theta_full              30.00
_diffrn_measured_fraction_theta_full   0.875
_refine_diff_density_max    1.214
_refine_diff_density_min   -0.895
_refine_diff_density_rms    0.173


data_bath1016

_audit_creation_method            SHELXL-97
_chemical_name_systematic
;
Bis(tricyclohexanephosphine)bis(n1-nitro/n1-nitrito)palladium(II)
;
```

```
_chemical_name_common             ?
_chemical_melting_point           ?
_chemical_formula_moiety          'C36 H66 N2 O4 P2 Pd'
_chemical_formula_sum             'C36 H66 N2 O4 P2 Pd'
_chemical_formula_weight          759.25

loop_
 _atom_type_symbol
 _atom_type_description
 _atom_type_scat_dispersion_real
 _atom_type_scat_dispersion_imag
 _atom_type_scat_source
 'C'  'C'   0.0033   0.0016
 'International Tables Vol C Tables 4.2.6.8 and 6.1.1.4'
 'H'  'H'   0.0000   0.0000
 'International Tables Vol C Tables 4.2.6.8 and 6.1.1.4'
 'N'  'N'   0.0061   0.0033
 'International Tables Vol C Tables 4.2.6.8 and 6.1.1.4'
 'O'  'O'   0.0106   0.0060
 'International Tables Vol C Tables 4.2.6.8 and 6.1.1.4'
 'P'  'P'   0.1023   0.0942
 'International Tables Vol C Tables 4.2.6.8 and 6.1.1.4'
 'Pd'  'Pd'  -0.9988   1.0072
 'International Tables Vol C Tables 4.2.6.8 and 6.1.1.4'

_symmetry_cell_setting            Triclinic
_symmetry_space_group_name_H-M    P-1

loop_
 _symmetry_equiv_pos_as_xyz
 'x, y, z'
 '-x, -y, -z'

_cell_length_a                    10.0521(7)
_cell_length_b                    10.4185(7)
_cell_length_c                    10.6814(7)
_cell_angle_alpha                 112.0220(10)
_cell_angle_beta                  110.3450(10)
_cell_angle_gamma                 94.0300(10)
_cell_volume                      946.07(11)
_cell_formula_units_Z             1
_cell_measurement_temperature     100(2)
_cell_measurement_reflns_used    6569
_cell_measurement_theta_min        2.97
_cell_measurement_theta_max       34.45

_exptl_crystal_description        block
_exptl_crystal_colour             colourless
_exptl_crystal_size_max           0.08
_exptl_crystal_size_mid           0.08
_exptl_crystal_size_min           0.07
_exptl_crystal_density_meas       ?
_exptl_crystal_density_diffrn     1.333
_exptl_crystal_density_method     'not measured'
_exptl_crystal_F_000              404
_exptl_absorpt_coefficient_mu     0.614
_exptl_absorpt_correction_T_min   0.6156
```

```
_exptl_absorpt_correction_T_max   0.7469
_exptl_absorpt_correction_type    'multi-scan'
_exptl_absorpt_process_details    'SORTAV (Blessing, 1995)'

_exptl_special_details
;
multi-scan from symmetry-related measurements
 Sortav (Blessing 1995)
;

_diffrn_ambient_temperature       100(2)
_diffrn_radiation_wavelength      0.77490
_diffrn_radiation_type            Synchrotron
_diffrn_radiation_source          'Station 11.3.1 ALS'
_diffrn_radiation_monochromator   'Channel-cut Si(111)'
_diffrn_measurement_device_type   'Bruker APEX2'
_diffrn_measurement_method        '3000 0.3 degree images with \f and
\w scans'
_diffrn_detector_area_resol_mean  ?
_diffrn_standards_number          ?
_diffrn_standards_interval_count  ?
_diffrn_standards_interval_time   ?
_diffrn_standards_decay_%         0
_diffrn_reflns_number             14110
_diffrn_reflns_av_R_equivalents   0.0587
_diffrn_reflns_av_sigmaI/netI     0.0719
_diffrn_reflns_limit_h_min        -14
_diffrn_reflns_limit_h_max        14
_diffrn_reflns_limit_k_min        -15
_diffrn_reflns_limit_k_max        14
_diffrn_reflns_limit_l_min        -15
_diffrn_reflns_limit_l_max        15
_diffrn_reflns_theta_min          2.97
_diffrn_reflns_theta_max          34.78
_reflns_number_total              5889
_reflns_number_gt                 5509
_reflns_threshold_expression      >2sigma(I)

_computing_data_collection      'Bruker SMART'
_computing_cell_refinement      'Bruker SMART'
_computing_data_reduction       'Bruker SAINT'
_computing_structure_solution   'SHELXTS (Sheldrick, 1997)'
_computing_structure_refinement 'SHELXTL (Sheldrick, 1997)'
_computing_molecular_graphics   'ORTEP'
_computing_publication_material 'SHELXTL (Sheldrick, 1997)'


_refine_special_details
;
 Refinement of F^2^ against ALL reflections.  The weighted R-factor wR
and
 goodness of fit S are based on F^2^, conventional R-factors R are
based
 on F, with F set to zero for negative F^2^. The threshold expression
of
 F^2^ > 2sigma(F^2^) is used only for calculating R-factors(gt) etc.
and is
```

```
 not relevant to the choice of reflections for refinement.  R-factors
based
 on F^2^ are statistically about twice as large as those based on F,
and R-
 factors based on ALL data will be even larger.
;

_refine_ls_structure_factor_coef  Fsqd
_refine_ls_matrix_type            full
_refine_ls_weighting_scheme       calc
_refine_ls_weighting_details
 'calc w=1/[\s^2^(Fo^2^)+(0.0520P)^2^+0.0810P] where
P=(Fo^2^+2Fc^2^)/3'
_atom_sites_solution_primary      direct
_atom_sites_solution_secondary    difmap
_atom_sites_solution_hydrogens    geom
_refine_ls_hydrogen_treatment     constr
_refine_ls_extinction_method      none
_refine_ls_extinction_coef        ?
_refine_ls_number_reflns          5889
_refine_ls_number_parameters      217
_refine_ls_number_restraints      13
_refine_ls_R_factor_all           0.0425
_refine_ls_R_factor_gt            0.0399
_refine_ls_wR_factor_ref          0.1054
_refine_ls_wR_factor_gt           0.1033
_refine_ls_goodness_of_fit_ref    1.037
_refine_ls_restrained_S_all       1.056
_refine_ls_shift/su_max           0.001
_refine_ls_shift/su_mean          0.000

loop_
 _atom_site_label
 _atom_site_type_symbol
 _atom_site_fract_x
 _atom_site_fract_y
 _atom_site_fract_z
 _atom_site_U_iso_or_equiv
 _atom_site_adp_type
 _atom_site_occupancy
 _atom_site_symmetry_multiplicity
 _atom_site_calc_flag
 _atom_site_refinement_flags
 _atom_site_disorder_assembly
 _atom_site_disorder_group
Pd1 Pd 0.5000 0.5000 1.0000 0.01387(7) Uani 1 2 d SD . .
P1 P 0.70870(5) 0.53386(5) 1.21151(5) 0.01493(10) Uani 1 1 d . A .
O1 O 0.6704(4) 0.6888(4) 0.9428(4) 0.0203(8) Uiso 0.566(3) 1 d PD A 1
O2 O 0.6889(6) 0.4909(6) 0.8479(5) 0.0300(13) Uiso 0.566(3) 1 d PD A 1
N1 N 0.6317(3) 0.5663(3) 0.9190(3) 0.0155(6) Uiso 0.566(3) 1 d PD A 1
C1 C 0.8749(2) 0.64626(19) 1.2329(2) 0.0178(3) Uani 1 1 d . . .
H13 H 0.8706 0.6162 1.1307 0.021 Uiso 1 1 calc R A .
C2 C 0.8740(2) 0.8057(2) 1.2888(2) 0.0206(3) Uani 1 1 d . A .
H14A H 0.7790 0.8177 1.2300 0.025 Uiso 1 1 calc R . .
H14B H 0.8865 0.8444 1.3932 0.025 Uiso 1 1 calc R . .
C3 C 0.9974(2) 0.8878(2) 1.2756(3) 0.0252(4) Uani 1 1 d . . .
H15A H 0.9808 0.8534 1.1703 0.030 Uiso 1 1 calc R A .
```

```
H15B H 0.9968 0.9902 1.3139 0.030 Uiso 1 1 calc R . .
C4 C 1.1457(2) 0.8688(2) 1.3620(3) 0.0270(4) Uani 1 1 d . A .
H16A H 1.2225 0.9172 1.3460 0.032 Uiso 1 1 calc R . .
H16B H 1.1675 0.9133 1.4689 0.032 Uiso 1 1 calc R . .
C5 C 1.1465(2) 0.7115(2) 1.3126(3) 0.0257(4) Uani 1 1 d . . .
H17A H 1.1370 0.6704 1.2091 0.031 Uiso 1 1 calc R A .
H17B H 1.2412 0.7017 1.3746 0.031 Uiso 1 1 calc R . .
C6 C 1.0235(2) 0.6277(2) 1.3235(2) 0.0219(4) Uani 1 1 d . A .
H18A H 1.0377 0.6619 1.4282 0.026 Uiso 1 1 calc R . .
H18B H 1.0253 0.5256 1.2857 0.026 Uiso 1 1 calc R . .
C7 C 0.7605(2) 0.36367(19) 1.2010(2) 0.0179(3) Uani 1 1 d . . .
H7 H 0.8585 0.3881 1.2829 0.021 Uiso 1 1 calc R A .
C8 C 0.7772(2) 0.2860(2) 1.0544(2) 0.0229(4) Uani 1 1 d . A .
H8A H 0.6826 0.2634 0.9704 0.027 Uiso 1 1 calc R . .
H8B H 0.8494 0.3494 1.0470 0.027 Uiso 1 1 calc R . .
C9 C 0.8273(2) 0.1477(2) 1.0450(3) 0.0271(4) Uani 1 1 d . . .
H9A H 0.8315 0.0973 0.9478 0.033 Uiso 1 1 calc R A .
H9B H 0.9266 0.1711 1.1225 0.033 Uiso 1 1 calc R . .
C10 C 0.7231(2) 0.0505(2) 1.0651(2) 0.0253(4) Uani 1 1 d . A .
H10A H 0.6260 0.0198 0.9824 0.030 Uiso 1 1 calc R . .
H10B H 0.7600 -0.0354 1.0636 0.030 Uiso 1 1 calc R . .
C11 C 0.7090(3) 0.1281(2) 1.2112(2) 0.0265(4) Uani 1 1 d . . .
H11A H 0.8048 0.1521 1.2940 0.032 Uiso 1 1 calc R A .
H11B H 0.6391 0.0645 1.2208 0.032 Uiso 1 1 calc R . .
C12 C 0.6565(2) 0.2650(2) 1.2207(2) 0.0227(4) Uani 1 1 d . A .
H12A H 0.6519 0.3146 1.3179 0.027 Uiso 1 1 calc R . .
H12B H 0.5572 0.2407 1.1431 0.027 Uiso 1 1 calc R . .
C13 C 0.6747(2) 0.60493(19) 1.38152(19) 0.0175(3) Uani 1 1 d . . .
H1 H 0.5951 0.5302 1.3664 0.021 Uiso 1 1 calc R A .
C14 C 0.8028(2) 0.6250(2) 1.5238(2) 0.0211(4) Uani 1 1 d . A .
H2A H 0.8406 0.5376 1.5068 0.025 Uiso 1 1 calc R . .
H2B H 0.8827 0.7047 1.5512 0.025 Uiso 1 1 calc R . .
C15 C 0.7516(2) 0.6567(2) 1.6492(2) 0.0239(4) Uani 1 1 d . . .
H3A H 0.8348 0.6714 1.7407 0.029 Uiso 1 1 calc R A .
H3B H 0.6762 0.5741 1.6241 0.029 Uiso 1 1 calc R . .
C16 C 0.6886(2) 0.7893(2) 1.6762(2) 0.0236(4) Uani 1 1 d . A .
H4A H 0.7666 0.8738 1.7115 0.028 Uiso 1 1 calc R . .
H4B H 0.6512 0.8040 1.7537 0.028 Uiso 1 1 calc R . .
C17 C 0.5647(2) 0.7736(2) 1.5340(2) 0.0237(4) Uani 1 1 d . . .
H5A H 0.5302 0.8630 1.5525 0.028 Uiso 1 1 calc R A .
H5B H 0.4820 0.6962 1.5054 0.028 Uiso 1 1 calc R . .
C18 C 0.6141(2) 0.7403(2) 1.4064(2) 0.0215(4) Uani 1 1 d . A .
H6A H 0.6905 0.8215 1.4303 0.026 Uiso 1 1 calc R . .
H6B H 0.5305 0.7257 1.3151 0.026 Uiso 1 1 calc R . .
O1A O 0.6053(7) 0.6607(7) 0.9778(7) 0.0261(17) Uiso 0.228(5) 1 d PD A
2
O2B O 0.6340(8) 0.4638(8) 0.8884(9) 0.030(2) Uiso 0.206(5) 1 d PD A 3
N1B N 0.6981(9) 0.5633(10) 0.8763(9) 0.0201(18) Uiso 0.206(5) 1 d PD A
3
N1A N 0.6848(8) 0.6320(10) 0.9076(9) 0.0224(18) Uiso 0.228(5) 1 d PD A
2
O1B O 0.6556(12) 0.7047(10) 0.9630(11) 0.024(3) Uiso 0.206(5) 1 d PD A
3
O2A O 0.6802(11) 0.4687(10) 0.8463(11) 0.019(2) Uiso 0.228(5) 1 d PD A
2

loop_
```

```
 _atom_site_aniso_label
 _atom_site_aniso_U_11
 _atom_site_aniso_U_22
 _atom_site_aniso_U_33
 _atom_site_aniso_U_23
 _atom_site_aniso_U_13
 _atom_site_aniso_U_12
Pd1 0.01282(10) 0.01479(10) 0.01186(9) 0.00598(7) 0.00258(7)
0.00176(7)
P1 0.0148(2) 0.0137(2) 0.0130(2) 0.00526(16) 0.00246(16) 0.00263(16)
C1 0.0150(8) 0.0171(8) 0.0176(8) 0.0072(6) 0.0029(6) 0.0024(6)
C2 0.0192(8) 0.0162(8) 0.0235(9) 0.0080(7) 0.0061(7) 0.0031(7)
C3 0.0220(9) 0.0212(9) 0.0320(10) 0.0145(8) 0.0077(8) 0.0024(7)
C4 0.0184(9) 0.0243(9) 0.0328(10) 0.0132(8) 0.0043(8) -0.0003(7)
C5 0.0167(8) 0.0263(10) 0.0314(10) 0.0129(8) 0.0060(8) 0.0053(7)
C6 0.0165(8) 0.0205(8) 0.0241(9) 0.0103(7) 0.0025(7) 0.0032(7)
C7 0.0180(8) 0.0148(7) 0.0177(7) 0.0071(6) 0.0035(6) 0.0043(6)
C8 0.0282(10) 0.0169(8) 0.0226(9) 0.0060(7) 0.0116(8) 0.0064(7)
C9 0.0283(10) 0.0178(9) 0.0303(10) 0.0050(8) 0.0118(9) 0.0070(8)
C10 0.0265(10) 0.0147(8) 0.0253(9) 0.0054(7) 0.0036(8) 0.0034(7)
C11 0.0307(11) 0.0161(8) 0.0288(10) 0.0114(7) 0.0061(8) 0.0039(8)
C12 0.0254(9) 0.0179(8) 0.0256(9) 0.0112(7) 0.0094(8) 0.0041(7)
C13 0.0177(8) 0.0173(8) 0.0137(7) 0.0058(6) 0.0032(6) 0.0033(6)
C14 0.0215(9) 0.0231(9) 0.0155(8) 0.0075(7) 0.0045(7) 0.0064(7)
C15 0.0258(9) 0.0255(9) 0.0169(8) 0.0087(7) 0.0056(7) 0.0031(8)
C16 0.0266(10) 0.0228(9) 0.0177(8) 0.0052(7) 0.0093(7) 0.0032(8)
C17 0.0231(9) 0.0243(9) 0.0225(9) 0.0075(7) 0.0102(8) 0.0073(7)
C18 0.0235(9) 0.0211(8) 0.0188(8) 0.0079(7) 0.0076(7) 0.0080(7)

_geom_special_details
;
 All esds (except the esd in the dihedral angle between two l.s.
planes)
 are estimated using the full covariance matrix.  The cell esds are
taken
 into account individually in the estimation of esds in distances,
angles
 and torsion angles; correlations between esds in cell parameters are
only
 used when they are defined by crystal symmetry.  An approximate
(isotropic)
 treatment of cell esds is used for estimating esds involving l.s.
planes.
;

loop_
 _geom_bond_atom_site_label_1
 _geom_bond_atom_site_label_2
 _geom_bond_distance
 _geom_bond_site_symmetry_2
 _geom_bond_publ_flag
Pd1 N1 2.024(3) 2_667 ?
Pd1 N1 2.024(3) . ?
Pd1 O1A 2.050(6) . ?
Pd1 O1A 2.050(6) 2_667 ?
Pd1 O2B 2.056(7) . ?
Pd1 O2B 2.056(7) 2_667 ?
```

Pd1 P1 2.3732(5) . ?
Pd1 P1 2.3732(5) 2_667 ?
P1 C13 1.8499(19) . ?
P1 C7 1.8569(18) . ?
P1 C1 1.864(2) . ?
O1 N1 1.209(4) . ?
O2 N1 1.202(5) . ?
C1 C2 1.542(3) . ?
C1 C6 1.544(3) . ?
C1 H13 1.0000 . ?
C2 C3 1.534(3) . ?
C2 H14A 0.9900 . ?
C2 H14B 0.9900 . ?
C3 C4 1.528(3) . ?
C3 H15A 0.9900 . ?
C3 H15B 0.9900 . ?
C4 C5 1.524(3) . ?
C4 H16A 0.9900 . ?
C4 H16B 0.9900 . ?
C5 C6 1.526(3) . ?
C5 H17A 0.9900 . ?
C5 H17B 0.9900 . ?
C6 H18A 0.9900 . ?
C6 H18B 0.9900 . ?
C7 C12 1.537(3) . ?
C7 C8 1.545(3) . ?
C7 H7 1.0000 . ?
C8 C9 1.540(3) . ?
C8 H8A 0.9900 . ?
C8 H8B 0.9900 . ?
C9 C10 1.530(3) . ?
C9 H9A 0.9900 . ?
C9 H9B 0.9900 . ?
C10 C11 1.527(3) . ?
C10 H10A 0.9900 . ?
C10 H10B 0.9900 . ?
C11 C12 1.535(3) . ?
C11 H11A 0.9900 . ?
C11 H11B 0.9900 . ?
C12 H12A 0.9900 . ?
C12 H12B 0.9900 . ?
C13 C14 1.541(3) . ?
C13 C18 1.544(3) . ?
C13 H1 1.0000 . ?
C14 C15 1.530(3) . ?
C14 H2A 0.9900 . ?
C14 H2B 0.9900 . ?
C15 C16 1.531(3) . ?
C15 H3A 0.9900 . ?
C15 H3B 0.9900 . ?
C16 C17 1.534(3) . ?
C16 H4A 0.9900 . ?
C16 H4B 0.9900 . ?
C17 C18 1.538(3) . ?
C17 H5A 0.9900 . ?
C17 H5B 0.9900 . ?
C18 H6A 0.9900 . ?

```
C18 H6B 0.9900 . ?
O1A N1A 1.254(8) . ?
O2B N1B 1.253(8) . ?
N1B O1B 1.598(12) . ?
N1A O2A 1.568(12) . ?

loop_
 _geom_angle_atom_site_label_1
 _geom_angle_atom_site_label_2
 _geom_angle_atom_site_label_3
 _geom_angle
 _geom_angle_site_symmetry_1
 _geom_angle_site_symmetry_3
 _geom_angle_publ_flag
N1 Pd1 N1 179.999(1) 2_667 . ?
N1 Pd1 O1A 149.87(19) 2_667 . ?
N1 Pd1 O1A 30.13(19) . . ?
N1 Pd1 O1A 30.13(19) 2_667 2_667 ?
N1 Pd1 O1A 149.87(19) . 2_667 ?
O1A Pd1 O1A 179.999(2) . 2_667 ?
N1 Pd1 O2B 151.7(2) 2_667 . ?
N1 Pd1 O2B 28.3(2) . . ?
O1A Pd1 O2B 58.4(3) . . ?
O1A Pd1 O2B 121.6(3) 2_667 . ?
N1 Pd1 O2B 28.3(2) 2_667 2_667 ?
N1 Pd1 O2B 151.7(2) . 2_667 ?
O1A Pd1 O2B 121.6(3) . 2_667 ?
O1A Pd1 O2B 58.4(3) 2_667 2_667 ?
O2B Pd1 O2B 180.000(3) . 2_667 ?
N1 Pd1 P1 90.61(8) 2_667 . ?
N1 Pd1 P1 89.39(8) . . ?
O1A Pd1 P1 90.50(19) . . ?
O1A Pd1 P1 89.5(2) 2_667 . ?
O2B Pd1 P1 86.4(2) . . ?
O2B Pd1 P1 93.6(2) 2_667 . ?
N1 Pd1 P1 89.39(8) 2_667 2_667 ?
N1 Pd1 P1 90.61(8) . 2_667 ?
O1A Pd1 P1 89.50(19) . 2_667 ?
O1A Pd1 P1 90.5(2) 2_667 2_667 ?
O2B Pd1 P1 93.6(2) . 2_667 ?
O2B Pd1 P1 86.4(2) 2_667 2_667 ?
P1 Pd1 P1 180.00(3) . 2_667 ?
C13 P1 C7 104.65(8) . . ?
C13 P1 C1 111.03(9) . . ?
C7 P1 C1 104.08(9) . . ?
C13 P1 Pd1 111.68(6) . . ?
C7 P1 Pd1 112.00(6) . . ?
C1 P1 Pd1 112.86(6) . . ?
O2 N1 O1 110.7(4) . . ?
O2 N1 Pd1 124.7(3) . . ?
O1 N1 Pd1 124.5(3) . . ?
C2 C1 C6 109.59(15) . . ?
C2 C1 P1 112.97(13) . . ?
C6 C1 P1 116.88(13) . . ?
C2 C1 H13 105.5 . . ?
C6 C1 H13 105.5 . . ?
P1 C1 H13 105.5 . . ?
```

C3 C2 C1 110.28(16) . . ?
C3 C2 H14A 109.6 . . ?
C1 C2 H14A 109.6 . . ?
C3 C2 H14B 109.6 . . ?
C1 C2 H14B 109.6 . . ?
H14A C2 H14B 108.1 . . ?
C4 C3 C2 111.17(17) . . ?
C4 C3 H15A 109.4 . . ?
C2 C3 H15A 109.4 . . ?
C4 C3 H15B 109.4 . . ?
C2 C3 H15B 109.4 . . ?
H15A C3 H15B 108.0 . . ?
C5 C4 C3 110.64(17) . . ?
C5 C4 H16A 109.5 . . ?
C3 C4 H16A 109.5 . . ?
C5 C4 H16B 109.5 . . ?
C3 C4 H16B 109.5 . . ?
H16A C4 H16B 108.1 . . ?
C6 C5 C4 112.49(18) . . ?
C6 C5 H17A 109.1 . . ?
C4 C5 H17A 109.1 . . ?
C6 C5 H17B 109.1 . . ?
C4 C5 H17B 109.1 . . ?
H17A C5 H17B 107.8 . . ?
C5 C6 C1 110.11(16) . . ?
C5 C6 H18A 109.6 . . ?
C1 C6 H18A 109.6 . . ?
C5 C6 H18B 109.6 . . ?
C1 C6 H18B 109.6 . . ?
H18A C6 H18B 108.2 . . ?
C12 C7 C8 110.81(16) . . ?
C12 C7 P1 114.44(13) . . ?
C8 C7 P1 109.63(13) . . ?
C12 C7 H7 107.2 . . ?
C8 C7 H7 107.2 . . ?
P1 C7 H7 107.2 . . ?
C9 C8 C7 111.10(16) . . ?
C9 C8 H8A 109.4 . . ?
C7 C8 H8A 109.4 . . ?
C9 C8 H8B 109.4 . . ?
C7 C8 H8B 109.4 . . ?
H8A C8 H8B 108.0 . . ?
C10 C9 C8 111.00(18) . . ?
C10 C9 H9A 109.4 . . ?
C8 C9 H9A 109.4 . . ?
C10 C9 H9B 109.4 . . ?
C8 C9 H9B 109.4 . . ?
H9A C9 H9B 108.0 . . ?
C11 C10 C9 110.56(17) . . ?
C11 C10 H10A 109.5 . . ?
C9 C10 H10A 109.5 . . ?
C11 C10 H10B 109.5 . . ?
C9 C10 H10B 109.5 . . ?
H10A C10 H10B 108.1 . . ?
C10 C11 C12 111.44(17) . . ?
C10 C11 H11A 109.3 . . ?
C12 C11 H11A 109.3 . . ?

```
C10 C11 H11B 109.3 . . ?
C12 C11 H11B 109.3 . . ?
H11A C11 H11B 108.0 . . ?
C11 C12 C7 110.44(17) . . ?
C11 C12 H12A 109.6 . . ?
C7 C12 H12A 109.6 . . ?
C11 C12 H12B 109.6 . . ?
C7 C12 H12B 109.6 . . ?
H12A C12 H12B 108.1 . . ?
C14 C13 C18 110.32(15) . . ?
C14 C13 P1 115.23(13) . . ?
C18 C13 P1 114.45(12) . . ?
C14 C13 H1 105.2 . . ?
C18 C13 H1 105.2 . . ?
P1 C13 H1 105.2 . . ?
C15 C14 C13 109.79(16) . . ?
C15 C14 H2A 109.7 . . ?
C13 C14 H2A 109.7 . . ?
C15 C14 H2B 109.7 . . ?
C13 C14 H2B 109.7 . . ?
H2A C14 H2B 108.2 . . ?
C14 C15 C16 111.24(16) . . ?
C14 C15 H3A 109.4 . . ?
C16 C15 H3A 109.4 . . ?
C14 C15 H3B 109.4 . . ?
C16 C15 H3B 109.4 . . ?
H3A C15 H3B 108.0 . . ?
C15 C16 C17 110.80(16) . . ?
C15 C16 H4A 109.5 . . ?
C17 C16 H4A 109.5 . . ?
C15 C16 H4B 109.5 . . ?
C17 C16 H4B 109.5 . . ?
H4A C16 H4B 108.1 . . ?
C16 C17 C18 111.73(17) . . ?
C16 C17 H5A 109.3 . . ?
C18 C17 H5A 109.3 . . ?
C16 C17 H5B 109.3 . . ?
C18 C17 H5B 109.3 . . ?
H5A C17 H5B 107.9 . . ?
C17 C18 C13 109.60(16) . . ?
C17 C18 H6A 109.7 . . ?
C13 C18 H6A 109.7 . . ?
C17 C18 H6B 109.7 . . ?
C13 C18 H6B 109.7 . . ?
H6A C18 H6B 108.2 . . ?
N1A O1A Pd1 118.8(6) . . ?
N1B O2B Pd1 120.9(6) . . ?
O2B N1B O1B 106.9(7) . . ?
O1A N1A O2A 110.1(7) . . ?

loop_
 _geom_torsion_atom_site_label_1
 _geom_torsion_atom_site_label_2
 _geom_torsion_atom_site_label_3
 _geom_torsion_atom_site_label_4
 _geom_torsion
 _geom_torsion_site_symmetry_1
```

```
 _geom_torsion_site_symmetry_2
 _geom_torsion_site_symmetry_3
 _geom_torsion_site_symmetry_4
 _geom_torsion_publ_flag
N1 Pd1 P1 C13 -37.80(10) 2_667 . . . ?
N1 Pd1 P1 C13 142.20(10) . . . . ?
O1A Pd1 P1 C13 112.10(18) . . . . ?
O1A Pd1 P1 C13 -67.90(18) 2_667 . . . ?
O2B Pd1 P1 C13 170.4(2) . . . . ?
O2B Pd1 P1 C13 -9.6(2) 2_667 . . . ?
P1 Pd1 P1 C13 -79(17) 2_667 . . . ?
N1 Pd1 P1 C7 79.21(10) 2_667 . . . ?
N1 Pd1 P1 C7 -100.79(10) . . . . ?
O1A Pd1 P1 C7 -130.89(19) . . . . ?
O1A Pd1 P1 C7 49.11(19) 2_667 . . . ?
O2B Pd1 P1 C7 -72.6(2) . . . . ?
O2B Pd1 P1 C7 107.4(2) 2_667 . . . ?
P1 Pd1 P1 C7 38(17) 2_667 . . . ?
N1 Pd1 P1 C1 -163.72(10) 2_667 . . . ?
N1 Pd1 P1 C1 16.28(10) . . . . ?
O1A Pd1 P1 C1 -13.82(18) . . . . ?
O1A Pd1 P1 C1 166.18(18) 2_667 . . . ?
O2B Pd1 P1 C1 44.4(2) . . . . ?
O2B Pd1 P1 C1 -135.6(2) 2_667 . . . ?
P1 Pd1 P1 C1 155(17) 2_667 . . . ?
N1 Pd1 N1 O2 -131(36) 2_667 . . . ?
O1A Pd1 N1 O2 177.9(6) . . . . ?
O1A Pd1 N1 O2 -2.0(6) 2_667 . . . ?
O2B Pd1 N1 O2 2.5(6) . . . . ?
O2B Pd1 N1 O2 -177.5(6) 2_667 . . . ?
P1 Pd1 N1 O2 85.9(4) . . . . ?
P1 Pd1 N1 O2 -94.1(4) 2_667 . . . ?
N1 Pd1 N1 O1 54(36) 2_667 . . . ?
O1A Pd1 N1 O1 2.6(4) . . . . ?
O1A Pd1 N1 O1 -177.4(4) 2_667 . . . ?
O2B Pd1 N1 O1 -172.9(6) . . . . ?
O2B Pd1 N1 O1 7.1(6) 2_667 . . . ?
P1 Pd1 N1 O1 -89.5(3) . . . . ?
P1 Pd1 N1 O1 90.5(3) 2_667 . . . ?
C13 P1 C1 C2 -51.17(15) . . . . ?
C7 P1 C1 C2 -163.24(13) . . . . ?
Pd1 P1 C1 C2 75.11(14) . . . . ?
C13 P1 C1 C6 77.38(16) . . . . ?
C7 P1 C1 C6 -34.69(16) . . . . ?
Pd1 P1 C1 C6 -156.34(12) . . . . ?
C6 C1 C2 C3 58.6(2) . . . . ?
P1 C1 C2 C3 -169.12(13) . . . . ?
C1 C2 C3 C4 -57.8(2) . . . . ?
C2 C3 C4 C5 55.2(2) . . . . ?
C3 C4 C5 C6 -55.0(2) . . . . ?
C4 C5 C6 C1 56.4(2) . . . . ?
C2 C1 C6 C5 -57.6(2) . . . . ?
P1 C1 C6 C5 172.28(14) . . . . ?
C13 P1 C7 C12 49.26(15) . . . . ?
C1 P1 C7 C12 165.87(14) . . . . ?
Pd1 P1 C7 C12 -71.91(14) . . . . ?
C13 P1 C7 C8 174.45(13) . . . . ?
```

```
C1 P1 C7 C8 -68.94(15) . . . . ?
Pd1 P1 C7 C8 53.28(14) . . . . ?
C12 C7 C8 C9 -55.5(2) . . . . ?
P1 C7 C8 C9 177.28(14) . . . . ?
C7 C8 C9 C10 55.8(2) . . . . ?
C8 C9 C10 C11 -56.5(2) . . . . ?
C9 C10 C11 C12 57.4(2) . . . . ?
C10 C11 C12 C7 -57.1(2) . . . . ?
C8 C7 C12 C11 55.8(2) . . . . ?
P1 C7 C12 C11 -179.65(13) . . . . ?
C7 P1 C13 C14 56.45(15) . . . . ?
C1 P1 C13 C14 -55.26(16) . . . . ?
Pd1 P1 C13 C14 177.82(12) . . . . ?
C7 P1 C13 C18 -174.06(14) . . . . ?
C1 P1 C13 C18 74.24(16) . . . . ?
Pd1 P1 C13 C18 -52.69(15) . . . . ?
C18 C13 C14 C15 59.2(2) . . . . ?
P1 C13 C14 C15 -169.32(13) . . . . ?
C13 C14 C15 C16 -57.9(2) . . . . ?
C14 C15 C16 C17 55.9(2) . . . . ?
C15 C16 C17 C18 -55.3(2) . . . . ?
C16 C17 C18 C13 56.3(2) . . . . ?
C14 C13 C18 C17 -58.2(2) . . . . ?
P1 C13 C18 C17 169.92(14) . . . . ?
N1 Pd1 O1A N1A 178.0(5) 2_667 . . . ?
N1 Pd1 O1A N1A -2.0(5) . . . . ?
O1A Pd1 O1A N1A 76(8) 2_667 . . . ?
O2B Pd1 O1A N1A 0.6(6) . . . . ?
O2B Pd1 O1A N1A -179.4(6) 2_667 . . . ?
P1 Pd1 O1A N1A 86.0(6) . . . . ?
P1 Pd1 O1A N1A -94.0(6) 2_667 . . . ?
N1 Pd1 O2B N1B -177.0(5) 2_667 . . . ?
N1 Pd1 O2B N1B 3.0(5) . . . . ?
O1A Pd1 O2B N1B 0.3(7) . . . . ?
O1A Pd1 O2B N1B -179.7(7) 2_667 . . . ?
O2B Pd1 O2B N1B 40(100) 2_667 . . . ?
P1 Pd1 O2B N1B -92.5(7) . . . . ?
P1 Pd1 O2B N1B 87.5(7) 2_667 . . . ?
Pd1 O2B N1B O1B 1.3(10) . . . . ?
Pd1 O1A N1A O2A 1.6(9) . . . . ?

_diffrn_measured_fraction_theta_max    0.931
_diffrn_reflns_theta_full              30.50
_diffrn_measured_fraction_theta_full   0.992
_refine_diff_density_max    0.616
_refine_diff_density_min   -0.596
_refine_diff_density_rms    0.112


data_p10prr98

_audit_creation_method            SHELXL-97
_chemical_name_systematic
;
Bis(tricyclohexanephosphine)bis(n1-nitro)platinum (II)
;
_chemical_name_common             ?
```

```
_chemical_melting_point           ?
_chemical_formula_moiety          'C36 H66 N2 O4 P2 Pt'
_chemical_formula_sum             'C36 H66 N2 O4 P2 Pt'
_chemical_formula_weight          847.94

loop_
 _atom_type_symbol
 _atom_type_description
 _atom_type_scat_dispersion_real
 _atom_type_scat_dispersion_imag
 _atom_type_scat_source
 'C'  'C'   0.0033   0.0016
 'International Tables Vol C Tables 4.2.6.8 and 6.1.1.4'
 'H'  'H'   0.0000   0.0000
 'International Tables Vol C Tables 4.2.6.8 and 6.1.1.4'
 'N'  'N'   0.0061   0.0033
 'International Tables Vol C Tables 4.2.6.8 and 6.1.1.4'
 'O'  'O'   0.0106   0.0060
 'International Tables Vol C Tables 4.2.6.8 and 6.1.1.4'
 'P'  'P'   0.1023   0.0942
 'International Tables Vol C Tables 4.2.6.8 and 6.1.1.4'
 'Pt'  'Pt'  -1.7033   8.3905
 'International Tables Vol C Tables 4.2.6.8 and 6.1.1.4'

_symmetry_cell_setting            Triclinic
_symmetry_space_group_name_H-M    P-1

loop_
 _symmetry_equiv_pos_as_xyz
 'x, y, z'
 '-x, -y, -z'

_cell_length_a                    10.122(5)
_cell_length_b                    10.388(5)
_cell_length_c                    10.617(5)
_cell_angle_alpha                 111.736(5)
_cell_angle_beta                  110.638(5)
_cell_angle_gamma                 94.229(5)
_cell_volume                      943.3(8)
_cell_formula_units_Z             1
_cell_measurement_temperature     100(2)
_cell_measurement_reflns_used     2861
_cell_measurement_theta_min       3.5165
_cell_measurement_theta_max       32.3372

_exptl_crystal_description        block
_exptl_crystal_colour             colourless
_exptl_crystal_size_max           0.10
_exptl_crystal_size_mid           0.08
_exptl_crystal_size_min           0.08
_exptl_crystal_density_meas       ?
_exptl_crystal_density_diffrn     1.493
_exptl_crystal_density_method     'not measured'
_exptl_crystal_F_000              436
_exptl_absorpt_coefficient_mu     3.842
_exptl_absorpt_correction_T_min   0.6999
_exptl_absorpt_correction_T_max   0.7486
```

```
_exptl_absorpt_correction_type            'multi-scan'
_exptl_absorpt_process_details
;
CrysAlisPro, Oxford Diffraction Ltd.,
Version 1.171.33.55 (release 05-01-2010 CrysAlis171 .NET)
(compiled Jan  5 2010,16:28:46)
Empirical absorption correction using spherical harmonics,
 implemented in SCALE3 ABSPACK scaling algorithm.
;

_diffrn_ambient_temperature       100(2)
_diffrn_radiation_wavelength      0.7107
_diffrn_radiation_type            'Mo K\a'
_diffrn_radiation_source          'Enhance (Mo) X-ray Source'
_diffrn_radiation_monochromator   'graphite'
_diffrn_measurement_device_type   'Xcalibur, Atlas, Gemini ultra'
_diffrn_measurement_method        '\w scans'
_diffrn_detector_area_resol_mean  ?
_diffrn_standards_number          ?
_diffrn_standards_interval_count  ?
_diffrn_standards_interval_time   ?
_diffrn_standards_decay_%         0
_diffrn_reflns_number             18288
_diffrn_reflns_av_R_equivalents   0.0287
_diffrn_reflns_av_sigmaI/netI     0.0359
_diffrn_reflns_limit_h_min        -15
_diffrn_reflns_limit_h_max        14
_diffrn_reflns_limit_k_min        -15
_diffrn_reflns_limit_k_max        15
_diffrn_reflns_limit_l_min        -15
_diffrn_reflns_limit_l_max        15
_diffrn_reflns_theta_min          2.95
_diffrn_reflns_theta_max          32.58
_reflns_number_total              6334
_reflns_number_gt                 6325
_reflns_threshold_expression      >2sigma(I)

_diffrn_orient_matrix_UB_11      -0.0085038000
_diffrn_orient_matrix_UB_12       0.0283198000
_diffrn_orient_matrix_UB_13      -0.0530221000
_diffrn_orient_matrix_UB_21      -0.0634326000
_diffrn_orient_matrix_UB_22      -0.0548812000
_diffrn_orient_matrix_UB_23      -0.0511448000
_diffrn_orient_matrix_UB_31      -0.0428747000
_diffrn_orient_matrix_UB_32       0.0436508000
_diffrn_orient_matrix_UB_33       0.0281068000
_diffrn_measurement_details
;
#__ type_ start__ end____ width___ exp.time_
  1 omega  -48.00   -4.00   1.0000    6.0000
omega____ theta____ kappa____ phi______ frames
    -      -19.3054  -69.0000 -146.0000 44

#__ type_ start__ end____ width___ exp.time_
  2 omega  -95.00  -20.00   1.0000    6.0000
omega____ theta____ kappa____ phi______ frames
    -      -19.3054  -69.0000   66.0000 75
```

```
;

_computing_data_collection
;
CrysAlisPro, Oxford Diffraction Ltd.,
Version 1.171.33.55 (release 05-01-2010 CrysAlis171 .NET)
(compiled Jan  5 2010,16:28:46)
;
_computing_cell_refinement
;
CrysAlisPro, Oxford Diffraction Ltd.,
Version 1.171.33.55 (release 05-01-2010 CrysAlis171 .NET)
(compiled Jan  5 2010,16:28:46)
;
_computing_data_reduction
;
CrysAlisPro, Oxford Diffraction Ltd.,
Version 1.171.33.55 (release 05-01-2010 CrysAlis171 .NET)
(compiled Jan  5 2010,16:28:46)
;
_computing_structure_solution   'SHELXTS (Sheldrick, 1997)'
_computing_structure_refinement 'SHELXTL (Sheldrick, 1997)'
_computing_molecular_graphics   'ORTEP'
_computing_publication_material 'SHELXTL (Sheldrick, 1997)'

_refine_special_details
;
 Refinement of F^2^ against ALL reflections.  The weighted R-factor wR
and
 goodness of fit S are based on F^2^, conventional R-factors R are
based
 on F, with F set to zero for negative F^2^. The threshold expression
of
 F^2^ > 2sigma(F^2^) is used only for calculating R-factors(gt) etc.
and is
 not relevant to the choice of reflections for refinement.  R-factors
based
 on F^2^ are statistically about twice as large as those based on F,
and R-
 factors based on ALL data will be even larger.
;

_refine_ls_structure_factor_coef  Fsqd
_refine_ls_matrix_type            full
_refine_ls_weighting_scheme       calc
_refine_ls_weighting_details
 'calc w=1/[\s^2^(Fo^2^)+(0.0149P)^2^+0.0000P] where
P=(Fo^2^+2Fc^2^)/3'
_atom_sites_solution_primary      direct
_atom_sites_solution_secondary    difmap
_atom_sites_solution_hydrogens    geom
_refine_ls_hydrogen_treatment     constr
_refine_ls_extinction_method      none
_refine_ls_extinction_coef        ?
_refine_ls_number_reflns          6334
_refine_ls_number_parameters      205
_refine_ls_number_restraints      0
```

```
_refine_ls_R_factor_all           0.0185
_refine_ls_R_factor_gt            0.0185
_refine_ls_wR_factor_ref          0.0352
_refine_ls_wR_factor_gt           0.0352
_refine_ls_goodness_of_fit_ref    1.004
_refine_ls_restrained_S_all       1.004
_refine_ls_shift/su_max           0.000
_refine_ls_shift/su_mean          0.000

loop_
 _atom_site_label
 _atom_site_type_symbol
 _atom_site_fract_x
 _atom_site_fract_y
 _atom_site_fract_z
 _atom_site_U_iso_or_equiv
 _atom_site_adp_type
 _atom_site_occupancy
 _atom_site_symmetry_multiplicity
 _atom_site_calc_flag
 _atom_site_refinement_flags
 _atom_site_disorder_assembly
 _atom_site_disorder_group
Pt1 Pt 0.0000 0.0000 0.0000 0.01068(2) Uani 1 2 d S . .
P1 P -0.20711(4) -0.03355(4) -0.21145(4) 0.01165(7) Uani 1 1 d . . .
N1 N -0.13143(14) -0.06214(13) 0.08267(13) 0.0161(2) Uani 1 1 d . . .
O2 O -0.16502(13) -0.18916(12) 0.05651(13) 0.0252(3) Uani 1 1 d . . .
C1 C -0.37193(15) -0.14595(14) -0.23242(16) 0.0141(3) Uani 1 1 d . . .
H1 H -0.3683 -0.1146 -0.1299 0.017 Uiso 1 1 calc R . .
C8 C -0.11640(17) -0.24089(15) -0.40651(17) 0.0181(3) Uani 1 1 d . . .
H8A H -0.0338 -0.2277 -0.3145 0.022 Uiso 1 1 calc R . .
H8B H -0.1940 -0.3215 -0.4315 0.022 Uiso 1 1 calc R . .
C13 C -0.25804(16) 0.13600(14) -0.20090(16) 0.0148(3) Uani 1 1 d . . .
H13 H -0.3546 0.1116 -0.2852 0.018 Uiso 1 1 calc R . .
C3 C -0.49350(18) -0.38646(17) -0.2723(2) 0.0241(3) Uani 1 1 d . . .
H3A H -0.4927 -0.4889 -0.3084 0.029 Uiso 1 1 calc R . .
H3B H -0.4775 -0.3502 -0.1665 0.029 Uiso 1 1 calc R . .
C16 C -0.2225(2) 0.44738(16) -0.06454(19) 0.0266(4) Uani 1 1 d . . .
H16A H -0.1268 0.4787 0.0207 0.032 Uiso 1 1 calc R . .
H16B H -0.2597 0.5331 -0.0637 0.032 Uiso 1 1 calc R . .
C12 C -0.29922(17) -0.12234(16) -0.52546(16) 0.0188(3) Uani 1 1 d . .
.
H12A H -0.3806 -0.2011 -0.5540 0.023 Uiso 1 1 calc R . .
H12B H -0.3348 -0.0338 -0.5085 0.023 Uiso 1 1 calc R . .
C18 C -0.27800(19) 0.21129(15) -0.05624(17) 0.0209(3) Uani 1 1 d . . .
H18A H -0.3513 0.1468 -0.0515 0.025 Uiso 1 1 calc R . .
H18B H -0.1851 0.2339 0.0301 0.025 Uiso 1 1 calc R . .
C4 C -0.64055(18) -0.36925(17) -0.3621(2) 0.0252(4) Uani 1 1 d . . .
H4A H -0.6613 -0.4147 -0.4692 0.030 Uiso 1 1 calc R . .
H4B H -0.7175 -0.4176 -0.3465 0.030 Uiso 1 1 calc R . .
C14 C -0.15281(18) 0.23660(16) -0.21669(19) 0.0211(3) Uani 1 1 d . . .
H14A H -0.0550 0.2613 -0.1362 0.025 Uiso 1 1 calc R . .
H14B H -0.1457 0.1879 -0.3130 0.025 Uiso 1 1 calc R . .
C11 C -0.24819(19) -0.15488(17) -0.65081(17) 0.0224(3) Uani 1 1 d . .
.
H11A H -0.3306 -0.1689 -0.7433 0.027 Uiso 1 1 calc R . .
H11B H -0.1717 -0.0729 -0.6252 0.027 Uiso 1 1 calc R . .
```

```
C15 C -0.2046(2) 0.37274(16) -0.20882(19) 0.0254(4) Uani 1 1 d . . .
H15A H -0.1332 0.4378 -0.2153 0.031 Uiso 1 1 calc R . .
H15B H -0.2986 0.3488 -0.2943 0.031 Uiso 1 1 calc R . .
C7 C -0.17356(16) -0.10430(14) -0.38125(15) 0.0145(3) Uani 1 1 d . . .
H7 H -0.0926 -0.0302 -0.3646 0.017 Uiso 1 1 calc R . .
O1 O -0.18324(13) 0.02575(12) 0.15406(13) 0.0261(3) Uani 1 1 d . . .
C6 C -0.51924(16) -0.12922(16) -0.32639(18) 0.0188(3) Uani 1 1 d . . .
H6A H -0.5212 -0.0271 -0.2906 0.023 Uiso 1 1 calc R . .
H6B H -0.5327 -0.1651 -0.4315 0.023 Uiso 1 1 calc R . .
C10 C -0.18870(19) -0.28777(17) -0.67650(17) 0.0235(3) Uani 1 1 d . .
.
H10A H -0.1508 -0.3033 -0.7539 0.028 Uiso 1 1 calc R . .
H10B H -0.2680 -0.3715 -0.7129 0.028 Uiso 1 1 calc R . .
C5 C -0.64185(18) -0.21219(17) -0.3152(2) 0.0237(3) Uani 1 1 d . . .
H5A H -0.6327 -0.1699 -0.2114 0.028 Uiso 1 1 calc R . .
H5B H -0.7361 -0.2035 -0.3792 0.028 Uiso 1 1 calc R . .
C9 C -0.06708(19) -0.27434(17) -0.53346(18) 0.0226(3) Uani 1 1 d . . .
H9A H -0.0348 -0.3647 -0.5523 0.027 Uiso 1 1 calc R . .
H9B H 0.0168 -0.1976 -0.5037 0.027 Uiso 1 1 calc R . .
C17 C -0.3270(2) 0.34837(16) -0.0482(2) 0.0264(4) Uani 1 1 d . . .
H17A H -0.4250 0.3246 -0.1283 0.032 Uiso 1 1 calc R . .
H17B H -0.3335 0.3977 0.0483 0.032 Uiso 1 1 calc R . .
C2 C -0.37107(17) -0.30525(15) -0.28611(17) 0.0176(3) Uani 1 1 d . . .
H2A H -0.3837 -0.3451 -0.3911 0.021 Uiso 1 1 calc R . .
H2B H -0.2765 -0.3165 -0.2257 0.021 Uiso 1 1 calc R . .

loop_
 _atom_site_aniso_label
 _atom_site_aniso_U_11
 _atom_site_aniso_U_22
 _atom_site_aniso_U_33
 _atom_site_aniso_U_23
 _atom_site_aniso_U_13
 _atom_site_aniso_U_12
Pt1 0.01229(4) 0.01128(4) 0.00880(4) 0.00456(3) 0.00430(3) 0.00316(2)
P1 0.01394(17) 0.01092(15) 0.00953(16) 0.00446(12) 0.00397(14)
0.00355(13)
N1 0.0149(6) 0.0212(6) 0.0123(6) 0.0093(5) 0.0032(5) 0.0041(5)
O2 0.0272(6) 0.0231(6) 0.0296(6) 0.0170(5) 0.0110(5) 0.0024(5)
C1 0.0136(7) 0.0140(6) 0.0137(7) 0.0060(5) 0.0043(5) 0.0033(5)
C8 0.0220(8) 0.0170(7) 0.0166(7) 0.0067(6) 0.0092(6) 0.0079(6)
C13 0.0159(7) 0.0123(6) 0.0146(7) 0.0063(5) 0.0039(6) 0.0041(5)
C3 0.0234(8) 0.0188(7) 0.0342(9) 0.0141(7) 0.0133(7) 0.0048(6)
C16 0.0304(9) 0.0129(7) 0.0285(9) 0.0065(6) 0.0058(7) 0.0051(6)
C12 0.0203(8) 0.0229(7) 0.0129(7) 0.0085(6) 0.0052(6) 0.0072(6)
C18 0.0287(9) 0.0158(7) 0.0194(8) 0.0064(6) 0.0119(7) 0.0075(6)
C4 0.0188(8) 0.0227(8) 0.0325(9) 0.0115(7) 0.0096(7) 0.0010(6)
C14 0.0229(8) 0.0178(7) 0.0269(8) 0.0124(6) 0.0116(7) 0.0058(6)
C11 0.0270(9) 0.0258(8) 0.0135(7) 0.0096(6) 0.0069(6) 0.0023(6)
C15 0.0300(9) 0.0165(7) 0.0297(9) 0.0138(6) 0.0081(7) 0.0050(6)
C7 0.0172(7) 0.0142(6) 0.0115(6) 0.0048(5) 0.0058(6) 0.0038(5)
O1 0.0280(6) 0.0321(6) 0.0259(6) 0.0115(5) 0.0195(5) 0.0119(5)
C6 0.0152(7) 0.0181(7) 0.0212(8) 0.0090(6) 0.0045(6) 0.0052(5)
C10 0.0301(9) 0.0247(8) 0.0145(7) 0.0045(6) 0.0127(7) 0.0030(6)
C5 0.0157(7) 0.0241(8) 0.0306(9) 0.0116(7) 0.0086(7) 0.0055(6)
C9 0.0251(8) 0.0223(7) 0.0220(8) 0.0065(6) 0.0145(7) 0.0071(6)
C17 0.0313(9) 0.0169(7) 0.0283(9) 0.0053(6) 0.0131(8) 0.0098(6)
```

```
C2 0.0174(7) 0.0142(6) 0.0219(8) 0.0080(6) 0.0083(6) 0.0053(5)

_geom_special_details
;
 All esds (except the esd in the dihedral angle between two l.s.
planes)
 are estimated using the full covariance matrix.  The cell esds are
taken
 into account individually in the estimation of esds in distances,
angles
 and torsion angles; correlations between esds in cell parameters are
only
 used when they are defined by crystal symmetry.  An approximate
(isotropic)
 treatment of cell esds is used for estimating esds involving l.s.
planes.
;

loop_
 _geom_bond_atom_site_label_1
 _geom_bond_atom_site_label_2
 _geom_bond_distance
 _geom_bond_site_symmetry_2
 _geom_bond_publ_flag
Pt1 N1 2.0181(14) 2 ?
Pt1 N1 2.0182(14) . ?
Pt1 P1 2.3561(9) 2 ?
Pt1 P1 2.3561(9) . ?
P1 C7 1.8418(16) . ?
P1 C13 1.8463(16) . ?
P1 C1 1.8587(17) . ?
N1 O1 1.2334(16) . ?
N1 O2 1.2368(17) . ?
C1 C6 1.538(2) . ?
C1 C2 1.540(2) . ?
C1 H1 1.0000 . ?
C8 C9 1.530(2) . ?
C8 C7 1.539(2) . ?
C8 H8A 0.9900 . ?
C8 H8B 0.9900 . ?
C13 C14 1.532(2) . ?
C13 C18 1.537(2) . ?
C13 H13 1.0000 . ?
C3 C4 1.523(2) . ?
C3 C2 1.528(2) . ?
C3 H3A 0.9900 . ?
C3 H3B 0.9900 . ?
C16 C17 1.519(3) . ?
C16 C15 1.522(3) . ?
C16 H16A 0.9900 . ?
C16 H16B 0.9900 . ?
C12 C11 1.526(2) . ?
C12 C7 1.542(2) . ?
C12 H12A 0.9900 . ?
C12 H12B 0.9900 . ?
C18 C17 1.525(2) . ?
C18 H18A 0.9900 . ?
```

```
C18 H18B 0.9900 . ?
C4 C5 1.522(2) . ?
C4 H4A 0.9900 . ?
C4 H4B 0.9900 . ?
C14 C15 1.528(2) . ?
C14 H14A 0.9900 . ?
C14 H14B 0.9900 . ?
C11 C10 1.517(2) . ?
C11 H11A 0.9900 . ?
C11 H11B 0.9900 . ?
C15 H15A 0.9900 . ?
C15 H15B 0.9900 . ?
C7 H7 1.0000 . ?
C6 C5 1.523(2) . ?
C6 H6A 0.9900 . ?
C6 H6B 0.9900 . ?
C10 C9 1.530(2) . ?
C10 H10A 0.9900 . ?
C10 H10B 0.9900 . ?
C5 H5A 0.9900 . ?
C5 H5B 0.9900 . ?
C9 H9A 0.9900 . ?
C9 H9B 0.9900 . ?
C17 H17A 0.9900 . ?
C17 H17B 0.9900 . ?
C2 H2A 0.9900 . ?
C2 H2B 0.9900 . ?

loop_
 _geom_angle_atom_site_label_1
 _geom_angle_atom_site_label_2
 _geom_angle_atom_site_label_3
 _geom_angle
 _geom_angle_site_symmetry_1
 _geom_angle_site_symmetry_3
 _geom_angle_publ_flag
N1 Pt1 N1 180.00(5) 2 . ?
N1 Pt1 P1 89.01(5) 2 2 ?
N1 Pt1 P1 90.99(5) . 2 ?
N1 Pt1 P1 90.99(5) 2 . ?
N1 Pt1 P1 89.01(5) . . ?
P1 Pt1 P1 180.0 2 . ?
C7 P1 C13 104.96(7) . . ?
C7 P1 C1 111.27(7) . . ?
C13 P1 C1 104.00(7) . . ?
C7 P1 Pt1 111.34(6) . . ?
C13 P1 Pt1 111.98(5) . . ?
C1 P1 Pt1 112.80(6) . . ?
O1 N1 O2 119.94(13) . . ?
O1 N1 Pt1 119.88(10) . . ?
O2 N1 Pt1 120.11(10) . . ?
C6 C1 C2 109.36(12) . . ?
C6 C1 P1 116.57(11) . . ?
C2 C1 P1 113.13(11) . . ?
C6 C1 H1 105.6 . . ?
C2 C1 H1 105.6 . . ?
P1 C1 H1 105.6 . . ?
```

C9 C8 C7 109.60(12) . . ?
C9 C8 H8A 109.8 . . ?
C7 C8 H8A 109.8 . . ?
C9 C8 H8B 109.8 . . ?
C7 C8 H8B 109.8 . . ?
H8A C8 H8B 108.2 . . ?
C14 C13 C18 110.78(12) . . ?
C14 C13 P1 114.38(11) . . ?
C18 C13 P1 109.82(10) . . ?
C14 C13 H13 107.2 . . ?
C18 C13 H13 107.2 . . ?
P1 C13 H13 107.2 . . ?
C4 C3 C2 110.92(14) . . ?
C4 C3 H3A 109.5 . . ?
C2 C3 H3A 109.5 . . ?
C4 C3 H3B 109.5 . . ?
C2 C3 H3B 109.5 . . ?
H3A C3 H3B 108.0 . . ?
C17 C16 C15 110.83(13) . . ?
C17 C16 H16A 109.5 . . ?
C15 C16 H16A 109.5 . . ?
C17 C16 H16B 109.5 . . ?
C15 C16 H16B 109.5 . . ?
H16A C16 H16B 108.1 . . ?
C11 C12 C7 110.33(14) . . ?
C11 C12 H12A 109.6 . . ?
C7 C12 H12A 109.6 . . ?
C11 C12 H12B 109.6 . . ?
C7 C12 H12B 109.6 . . ?
H12A C12 H12B 108.1 . . ?
C17 C18 C13 110.84(13) . . ?
C17 C18 H18A 109.5 . . ?
C13 C18 H18A 109.5 . . ?
C17 C18 H18B 109.5 . . ?
C13 C18 H18B 109.5 . . ?
H18A C18 H18B 108.1 . . ?
C5 C4 C3 110.60(13) . . ?
C5 C4 H4A 109.5 . . ?
C3 C4 H4A 109.5 . . ?
C5 C4 H4B 109.5 . . ?
C3 C4 H4B 109.5 . . ?
H4A C4 H4B 108.1 . . ?
C15 C14 C13 110.73(14) . . ?
C15 C14 H14A 109.5 . . ?
C13 C14 H14A 109.5 . . ?
C15 C14 H14B 109.5 . . ?
C13 C14 H14B 109.5 . . ?
H14A C14 H14B 108.1 . . ?
C10 C11 C12 110.90(13) . . ?
C10 C11 H11A 109.5 . . ?
C12 C11 H11A 109.5 . . ?
C10 C11 H11B 109.5 . . ?
C12 C11 H11B 109.5 . . ?
H11A C11 H11B 108.0 . . ?
C16 C15 C14 110.92(14) . . ?
C16 C15 H15A 109.5 . . ?
C14 C15 H15A 109.5 . . ?

```
C16 C15 H15B 109.5 . . ?
C14 C15 H15B 109.5 . . ?
H15A C15 H15B 108.0 . . ?
C8 C7 C12 110.11(11) . . ?
C8 C7 P1 114.36(10) . . ?
C12 C7 P1 115.78(11) . . ?
C8 C7 H7 105.1 . . ?
C12 C7 H7 105.1 . . ?
P1 C7 H7 105.1 . . ?
C5 C6 C1 110.05(13) . . ?
C5 C6 H6A 109.7 . . ?
C1 C6 H6A 109.7 . . ?
C5 C6 H6B 109.7 . . ?
C1 C6 H6B 109.7 . . ?
H6A C6 H6B 108.2 . . ?
C11 C10 C9 111.21(12) . . ?
C11 C10 H10A 109.4 . . ?
C9 C10 H10A 109.4 . . ?
C11 C10 H10B 109.4 . . ?
C9 C10 H10B 109.4 . . ?
H10A C10 H10B 108.0 . . ?
C4 C5 C6 112.24(14) . . ?
C4 C5 H5A 109.2 . . ?
C6 C5 H5A 109.2 . . ?
C4 C5 H5B 109.2 . . ?
C6 C5 H5B 109.2 . . ?
H5A C5 H5B 107.9 . . ?
C8 C9 C10 111.87(14) . . ?
C8 C9 H9A 109.2 . . ?
C10 C9 H9A 109.2 . . ?
C8 C9 H9B 109.2 . . ?
C10 C9 H9B 109.2 . . ?
H9A C9 H9B 107.9 . . ?
C16 C17 C18 111.49(15) . . ?
C16 C17 H17A 109.3 . . ?
C18 C17 H17A 109.3 . . ?
C16 C17 H17B 109.3 . . ?
C18 C17 H17B 109.3 . . ?
H17A C17 H17B 108.0 . . ?
C3 C2 C1 110.22(13) . . ?
C3 C2 H2A 109.6 . . ?
C1 C2 H2A 109.6 . . ?
C3 C2 H2B 109.6 . . ?
C1 C2 H2B 109.6 . . ?
H2A C2 H2B 108.1 . . ?

loop_
 _geom_torsion_atom_site_label_1
 _geom_torsion_atom_site_label_2
 _geom_torsion_atom_site_label_3
 _geom_torsion_atom_site_label_4
 _geom_torsion
 _geom_torsion_site_symmetry_1
 _geom_torsion_site_symmetry_2
 _geom_torsion_site_symmetry_3
 _geom_torsion_site_symmetry_4
 _geom_torsion_publ_flag
```

N1 Pt1 P1 C7 36.54(6) 2 . . . ?
N1 Pt1 P1 C7 -143.46(6) . . . . ?
P1 Pt1 P1 C7 -3(3) 2 . . . ?
N1 Pt1 P1 C13 -80.63(7) 2 . . . ?
N1 Pt1 P1 C13 99.37(7) . . . . ?
P1 Pt1 P1 C13 -120(3) 2 . . . ?
N1 Pt1 P1 C1 162.46(6) 2 . . . ?
N1 Pt1 P1 C1 -17.54(6) . . . . ?
P1 Pt1 P1 C1 123(3) 2 . . . ?
N1 Pt1 N1 O1 -12(6) 2 . . . ?
P1 Pt1 N1 O1 94.19(11) 2 . . . ?
P1 Pt1 N1 O1 -85.81(11) . . . . ?
N1 Pt1 N1 O2 165(6) 2 . . . ?
P1 Pt1 N1 O2 -88.89(11) 2 . . . ?
P1 Pt1 N1 O2 91.11(11) . . . . ?
C7 P1 C1 C6 -76.40(12) . . . . ?
C13 P1 C1 C6 36.10(12) . . . . ?
Pt1 P1 C1 C6 157.64(9) . . . . ?
C7 P1 C1 C2 51.68(12) . . . . ?
C13 P1 C1 C2 164.18(10) . . . . ?
Pt1 P1 C1 C2 -74.28(11) . . . . ?
C7 P1 C13 C14 -50.56(12) . . . . ?
C1 P1 C13 C14 -167.53(11) . . . . ?
Pt1 P1 C13 C14 70.38(12) . . . . ?
C7 P1 C13 C18 -175.83(10) . . . . ?
C1 P1 C13 C18 67.20(12) . . . . ?
Pt1 P1 C13 C18 -54.89(12) . . . . ?
C14 C13 C18 C17 55.33(17) . . . . ?
P1 C13 C18 C17 -177.36(11) . . . . ?
C2 C3 C4 C5 -55.69(19) . . . . ?
C18 C13 C14 C15 -56.04(17) . . . . ?
P1 C13 C14 C15 179.20(11) . . . . ?
C7 C12 C11 C10 57.54(17) . . . . ?
C17 C16 C15 C14 -56.93(19) . . . . ?
C13 C14 C15 C16 56.92(18) . . . . ?
C9 C8 C7 C12 57.70(17) . . . . ?
C9 C8 C7 P1 -169.91(11) . . . . ?
C11 C12 C7 C8 -58.79(16) . . . . ?
C11 C12 C7 P1 169.56(10) . . . . ?
C13 P1 C7 C8 175.10(11) . . . . ?
C1 P1 C7 C8 -73.02(12) . . . . ?
Pt1 P1 C7 C8 53.74(11) . . . . ?
C13 P1 C7 C12 -55.28(12) . . . . ?
C1 P1 C7 C12 56.60(12) . . . . ?
Pt1 P1 C7 C12 -176.64(9) . . . . ?
C2 C1 C6 C5 57.89(17) . . . . ?
P1 C1 C6 C5 -172.22(10) . . . . ?
C12 C11 C10 C9 -55.62(19) . . . . ?
C3 C4 C5 C6 55.3(2) . . . . ?
C1 C6 C5 C4 -56.70(18) . . . . ?
C7 C8 C9 C10 -56.29(17) . . . . ?
C11 C10 C9 C8 55.51(18) . . . . ?
C15 C16 C17 C18 56.48(19) . . . . ?
C13 C18 C17 C16 -55.68(18) . . . . ?
C4 C3 C2 C1 58.34(18) . . . . ?
C6 C1 C2 C3 -59.13(17) . . . . ?
P1 C1 C2 C3 169.13(11) . . . . ?

```
_diffrn_measured_fraction_theta_max    0.922
_diffrn_reflns_theta_full              30.50
_diffrn_measured_fraction_theta_full   0.998
_refine_diff_density_max    0.564
_refine_diff_density_min   -0.640
_refine_diff_density_rms    0.093


data_prr98b_1

_audit_creation_method            SHELXL-97
_chemical_name_systematic
;
Bis(tricyclohexanephosphine)bis(n1-nitro/n1-nitrito)platinum (II)
;
_chemical_name_common             ?
_chemical_melting_point           ?
_chemical_formula_moiety          'C36 H66 N2 O4 P2 Pt'
_chemical_formula_sum             'C36 H66 N2 O4 P2 Pt'
_chemical_formula_weight          847.94

loop_
 _atom_type_symbol
 _atom_type_description
 _atom_type_scat_dispersion_real
 _atom_type_scat_dispersion_imag
 _atom_type_scat_source
 'C'  'C'   0.0033   0.0016
 'International Tables Vol C Tables 4.2.6.8 and 6.1.1.4'
 'H'  'H'   0.0000   0.0000
 'International Tables Vol C Tables 4.2.6.8 and 6.1.1.4'
 'N'  'N'   0.0061   0.0033
 'International Tables Vol C Tables 4.2.6.8 and 6.1.1.4'
 'O'  'O'   0.0106   0.0060
 'International Tables Vol C Tables 4.2.6.8 and 6.1.1.4'
 'P'  'P'   0.1023   0.0942
 'International Tables Vol C Tables 4.2.6.8 and 6.1.1.4'
 'Pt'  'Pt'  -1.7033   8.3905
 'International Tables Vol C Tables 4.2.6.8 and 6.1.1.4'

_symmetry_cell_setting            Triclinic
_symmetry_space_group_name_H-M    P-1


loop_
 _symmetry_equiv_pos_as_xyz
 'x, y, z'
 '-x, -y, -z'

_cell_length_a                    10.107(5)
_cell_length_b                    10.428(5)
_cell_length_c                    10.676(5)
_cell_angle_alpha                 112.184(5)
_cell_angle_beta                  110.509(5)
_cell_angle_gamma                 93.825(5)
_cell_volume                      949.8(8)
```

```
_cell_formula_units_Z             1
_cell_measurement_temperature     100(2)
_cell_measurement_reflns_used     2861
_cell_measurement_theta_min       3.5165
_cell_measurement_theta_max       32.3372

_exptl_crystal_description        block
_exptl_crystal_colour             colourless
_exptl_crystal_size_max           0.10
_exptl_crystal_size_mid           0.08
_exptl_crystal_size_min           0.08
_exptl_crystal_density_meas       ?
_exptl_crystal_density_diffrn     1.482
_exptl_crystal_density_method     'not measured'
_exptl_crystal_F_000              436
_exptl_absorpt_coefficient_mu     3.816
_exptl_absorpt_correction_T_min                    0.907
_exptl_absorpt_correction_T_max                    0.946
_exptl_absorpt_correction_type            'analytical'
_exptl_absorpt_process_details
;
CrysAlisPro, Oxford Diffraction Ltd.,
Version 1.171.33.55 (release 05-01-2010 CrysAlis171 .NET)
(compiled Jan  5 2010,16:28:46)
Analytical numeric absorption correction using a multifaceted crystal
                model based on expressions derived by R.C. Clark &
J.S. Reid.
(Clark, R. C. & Reid, J. S. (1995). Acta Cryst. A51, 887-897)
;

_diffrn_ambient_temperature       100(2)
_diffrn_radiation_wavelength      0.7107
_diffrn_radiation_type            'Mo K\a'
_diffrn_radiation_source          'Enhance (Mo) X-ray Source'
_diffrn_radiation_monochromator   'graphite'
_diffrn_measurement_device_type   'Xcalibur, Atlas, Gemini ultra'
_diffrn_measurement_method        '\w scans'
_diffrn_detector_area_resol_mean  ?
_diffrn_standards_number          ?
_diffrn_standards_interval_count  ?
_diffrn_standards_interval_time   ?
_diffrn_standards_decay_%         0
_diffrn_reflns_number             23431
_diffrn_reflns_av_R_equivalents   0.0430
_diffrn_reflns_av_sigmaI/netI     0.0669
_diffrn_reflns_limit_h_min        -17
_diffrn_reflns_limit_h_max        17
_diffrn_reflns_limit_k_min        -17
_diffrn_reflns_limit_k_max        17
_diffrn_reflns_limit_l_min        -17
_diffrn_reflns_limit_l_max        18
_diffrn_reflns_theta_min          3.43
_diffrn_reflns_theta_max          37.91
_reflns_number_total              9014
_reflns_number_gt                 8180
_reflns_threshold_expression      >2sigma(I)
_diffrn_orient_matrix_UB_11      -0.0071625000
```

```
_diffrn_orient_matrix_UB_12        0.0246179000
_diffrn_orient_matrix_UB_13       -0.0550463000
_diffrn_orient_matrix_UB_21       -0.0657979000
_diffrn_orient_matrix_UB_22       -0.0520737000
_diffrn_orient_matrix_UB_23       -0.0482959000
_diffrn_orient_matrix_UB_31       -0.0393086000
_diffrn_orient_matrix_UB_32        0.0487660000
_diffrn_orient_matrix_UB_33        0.0283607000
_diffrn_measurement_details
;
#__ type_ start__ end____ width___ exp.time_
  1 omega  -50.00   -6.00   1.0000    6.0000
omega____ theta____ kappa____ phi______ frames
    -       -32.0000  -37.0000 -146.0000 44

#__ type_ start__ end____ width___ exp.time_
  2 omega -108.00   -6.00   1.0000    6.0000
omega____ theta____ kappa____ phi______ frames
    -       -32.0000  -37.0000   66.0000 102

#__ type_ start__ end____ width___ exp.time_
  3 omega  -36.00   44.00   1.0000    6.0000
omega____ theta____ kappa____ phi______ frames
    -       -32.0000   37.0000 -174.0000 80

#__ type_ start__ end____ width___ exp.time_
  4 omega  -56.00   -6.00   1.0000    6.0000
omega____ theta____ kappa____ phi______ frames
    -       -32.0000  -37.0000 -150.0000 50

#__ type_ start__ end____ width___ exp.time_
  5 omega  -34.00   -8.00   1.0000    6.0000
omega____ theta____ kappa____ phi______ frames
    -       -32.0000   37.0000   30.0000 26

#__ type_ start__ end____ width___ exp.time_
  6 omega  -98.00   -6.00   1.0000    6.0000
omega____ theta____ kappa____ phi______ frames
    -       -32.0000  -37.0000  -40.0000 92

#__ type_ start__ end____ width___ exp.time_
  7 omega  -58.00   44.00   1.0000    6.0000
omega____ theta____ kappa____ phi______ frames
    -       -32.0000   37.0000  -83.0000 102

#__ type_ start__ end____ width___ exp.time_
  8 omega  -46.00  -21.00   1.0000    6.0000
omega____ theta____ kappa____ phi______ frames
    -       -32.0000   37.0000  150.0000 25

#__ type_ start__ end____ width___ exp.time_
  9 omega  -58.00   44.00   1.0000    6.0000
omega____ theta____ kappa____ phi______ frames
    -       -32.0000   37.0000   60.0000 102

#__ type_ start__ end____ width___ exp.time_
 10 omega  -50.00   -6.00   1.0000    6.0000
```

```
omega____ theta____ kappa____ phi______ frames
    -      -32.0000  -37.0000 -146.0000 44

#__ type_ start__ end____ width___ exp.time_
 11 omega -108.00   -6.00   1.0000    6.0000
omega____ theta____ kappa____ phi______ frames
    -      -32.0000  -37.0000   66.0000 102

#__ type_ start__ end____ width___ exp.time_
 12 omega  -36.00   44.00   1.0000    6.0000
omega____ theta____ kappa____ phi______ frames
    -      -32.0000   37.0000 -174.0000 80

#__ type_ start__ end____ width___ exp.time_
 13 omega  -56.00   -6.00   1.0000    6.0000
omega____ theta____ kappa____ phi______ frames
    -      -32.0000  -37.0000 -150.0000 50

#__ type_ start__ end____ width___ exp.time_
 14 omega  -34.00   -8.00   1.0000    6.0000
omega____ theta____ kappa____ phi______ frames
    -      -32.0000   37.0000   30.0000 26

#__ type_ start__ end____ width___ exp.time_
 15 omega  -98.00   -6.00   1.0000    6.0000
omega____ theta____ kappa____ phi______ frames
    -      -32.0000  -37.0000  -40.0000 92

#__ type_ start__ end____ width___ exp.time_
 16 omega  -58.00   44.00   1.0000    6.0000
omega____ theta____ kappa____ phi______ frames
    -      -32.0000   37.0000  -83.0000 102

#__ type_ start__ end____ width___ exp.time_
 17 omega  -46.00  -21.00   1.0000    6.0000
omega____ theta____ kappa____ phi______ frames
    -      -32.0000   37.0000  150.0000 25

#__ type_ start__ end____ width___ exp.time_
 18 omega  -58.00   44.00   1.0000    6.0000
omega____ theta____ kappa____ phi______ frames
    -      -32.0000   37.0000   60.0000 102

#__ type_ start__ end____ width___ exp.time_
 19 omega  -50.00   -6.00   1.0000    6.0000
omega____ theta____ kappa____ phi______ frames
    -      -32.0000  -37.0000 -146.0000 44

#__ type_ start__ end____ width___ exp.time_
 20 omega -108.00   -6.00   1.0000    6.0000
omega____ theta____ kappa____ phi______ frames
    -      -32.0000  -37.0000   66.0000 102

#__ type_ start__ end____ width___ exp.time_
 21 omega  -36.00   44.00   1.0000    6.0000
omega____ theta____ kappa____ phi______ frames
    -      -32.0000   37.0000 -174.0000 80
```

```
#__ type_ start__ end____ width___ exp.time_
 22 omega  -56.00   -6.00   1.0000    6.0000
omega____ theta____ kappa____ phi______ frames
    -      -32.0000  -37.0000 -150.0000 50

#__ type_ start__ end____ width___ exp.time_
 23 omega  -34.00   -8.00   1.0000    6.0000
omega____ theta____ kappa____ phi______ frames
    -      -32.0000   37.0000   30.0000 26

#__ type_ start__ end____ width___ exp.time_
 24 omega  -98.00   -6.00   1.0000    6.0000
omega____ theta____ kappa____ phi______ frames
    -      -32.0000  -37.0000  -40.0000 92

#__ type_ start__ end____ width___ exp.time_
 25 omega  -58.00   44.00   1.0000    6.0000
omega____ theta____ kappa____ phi______ frames
    -      -32.0000   37.0000  -83.0000 102

#__ type_ start__ end____ width___ exp.time_
 26 omega  -46.00  -21.00   1.0000    6.0000
omega____ theta____ kappa____ phi______ frames
    -      -32.0000   37.0000  150.0000 25

#__ type_ start__ end____ width___ exp.time_
 27 omega  -58.00   44.00   1.0000    6.0000
omega____ theta____ kappa____ phi______ frames
    -      -32.0000   37.0000   60.0000 102

#__ type_ start__ end____ width___ exp.time_
 28 omega  -50.00   -6.00   1.0000    6.0000
omega____ theta____ kappa____ phi______ frames
    -      -32.0000  -37.0000 -146.0000 44

#__ type_ start__ end____ width___ exp.time_
 29 omega -108.00   -6.00   1.0000    6.0000
omega____ theta____ kappa____ phi______ frames
    -      -32.0000  -37.0000   66.0000 102

#__ type_ start__ end____ width___ exp.time_
 30 omega  -36.00   44.00   1.0000    6.0000
omega____ theta____ kappa____ phi______ frames
    -      -32.0000   37.0000 -174.0000 80

#__ type_ start__ end____ width___ exp.time_
 31 omega  -56.00   -6.00   1.0000    6.0000
omega____ theta____ kappa____ phi______ frames
    -      -32.0000  -37.0000 -150.0000 50

#__ type_ start__ end____ width___ exp.time_
 32 omega  -34.00   -8.00   1.0000    6.0000
omega____ theta____ kappa____ phi______ frames
    -      -32.0000   37.0000   30.0000 26

#__ type_ start__ end____ width___ exp.time_
```

```
 33 omega  -98.00   -6.00   1.0000    6.0000
omega____ theta____ kappa____ phi______ frames
    -      -32.0000  -37.0000  -40.0000 92

#__ type_ start__ end____ width___ exp.time_
 34 omega  -58.00   44.00   1.0000    6.0000
omega____ theta____ kappa____ phi______ frames
    -      -32.0000   37.0000  -83.0000 102

#__ type_ start__ end____ width___ exp.time_
 35 omega  -46.00  -21.00   1.0000    6.0000
omega____ theta____ kappa____ phi______ frames
    -      -32.0000   37.0000  150.0000 25

#__ type_ start__ end____ width___ exp.time_
 36 omega  -58.00   44.00   1.0000    6.0000
omega____ theta____ kappa____ phi______ frames
    -      -32.0000   37.0000   60.0000 102

#__ type_ start__ end____ width___ exp.time_
 37 omega  -50.00   -6.00   1.0000    6.0000
omega____ theta____ kappa____ phi______ frames
    -      -32.0000  -37.0000 -146.0000 44

#__ type_ start__ end____ width___ exp.time_
 38 omega -108.00   -6.00   1.0000    6.0000
omega____ theta____ kappa____ phi______ frames
    -      -32.0000  -37.0000   66.0000 102

#__ type_ start__ end____ width___ exp.time_
 39 omega  -36.00   44.00   1.0000    6.0000
omega____ theta____ kappa____ phi______ frames
    -      -32.0000   37.0000 -174.0000 80

#__ type_ start__ end____ width___ exp.time_
 40 omega  -56.00   -6.00   1.0000    6.0000
omega____ theta____ kappa____ phi______ frames
    -      -32.0000  -37.0000 -150.0000 50

#__ type_ start__ end____ width___ exp.time_
 41 omega  -34.00   -8.00   1.0000    6.0000
omega____ theta____ kappa____ phi______ frames
    -      -32.0000   37.0000   30.0000 26

#__ type_ start__ end____ width___ exp.time_
 42 omega  -98.00   -6.00   1.0000    6.0000
omega____ theta____ kappa____ phi______ frames
    -      -32.0000  -37.0000  -40.0000 92

#__ type_ start__ end____ width___ exp.time_
 43 omega  -58.00   17.00   1.0000    6.0000
omega____ theta____ kappa____ phi______ frames
    -      -32.0000   37.0000  -83.0000 75
;

_computing_data_collection
;
```

```
CrysAlisPro, Oxford Diffraction Ltd.,
Version 1.171.33.55 (release 05-01-2010 CrysAlis171 .NET)
(compiled Jan  5 2010,16:28:46)
;
_computing_cell_refinement
;
CrysAlisPro, Oxford Diffraction Ltd.,
Version 1.171.33.55 (release 05-01-2010 CrysAlis171 .NET)
(compiled Jan  5 2010,16:28:46)
;
_computing_data_reduction
;
CrysAlisPro, Oxford Diffraction Ltd.,
Version 1.171.33.55 (release 05-01-2010 CrysAlis171 .NET)
(compiled Jan  5 2010,16:28:46)
;
_computing_structure_solution   'SHELXTS (Sheldrick, 1997)'
_computing_structure_refinement 'SHELXTL (Sheldrick, 1997)'
_computing_molecular_graphics   'ORTEP'
_computing_publication_material 'SHELXTL (Sheldrick, 1997)'


_refine_special_details
;
 Refinement of F^2^ against ALL reflections.  The weighted R-factor wR
and
 goodness of fit S are based on F^2^, conventional R-factors R are
based
 on F, with F set to zero for negative F^2^. The threshold expression
of
 F^2^ > 2sigma(F^2^) is used only for calculating R-factors(gt) etc.
and is
 not relevant to the choice of reflections for refinement.  R-factors
based
 on F^2^ are statistically about twice as large as those based on F,
and R-
 factors based on ALL data will be even larger.
;

_refine_ls_structure_factor_coef  Fsqd
_refine_ls_matrix_type            full
_refine_ls_weighting_scheme       calc
_refine_ls_weighting_details
 'calc w=1/[\s^2^(Fo^2^)+(0.0239P)^2^+0.0000P] where
P=(Fo^2^+2Fc^2^)/3'
_atom_sites_solution_primary      direct
_atom_sites_solution_secondary    difmap
_atom_sites_solution_hydrogens    geom
_refine_ls_hydrogen_treatment     constr
_refine_ls_extinction_method      none
_refine_ls_extinction_coef        ?
_refine_ls_number_reflns          9014
_refine_ls_number_parameters      218
_refine_ls_number_restraints      7
_refine_ls_R_factor_all           0.0402
_refine_ls_R_factor_gt            0.0340
_refine_ls_wR_factor_ref          0.0572
```

```
_refine_ls_wR_factor_gt           0.0559
_refine_ls_goodness_of_fit_ref    0.903
_refine_ls_restrained_S_all       0.907
_refine_ls_shift/su_max           0.005
_refine_ls_shift/su_mean          0.000

loop_
 _atom_site_label
 _atom_site_type_symbol
 _atom_site_fract_x
 _atom_site_fract_y
 _atom_site_fract_z
 _atom_site_U_iso_or_equiv
 _atom_site_adp_type
 _atom_site_occupancy
 _atom_site_symmetry_multiplicity
 _atom_site_calc_flag
 _atom_site_refinement_flags
 _atom_site_disorder_assembly
 _atom_site_disorder_group
Pt1 Pt 0.0000 0.0000 0.0000 0.01871(3) Uani 1 2 d SD . .
P1 P -0.20680(5) -0.03306(5) -0.21029(5) 0.01694(9) Uani 1 1 d . A .
O1 O 0.1854(3) -0.0206(4) -0.1530(3) 0.0302(8) Uiso 0.720(4) 1 d PD A
1
O2 O 0.1665(3) 0.1888(3) -0.0573(3) 0.0263(6) Uiso 0.720(4) 1 d PD A 1
N1 N 0.1314(2) 0.0612(3) -0.0827(3) 0.0156(4) Uiso 0.720(4) 1 d PD A 1
C1 C -0.1729(2) -0.10409(19) -0.3796(2) 0.0199(3) Uani 1 1 d . . .
H1 H -0.0926 -0.0300 -0.3632 0.024 Uiso 1 1 calc R A .
C2 C -0.1144(2) -0.2403(2) -0.4056(2) 0.0235(4) Uani 1 1 d . A .
H2A H -0.0316 -0.2268 -0.3142 0.028 Uiso 1 1 calc R . .
H2B H -0.1913 -0.3208 -0.4304 0.028 Uiso 1 1 calc R . .
C3 C -0.0650(2) -0.2740(2) -0.5326(2) 0.0277(4) Uani 1 1 d . . .
H3A H 0.0185 -0.1975 -0.5029 0.033 Uiso 1 1 calc R A .
H3B H -0.0323 -0.3642 -0.5521 0.033 Uiso 1 1 calc R . .
C4 C -0.1872(2) -0.2872(2) -0.6745(2) 0.0288(4) Uani 1 1 d . A .
H4A H -0.1495 -0.3023 -0.7518 0.035 Uiso 1 1 calc R . .
H4B H -0.2659 -0.3709 -0.7110 0.035 Uiso 1 1 calc R . .
C5 C -0.2478(2) -0.1546(2) -0.6475(2) 0.0276(4) Uani 1 1 d . . .
H5A H -0.3304 -0.1685 -0.7392 0.033 Uiso 1 1 calc R A .
H5B H -0.1720 -0.0727 -0.6217 0.033 Uiso 1 1 calc R . .
C6 C -0.2988(2) -0.1223(2) -0.5223(2) 0.0246(4) Uani 1 1 d . A .
H6A H -0.3795 -0.2011 -0.5507 0.030 Uiso 1 1 calc R . .
H6B H -0.3352 -0.0341 -0.5047 0.030 Uiso 1 1 calc R . .
C7 C -0.2589(2) 0.13672(18) -0.1999(2) 0.0200(3) Uani 1 1 d . . .
H7 H -0.3556 0.1122 -0.2834 0.024 Uiso 1 1 calc R A .
C8 C -0.1541(2) 0.2361(2) -0.2163(2) 0.0263(4) Uani 1 1 d . A .
H8A H -0.0562 0.2608 -0.1365 0.032 Uiso 1 1 calc R . .
H8B H -0.1471 0.1869 -0.3124 0.032 Uiso 1 1 calc R . .
C9 C -0.2066(3) 0.3719(2) -0.2081(3) 0.0315(5) Uani 1 1 d . . .
H9A H -0.1359 0.4361 -0.2154 0.038 Uiso 1 1 calc R A .
H9B H -0.3008 0.3477 -0.2927 0.038 Uiso 1 1 calc R . .
C10 C -0.2240(3) 0.4481(2) -0.0638(3) 0.0322(5) Uani 1 1 d . A .
H10A H -0.1283 0.4795 0.0206 0.039 Uiso 1 1 calc R . .
H10B H -0.2614 0.5336 -0.0630 0.039 Uiso 1 1 calc R . .
C11 C -0.3279(3) 0.3508(2) -0.0464(3) 0.0327(5) Uani 1 1 d . . .
H11A H -0.4260 0.3273 -0.1253 0.039 Uiso 1 1 calc R A .
H11B H -0.3337 0.4009 0.0502 0.039 Uiso 1 1 calc R . .
```

```
C12 C -0.2784(2) 0.2129(2) -0.0551(2) 0.0265(4) Uani 1 1 d . A .
H12A H -0.3514 0.1491 -0.0500 0.032 Uiso 1 1 calc R . .
H12B H -0.1854 0.2353 0.0303 0.032 Uiso 1 1 calc R . .
C13 C -0.3722(2) -0.14563(19) -0.2329(2) 0.0192(3) Uani 1 1 d . . .
H13 H -0.3691 -0.1142 -0.1308 0.023 Uiso 1 1 calc R A .
C14 C -0.5194(2) -0.1286(2) -0.3254(2) 0.0262(4) Uani 1 1 d . A .
H14A H -0.5217 -0.0266 -0.2889 0.031 Uiso 1 1 calc R . .
H14B H -0.5326 -0.1642 -0.4301 0.031 Uiso 1 1 calc R . .
C15 C -0.6425(2) -0.2116(2) -0.3149(3) 0.0323(5) Uani 1 1 d . . .
H15A H -0.6340 -0.1694 -0.2116 0.039 Uiso 1 1 calc R A .
H15B H -0.7366 -0.2028 -0.3786 0.039 Uiso 1 1 calc R . .
C16 C -0.6405(2) -0.3679(2) -0.3621(3) 0.0331(5) Uani 1 1 d . A .
H16A H -0.6610 -0.4135 -0.4689 0.040 Uiso 1 1 calc R . .
H16B H -0.7173 -0.4160 -0.3467 0.040 Uiso 1 1 calc R . .
C17 C -0.4935(2) -0.3855(2) -0.2735(3) 0.0301(5) Uani 1 1 d . . .
H17A H -0.4921 -0.4877 -0.3106 0.036 Uiso 1 1 calc R A .
H17B H -0.4780 -0.3500 -0.1682 0.036 Uiso 1 1 calc R . .
C18 C -0.3709(2) -0.3039(2) -0.2860(2) 0.0237(4) Uani 1 1 d . A .
H18A H -0.3824 -0.3440 -0.3903 0.028 Uiso 1 1 calc R . .
H18B H -0.2764 -0.3149 -0.2255 0.028 Uiso 1 1 calc R . .
O1A O 0.1290(15) -0.014(2) -0.1037(17) 0.052(5) Uiso 0.134(5) 1 d PD A
2
O2A O 0.1528(15) 0.2135(15) -0.0250(17) 0.026(4) Uiso 0.134(5) 1 d P A
2
N1A N 0.1911(17) 0.121(3) -0.100(2) 0.029(3) Uiso 0.134(5) 1 d P A 2
O1B O 0.1788(17) -0.0541(16) -0.1544(17) 0.031(4) Uiso 0.146(5) 1 d P
A 3
O2B O 0.1138(13) 0.1144(14) -0.0531(14) 0.038(4) Uiso 0.146(5) 1 d PD
A 3
N1B N 0.1997(19) 0.060(4) -0.129(2) 0.045(4) Uiso 0.146(5) 1 d P A 3

loop_
 _atom_site_aniso_label
 _atom_site_aniso_U_11
 _atom_site_aniso_U_22
 _atom_site_aniso_U_33
 _atom_site_aniso_U_23
 _atom_site_aniso_U_13
 _atom_site_aniso_U_12
Pt1 0.01962(5) 0.02084(5) 0.01463(5) 0.00794(4) 0.00597(4) 0.00173(3)
P1 0.0201(2) 0.01515(19) 0.0144(2) 0.00598(16) 0.00638(17) 0.00303(16)
C1 0.0234(9) 0.0196(8) 0.0159(8) 0.0071(7) 0.0080(7) 0.0041(7)
C2 0.0290(10) 0.0210(9) 0.0192(9) 0.0073(7) 0.0097(8) 0.0063(7)
C3 0.0281(10) 0.0271(10) 0.0270(10) 0.0075(8) 0.0148(9) 0.0066(8)
C4 0.0349(11) 0.0281(10) 0.0199(9) 0.0040(8) 0.0153(9) 0.0002(8)
C5 0.0327(11) 0.0306(10) 0.0174(9) 0.0107(8) 0.0080(8) 0.0032(8)
C6 0.0282(10) 0.0278(9) 0.0168(8) 0.0095(7) 0.0079(8) 0.0075(8)
C7 0.0227(9) 0.0156(8) 0.0192(8) 0.0067(7) 0.0069(7) 0.0038(6)
C8 0.0318(11) 0.0195(9) 0.0305(10) 0.0118(8) 0.0148(9) 0.0052(8)
C9 0.0370(12) 0.0199(9) 0.0350(12) 0.0145(9) 0.0094(10) 0.0039(8)
C10 0.0360(12) 0.0167(9) 0.0314(11) 0.0063(8) 0.0046(9) 0.0037(8)
C11 0.0379(12) 0.0194(9) 0.0342(12) 0.0043(8) 0.0150(10) 0.0085(8)
C12 0.0347(11) 0.0198(9) 0.0259(10) 0.0077(8) 0.0154(9) 0.0075(8)
C13 0.0202(8) 0.0175(8) 0.0187(8) 0.0077(7) 0.0069(7) 0.0035(6)
C14 0.0214(9) 0.0237(9) 0.0302(10) 0.0133(8) 0.0050(8) 0.0042(7)
C15 0.0209(10) 0.0336(11) 0.0407(13) 0.0166(10) 0.0100(9) 0.0059(8)
C16 0.0249(10) 0.0288(10) 0.0426(13) 0.0149(10) 0.0122(10) -0.0001(8)
```

```
C17 0.0293(11) 0.0244(10) 0.0398(12) 0.0179(9) 0.0136(9) 0.0037(8)
C18 0.0230(9) 0.0198(8) 0.0273(10) 0.0106(7) 0.0091(8) 0.0032(7)

_geom_special_details
;
 All esds (except the esd in the dihedral angle between two l.s.
planes)
 are estimated using the full covariance matrix.  The cell esds are
taken
 into account individually in the estimation of esds in distances,
angles
 and torsion angles; correlations between esds in cell parameters are
only
 used when they are defined by crystal symmetry.  An approximate
(isotropic)
 treatment of cell esds is used for estimating esds involving l.s.
planes.
;

loop_
 _geom_bond_atom_site_label_1
 _geom_bond_atom_site_label_2
 _geom_bond_distance
 _geom_bond_site_symmetry_2
 _geom_bond_publ_flag
Pt1 O2B 1.963(8) . ?
Pt1 O2B 1.963(8) 2 ?
Pt1 O1A 1.965(9) 2 ?
Pt1 O1A 1.965(9) . ?
Pt1 N1 2.021(2) 2 ?
Pt1 N1 2.021(2) . ?
Pt1 P1 2.3577(10) . ?
Pt1 P1 2.3578(10) 2 ?
P1 C1 1.841(2) . ?
P1 C7 1.855(2) . ?
P1 C13 1.864(2) . ?
O1 N1 1.207(5) . ?
O2 N1 1.252(4) . ?
C1 C6 1.539(3) . ?
C1 C2 1.541(3) . ?
C1 H1 1.0000 . ?
C2 C3 1.533(3) . ?
C2 H2A 0.9900 . ?
C2 H2B 0.9900 . ?
C3 C4 1.533(3) . ?
C3 H3A 0.9900 . ?
C3 H3B 0.9900 . ?
C4 C5 1.518(3) . ?
C4 H4A 0.9900 . ?
C4 H4B 0.9900 . ?
C5 C6 1.527(3) . ?
C5 H5A 0.9900 . ?
C5 H5B 0.9900 . ?
C6 H6A 0.9900 . ?
C6 H6B 0.9900 . ?
C7 C8 1.532(3) . ?
C7 C12 1.538(3) . ?
```

```
C7 H7 1.0000 . ?
C8 C9 1.527(3) . ?
C8 H8A 0.9900 . ?
C8 H8B 0.9900 . ?
C9 C10 1.523(3) . ?
C9 H9A 0.9900 . ?
C9 H9B 0.9900 . ?
C10 C11 1.518(3) . ?
C10 H10A 0.9900 . ?
C10 H10B 0.9900 . ?
C11 C12 1.535(3) . ?
C11 H11A 0.9900 . ?
C11 H11B 0.9900 . ?
C12 H12A 0.9900 . ?
C12 H12B 0.9900 . ?
C13 C18 1.532(3) . ?
C13 C14 1.535(3) . ?
C13 H13 1.0000 . ?
C14 C15 1.529(3) . ?
C14 H14A 0.9900 . ?
C14 H14B 0.9900 . ?
C15 C16 1.518(3) . ?
C15 H15A 0.9900 . ?
C15 H15B 0.9900 . ?
C16 C17 1.523(3) . ?
C16 H16A 0.9900 . ?
C16 H16B 0.9900 . ?
C17 C18 1.529(3) . ?
C17 H17A 0.9900 . ?
C17 H17B 0.9900 . ?
C18 H18A 0.9900 . ?
C18 H18B 0.9900 . ?
O1A N1A 1.48(3) . ?
O2A N1A 1.18(3) . ?
O1B N1B 1.10(3) . ?
O2B N1B 1.40(3) . ?

loop_
 _geom_angle_atom_site_label_1
 _geom_angle_atom_site_label_2
 _geom_angle_atom_site_label_3
 _geom_angle
 _geom_angle_site_symmetry_1
 _geom_angle_site_symmetry_3
 _geom_angle_publ_flag
O2B Pt1 O2B 179.999(1) . 2 ?
O2B Pt1 O1A 141.9(6) . 2 ?
O2B Pt1 O1A 38.1(6) 2 2 ?
O2B Pt1 O1A 38.1(6) . . ?
O2B Pt1 O1A 141.9(6) 2 . ?
O1A Pt1 O1A 179.999(1) 2 . ?
O2B Pt1 N1 162.8(4) . 2 ?
O2B Pt1 N1 17.2(4) 2 2 ?
O1A Pt1 N1 20.9(5) 2 2 ?
O1A Pt1 N1 159.1(5) . 2 ?
O2B Pt1 N1 17.2(4) . . ?
O2B Pt1 N1 162.8(4) 2 . ?
```

O1A Pt1 N1 159.1(5) 2 . ?
O1A Pt1 N1 20.9(5) . . ?
N1 Pt1 N1 180.0 2 . ?
O2B Pt1 P1 89.0(4) . . ?
O2B Pt1 P1 91.0(4) 2 . ?
O1A Pt1 P1 87.1(5) 2 . ?
O1A Pt1 P1 92.9(5) . . ?
N1 Pt1 P1 89.15(7) 2 . ?
N1 Pt1 P1 90.85(7) . . ?
O2B Pt1 P1 91.0(4) . 2 ?
O2B Pt1 P1 89.0(4) 2 2 ?
O1A Pt1 P1 92.9(5) 2 2 ?
O1A Pt1 P1 87.1(5) . 2 ?
N1 Pt1 P1 90.85(7) 2 2 ?
N1 Pt1 P1 89.15(7) . 2 ?
P1 Pt1 P1 180.0 . 2 ?
C1 P1 C7 104.71(9) . . ?
C1 P1 C13 110.86(9) . . ?
C7 P1 C13 104.01(9) . . ?
C1 P1 Pt1 111.39(7) . . ?
C7 P1 Pt1 112.28(6) . . ?
C13 P1 Pt1 113.06(7) . . ?
O1 N1 O2 116.3(3) . . ?
O1 N1 Pt1 122.5(3) . . ?
O2 N1 Pt1 121.2(3) . . ?
C6 C1 C2 110.19(15) . . ?
C6 C1 P1 115.50(14) . . ?
C2 C1 P1 114.57(13) . . ?
C6 C1 H1 105.1 . . ?
C2 C1 H1 105.1 . . ?
P1 C1 H1 105.1 . . ?
C3 C2 C1 109.72(16) . . ?
C3 C2 H2A 109.7 . . ?
C1 C2 H2A 109.7 . . ?
C3 C2 H2B 109.7 . . ?
C1 C2 H2B 109.7 . . ?
H2A C2 H2B 108.2 . . ?
C4 C3 C2 111.71(18) . . ?
C4 C3 H3A 109.3 . . ?
C2 C3 H3A 109.3 . . ?
C4 C3 H3B 109.3 . . ?
C2 C3 H3B 109.3 . . ?
H3A C3 H3B 107.9 . . ?
C5 C4 C3 111.23(16) . . ?
C5 C4 H4A 109.4 . . ?
C3 C4 H4A 109.4 . . ?
C5 C4 H4B 109.4 . . ?
C3 C4 H4B 109.4 . . ?
H4A C4 H4B 108.0 . . ?
C4 C5 C6 111.08(17) . . ?
C4 C5 H5A 109.4 . . ?
C6 C5 H5A 109.4 . . ?
C4 C5 H5B 109.4 . . ?
C6 C5 H5B 109.4 . . ?
H5A C5 H5B 108.0 . . ?
C5 C6 C1 110.19(17) . . ?
C5 C6 H6A 109.6 . . ?

C1 C6 H6A 109.6 . . ?
C5 C6 H6B 109.6 . . ?
C1 C6 H6B 109.6 . . ?
H6A C6 H6B 108.1 . . ?
C8 C7 C12 110.80(16) . . ?
C8 C7 P1 114.28(14) . . ?
C12 C7 P1 109.64(13) . . ?
C8 C7 H7 107.3 . . ?
C12 C7 H7 107.3 . . ?
P1 C7 H7 107.3 . . ?
C9 C8 C7 110.55(18) . . ?
C9 C8 H8A 109.5 . . ?
C7 C8 H8A 109.5 . . ?
C9 C8 H8B 109.5 . . ?
C7 C8 H8B 109.5 . . ?
H8A C8 H8B 108.1 . . ?
C10 C9 C8 111.19(18) . . ?
C10 C9 H9A 109.4 . . ?
C8 C9 H9A 109.4 . . ?
C10 C9 H9B 109.4 . . ?
C8 C9 H9B 109.4 . . ?
H9A C9 H9B 108.0 . . ?
C11 C10 C9 110.79(17) . . ?
C11 C10 H10A 109.5 . . ?
C9 C10 H10A 109.5 . . ?
C11 C10 H10B 109.5 . . ?
C9 C10 H10B 109.5 . . ?
H10A C10 H10B 108.1 . . ?
C10 C11 C12 111.39(19) . . ?
C10 C11 H11A 109.3 . . ?
C12 C11 H11A 109.3 . . ?
C10 C11 H11B 109.3 . . ?
C12 C11 H11B 109.3 . . ?
H11A C11 H11B 108.0 . . ?
C11 C12 C7 110.84(17) . . ?
C11 C12 H12A 109.5 . . ?
C7 C12 H12A 109.5 . . ?
C11 C12 H12B 109.5 . . ?
C7 C12 H12B 109.5 . . ?
H12A C12 H12B 108.1 . . ?
C18 C13 C14 109.52(15) . . ?
C18 C13 P1 113.42(14) . . ?
C14 C13 P1 116.88(14) . . ?
C18 C13 H13 105.3 . . ?
C14 C13 H13 105.3 . . ?
P1 C13 H13 105.3 . . ?
C15 C14 C13 110.25(17) . . ?
C15 C14 H14A 109.6 . . ?
C13 C14 H14A 109.6 . . ?
C15 C14 H14B 109.6 . . ?
C13 C14 H14B 109.6 . . ?
H14A C14 H14B 108.1 . . ?
C16 C15 C14 112.25(19) . . ?
C16 C15 H15A 109.2 . . ?
C14 C15 H15A 109.2 . . ?
C16 C15 H15B 109.2 . . ?
C14 C15 H15B 109.2 . . ?

```
H15A C15 H15B 107.9 . . ?
C15 C16 C17 110.71(18) . . ?
C15 C16 H16A 109.5 . . ?
C17 C16 H16A 109.5 . . ?
C15 C16 H16B 109.5 . . ?
C17 C16 H16B 109.5 . . ?
H16A C16 H16B 108.1 . . ?
C16 C17 C18 111.00(18) . . ?
C16 C17 H17A 109.4 . . ?
C18 C17 H17A 109.4 . . ?
C16 C17 H17B 109.4 . . ?
C18 C17 H17B 109.4 . . ?
H17A C17 H17B 108.0 . . ?
C17 C18 C13 110.75(17) . . ?
C17 C18 H18A 109.5 . . ?
C13 C18 H18A 109.5 . . ?
C17 C18 H18B 109.5 . . ?
C13 C18 H18B 109.5 . . ?
H18A C18 H18B 108.1 . . ?
N1A O1A Pt1 115.8(15) . . ?
O2A N1A O1A 107.8(14) . . ?
N1B O2B Pt1 123.1(15) . . ?
O1B N1B O2B 104.1(16) . . ?

loop_
 _geom_torsion_atom_site_label_1
 _geom_torsion_atom_site_label_2
 _geom_torsion_atom_site_label_3
 _geom_torsion_atom_site_label_4
 _geom_torsion
 _geom_torsion_site_symmetry_1
 _geom_torsion_site_symmetry_2
 _geom_torsion_site_symmetry_3
 _geom_torsion_site_symmetry_4
 _geom_torsion_publ_flag
O2B Pt1 P1 C1 53.6(4) . . . . ?
O2B Pt1 P1 C1 -126.4(4) 2 . . . ?
O1A Pt1 P1 C1 -164.3(6) 2 . . . ?
O1A Pt1 P1 C1 15.7(6) . . . . ?
N1 Pt1 P1 C1 -143.50(11) 2 . . . ?
N1 Pt1 P1 C1 36.50(11) . . . . ?
P1 Pt1 P1 C1 -129(5) 2 . . . ?
O2B Pt1 P1 C7 -63.4(4) . . . . ?
O2B Pt1 P1 C7 116.6(4) 2 . . . ?
O1A Pt1 P1 C7 78.6(6) 2 . . . ?
O1A Pt1 P1 C7 -101.4(6) . . . . ?
N1 Pt1 P1 C7 99.42(11) 2 . . . ?
N1 Pt1 P1 C7 -80.58(11) . . . . ?
P1 Pt1 P1 C7 114(5) 2 . . . ?
O2B Pt1 P1 C13 179.3(4) . . . . ?
O2B Pt1 P1 C13 -0.7(4) 2 . . . ?
O1A Pt1 P1 C13 -38.7(6) 2 . . . ?
O1A Pt1 P1 C13 141.3(6) . . . . ?
N1 Pt1 P1 C13 -17.88(10) 2 . . . ?
N1 Pt1 P1 C13 162.12(10) . . . . ?
P1 Pt1 P1 C13 -3(5) 2 . . . ?
O2B Pt1 N1 O1 -178.5(14) . . . . ?
```

O2B Pt1 N1 O1 1.5(14) 2 . . . ?
O1A Pt1 N1 O1 -178.6(13) 2 . . . ?
O1A Pt1 N1 O1 1.4(13) . . . . ?
N1 Pt1 N1 O1 26(5) 2 . . . ?
P1 Pt1 N1 O1 -94.5(2) . . . . ?
P1 Pt1 N1 O1 85.5(2) 2 . . . ?
O2B Pt1 N1 O2 4.9(13) . . . . ?
O2B Pt1 N1 O2 -175.1(13) 2 . . . ?
O1A Pt1 N1 O2 4.7(14) 2 . . . ?
O1A Pt1 N1 O2 -175.3(14) . . . . ?
N1 Pt1 N1 O2 -151(5) 2 . . . ?
P1 Pt1 N1 O2 88.9(2) . . . . ?
P1 Pt1 N1 O2 -91.1(2) 2 . . . ?
C7 P1 C1 C6 -55.39(16) . . . . ?
C13 P1 C1 C6 56.19(16) . . . . ?
Pt1 P1 C1 C6 -176.98(12) . . . . ?
C7 P1 C1 C2 174.93(14) . . . . ?
C13 P1 C1 C2 -73.49(16) . . . . ?
Pt1 P1 C1 C2 53.34(15) . . . . ?
C6 C1 C2 C3 57.8(2) . . . . ?
P1 C1 C2 C3 -169.95(14) . . . . ?
C1 C2 C3 C4 -56.0(2) . . . . ?
C2 C3 C4 C5 55.2(2) . . . . ?
C3 C4 C5 C6 -55.6(2) . . . . ?
C4 C5 C6 C1 57.7(2) . . . . ?
C2 C1 C6 C5 -58.9(2) . . . . ?
P1 C1 C6 C5 169.37(14) . . . . ?
C1 P1 C7 C8 -50.40(16) . . . . ?
C13 P1 C7 C8 -166.82(14) . . . . ?
Pt1 P1 C7 C8 70.60(15) . . . . ?
C1 P1 C7 C12 -175.47(13) . . . . ?
C13 P1 C7 C12 68.11(15) . . . . ?
Pt1 P1 C7 C12 -54.47(15) . . . . ?
C12 C7 C8 C9 -56.2(2) . . . . ?
P1 C7 C8 C9 179.35(14) . . . . ?
C7 C8 C9 C10 57.2(2) . . . . ?
C8 C9 C10 C11 -57.1(3) . . . . ?
C9 C10 C11 C12 56.2(3) . . . . ?
C10 C11 C12 C7 -55.5(2) . . . . ?
C8 C7 C12 C11 55.3(2) . . . . ?
P1 C7 C12 C11 -177.61(15) . . . . ?
C1 P1 C13 C18 52.22(16) . . . . ?
C7 P1 C13 C18 164.25(14) . . . . ?
Pt1 P1 C13 C18 -73.69(14) . . . . ?
C1 P1 C13 C14 -76.67(16) . . . . ?
C7 P1 C13 C14 35.36(17) . . . . ?
Pt1 P1 C13 C14 157.43(13) . . . . ?
C18 C13 C14 C15 57.3(2) . . . . ?
P1 C13 C14 C15 -171.96(14) . . . . ?
C13 C14 C15 C16 -56.4(2) . . . . ?
C14 C15 C16 C17 55.1(3) . . . . ?
C15 C16 C17 C18 -55.2(3) . . . . ?
C16 C17 C18 C13 57.7(2) . . . . ?
C14 C13 C18 C17 -58.5(2) . . . . ?
P1 C13 C18 C17 168.96(14) . . . . ?
O2B Pt1 O1A N1A 3.1(9) . . . . ?
O2B Pt1 O1A N1A -176.9(9) 2 . . . ?

```
O1A Pt1 O1A N1A -6(4) 2 . . . ?
N1 Pt1 O1A N1A -176.9(7) 2 . . . ?
N1 Pt1 O1A N1A 3.1(7) . . . . ?
P1 Pt1 O1A N1A 87.9(11) . . . . ?
P1 Pt1 O1A N1A -92.1(11) 2 . . . ?
Pt1 O1A N1A O2A 3.4(17) . . . . ?
O2B Pt1 O2B N1B -174(3) 2 . . . ?
O1A Pt1 O2B N1B 177.8(12) 2 . . . ?
O1A Pt1 O2B N1B -2.2(12) . . . . ?
N1 Pt1 O2B N1B 177.9(10) 2 . . . ?
N1 Pt1 O2B N1B -2.1(10) . . . . ?
P1 Pt1 O2B N1B -98.1(12) . . . . ?
P1 Pt1 O2B N1B 81.9(12) 2 . . . ?
Pt1 O2B N1B O1B 4.1(19) . . . . ?

_diffrn_measured_fraction_theta_max    0.876
_diffrn_reflns_theta_full              30.50
_diffrn_measured_fraction_theta_full   0.969
_refine_diff_density_max    2.205
_refine_diff_density_min   -0.883
_refine_diff_density_rms    0.134
```
